# The efficacy of an embryonic stem cell-based vaccine for lung cancer prevention depends on the undifferentiated state of the stem cells

**DOI:** 10.1038/s41598-024-83932-0

**Published:** 2024-12-30

**Authors:** Shuhan Meng, Aaron G. Whitt, John W. Eaton, Kavitha Yaddanapudi, Chi Li

**Affiliations:** 1 Department of Pharmacology and Toxicology, University of Louisville, Louisville, KY USA; 2 Experimental Therapeutics Group, Brown Cancer Center, Department of Medicine, University of Louisville, Louisville, KY USA; 3 Department of Microbiology and Immunology, University of Louisville, Louisville, KY USA; 4 Immuno-Oncology Program, Brown Cancer Center, Department of Medicine, University of Louisville, Louisville, KY USA; 5 Division of Immunotherapy, Department of Surgery, University of Louisville, Louisville, KY USA

**Keywords:** Cancer, Vaccine, Embryonic stem cells, Differentiation, Granulocyte macrophage-colony stimulating factor

## Abstract

Based on the antigenic similarity between tumor cells and embryonic stem cells (ESCs), several recent studies report the use of intact murine ESCs or exosomes from murine ESCs as cancer vaccines. Since the capacity for self-renewal is one of the most specialized properties shared between ESCs and a subset of tumor cells, cancer stem cells (CSCs), we investigated whether the undifferentiated state of murine ESCs is essential for the prophylactic effectiveness of an ESC-based vaccine. The undifferentiated state of ES-D3, a murine ESC line, was essential for their anchorage-independent growth potential. Importantly, differentiation of ES-D3 cells decreased their efficacy in preventing the outgrowth of implanted lung tumors. Furthermore, the long-term cancer-preventive potential of this vaccine was also inhibited by the differentiation of these cells. To examine the antigenicity of the ESC-derived vaccine, we performed combined affinity chromatography shotgun immunoproteomic experiments to identify antigens specific to the whole-cell ES vaccine as well as to the ESC-derived exosome vaccine. Our data demonstrate that antibodies against several lung cancer-associated keratin members were enriched in the serum of vaccinated mice. In summary, these data suggest that the tumor-preventing efficacy of ESC-based vaccine is reliant on the differentiation properties of these stem cells.

**Supplementary Information:**

The online version contains supplementary material available at 10.1038/s41598-024-83932-0.

## Introduction

In histologically poorly differentiated human tumors, genes that normally overexpress in embryonic stem cells (ESCs) are enriched when compared with well-differentiated tumors^1^. Furthermore, increased expression of ESC markers in various cancer types is also associated with unfavorable prognosis for patients^2^. The resemblance between ESCs and tumors has sparked numerous research efforts focusing on exploring the preventive effects of ESCs in cancer^3–6^. It is well known that ESCs and tumor cells share common antigens^7^, and this antigenic overlap between embryonic tissues and neoplastic cells, such as carcinoembryonic antigen (CEA), prostate-specific antigen (PSA), and cancer/testis antigen (CTA), has been extensively studied^8^. However, the extent to which embryonic/cancer tissue antigens overlap remains unknown. Thus, understanding this overlap remains an area of ongoing research. It has been demonstrated that embryonic materials possess immune regulatory activity to prevent cancers^4^. Exploiting the antigenic similarity between malignant cells and ESCs, we have previously reported the development of an anti-lung cancer vaccine that consists of allogeneic murine ESCs along with allogeneic murine fibroblasts expressing granulocyte–macrophage colony-stimulating factor (GM-CSF) as an immunostimulatory adjuvant^3^. Studies published recently provide support to our strategy by demonstrating that irradiated, induced pluripotent stem cells (iPSCs) function as a prophylactic vaccine against transplanted tumors by eliciting anti-tumor immune responses^9,10^. Furthermore, our recent work shows that a vaccine composed of exosomes from murine ESCs engineered to produce the immunostimulant GM-CSF is effective in preventing the outgrowth of several types of implanted tumors in mice^11,12^. Importantly, the expression patterns of a panel of tumor-associated antigens in murine ESCs and murine lung tumor cells were similar, raising the possibility that shared expression of antigens between ESCs and lung cancer cells might induce immunity against pulmonary malignancy^12^.

Accumulating evidence suggest that lung tumorigenesis is initiated and maintained by cancer stem cells (CSCs) localized in the airways^13^. Lung CSCs display characteristics of high tumorigenic activity, robust self-renewal capability, and strong resistance to chemotherapy and radiation therapy, similar to CSCs found in other tumor types^14^. The capacity for self-renewal of ESCs is one of the most specialized properties shared with CSCs present in tumors^15^. Both tumors and CSCs are capable of self-renewal and phenotypic plasticity^15^. Cancer prognosis in patients is often dictated by the differentiation status of the tumor type—patients with poorly differentiated tumors have the worst prognosis^16^. A panel of genes identified in different histologically poorly differentiated tumors have been shown to be enriched in ESCs^1^. Moreover, genes encoding specific transcriptional regulators in ESCs are often overexpressed in poorly differentiated tumors^17^. Similarly, a large portion of ESC markers have been discovered to be overexpressed in tumors from 40 different cancer types when compared with their normal tissue counterparts^2^. Furthermore, an analysis of large-scale gene data sets from ESCs and human cancers have revealed an ESC-like transcriptional program activated in various human epithelial cancers, which is strongly correlated with bad prognosis^18^. This suggests that the preventative effect of ESC-based vaccines against cancer may be reliant on their differentiation status. It appears that the mechanisms involved in the prophylactic efficacy of vaccines derived from diverse early-stage embryonic cells vary to a certain degree. Mice immunized with a vaccine consisting of murine ESCs along with GM-CSF induce robust tumor-reactive primary and memory CD8^+^ T effector responses against lung tumors^3^.

In this study, we examined how the differentiation status of ESCs influences their preventive effectiveness against lung cancer. We show that differentiation of ESCs decreases the efficacy of an ESC-based vaccine in preventing lung tumor development. Although ESC-based vaccine holds promise for inducing protection against lung cancer, the nature of (presumably multiple) tumor antigens that lead to vaccine-induced anti-lung tumor immune responses is still unknown. We hypothesize that both known and/or unknown tumor-associated antigens shared by tumors cells and ESCs are responsible for ESC-based vaccine-induced protection against lung cancer. One of the major goals of this study is to identify tumor antigens, which could potentially lead to the development of novel prophylactic agents for lung cancer prevention in humans. Here, we investigated the specificity of the antibodies generated by ESC-based vaccines using an unbiased immunoproteomics approach. Cancer-associated keratin family members were the most predominant antigens identified in our studies. As proteins involved in forming intermediate filaments in epithelial cells, keratin proteins are crucial for uncontrolled proliferation of malignant cells of epithelial origin^19^. Accordingly, since 1980, keratins have been extensively used as immunohistochemical markers in clinical tumor diagnosis^20^. Importantly, the expression patterns of keratin subtypes have been utilized to predict the origin of primary tumors as well as in the assessment of different types of cancer metastases^19^. Our data show the enrichment of serum antibodies against several keratin proteins associated with lung tumorigenesis in immunized mice.

## Results

### The expression of pluripotency and differentiation markers in murine ESCs is altered during differentiation

**Fig. 1:**
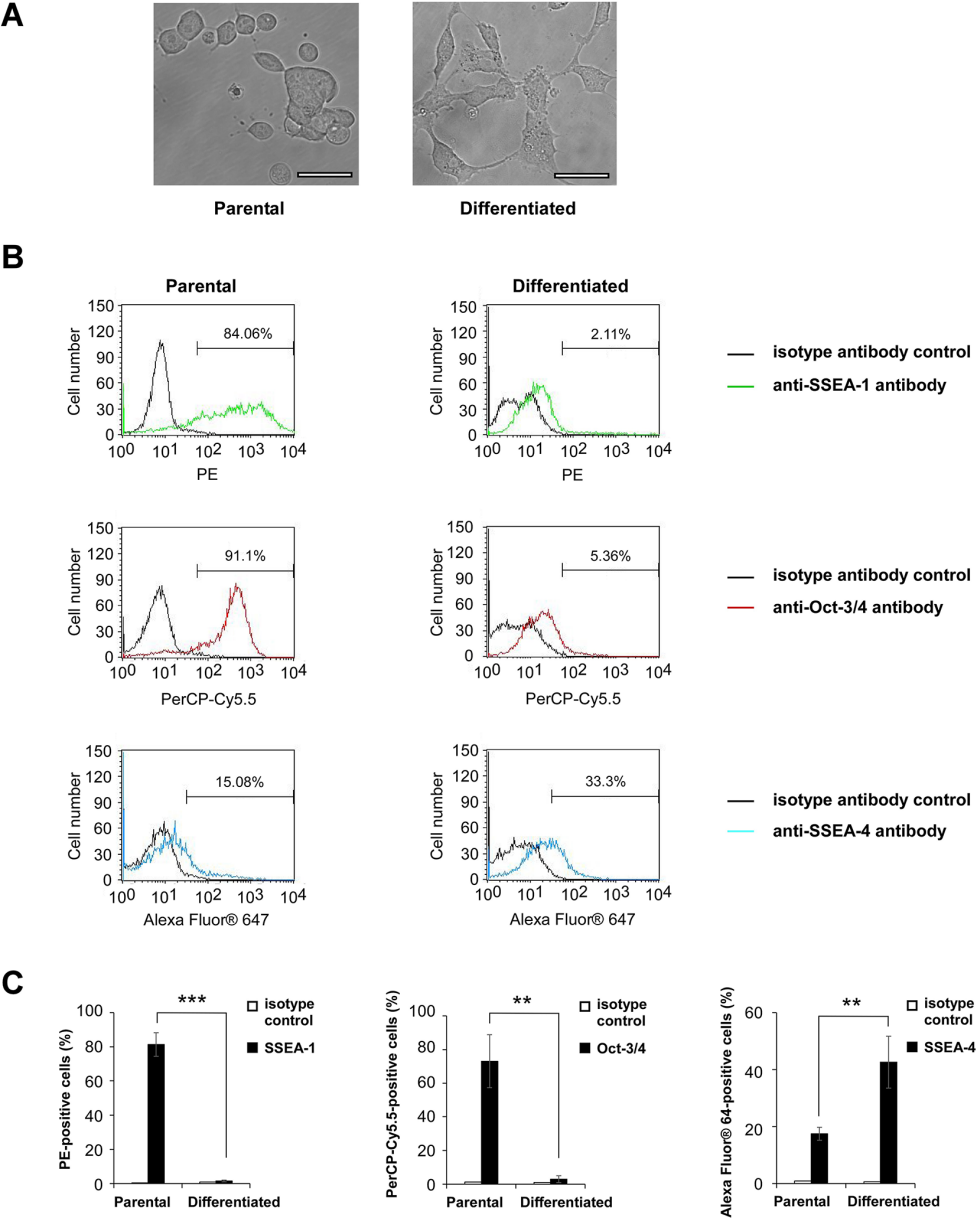
Characterization of differentiated ES-D3 cells. Murine embryonic stem cell line ES-D3 cells were cultured in the absence of LIF and β-mercaptoethanol for 4 days. Then ES-D3 cells were cultured in the presence of 0.5 μM retinoic acid for 4 days. (**A**) Representative images of parental and differentiated ES-D3 cells. Scale bar, 50 µM. (**B**) Flow cytometry analysis of the expression of pluripotency and differentiation markers (SSEA-1, Oct-3/4 and SSEA-4) in parental and differentiated ES-D3 cells. Numbers in the histogram plots represent the percentages of subpopulations positive for antibody of a particular pluripotency and differentiation marker. (**C**) Bar graphs showing the percentages of subpopulations positive for a particular fluorophore as presented in (**B**). The data are shown as means ± standard deviations of n = 3 independent experiments in each group. Student’s unpaired t test. Asterisks (**), indicate p < 0.01; Asterisks (***), indicate p < 0.001.

ESCs and lung cancer cells express common protein markers and share several genotypic and phenotypic traits^8,17^. Among them, the capacity for self-renewal is one of the most specialized properties shared between ESCs and CSCs. We reasoned that the anti-lung tumor immunity generated by ESC-based vaccines, either as intact cells or as secreted exosomes, is likely attributed to shared carcinoembryonic antigens whose expression is reduced or eliminated during stem cell differentiation. Here, a previously published approach was employed to differentiate the murine ESC line ES-D3 by deprivation of extrinsic self-renewal signals from leukemia inhibitory factor (LIF) coupled with the addition of retinoic acid^21,22^. Morphological characterization of parental and differentiated ES-D3 cells was carried out using microscopy (Fig. 1A). Undifferentiated parental ES-D3 cells had morphology of typical ESCs with rounded cell shape and smooth cytoplasmic membrane, and these cells generated close cytoplasmic membrane contact with each other to form colonies^23^. In contrast, differentiated ES-D3 cells displayed morphology with protrusions, which is consistent with the morphological characteristics of differentiated cells^24^.

A number of molecular markers have been reported to be indicative of the pluripotency of murine ESCs, among which SSEA-1, SSEA-4, and Oct-3/4 expression are commonly studied to examine the differentiation status of stem cells^25^. Generally, pluripotent murine ESCs exhibit elevated SSEA-1 and Oct-3/4 reactivity, but low expression of SSEA-4^26^. Therefore, we evaluated the differentiation status of differentiated ES-D3 cells by measuring SSEA-1, SSEA-4 and Oct-3/4 expression levels via flow cytometry. Loss of undifferentiated state of differentiated ES-D3 cells was indicated by decreased SSEA-1 and Oct-3/4 expression and increased SSEA-4 expression compared with parental ES-D3 cells (Fig. 1B and C, Supplementary figure 1). Overall, differentiated ES-D3 cells are distinct from their undifferentiated counterparts with both morphological and protein marker expression changes.

### The differentiation status of ESCs is important for their anchorage-independent growth potential

**Fig. 2:**
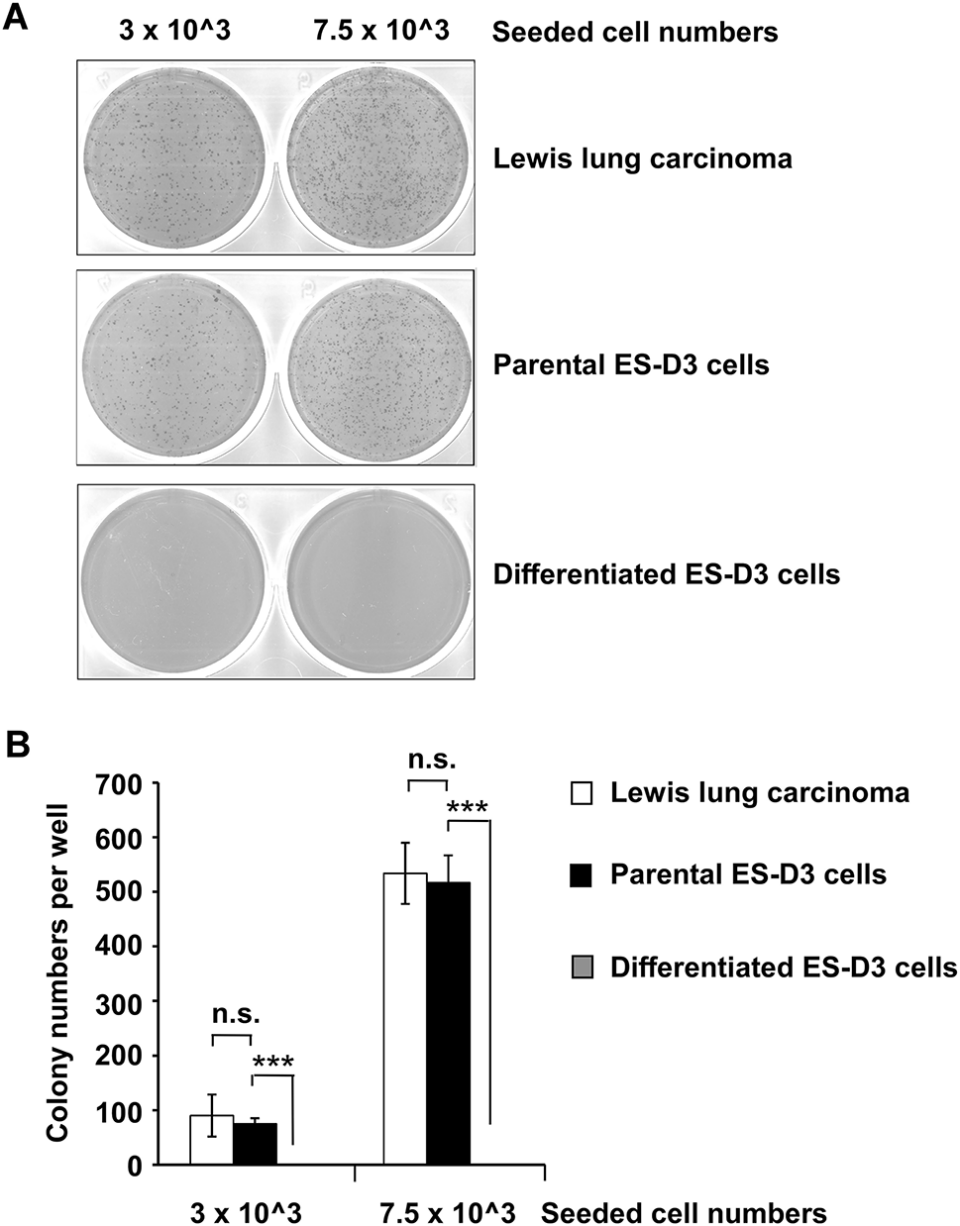
Differentiation of ES-D3 cells inhibits their anchorage-independent proliferation. The anchorage-independent growth capability of differentiated ES-D3 cells and their parental counterparts was examined by a soft agar colony formation assay. (**A**) The indicated number of LLC, parental ES-D3 cells and differentiated ES-D3 cells were cultured in soft agar for 8 days. Representative images of the plates are shown. (**B**) The colonies of LLC, parental ES-D3 and differentiated ES-D3 on the soft agar plates shown in (A) were counted. The data are presented as means ± standard deviations of n = 3 independent experiments. Student’s unpaired t test. Asterisks (***), indicate p < 0.001. “ns”, no significance.

A hallmark of tumorigenesis is the proliferation of neoplastic cells in an anchorage-independent manner, which correlates with tumorigenic and metastatic potential in vivo^27^. The soft agar colony formation assay is a well-accepted method to evaluate anchorage-independent growth of tumor cells^28^. Due to anoikis, a particular type of apoptosis, almost no colonies are formed when non-transformed cells are plated in an anchorage-independent culture model^29^. Although ESCs and malignant cells share some genotypic and phenotypic traits, it was unclear whether ESCs possess anchorage-independent growth potential, as tumor cells do. To determine this, equal numbers of parental ES-D3 cells and Lewis lung carcinoma (LLC), an aggressive non-small cell lung cancer cell line that proliferate very quickly (within days) when implanted into immunocompetent mice^30^, were seeded in soft agar. As shown in Fig. 2, LLC cells and parental ES-D3 cells displayed similar anchorage-independent growth capacity. To our knowledge, this is the first study demonstrating that the anchorage-independent growth potential of undifferentiated ESCs is similar to that of lung tumor cells. To further explore if anchorage-independent growth of ESCs is dependent on their differentiation status, we compared the colony forming abilities of undifferentiated parental and differentiated ESC cells. The ability of differentiated ES-D3 cells to form colonies in soft agar was reduced to extremely low levels compared with those of undifferentiated ES-D3 and LLC cells. These results indicate that differentiation status of ESCs is critical for their ability to evade anoikis and grow in an anchorage-independent manner.

### Differentiation of ESCs decreases their efficacy in preventing lung tumor development

**Fig. 3:**
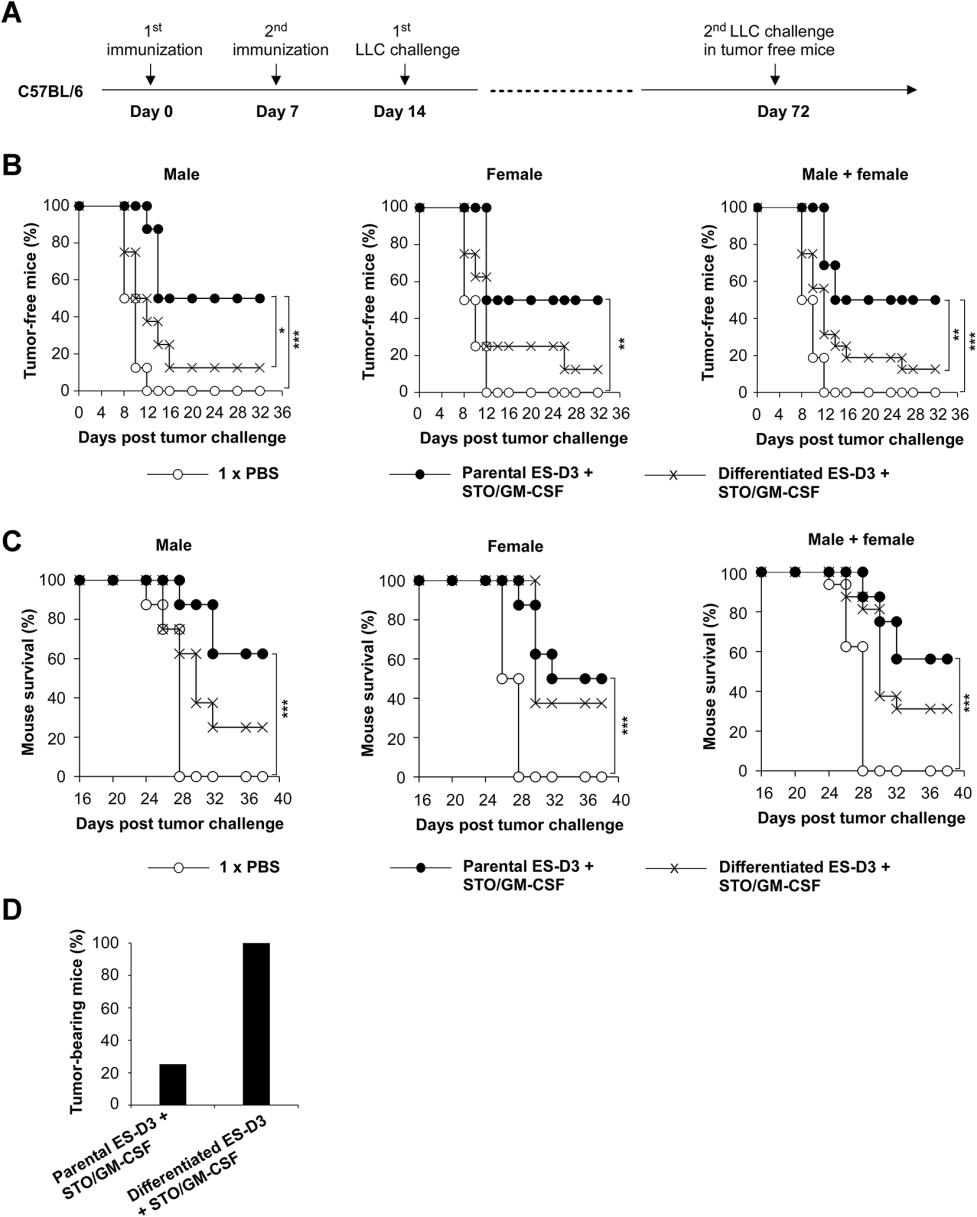
Differentiation of ES-D3 cells decreases their efficacy to prevent the development of implanted lung tumors. (**A**) The schematic depiction of immunization regimen. Male and female C57BL/6 mice were immunized twice with 1 × PBS (vehicle control), parental ES-D3 cells + STO fibroblasts expressing GM-CSF (STO/GM-CSF) or differentiated ES-D3 cells + STO/GM-CSF prior to subcutaneous challenge with LLC cells (0.15 × 10^6^) at Day 14. Tumor-free mice were challenged again with LLC cells (0.15 × 10^6^) at day 72. (**B**) The percentages of tumor-free male mice (n = 8), female mice (n = 8), or male + female mice (n = 16, 8 mice/gender) following the first LLC injection are shown. Log-rank test; Asterisk (*) indicates p < 0.05; Asterisks (**) indicate p < 0.01; Asterisks (***) indicate p < 0.001. (**C**) Kaplan–Meier survival curve demonstrates the probability of survival in male mice (n = 8), female mice (n = 8), or male + female mice (n = 16, 8 mice/gender). Log-rank test; Asterisks (***) indicate p < 0.001. (**D**) Twenty-eight days after the second LLC challenge, the percentages of tumor-bearing mice in mice immunized with parental ES-D3 cells + STO/GM-CSF (n = 8) or differentiated ES-D3 cells + STO/GM-CSF (n = 2) were determined.

To evaluate if differentiation status of ES-D3 cells influences their anti-tumor efficacy, we injected mice with ES-D3-based vaccine according to a previously established protocol in an earlier study^3^. For this purpose, three groups of C57BL/6 mice were immunized (8-week-old, 8 per sex per group): vehicle control (PBS), parental ES-D3 cells along with STO fibroblasts expressing GM-CSF (STO/GM-CSF) or differentiated ES-D3 cells along with STO/GM-CSF. Mice were immunized twice (day 0 and day 7) followed by a challenge with subcutaneous injection of LLC cells at day 14 (Fig. 3A). The rates of tumor development were similar between male and female mice (Fig. 3B). All control mice developed tumors starting 12 days post-LLC cell administration. In contrast, 50% of mice vaccinated with undifferentiated ES-D3 cells were protected from tumor outgrowth even at day 32 post-challenge. Importantly, vaccination with differentiated ES-D3 cells exhibited lower efficacy against lung cancer by 37.5% when compared to undifferentiated ES-D3 cells (Fig. 3B). Furthermore, differentiation of ES-D3 cells significantly decreased their effectiveness to inhibit LLC tumor progression in female mice (Supplementary figure 2). Kaplan–Meier survival curves showed that all non-vaccinated control animals died within 28 days of tumor cell challenge, whereas 56% of mice vaccinated with undifferentiated ES-D3 cells survived beyond 38 days (Fig. 3C). Importantly, only 31% of animals immunized with differentiated ES-D3 survived for the 38-day period (Fig. 3C), indicating that differentiation decreases the cancer prevention efficacy of ESC-based vaccine. Additionally, the results obtained from male mice are very comparable with those from female mice (Fig. 3C).

To evaluate if the differentiation status of ES-D3 cells influences their ability to generate long-term immunity against lung cancer, we tested the anti-tumor responses using the mice that had been previously vaccinated and challenged with LLC cells. The previously LLC-challenged tumor-free mice were re-injected with LLC cells 58 days after the first LLC injection (Fig. 3A). Notably, 2 out of 2 tumor-free mice (100%) vaccinated with differentiated ES-D3 cells developed tumors 8 days after the second LLC challenge, whereas only 25% of mice (2 out of 8) in the parental undifferentiated ES-D3 + STO/GM-CSF vaccination group developed tumors following LLC cell re-inoculation (Fig. 3D). This provides evidence that the differentiation status of ESCs is essential for their long-term anti-tumor protection activity.

### ES-D3 cells and lung tumor cells share a common antigenic signature

Earlier studies have demonstrated that vaccination with exosomes of ES-D3 cells expressing GM-CSF prevents lung tumorigenesis in several tumor models, including implanted LLC tumors^11,12^. However, the antigens responsible for prophylactic efficacy of pluripotent ES-D3 cells or exosomes isolated from ES-D3 cells have not been identified yet. To gain a comprehensive understanding of the antigenicity of ESC

vaccine, an immunoproteomic strategy was employed (Supplementary figure 3). This approach has the potential to reveal the complete repertoire of potentially immunogenic antigens in the serum of immunized mice. This combined affinity chromatography shotgun immunoproteomic approach starts with capture of antibodies in the serum by an IgG affinity column^31^. Specifically, mice (C57BL/6) were immunized with either a cell-based vaccine (parental ES-D3 cells + STO/GM-CSF cells) or an exosome-based vaccine (exosomes from GM-CSF-expressing ES-D3 cells) as described earlier^11,12,32^. After the binding of serum antibodies to protein A beads, antigen-containing ESC lysates or exosomal lysates were loaded to the respective mixture of serum antibodies bound protein A beads. Antigens bound to the beads were identified by liquid chromatography tandem mass spectrometry (LC–MS/MS) analysis.

We identified a total of 161 immunoglobin-unrelated proteins whose antibody levels were higher in the serum from mice immunized with the cell-based vaccine than their counterparts from unvaccinated control mice (Supplementary figure 4A and Supplementary table 2). Among them, 20 identified proteins are members of the keratin family, some of which have been shown to be tumor-associated antigens^33–35^ (Table 1). Indeed, several members of keratin proteins are associated with lung malignancy^19,20^. In our study, the levels of antibodies specific for lung-cancer-associated keratins 7, 8, 16, 17, and 19 were higher in the serum of mice vaccinated with intact pluripotent ES-D3 cells (Table 1).

**Table 1:** Relative abundance of keratin members recognized by antibodies in the serum of mice vaccinated with ES-D3 cells.

Keratin member	Relative abundance (control)	Relative abundance (immunized by ES-D3 + STO-GMCSF)	Ratio of abundance (control/immunized)
Keratin 1	65	90	0.72
Keratin 2	65	113.3	0.57
Keratin 4	0	31.5	0
Keratin 5	81.1	152.4	0.53
Keratin 6A	32.5	66.6	0.49
Keratin 7*	0	7.6	0
Keratin 8*	44.7	66.4	0.67
Keratin 10	101.8	191	0.53
Keratin 15	48.6	90.3	0.54
Keratin 16*	40.9	70.2	0.58
Keratin 17*	36.4	43.1	0.85
Keratin 19*	12	31.3	0.38
Keratin 42	28.3	31.2	0.91
Keratin 72	0	39.3	0
Keratin 73	20.5	70.4	0.29
Keratin 76	16.1	47	0.34
Keratin 77	69.1	148.6	0.46
Keratin 78	12	35	0.34
Keratin 79	28.9	70.4	0.41
Keratin 80	0	4	0

The abundance of keratin proteins recognized by antibodies in the serum of control mice and the mice immunized with ES-D3 cells is presented. Protein abundance was normalized against immunoglobulin G-binding protein A using the following formula: relative protein abundance = abundance value of an antigen / abundance value of immunoglobulin G-binding protein A × 10,000. Data presented are the average values of two independent experiments. Keratin members associated with lung tumorigenesis are marked with an asterisk (*).

The preventive efficacy of exosomes from GM-CSF-expressing ES-D3 cells has been demonstrated using mouse implanted tumor models^11,12^. Like the vaccine based on ES-D3 cells, the exosome-based vaccine generated more antibodies that recognize 105 non-immunoglobin proteins than those from vehicle control mice (Supplementary figure 4B and Supplementary table 3). Consistent with the results acquired with the whole cell-based vaccine, a large portion of identified antigens (19 out 105) were keratin members. Importantly, antibodies against lung tumor-associated keratin members 8, 14, 16, and 17 were also enriched in the serum from exosome-immunized mice (Table 2, Supplementary table 4 and Supplementary figure 5A).

**Table 2:** Comparison of relative abundance of keratin proteins recognized by serum antibodies of control mice or mice vaccinated with ES-D3 exosomes.

Keratin member	Relative abundance (control )	Relative abundance (immunized by ES-D3 exosomes)	Ratio of abundance (control/immunized)
Keratin 2	23.1	42.8	0.54
Keratin 5	26.4	42.8	0.62
Keratin 6A	0	52.6	0
Keratin 6B	13.2	42.8	0.31
Keratin 8*	13.2	19.7	0.67
Keratin 13	13.2	26.3	0.50
Keratin 14*	0	42.8	0
Keratin 15	19.8	36.2	0.55
Keratin 16*	16.5	42.8	0.39
Keratin 17*	13.2	29.6	0.45
Keratin 20	3.3	6.6	0.50
Keratin 42	6.6	39.5	0.17
Keratin 71	16.5	19.7	0.84
Keratin 72	6.6	13.2	0.50
Keratin 73	0	23	0
Keratin 75	0	29.6	0
Keratin 77	23.1	32.9	0.70
Keratin 78	9.9	13.2	0.75
Keratin 79	0	29.6	0
Keratin 76	3.3	3.3	1
Keratin 1	29.7	0	
Keratin 27	13.2	0	

The abundance of keratin members recognized by antibodies in the serum of control mice and the mice vaccinated with exosomes of ES-D3 cells expressing GM-CSF was normalized against immunoglobulin G-binding protein A. The relative abundance of antigens is calculated using the following formula: relative protein abundance = abundance value of an antigen / abundance value of immunoglobulin G-binding protein A × 10,000. Data presented are the values from one experiment. Lung cancer-associated keratin members are marked with an asterisk (*).

To further evaluate the potential involvement of lung cancer-associated keratin proteins in anti-lung tumor immunity evoked by ESC vaccines, the protein expression profile of LLC cells was examined by a proteomics approach (Supplementary table 5). In contrast to identifying the antigenic profile of mouse serum by enzymatically digesting antigens while bound to Protein A agarose beads (Supplementary figure 3), the experimental approach for proteomics analysis of LLC cell lysate was different in such a way that proteins in the lysate of LLC cells were directly digested. As expected, several keratins linked to lung tumorigenesis (keratins 7, 8, 16, 17 and 18) were found to be expressed in LLC cells (Table 3, Supplementary figures 5B and 5C).

**Table 3:** Lung cancer-associated Keratin members are identified in the serum of vaccinated mice and Lewis lung carcinoma cells.

Keratin member	Cell-based vaccine	Exosome-based vaccine	Lewis lung carcinoma
Keratin 7	+		+
Keratin 8*	+	+	+
Keratin 14		+	
Keratin 16*	+	+	+
Keratin 17*	+	+	+
Keratin 18			+
Keratin 19	+		

The list of lung cancer-associated keratin members identified in the serum of the mice immunized with intact ES-D3 cells or ES-D3 exosomes and Lewis lung carcinoma cells. Lung cancer-associated keratin members present in both serum of vaccinated mice and LLC cells are marked with an asterisk (*).

## Discussion

The generation of different types of tumors is a complex process in which tumors acquire the ability of dynamic regulation and constituent cellular populations, recapitulating the complexity of organs or tissues^36^. Embryonic materials can be used as a vaccination strategy against cancers based on the similarities between embryos and tumors^8^. It has been found that some genes expressed in histologically poorly differentiated tumors cells are overexpressed in ESCs, and vice versa^1^. The identification of CSCs indicates that most tumors harbor stem cells that are capable of regenerating tumors^15^. In our study, parental ES-D3 cells display robust anchorage-independent growth capability, a key signature that correlates with tumorigenic and metastatic potential of tumors in animals^27^. This suggests that the molecular mechanisms governing cell proliferation in ESCs is similar to that in neoplastic cells. Importantly, a decrease in the undifferentiated state of ESCs completely abolished their anchorage-independent growth potential (Fig. 2), lending more support for the importance of ESC differentiation status in regulating the cellular phenotypes that are similar to those present in tumor cells.

Anti-tumor immune responses induced by ESC vaccine are critical for the prevention of tumorigenesis. The similarity between ESC antigens and tumor antigens is considered to be responsible for ESC vaccine-induced immune responses against malignancy^3–6^. In this study, vaccination with parental pluripotent ESCs inhibited lung tumor development, whereas the differentiation of ESCs suppressed the efficacy of the vaccine. As the protection against lung cancer conferred by ESC vaccination likely involves a number of shared antigens, it is conceivable that differentiation could decrease expression of shared antigens in ESCs, leading to a reduction in cross-immunity against tumors. This is in agreement with the differentiation-evoked loss of tumor cell signatures in ESCs, such as anchorage-independent growth.

Our earlier studies indicate the anti-tumor efficacies of both ESC cell-based or exosome-based vaccines were correlated with significantly enhanced T cell-mediated immune responses, including potent Th1-mediated cytokine responses in splenic CD8^+^ T cells and higher CD8^+^ T/T_regs_ cell ratio in tumors^3,11^. However, whether the humoral immune response mediated by antibodies also contributes to the anti-cancer efficacy of ESC vaccination strategy remains unknown. Interestingly, a similar iPSC-based vaccine induced antibodies that showed reactivity against tumors cells^9^. Immunization with antibodies specific for tumor‐associated antigens has been reported to trigger an immunological response against tumor cells through antibody-dependent cellular cytotoxicity (ADCC), a cell-mediated immune defense mechanism^37^.Thus, it is possible that antibodies generated by ESC-based vaccine recognize the antigens expressed on lung tumor cells to evoke ADCC-mediated anti-tumor immunity.

A large portion of antigens identified by immunoproteomic analysis are keratin family members. Notably, in our study, keratins 8, 16, and 17 are among the lung cancer-associated keratin family members whose serum antibody levels were enhanced by both the whole cell ESC vaccine as well as the ESC exosome-based vaccine. Importantly, these keratin proteins are also present in LLC cell lysate. Based on clinical research, the expression of keratin 8 is significantly higher in patients with non-small cell lung cancer (NSCLC) compared with small cell lung cancer (SCLC) patients, and high expression of keratin 8 enhances tumor progression and indicates poor prognosis^38^. In human lung adenocarcinoma, keratin 8 has been found to promote metastasis and epithelial-mesenchymal transition (EMT) through NF-κB signaling^39^. Furthermore, a recent study has demonstrated that lung adenocarcinoma may develop through aberrant persistence of a keratin 8 + transitional stem cell state that can generate a distinct cell–cell communication network and dysregulate molecular checkpoints on differentiation^40,41^. As a member of the type I cytokeratin, keratin 16 has been found to associate with squamous differentiation^42^. Recent studies demonstrate that keratin 16 possesses oncogenic activity to promote the tumorigenesis of lung adenocarcinoma and is a predictive factor of poor patients’ prognosis^43,44^. Importantly, keratin 16 overexpression promotes lung adenocarcinoma metastasis by stabilizing the structural protein vimentin, which is essential for metastasis driven by keratin 16^44^. Accumulating studies indicate that keratin 17 is overexpressed in several malignancies and plays an important role in the progression of tumors^45–48^. Markedly, metastasis and poor survival of lung cancer patients are correlated with high expression of keratin 17^48,49^.

Although keratin proteins form intermediate filaments inside cells, emerging evidence indicates that some keratin family members are localized on the cell surface. For instance, the presence of keratins 8, 18 and 19 are detected on the outer surface of human mammary carcinoma cells as well as in culture medium^50^. Similarly, keratin 8 is expressed at the surface of lung carcinoma cells, but not in normal epithelial cells^51^. Furthermore, fragments of keratin 8 released by lung cancer cells were utilized as an indicator of tumor progression in clinical studies^52,53^. Importantly, the interaction between keratin 8 and MHC class I was involved in CD8^+^ T cell activation in a lymph node metastatic carcinoma cell line^54^. It is conceivable that keratin 8 molecules on lung tumor cell surface are recognized by specific antibodies generated by ESC-based vaccine, resulting in recruitment of effector immune cells and subsequent tumor cell apoptosis. Unlike keratin 8, whether keratins 16 and 17 are expressed on the surface of lung tumor cells is currently unknown. More studies are needed to address how keratin members are involved in ESC vaccine-evoked immunity.

It is notable that the implanted lung tumor model examined in this study has some limitations, as subcutaneous injection does not represent an appropriate site for lung tumors. Our future research will concentrate on mouse models that are clinically relevant, such as experimental metastasis models, orthotopic models, and primary tumor models^30^.

Overall, our studies reveal an important role of undifferentiated state of ESCs in anti-lung cancer efficacy of ESC-based vaccines. Furthermore, several keratin family members were identified with their serum antibody levels increased by both the whole cell ESC vaccine and the ESC exosome-based vaccine. Future research is warranted to elucidate if targeting tumor-associated keratins can be an effective preventive strategy against lung cancer.

## Methods

### Cell culture

Murine embryonic stem cell line ES-D3 (CRL-11632) and murine Lewis lung carcinoma (CRL-1642) were acquired from ATCC (Manassas, VA). Murine fibroblast cell line STO (ATCC # CRL-1503) infected with a replication-defective retrovirus expressing murine GM-CSF cDNA was acquired from Dr. Glenn Dranoff (Novartis Institutes for Biomedical Research; Boston, MA). ES-D3 cells were cultured in KnockOut™ Dulbecco’s Modified Eagle’s Medium (10–829-018; Thermo Fisher; Waltham, MA) supplemented with 15% exosome-free fetal bovine serum (FBS), 50 U/ml penicillin (sc45000-652; Mediatech; Manassas, VA), 50 μg/ml streptomycin (sc45000-652; Mediatech), 0.1 mM non-essential amino acids (SH3023801; Thermo Fisher), 2 mM L-glutamine (L0131-0100; VWR; Radnor, PA), 0.1 mM β-mercaptoethanol (21,985,023; Thermo Fisher) and 100 units/ml leukemia inhibitory factor (LIF; ESG1106; Thermo Fisher). Exosome-free FBS was acquired as described before^12,32^. ES-D3 cells were cultured in 15-cm tissue culture dishes pre-coated with 0.1% gelatin (ES006B; Thermo Fisher). Lewis lung carcinoma (LLC) and STO cells were grown and maintained in Dulbecco’s modified eagle’s medium (SH30243; VWR) supplemented with 10% FBS (900–108; Gemini; Broderick, CA), 100 units/ml penicillin (Mediatech) and 100 µg/ml streptomycin (Mediatech). GM-CSF produced by STO cells was evaluated by measuring GM-CSF concentration in the culture medium with a murine GM-CSF ELISA kit (88733422; Thermo Fisher) following manufacturer’s protocol. All of the cells were cultured in a 5% CO_2_ humidified incubator at 37 °C.

### Mice

Male and female C57BL/6 mice (8 weeks of age) were acquired from Jackson Laboratory (Bar Harbor, ME). Mice were maintained at the University of Louisville pathogen-free Research Resources Facilities (RRF).

### Ethics declarations

Mice were handled in accordance with the American Association for the Accreditation of Laboratory Animal Care (AAALC) guidelines and the "Guide for the Care and Use of Laboratory Animals" (Institute of Laboratory Animal Resources, National Research Council, National Academy Press, 1996). The mouse study was approved by the Institutional Animal Care and Use Committee (IACUC) of the University of Louisville (protocol number: 18301). The authors complied with the ARRIVE guidelines.

### Differentiation of murine ESCs

Murine ESCs were differentiated following a published protocol^21^. Briefly, ES-D3 cells were seeded onto a standard 10-cm petri dish in standard ESC medium lacking LIF and β-mercaptoethanol. Forty-eight hours later, the medium was replaced with fresh medium, and the cells were cultured for additional 48 h. Next, medium was replaced with fresh medium containing 0.5 μM retinoic acid (R2625; Sigma-Aldrich; St. Louis, MO) and the cells were cultured for 48 h. Finally, the cells were cultured in fresh retinoic acid-containing medium for another 48 h. The morphology of differentiated cells was examined using an EVOS cell imaging system (Thermo Fisher).

### Examining differentiation status

The differentiation status of parental and differentiated ES-D3 cells was examined using a Stemflow™ Human and Mouse Pluripotent Stem Cell Analysis Kit (BDB560477; Beckon Dickinson; Franklin Lakes, NJ) according to manufacturer’s protocol. The expression of different pluripotency and differentiation markers SSEA-1, Oct-3/4 and SSEA-4 was evaluated using flow cytometry (FACScalibur; Beckon Dickinson). The information of antibodies is presented in Supplementary table 1.

### Anchorage-independent cell culturing

After heating in a 42 °C water bath for 30 min, 3.5% soft agar solution was diluted to 0.7% with fresh ES-D3 cell culture medium. To form the agar base, 3 ml of 0.7% agar solution was plated in each well of a 6-well tissue culture plate. The agar in the plates was then solidified at 4 °C for 30 min. Parental ES-D3 cells, differentiated ES-D3 cells, and LLC cells (3 × 10^3^ or 7.5 × 10^3^) were transferred on top of agar base in each well in a final volume of 3 ml 0.7% agar solution. Each cell line and seeding density was plated in duplicate. The plates were placed at 4 °C for 5 min before being transferred to a 5% CO_2_ humidified incubator at 37 °C for 8 day-culture. The plates were then stained with 1 mg/ml iodonitrotetrazolium chloride (I10406; Sigma-Aldrich) overnight. The images were acquired using an EVOS cell imaging system (Thermo Fisher) and the numbers of cell colonies were determined using “Analyze Particles" function of the software ImageJ (National Institutes of Health; Bethesda, MD), which allows automatically counting and measuring individual colonies within a binary or thresholded image.

### Vaccination against tumor challenge

Parental or differentiated ES-D3 cells and STO cells overexpressing GM-CSF were disassociated from tissue culture plates with 0.05% trypsin/EDTA (25–052-CI; Corning; Corning, NY). Collected cells were washed twice with sterile 1 × PBS and suspended in 1 × PBS at a concentration of 10 × 10^6^/ml. Parental or differentiated ES-D3 cells (1 × 10^6^) along with GM-CSF-expressing STO cells (1 × 10^6^) were injected subcutaneously (s.c.) in the right flank of 8-week-old male or female C57BL/6 mice. Primary vaccination was carried out on day 0, and a booster dose was injected on day 7. Seven days after the second vaccination dose, mice were subcutaneously administered with LLC cells (0.15 × 10^6^) on the left flank. Tumor growth in mice was monitored daily. Tumor growth was measured using dull-edged Vernier calipers every day, and tumor volumes were calculated using the following formula: V = L*W^2^/2, where V is volume, L is length, and W is width. Mice were euthanized when tumors exceeded 5% of body weight via CO_2_ asphyxiation. On day 72, tumor-free mice were challenged with LLC cells (0.15 × 10^6^) for a second time by subcutaneous injection on the left flank. Tumor growth was monitored daily. At endpoint, mice were euthanized via CO_2_ asphyxiation.

### Proteomics analysis of LLC whole cell extract

LLC cells (10 × 10^6^) were extracted with 500 µl lysis buffer containing 50 mM Tris (pH7.4), 150 mM NaCl, 1% NP-40, and 0.05% SDS and stored at 4 °C. The lysate was analyzed at the Genome Center, University of California at Davis. Following the standard procedure of protein digestion by Trypsin/Lys-C (V5071; Promega; Madison, WI), samples were desalted by C18 Microspin columns (SEMSS18V; Nest Group; Southborough, MA) and lyophilized by vacuum centrifugation. Liquid chromatography (LC) separation was carried out with an Easy-nLC 1000 LC system (Thermo Fisher). Digested peptides were reconstituted in 2% acetonitrile/0.1% trifluoroacetic acid. The samples (3 µg) were loaded onto a 100 micron × 25 mm Magic C18 100 Å 5U reverse phase trap where they were desalted online before being separated on a 75 micron × 150 mm Magic C18 200 Å 3U reverse phase column. Peptides were eluted with a gradient of 0.1% formic acid (A) and 100% acetonitrile (B), which was run with 5% to 35% B (45 min), 35% to 80% B (8 min), 80% B (1 min), 80% to 5% B (1 min), and 5% B (10 min). The collection of mass spectra (MS) was carried out with a mass spectrometer (Orbitrap Q Exactive Plus; Thermo Fisher) in a data-dependent mode with MS precursor scan followed by 15 MS/MS scans. A dynamic exclusion of 15 s was used. All MS/MS samples were analyzed using X! Tandem (CYCLONE; 2013.02.01.1) to search the uniprot mouse proteome plus an equal number of reverse decoy sequences (142,010 entries). MS/MS-based peptide and protein identifications were validated with the software Scaffold (Scaffold_4.8.2; Proteome Software; Portland, OR). Peptide identifications were accepted if they could be established at greater than 98% probability by the Scaffold Local FDR algorithm. Protein identifications were accepted if they could be established at greater than 5% probability to achieve an FDR less than 5% and contained at least one identified peptide.

### Antigen capture experiments in mice immunized with intact ESCs

Vehicle only (1 × PBS) or ES-D3 cells (1 × 10^6^) combined with STO-fibroblasts expressing GM-CSF (1 × 10^6^) were subcutaneously injected at right flank into eight-week-old male C57BL/6 mice (2 mice/group) twice with one week apart. One week following the second immunization, whole blood was collected by cardiac puncture immediately after CO_2_ asphyxiation. Whole blood was transferred to a serum separator tube (365,967; Beckon Dickinson) and serum was collected. Protein A agarose beads (200 µl; 9863S; Cell Signaling; Danvers, MA) were resuspended with 1 ml binding buffer containing 50 mM Tris (pH7.5) and 150 mM NaCl and centrifuged at 390 × g for 30 s. Binding buffer was removed, and wash was repeated twice. The mouse serum (200 µl) was added to the tubes containing the beads. The serum-bead mixture was incubated on a rotator for 3 h at room temperature. ES-D3 cells were collected (30 × 10^6^ cells/each group) by trypsinization. Cell pellets were resuspended in 5 ml 1 × lysis buffer containing 50 mM Tris (pH7.5), 150 mM NaCl, 1% NP-40, 0.05% SDS, protease inhibitor (P8349; Sigma-Aldrich) and phosphatase inhibitor (04 906 845 001; Roche Diagnostics; Indianapolis, IN) and sonicated at 20% amplitude using a sonicator (Sonic Dismembrator Model 500, Thermo Fisher) twice for 5 s each time with at least one minute-rest on ice between each 5-s pulse. Then the cell lysate was pushed through an insulin syringe 10 times and centrifuged at 13,000 × g for 10 min at 4 °C. The supernatant was taken out as whole cell extract for the antigen binding experiment. The serum-bead mixtures were centrifuged at 390 × g for 30 s at 4 °C. The beads were washed for 3 times with 500 μl of 1 × lysis buffer by rotating for 10 min at 4 °C. The whole cell extract was added to each tube containing the beads, and the tubes were rotated at 4 °C overnight. Then the extract-bead mixtures were centrifuged at 390 × g for 30 s at 4 °C. The beads were washed 5 times with 1 ml of binding buffer by rotating for 10 min at 4 °C. Abundance of proteins bound to the beads was evaluated by proteomic analysis at the Genome Center, University of California at Davis.

### Antigen capture experiments in mice immunized with exosomes of ESCs

Exosomes from ES-D3 cells stably expressing GM-CSF were isolated as described earlier^32^. Male C57BL/6 mice (8 weeks of age, 2 mice/group) were immunized twice (day 0 and day 7) with vehicle only (1 × PBS) or 225 µg of ES-D3 exosomes by subcutaneous injection. Seven days following the second vaccination, serum was collected as described above with the serum acquired from mice immunized by intact ES-D3 cells. The serum collected from 2 mice was combined (200 µl, 100 µl from each mouse) and added to the tubes containing the beads. The mixture was rotated at room temperature for 3 h. Exosomes (9 mg) were resuspended in 1.2 ml 2 × lysis buffer containing 100 mM Tris (pH7.5), 300 mM NaCl, 2% NP-40, 0.1% SDS, protease inhibitor (P8349; Sigma-Aldrich) and phosphatase inhibitor (04 906 845 001; Roche Diagnostics; Indianapolis, IN). The lysate of exosomes was acquired in the same fashion as for the whole cell extract of ES-D3 cells described above. The exosomal lysate went through an insulin syringe 10 times. Following centrifugation at 13,000 × g for 10 min at 4 °C, the exosomal extract was acquired. The Protein A agarose beads incubated with serum were collected by 390 × g centrifugation at 4 °C for 30 s. Following wash with 500 μl of 1 × lysis buffer 3 times, the beads were incubated with exosomal extract (in 1 ml 1 × lysis buffer) at 4 °C overnight. The extract-bead mixtures were collected by centrifugation at 390 × g for 30 s at 4 °C. The beads were washed 5 times with 1 ml of binding buffer by rotating for 10 min at 4 °C. Samples were then examined by proteomics analysis.

### Proteomics analysis of protein samples bound to Protein A agarose beads

Protein samples bound to protein A agarose beads were washed four times with 200 μl of 50 mM ammonium bicarbonate (AMBIC; A6141; Sigma-Aldrich) with a 20-min shake at 4 °C between each wash. About 2.5 µg of Trypsin/Lys-C (V5071; Promega) was added to the beads and AMBIC mixture and the samples were digested overnight at 800 × rpm shake speed. Following digestion, the supernatant was transferred to a microfuge tube and the beads were washed once with 50 mM AMBIC. After a 20-min gentle shake, the wash was transferred and combined with the initial supernatant. The peptide extracts were reduced in volume by vacuum centrifugation and a small portion of the extract was used for fluorometric peptide quantification (23,290; Thermo Fisher). One microgram of sample based on the fluorometric peptide assay was loaded for each LC–MS analysis.

Digested peptides were analyzed by LC–MS/MS on a Thermo Scientific Q Exactive Orbitrap Mass spectrometer in conjunction Proxeon Easy-nLC II HPLC (Thermo Fisher) and Proxeon nanospray source. The digested peptides were loaded into a 100 micron × 25 mm Magic C18 100 Å 5U reverse phase trap where they were desalted online before being separated using a 75 micron × 150 mm Magic C18 200 Å 3U reverse phase column. Peptides were eluted using a 60-min gradient with a flow rate of 300 nl/min. An MS survey scan was obtained for the m/z range 300–1600, MS/MS spectra were acquired using a top 15 method, where the top 15 ions in the MS spectra were subjected to HCD (High Energy Collisional Dissociation). An isolation mass window of 2.0 m/z was for the precursor ion selection, and normalized collision energy of 27% was used for fragmentation. A 15-s duration was used for the dynamic exclusion.

Tandem mass spectra were extracted, and charge state was deconvoluted by Proteome Discoverer (Thermo Fisher). MS/MS samples were analyzed using X! Tandem (The GPM, thegpm.org; version TORNADO (2013.02.01.1)). X! Tandem was set up to search (20,180,405 Uniprot Mouse) database (108,924 entries), the cRAP database of common laboratory contaminants (www.thegpm.org/crap; 114 entries) plus an equal number of reverse protein sequences assuming the digestion enzyme trypsin. X! Tandem was searched with a fragment ion mass tolerance of 20 PPM and a parent ion tolerance of 20 PPM. Deamidation of asparagine and glutamine, oxidation of methionine and tryptophan, sulphone of methionine, tryptophan oxidation to formylkynurenin of tryptophan and acetylation of the n-terminus were specified in X! Tandem as variable modifications.

Scaffold (version 4.4.1, Proteome Software) was used to validate MS/MS-based peptide and protein identifications. Peptide identifications were accepted if they could be established at greater than 85% probability by the Scaffold Local FDR algorithm. Protein identifications were accepted if they could be established at greater than 80% probability to achieve an FDR less than 5% and contained at least 1 identified peptide. Protein probabilities were assigned by the ProteinProphet algorithm. Proteins that contained similar peptides and could not be differentiated based on MS/MS analysis alone were grouped to satisfy the principles of parsimony. Proteins sharing significant peptide evidence were grouped into clusters.

### Statistical analysis

Two-group comparisons between control and test samples were analyzed using Unpaired Student’s t tests. Kaplan–Meier curve analyses were carried out by the log-rank test. A P value < 0.05 was considered significant. GraphPad Prism 10 software (GraphPad Prism Software, Inc., La Jolla, CA) was used for all statistical evaluations.

## Electronic supplementary material

Below is the link to the electronic supplementary material.

Supplementary Information.

## Data Availability

Data is provided within the manuscript.
