# Supplementary material for: The efficacy of an embryonic stem cell-based vaccine for lung cancer prevention depends on the undifferentiated state of the stem cells
Source: Sci Rep. 2024 Dec 30;14:32127. doi: 10.1038/s41598-024-83932-0 (PMC11685895; doi:10.1038/s41598-024-83932-0)
Supplement: Supplementary file 1 — Supplementary Information. [file 41598_2024_83932_MOESM1_ESM.pdf]

SUPPLEMENTARY MATERIALS

The Efficacy of an Embryonic Stem Cell-Based Vaccine for Lung Cancer Prevention Depends on The Undifferentiated State of The Stem Cells

Shuhan Meng, Aaron G. Whitt, John W. Eaton, Kavitha Yaddanapudi, Chi Li

SUPPLEMENTARY FIGURE LEGENDS

Supplementary figure 1. Evaluating the expression of pluripotency and differentiation markers in ES-D3 cells by flow cytometry.

The expression of SSEA-1, Oct-3/4 and SSEA-4 were examined by flow cytometry. Parental and differentiated ES-D3 cells were defined by gating of forward scatter (FSC) and side scatter (SSC). Numbers in the histogram plots represent the percentages of subpopulations positive for a particular fluorophore.

Supplementary figure 2. The effects of differentiation of ES-D3 cells on their activities against tumor growth.

C57BL/6 mice were vaccinated twice (days 0 and 7) with 1 x PBS (vehicle control), parental ES-D3 cells + STO/GM-CSF or differentiated ES-D3 cells + STO/GM-CSF. On day 14, LLC cells (0.15×10^6) was inoculated into mice by subcutaneous injection. Tumor growth was determined by dull-edged Vernier calipers. Tumor volumes are presented as means ± standard deviations. **(A)** Tumor growth in male mice is presented: 1 x PBS (n=8), parental ES-D3 cells + STO/GM-CSF (n=4), or differentiated ES-D3 cells + STO/GM-CSF (n=7). Student's unpaired t test; Asterisk (*) indicates $p < 0.05$. **(B)** Tumor growth in female mice is shown: 1 x PBS (n=8), parental ES-D3 cells + STO/GM-CSF (n=4), or differentiated ES-D3 cells + STO/GM-CSF (n=6). Student's unpaired t test; Asterisk (*) indicates $p < 0.05$; Asterisks (**) indicate $p < 0.01$. **(C)** The data shown in (A) and (B) are combined: 1 x PBS (n=16), parental ES-D3 cells + STO/GM-CSF (n=8) or differentiated ES-D3 cells + STO/GM-CSF (n=13). Student's unpaired t test; Asterisks (**) indicate $p < 0.01$.

Supplementary figure 3. Identification of the antigens specific for the antibodies generated by ESC vaccines.

The schematic depiction of a combined affinity chromatography shotgun immunoproteomics approach. C57BL/6 mice were immunized with a vaccine composed of parental ES-D3 cells + STO/GM-CSF or exosomes from GM-CSF-expressing ES-D3 cells. Lysates containing antigens of cell- or exosomes-based vaccine were loaded to Protein A agarose beads bound with serum antibodies. LC-MS/MS studies were conducted to identify the antigens bound to the beads.

Supplementary figure 4. Summary of antigens that are specific to vaccine-generated antibodies.

Antigens were identified by an affinity chromatography shotgun immunoproteomics strategy. **(A)** Numbers of antigens present in the serum of control mice or mice immunized with the vaccine composed of parental ES-D3 cells + STO/GM-CSF. **(B)** Numbers of antigens presented in the serum of control mice or mice immunized with exosome-based vaccine.

Supplementary figure 5. Summary of antigens recognized by serum antibodies of mice vaccinated with intact ES-D3 cells or exosomes derived from ES-D3 cells.

(A) The number of shared candidate antigens specific to serum antibodies generated by ES-D3-cell-based vaccine and ES-D3-exosome-based vaccine. **(B)** The number of shared candidate antigens between antigens recognized by serum antibodies in mice immunized with ES-D3-cell-based vaccine and those in Lewis lung carcinoma cells. **(C)**

The number of shared candidate antigens between antigens specific to serum antibodies produced by ES-D3-exosome-based vaccine and those in Lewis lung carcinoma cells.

SUPPLEMENTARY TABLE LEGENDS

Supplemental Table 1. Mouse antibodies used in the study of pluripotency and differentiation of ES-D3 cells.

The information about the antibodies used for the experiments examining pluripotency and differentiation of ES-D3 cells.

Supplementary table 2. Comparison of relative abundance of antigens recognized by serum antibodies of control mice or mice vaccinated with ES-D3 cells.

The list of relative abundance of antigens recognized by antibodies in the serum of mice administered with 1 x PBS (control) or parental ES-D3 cells + STO fibroblasts expressing GM-CSF (immunized). Protein abundance was normalized against immunoglobulin G-binding protein A using the following formula: relative protein abundance = abundance value of an antigen / abundance value of immunoglobulin G-binding protein A x 10,000. Data presented are the average values of two independent experiments. "OS" stands for OrganismName, scientific name of the organism of the UniProtKB entry. "OX" stands for OrganismIdentifier, unique identifier of the source organism, assigned by the NCBI. "GN" stands for GeneName, the first gene name of the UniProtKB entry. "PE" stands for ProteinExistence, the numerical value describing

the evidence for the existence of the protein. “SV” stands for SequenceVersion, the version number of the sequence. Keratin members are marked with an asterisk (*).

Supplementary table 3. Relative abundance of antigens recognized by serum antibodies of mice with or without vaccination with exosomes from ES-D3 cells.
The abundance of antigens recognized by antibodies in the serum of control mice (control) and the mice vaccinated with exosomes from ES-D3 cells expressing GM-CSF (immunized). The abundance of antigens was normalized against immunoglobulin G-binding protein A using the following formula: relative protein abundance = abundance value of an antigen / abundance value of immunoglobulin G-binding protein A x 10,000. Data presented are the values from one experiment. “OS” stands for OrganismName, scientific name of the organism of the UniProtKB entry. “OX” stands for OrganismIdentifier, unique identifier of the source organism, assigned by the NCBI. “GN” stands for GeneName, the first gene name of the UniProtKB entry. “PE” stands for ProteinExistence, the numerical value describing the evidence for the existence of the protein. “SV” stands for SequenceVersion, the version number of the sequence. Keratin members are marked with an asterisk (*).

Supplementary table 4. ES-D3-cell-based vaccine and vaccine based on exosomes from ES-D3 cells share candidate antigens.
Shared candidate antigens listed in Supplementary table 2 (identified for serum antibodies of the mice immunized with intact ES-D3 cells + STO fibroblasts expressing GM-CSF) and Supplementary table 3 (identified for serum antibodies of the mice

vaccinated with exosomes from ES-D3 cells expressing GM-CSF). Ratios of relative protein abundance in the serum of control mice (Control) and relative protein abundance in the serum of immunized mice (Immunized) are presented. "OS" stands for OrganismName, scientific name of the organism of the UniProtKB entry. "OX" stands for OrganismIdentifier, unique identifier of the source organism, assigned by the NCBI. "GN" stands for GeneName, the first gene name of the UniProtKB entry. "PE" stands for ProteinExistence, the numerical value describing the evidence for the existence of the protein. "SV" stands for SequenceVersion, the version number of the sequence. Keratin members are marked with an asterisk (*).

Supplementary table 5. Proteins identified in the lysates of Lewis lung carcinoma cells.

Whole cell extract lysate was prepared from Lewis lung carcinoma cells. The complete profile of proteins present in the lysate was determined by a proteomic approach. The list of 2, 698 proteins identified in the lysates of Lewis lung carcinoma cells along with their molecular weights and abundance. Data presented are the values from one experiment. "OS" stands for OrganismName, scientific name of the organism of the UniProtKB entry. "OX" stands for OrganismIdentifier, unique identifier of the source organism, assigned by the NCBI. "GN" stands for GeneName, the first gene name of the UniProtKB entry. "PE" stands for ProteinExistence, the numerical value describing the evidence for the existence of the protein). "SV" stands for SequenceVersion, the version number of the sequence. Keratin members are marked with an asterisk (*).

Meng et al., Supplementary figure 1

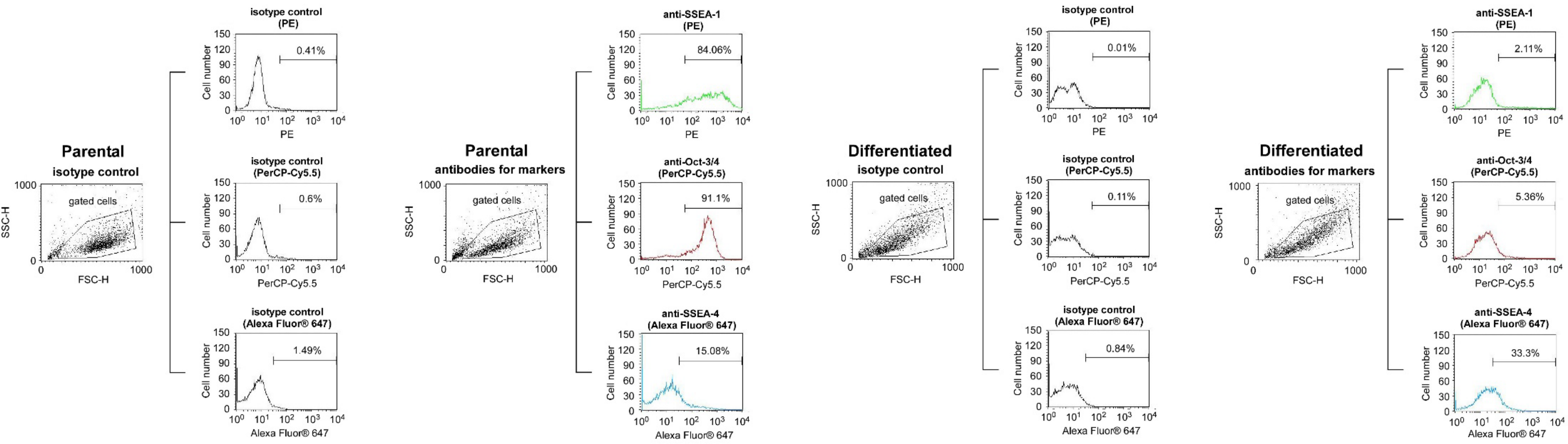

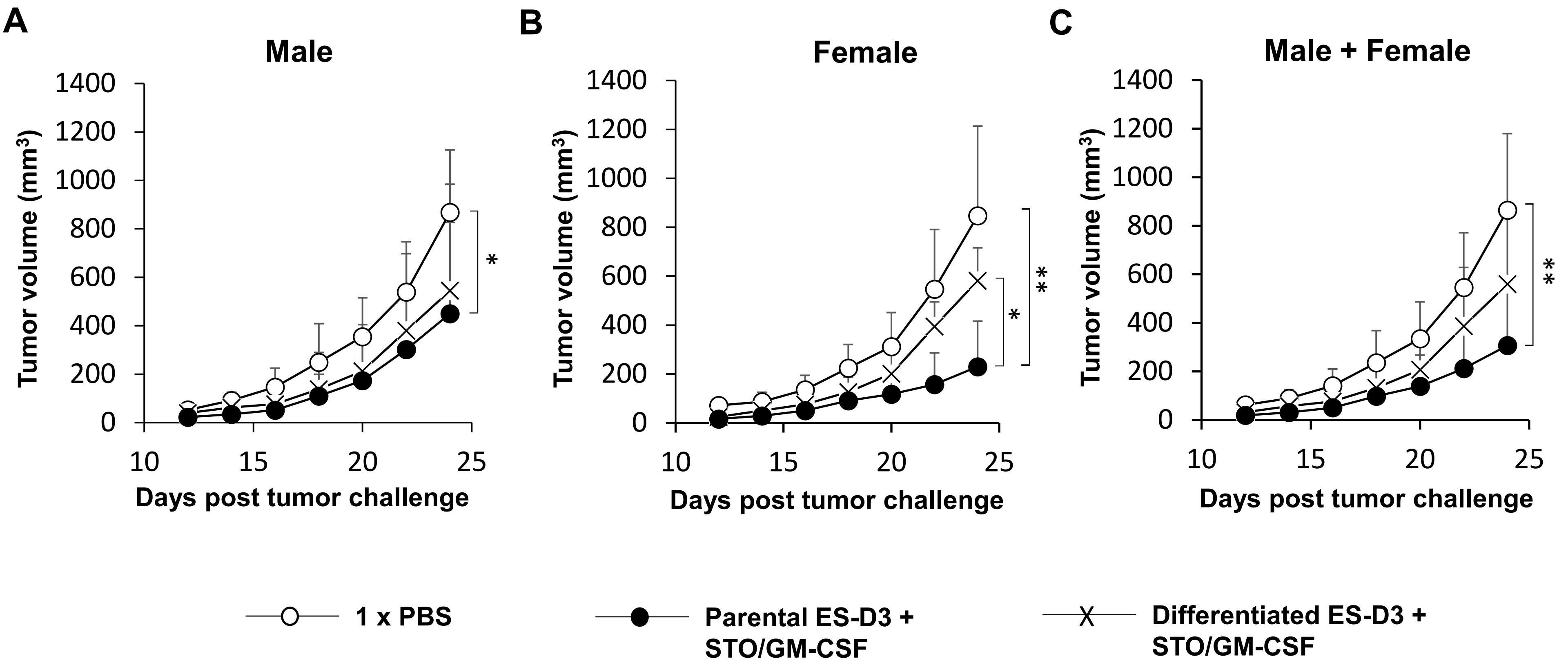

A
Male
B
Female
C
Male + Female
Tumor volume (mm3)
Days post tumor challenge
0
200
400
600
800
1000
1200
1400
10
15
20
25
*
**
1 x PBS
Parental ES-D3 + STO/GM-CSF
Differentiated ES-D3 + STO/GM-CSF

Meng et al., Supplementary figure 3

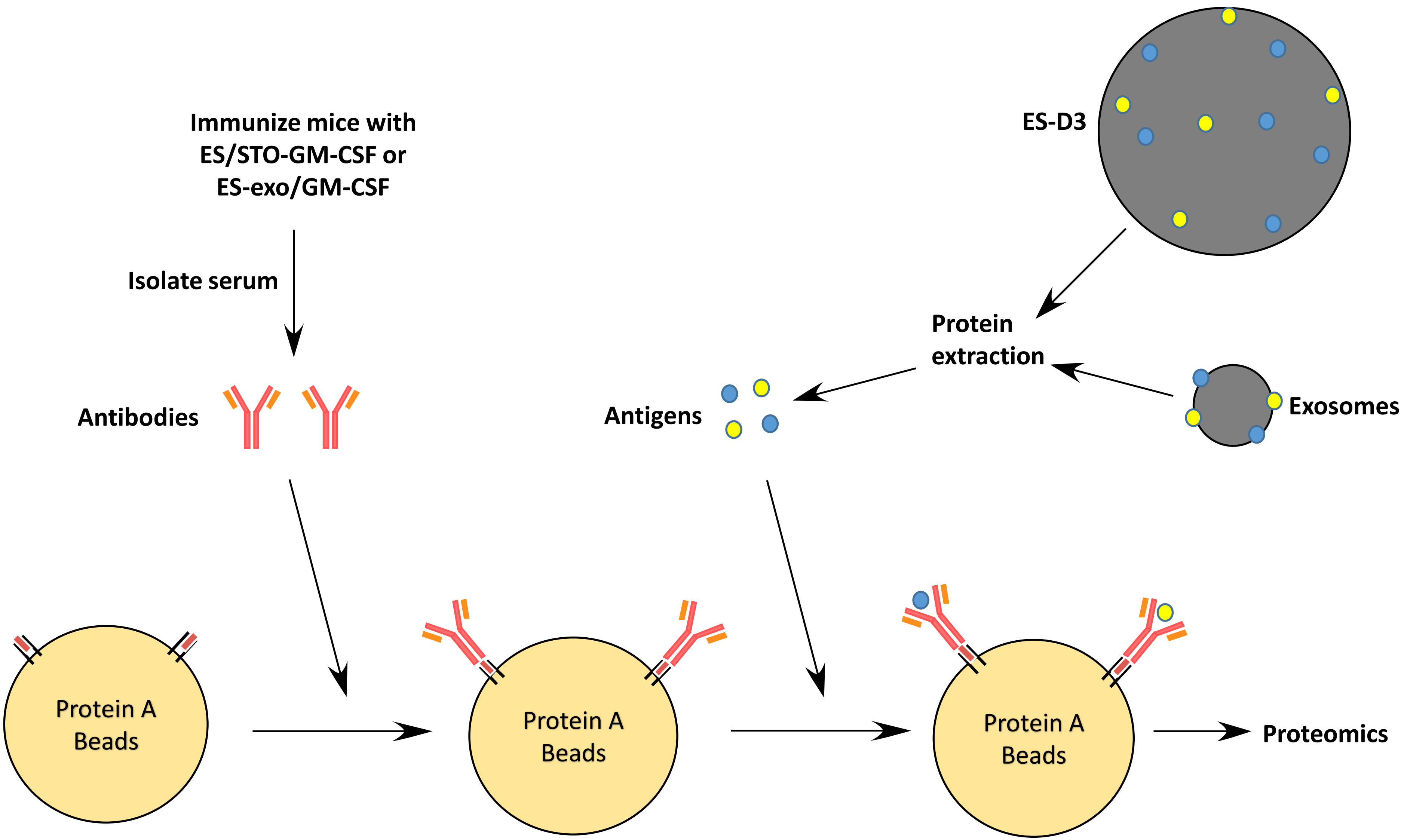

Meng et al., Supplementary figure 4

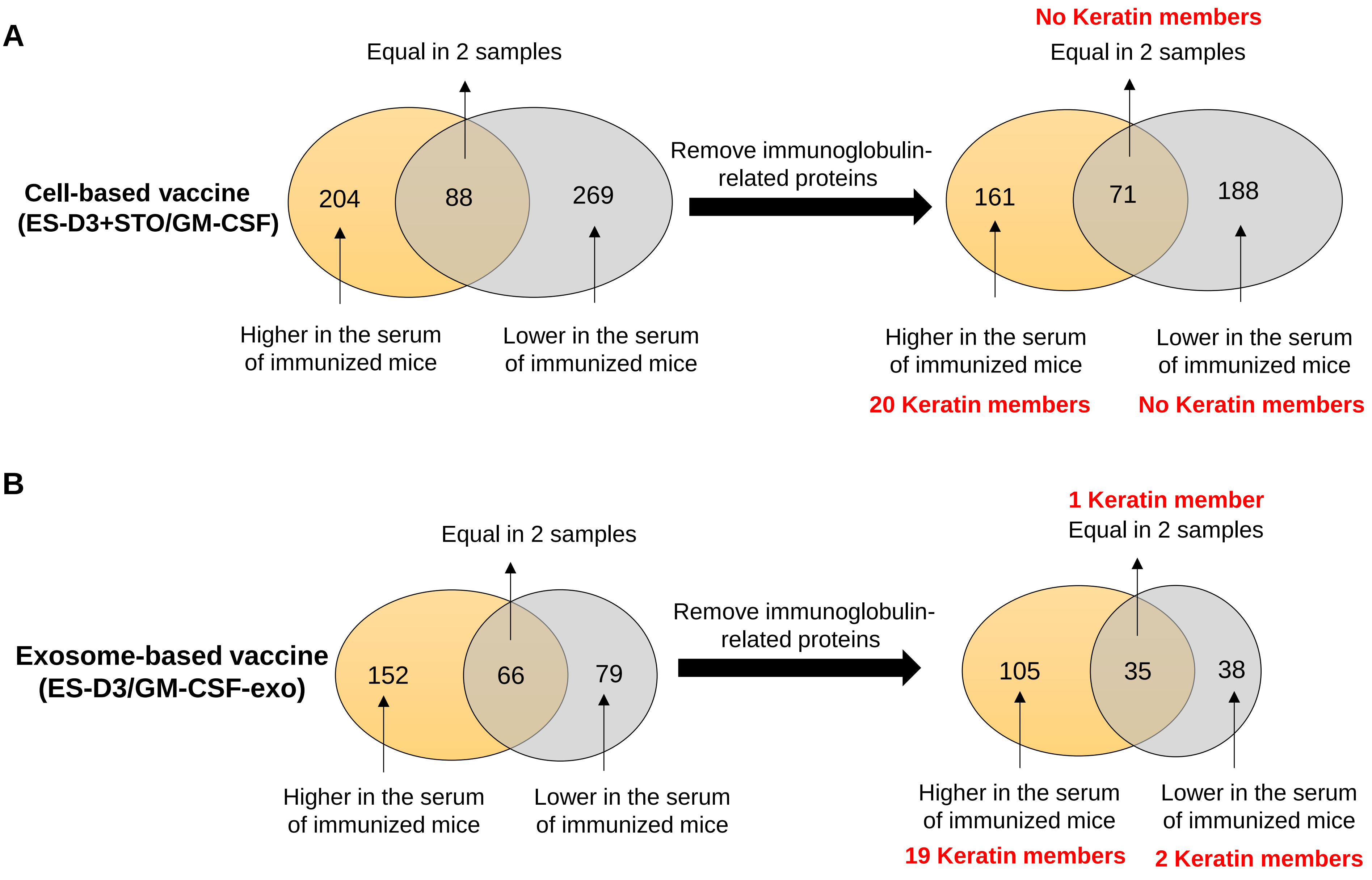

Meng et al., Supplementary figure 5

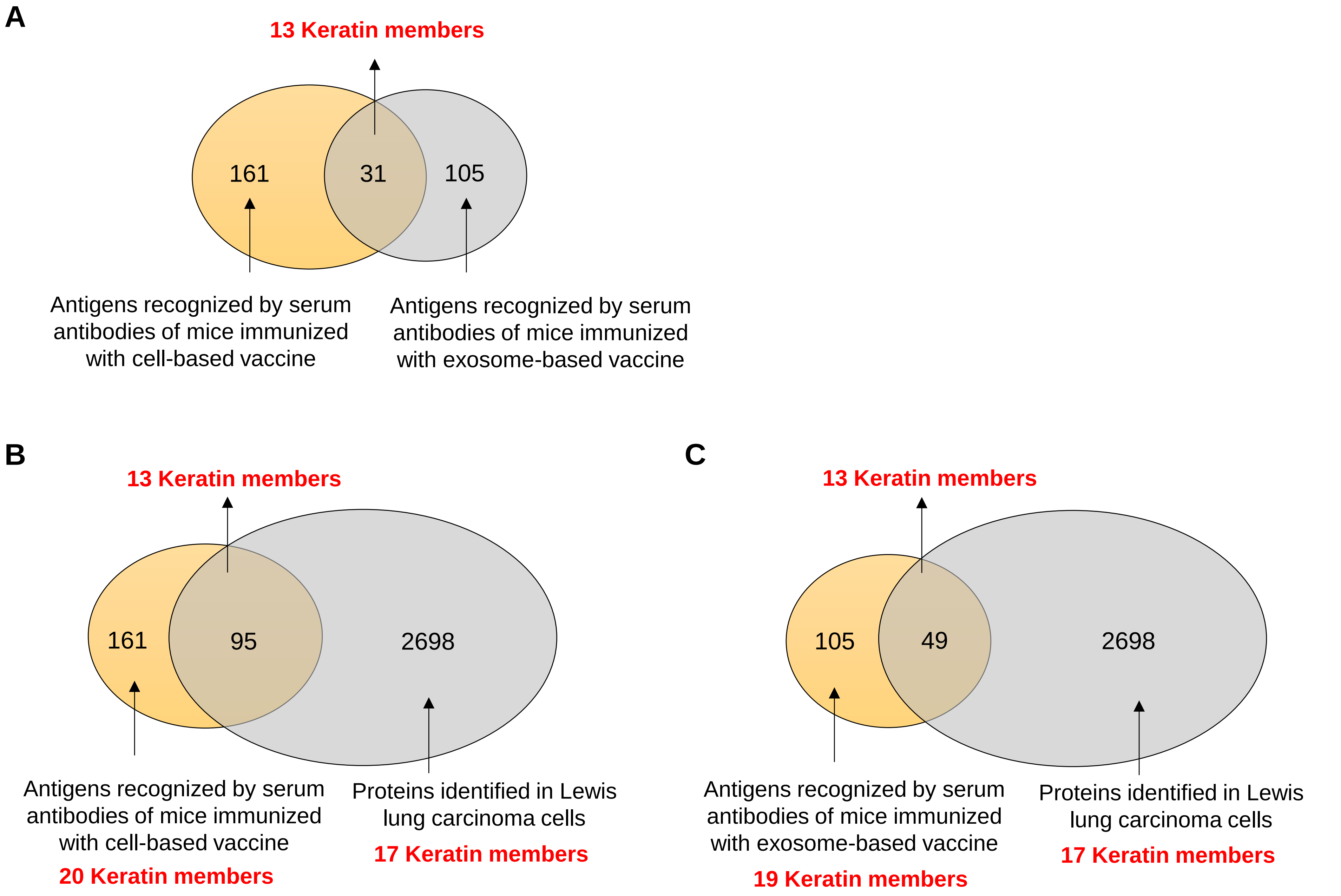

Meng et al., Supplementary table 1

Antibody	Fluorophore	Clone	Manufacturer
SSEA-1	PE	MC480	BD Biosciences
Oct3/4	PerCP-Cy5.5	40/Oct-3	BD Biosciences
SSEA-4	Alexa Fluor® 647	MC813	BD Biosciences
IgM, κ isotype control	PE	G155-228	BD Biosciences
IgG1, κ isotype control	PerCP-Cy5.5	X40	BD Biosciences
IgG3, κ isotype control	Alexa Fluor® 647	J606	BD Biosciences

Meng et al., Supplementary table 2

Antigens	Control (C)	Immunized (I)	Ratio (C/I)
14-3-3 protein theta (Fragment) OS=Mus musculus OX=10090 GN=Ywhaq PE=1 SV=1	0	7.8	0
14-3-3 protein zeta/delta OS=Mus musculus OX=10090 GN=Ywhaz PE=1 SV=1	0	7.8	0
40S ribosomal protein S26 OS=Mus musculus OX=10090 GN=Rps26 PE=1 SV=3	0	7.8	0
40S ribosomal protein S30 OS=Mus musculus OX=10090 GN=Fau PE=1 SV=1	0	4.0	0
40S ribosomal protein S7 OS=Mus musculus OX=10090 GN=Rps7 PE=2 SV=1	0	3.8	0
60S ribosomal protein L23 OS=Mus musculus OX=10090 GN=Rpl23 PE=1 SV=1	0	11.6	0
Acidic leucine-rich nuclear phosphoprotein 32 family member A (Fragment) OS=Mus musculus OX=10090 GN=Anp32a PE=1 SV=1	0	4.0	0
Adenylyl cyclase-associated protein 1 OS=Mus musculus OX=10090 GN=Cap1 PE=1 SV=4	0	7.6	0
Alanine--tRNA ligase, cytoplasmic OS=Mus musculus OX=10090 GN=Aars PE=1 SV=1	0	3.8	0
Alkylated DNA repair protein alkB homolog 8 OS=Mus musculus OX=10090 GN=Alkbh8 PE=1 SV=1	0	3.8	0
Alpha-actinin-4 OS=Mus musculus OX=10090 GN=Actn4 PE=1 SV=1	0	19.1	0
Aminoacyl tRNA synthase complex-interacting multifunctional protein 2 OS=Mus musculus OX=10090 GN=Aimp2 PE=1 SV=2	0	7.6	0
Amyloid-beta A4 precursor protein-binding family B member 2 OS=Mus musculus OX=10090 GN=Apbb2 PE=1 SV=1	0	3.8	0
Antithrombin-III OS=Mus musculus OX=10090 GN=Serpinc1 PE=1 SV=1	0	3.8	0
Beta-2-syntrophin OS=Mus musculus OX=10090 GN=Sntb2 PE=1 SV=2	0	4.0	0
Beta-galactosidase-1-like protein (Fragment) OS=Mus musculus OX=10090 GN=Glb1l PE=1 SV=1	0	4.0	0
Calponin-3 OS=Mus musculus OX=10090 GN=Cnn3 PE=1 SV=1	0	4.0	0
Catalase OS=Mus musculus OX=10090 GN=Cat PE=1 SV=4	0	4.0	0
Centrosomal protein of 290 kDa OS=Mus musculus OX=10090 GN=Cep290 PE=1 SV=1	0	4.0	0
Ceruloplasmin OS=Mus musculus OX=10090 GN=Cp PE=1 SV=2	0	3.8	0

Cilia- and flagella-associated protein 43 OS=Mus musculus OX=10090 GN=Cfap43 PE=1 SV=1	0	3.8	0
Cleavage and polyadenylation specificity factor subunit 1 OS=Mus musculus OX=10090 GN=Cpsf1 PE=1 SV=1	0	4.0	0
Collagen alpha-2(I) chain OS=Mus musculus OX=10090 GN=Col1a2 PE=1 SV=2	0	3.8	0
Complement component 6 OS=Mus musculus OX=10090 GN=C6 PE=1 SV=2	0	4.0	0
Complement component C8 beta chain OS=Mus musculus OX=10090 GN=C8b PE=1 SV=1	0	3.8	0
Complement factor H OS=Mus musculus OX=10090 GN=Cfh PE=1 SV=2	0	7.8	0
Cysteine and glycine-rich protein 1 OS=Mus musculus OX=10090 GN=Csrp1 PE=1 SV=3	0	4.0	0
Cysteine-rich perinuclear theca 3 OS=Mus musculus OX=10090 GN=Cypt3 PE=2 SV=1	0	4.0	0
D-3-phosphoglycerate dehydrogenase OS=Mus musculus OX=10090 GN=Phgdh PE=1 SV=3	0	7.8	0
Desmoplakin OS=Mus musculus OX=10090 GN=Dsp PE=1 SV=1	0	11.8	0
Destrin OS=Mus musculus OX=10090 GN=Dstn PE=1 SV=3	0	7.8	0
ELAV-like protein 1 OS=Mus musculus OX=10090 GN=Elavl1 PE=1 SV=2	0	4.0	0
Elongation factor 1-alpha 2 OS=Mus musculus OX=10090 GN=Eef1a2 PE=1 SV=1	0	23.8	0
ER membrane protein complex subunit 6 OS=Mus musculus OX=10090 GN=Emc6 PE=1 SV=1	0	3.8	0
Four and a half LIM domains protein 3 OS=Mus musculus OX=10090 GN=Fhl3 PE=1 SV=2	0	3.8	0
Fragile X mental retardation syndrome-related protein 2 OS=Mus musculus OX=10090 GN=Fxr2 PE=1 SV=1	0	3.8	0
GTP-binding nuclear protein Ran, testis-specific isoform OS=Mus musculus OX=10090 GN=Rasl2-9 PE=2 SV=1	0	7.6	0
Guanine nucleotide-binding protein-like 3 OS=Mus musculus OX=10090 GN=Gnl3 PE=1 SV=2	0	7.8	0
Heat shock 70 kDa protein 4 OS=Mus musculus OX=10090 GN=Hspa4 PE=1 SV=1	0	4.0	0
Heat shock protein 75 kDa, mitochondrial OS=Mus musculus OX=10090 GN=Trap1 PE=1 SV=1	0	3.8	0
Heat shock protein beta-1 OS=Mus musculus OX=10090 GN=Hspb1 PE=1 SV=3	0	7.9	0
Heterogeneous nuclear ribonucleoprotein Q OS=Mus musculus OX=10090 GN=Syncrip PE=1 SV=2	0	4.0	0
Histone deacetylase 1 OS=Mus musculus OX=10090 GN=Hdac1 PE=1 SV=1	0	4.0	0

	Histone H1.5 OS=Mus musculus OX=10090 GN=Hist1h1b PE=1 SV=2	0	3.8	0
	Histone H2A type 1-B OS=Mus musculus OX=10090 GN=Hist1h2ab PE=1 SV=1	0	3.8	0
	Hydroxymethylglutaryl-CoA synthase, mitochondrial OS=Mus musculus OX=10090 GN=Hmgcs2 PE=1 SV=2	0	4.0	0
	Inter-alpha-trypsin inhibitor heavy chain H1 OS=Mus musculus OX=10090 GN=Itih1 PE=1 SV=2	0	3.8	0
	Kelch-like protein 22 OS=Mus musculus OX=10090 GN=Klhl22 PE=1 SV=1	0	3.8	0
*	Keratin, type II cytoskeletal 4 OS=Mus musculus OX=10090 GN=Krt4 PE=1 SV=2	0	31.5	0
*	Keratin, type II cytoskeletal 7 OS=Mus musculus OX=10090 GN=Krt7 PE=1 SV=1	0	7.6	0
*	Keratin, type II cytoskeletal 72 OS=Mus musculus OX=10090 GN=Krt72 PE=3 SV=1	0	39.3	0
*	Keratin, type II cytoskeletal 80 OS=Mus musculus OX=10090 GN=Krt80 PE=1 SV=1	0	4.0	0
	Krueppel-like factor 8 OS=Mus musculus OX=10090 GN=Klf8 PE=2 SV=1	0	3.8	0
	Low molecular weight phosphotyrosine protein phosphatase OS=Mus musculus OX=10090 GN=Acp1 PE=1 SV=3	0	4.0	0
	MCG140784 OS=Mus musculus OX=10090 GN=Try10 PE=1 SV=1	0	19.4	0
	Methylglutaconyl-CoA hydratase, mitochondrial OS=Mus musculus OX=10090 GN=Auh PE=1 SV=1	0	4.0	0
	MPN domain-containing protein OS=Mus musculus OX=10090 GN=Mpnd PE=1 SV=1	0	4.0	0
	NAD kinase 2, mitochondrial OS=Mus musculus OX=10090 GN=Nadk2 PE=1 SV=2	0	30.5	0
	Nucleolar protein 56 OS=Mus musculus OX=10090 GN=Nop56 PE=1 SV=2	0	3.8	0
	Nucleoside diphosphate kinase B OS=Mus musculus OX=10090 GN=Nme2 PE=1 SV=1	0	3.8	0
	Oxysterol-binding protein-related protein 1 OS=Mus musculus OX=10090 GN=Osbpl1a PE=1 SV=2	0	3.8	0
	Phenylalanine--tRNA ligase alpha subunit OS=Mus musculus OX=10090 GN=Farsa PE=1 SV=1	0	4.0	0
	Phospholipid phosphatase 1 OS=Mus musculus OX=10090 GN=Plpp1 PE=1 SV=1	0	7.6	0
	Proteasome subunit alpha type-2 OS=Mus musculus OX=10090 GN=Psma2 PE=1 SV=3	0	4.0	0
	Protein FAM91A1 OS=Mus musculus OX=10090 GN=Fam91a1 PE=1 SV=1	0	7.9	0
	Protein SGT1 homolog OS=Mus musculus OX=10090 GN=Sugt1 PE=1 SV=3	0	3.8	0
	Proteolipid protein 2 OS=Mus musculus OX=10090 GN=Plp2 PE=1 SV=1	0	3.8	0

Pyruvate dehydrogenase E1 component subunit beta, mitochondrial OS=Mus musculus OX=10090 GN=Pdhb PE=1 SV=1	0	15.3	0
RAB6-interacting golgin OS=Mus musculus OX=10090 GN=Gorab PE=1 SV=1	0	4.0	0
Ras and Rab interactor 1 OS=Mus musculus OX=10090 GN=Rin1 PE=1 SV=1	0	3.8	0
Ras-related protein Rab-11A OS=Mus musculus OX=10090 GN=Rab11a PE=1 SV=3	0	3.8	0
RING finger protein 214 OS=Mus musculus OX=10090 GN=Rnf214 PE=1 SV=1	0	4.0	0
Sjoegren syndrome/scleroderma autoantigen 1 homolog OS=Mus musculus OX=10090 GN=Sssca1 PE=1 SV=1	0	7.8	0
Sjoegren syndrome/scleroderma autoantigen 1 homolog OS=Mus musculus OX=10090 GN=Sssca1 PE=1 SV=1	0	3.8	0
Stomatin-like protein 2, mitochondrial OS=Mus musculus OX=10090 GN=Stoml2 PE=1 SV=1	0	3.8	0
TAR DNA-binding protein 43 OS=Mus musculus OX=10090 GN=Tardbp PE=1 SV=1	0	3.8	0
Thioredoxin-like protein 1 OS=Mus musculus OX=10090 GN=Txnl1 PE=1 SV=3	0	4.0	0
Transcription elongation factor A protein 1 OS=Mus musculus OX=10090 GN=Tcea1 PE=1 SV=2	0	4.0	0
Transforming protein RhoA OS=Mus musculus OX=10090 GN=Rhoa PE=1 SV=1	0	7.8	0
Transport and Golgi organization protein 1 homolog OS=Mus musculus OX=10090 GN=Mia3 PE=1 SV=1	0	22.9	0
Transport and Golgi organization protein 1 homolog OS=Mus musculus OX=10090 GN=Mia3 PE=1 SV=2	0	119.9	0
Ubiquitin-conjugating enzyme E2 E3 (Fragment) OS=Mus musculus OX=10090 GN=Ube2e3 PE=1 SV=8	0	3.8	0
Ubiquitin-conjugating enzyme E2 variant 1 OS=Mus musculus OX=10090 GN=Ube2v1 PE=1 SV=1	0	7.9	0
UBX domain-containing protein 1 OS=Mus musculus OX=10090 GN=Ubxn1 PE=1 SV=1	0	3.8	0
Voltage-dependent anion-selective channel protein 1 OS=Mus musculus OX=10090 GN=Vdac1 PE=1 SV=1	0	7.6	0
WD repeat-containing protein 11 OS=Mus musculus OX=10090 GN=Wdr11 PE=1 SV=1	0	19.9	0
Pyruvate dehydrogenase protein X component, mitochondrial OS=Mus musculus OX=10090 GN=Pdhx PE=1 SV=1	3.9	61.5	0.06
Pyruvate dehydrogenase E1 component subunit alpha, somatic form, mitochondrial OS=Mus musculus OX=10090 GN=Pdha1 PE=1 SV=1	4.1	34.7	0.12

	Dihydrolipoyllysine-residue acetyltransferase component of pyruvate dehydrogenase complex, mitochondrial OS=Mus musculus OX=10090 GN=Dlat PE=1 SV=2	31.9	234.2	0.14
	Inosine-5'-monophosphate dehydrogenase 2 OS=Mus musculus OX=10090 GN=Impdh2 PE=1 SV=2	8.1	51.6	0.16
	Junction plakoglobin OS=Mus musculus OX=10090 GN=Jup PE=1 SV=3	4.1	23.1	0.18
	Aspartate--tRNA ligase, cytoplasmic OS=Mus musculus OX=10090 GN=Dars PE=1 SV=2	4.1	22.9	0.18
	40S ribosomal protein S6 OS=Mus musculus OX=10090 GN=Rps6 PE=1 SV=1	8.3	30.8	0.27
*	Keratin, type II cytoskeletal 73 OS=Mus musculus OX=10090 GN=Krt73 PE=1 SV=1	20.5	70.4	0.29
*	Keratin, type II cytoskeletal 2 oral OS=Mus musculus OX=10090 GN=Krt76 PE=1 SV=1	16.1	47.0	0.34
*	Keratin 78 OS=Mus musculus OX=10090 GN=Krt78 PE=1 SV=1	12.0	35.0	0.34
	Ribosomal L1 domain-containing protein 1 OS=Mus musculus OX=10090 GN=Rsl1d1 PE=1 SV=1	4.1	11.8	0.35
*	Keratin, type I cytoskeletal 19 OS=Mus musculus OX=10090 GN=Krt19 PE=1 SV=1	12.0	31.3	0.38
	40S ribosomal protein S15a OS=Mus musculus OX=10090 GN=Rps15a PE=1 SV=2	7.9	19.5	0.40
*	Keratin, type II cytoskeletal 79 OS=Mus musculus OX=10090 GN=Krt79 PE=1 SV=2	28.9	70.4	0.41
*	Keratin, type II cytoskeletal 1b OS=Mus musculus OX=10090 GN=Krt77 PE=1 SV=1	69.1	148.6	0.46
*	Keratin, type II cytoskeletal 6A OS=Mus musculus OX=10090 GN=Krt6a PE=1 SV=3	32.5	66.6	0.49
	Stress-induced-phosphoprotein 1 OS=Mus musculus OX=10090 GN=Stip1 PE=1 SV=1	3.9	7.9	0.50
	DNA-directed RNA polymerases I, II, and III subunit RPABC3 OS=Mus musculus OX=10090 GN=Polr2h PE=1 SV=1	3.9	7.8	0.51
	Phosphoribosyl pyrophosphate synthase-associated protein 1 OS=Mus musculus OX=10090 GN=Prpsap1 PE=1 SV=1	3.9	7.8	0.51
	14-3-3 protein gamma OS=Mus musculus OX=10090 GN=Ywhag PE=1 SV=2	12.0	23.4	0.51
	14-3-3 protein epsilon OS=Mus musculus OX=10090 GN=Ywhae PE=1 SV=1	4.1	7.8	0.53
	ATP synthase subunit d, mitochondrial OS=Mus musculus OX=10090 GN=Atp5pd PE=1 SV=3	4.1	7.8	0.53
	CAD protein OS=Mus musculus OX=10090 GN=Cad PE=1 SV=1	4.1	7.8	0.53
	Dolichyl-diphosphooligosaccharide--protein glycosyltransferase subunit 1 OS=Mus musculus OX=10090 GN=Rpn1 PE=1 SV=1	4.1	7.8	0.53

	Protein transport protein Sec61 subunit beta OS=Mus musculus OX=10090 GN=Sec61b PE=1 SV=3	4.1	7.8	0.53
	Thrombospondin-1 OS=Mus musculus OX=10090 GN=Thbs1 PE=1 SV=1	4.1	7.8	0.53
*	Keratin, type II cytoskeletal 5 OS=Mus musculus OX=10090 GN=Krt5 PE=1 SV=1	81.1	152.4	0.53
*	Keratin, type I cytoskeletal 10 OS=Mus musculus OX=10090 GN=Krt10 PE=1 SV=3	101.8	191.0	0.53
*	Keratin 15, isoform CRA_a OS=Mus musculus OX=10090 GN=Krt15 PE=1 SV=1	48.6	90.3	0.54
	14-3-3 protein beta/alpha OS=Mus musculus OX=10090 GN=Ywhab PE=1 SV=3	4.1	7.6	0.54
	Plasma protease C1 inhibitor OS=Mus musculus OX=10090 GN=Serping1 PE=1 SV=3	24.2	42.8	0.57
*	Keratin, type II cytoskeletal 2 epidermal OS=Mus musculus OX=10090 GN=Krt2 PE=1 SV=1	65.0	113.3	0.57
*	Keratin, type I cytoskeletal 16 OS=Mus musculus OX=10090 GN=Krt16 PE=1 SV=3	40.9	70.2	0.58
	40S ribosomal protein S2 OS=Mus musculus OX=10090 GN=Rps2 PE=1 SV=3	12.0	19.5	0.61
	DNA replication licensing factor MCM5 OS=Mus musculus OX=10090 GN=Mcm5 PE=1 SV=2	12.2	19.4	0.63
	Predicted pseudogene 5478 OS=Mus musculus OX=10090 GN=Gm5478 PE=1 SV=1	32.7	50.9	0.64
*	Keratin, type II cytoskeletal 8 OS=Mus musculus OX=10090 GN=Krt8 PE=1 SV=4	44.7	66.4	0.67
	40S ribosomal protein S3a OS=Mus musculus OX=10090 GN=Rps3a PE=1 SV=3	16.1	23.5	0.69
	Histone H2A.Z OS=Mus musculus OX=10090 GN=H2afz PE=1 SV=2	8.1	11.8	0.69
	Protein RCC2 OS=Mus musculus OX=10090 GN=Rcc2 PE=1 SV=1	8.1	11.8	0.69
	40S ribosomal protein S17 OS=Mus musculus OX=10090 GN=Rps17 PE=1 SV=2	8.3	11.9	0.69
	60S ribosomal protein L38 OS=Mus musculus OX=10090 GN=Rpl38 PE=1 SV=3	24.4	35.1	0.69
	40S ribosomal protein S25 OS=Mus musculus OX=10090 GN=Rps25 PE=1 SV=1	8.1	11.6	0.70
	Eukaryotic translation initiation factor 2 subunit 3, X-linked OS=Mus musculus OX=10090 GN=Eif2s3x PE=1 SV=2	8.1	11.6	0.70
	Polymeric immunoglobulin receptor OS=Mus musculus OX=10090 GN=Pigr PE=1 SV=1	16.3	23.4	0.70
*	Keratin, type II cytoskeletal 1 OS=Mus musculus OX=10090 GN=Krt1 PE=1 SV=4	65.0	90.0	0.72
	Histone H2B type 1-M OS=Mus musculus OX=10090 GN=Hist1h2bm PE=1 SV=2	28.3	38.8	0.73
	Beta-globin OS=Mus musculus OX=10090 GN=Hbb-bs PE=1 SV=1	81.1	109.2	0.74

	Docking protein 2 OS=Mus musculus OX=10090 GN=Dok2 PE=1 SV=1	20.3	27.2	0.75
	60S ribosomal protein L3 OS=Mus musculus OX=10090 GN=Rpl3 PE=1 SV=3	32.1	42.8	0.75
	Hemoglobin subunit alpha OS=Mus musculus OX=10090 GN=Hba PE=1 SV=2	68.5	89.5	0.77
	Histone H1.2 OS=Mus musculus OX=10090 GN=Hist1h1c PE=1 SV=2	24.0	31.2	0.77
	Serum albumin OS=Oryctolagus cuniculus GN=ALB PE=1 SV=2	12.0	15.4	0.78
	40S ribosomal protein S11 OS=Mus musculus OX=10090 GN=Rps11 PE=1 SV=3	12.2	15.6	0.78
	60S ribosomal protein L34 OS=Mus musculus OX=10090 GN=Rpl34 PE=1 SV=2	12.2	15.6	0.78
	Properdin OS=Mus musculus OX=10090 GN=Cfp PE=2 SV=2	12.2	15.6	0.78
	Endoplasmic reticulum chaperone BiP OS=Mus musculus OX=10090 GN=Hspa5 PE=1 SV=3	64.2	77.7	0.83
	Gelsolin OS=Mus musculus OX=10090 GN=Gsn PE=1 SV=3	16.1	19.5	0.83
	Plasminogen activator inhibitor 1 RNA-binding protein OS=Mus musculus OX=10090 GN=Serbp1 PE=1 SV=2	32.5	39.3	0.83
	40S ribosomal protein S18 OS=Mus musculus OX=10090 GN=Rps18 PE=1 SV=3	32.5	39.1	0.83
	Histone H1.4 OS=Mus musculus OX=10090 GN=Hist1h1e PE=1 SV=2	16.1	19.4	0.83
*	Keratin, type I cytoskeletal 17 OS=Mus musculus OX=10090 GN=Krt17 PE=1 SV=3	36.4	43.1	0.85
	60S ribosomal protein L18 OS=Mus musculus OX=10090 GN=Rpl18 PE=1 SV=3	44.5	50.7	0.88
	Transcription intermediary factor 1-beta OS=Mus musculus OX=10090 GN=Trim28 PE=1 SV=3	24.0	27.3	0.88
	40S ribosomal protein S14 OS=Mus musculus OX=10090 GN=Rps14 PE=1 SV=3	24.2	27.3	0.89
	Actin, cytoplasmic 2 (Fragment) OS=Mus musculus OX=10090 GN=Actg1 PE=1 SV=1	149.8	166.9	0.90
	Stress-70 protein, mitochondrial OS=Mus musculus OX=10090 GN=Hspa9 PE=1 SV=3	205.9	226.6	0.91
	Complement C3 OS=Mus musculus OX=10090 GN=C3 PE=1 SV=3	4338.2	4772.6	0.91
*	Keratin, type I cytoskeletal 42 OS=Mus musculus OX=10090 GN=Krt42 PE=1 SV=1	28.3	31.2	0.91
	Mannose-binding protein C OS=Mus musculus OX=10090 GN=Mbl2 PE=1 SV=2	117.0	128.3	0.91
	Pyruvate kinase PKM OS=Mus musculus OX=10090 GN=Pkm PE=1 SV=4	92.7	101.4	0.91
	Heat shock cognate 71 kDa protein OS=Mus musculus OX=10090 GN=Hspa8 PE=1 SV=1	189.6	207.2	0.91

Histone H1.3 OS=Mus musculus OX=10090 GN=Hist1h1d PE=1 SV=2	32.1	35.0	0.92
Protein-L-isoaspartate O-methyltransferase domain-containing protein 1 OS=Mus musculus OX=10090 GN=Pcmtd1 PE=1 SV=1	36.2	38.9	0.93
ATP synthase subunit alpha, mitochondrial OS=Mus musculus OX=10090 GN=Atp5f1a PE=1 SV=1	40.4	42.8	0.94
Actin, alpha cardiac muscle 1 OS=Mus musculus OX=10090 GN=Actc1 PE=1 SV=1	162.0	166.9	0.97
Fibronectin (Fragment) OS=Mus musculus OX=10090 GN=Fn1 PE=1 SV=1	68.5	70.1	0.98
Mannan-binding lectin serine protease 2 OS=Mus musculus OX=10090 GN=Masp2 PE=1 SV=1	84.5	85.8	0.98
Actin, cytoplasmic 1 OS=Mus musculus OX=10090 GN=Actb PE=1 SV=1	234.5	237.0	0.99
60S ribosomal protein L24 OS=Mus musculus OX=10090 GN=Rpl24 PE=1 SV=2	3.9	4.0	0.99
Complement C5 OS=Mus musculus OX=10090 GN=C5 PE=1 SV=2	3.9	4.0	0.99
Elongation factor 2 OS=Mus musculus OX=10090 GN=Eef2 PE=1 SV=2	3.9	4.0	0.99
Ran-specific GTPase-activating protein OS=Mus musculus OX=10090 GN=Ranbp1 PE=1 SV=2	3.9	4.0	0.99
Spliceosome RNA helicase Ddx39b OS=Mus musculus OX=10090 GN=Ddx39b PE=1 SV=1	3.9	4.0	0.99
Vitronectin OS=Mus musculus OX=10090 GN=Vtn PE=1 SV=2	3.9	4.0	0.99
Heterogeneous nuclear ribonucleoprotein L (Fragment) OS=Mus musculus OX=10090 GN=Hnrnpl PE=1 SV=1	12.0	11.9	1.01
Alpha-2-HS-glycoprotein OS=Mus musculus OX=10090 GN=Ahsg PE=1 SV=1	12.0	11.8	1.02
Serine/arginine-rich splicing factor 5 OS=Mus musculus OX=10090 GN=Srsf5 PE=1 SV=2	12.0	11.8	1.02
L-threonine 3-dehydrogenase, mitochondrial OS=Mus musculus OX=10090 GN=Tdh PE=1 SV=1	32.1	31.3	1.03
Heat shock protein HSP 90-beta OS=Mus musculus OX=10090 GN=Hsp90ab1 PE=1 SV=3	48.2	46.9	1.03
Histone H1.1 OS=Mus musculus OX=10090 GN=Hist1h1a PE=1 SV=2	28.2	27.3	1.03
Elongin-C OS=Mus musculus OX=10090 GN=Eloc PE=1 SV=1	36.2	35.1	1.03
60S ribosomal protein L31 OS=Mus musculus OX=10090 GN=Rpl31 PE=1 SV=1	3.9	3.8	1.03
Laminin subunit alpha-3 OS=Mus musculus OX=10090 GN=Lama3 PE=1 SV=4	3.9	3.8	1.03
Myelin expression factor 2 OS=Mus musculus OX=10090 GN=Myef2 PE=1 SV=1	3.9	3.8	1.03

Probable E3 ubiquitin-protein ligase IRF2BPL OS=Mus musculus OX=10090 GN=Irf2bpl PE=1 SV=1	3.9	3.8	1.03
T-complex protein 1 subunit zeta OS=Mus musculus OX=10090 GN=Cct6a PE=1 SV=3	3.9	3.8	1.03
Heat shock protein HSP 90-alpha OS=Mus musculus OX=10090 GN=Hsp90aa1 PE=1 SV=4	64.2	62.2	1.03
40S ribosomal protein S9 OS=Mus musculus OX=10090 GN=Rps9 PE=1 SV=3	12.0	11.6	1.04
60S ribosomal protein L35a OS=Mus musculus OX=10090 GN=Rpl35a PE=1 SV=2	12.0	11.6	1.04
GON-4-like protein OS=Mus musculus OX=10090 GN=Gon4l PE=1 SV=1	12.0	11.6	1.04
T-complex protein 1 subunit theta OS=Mus musculus OX=10090 GN=Cct8 PE=1 SV=3	12.0	11.6	1.04
40S ribosomal protein S5 OS=Mus musculus OX=10090 GN=Rps5 PE=1 SV=3	20.1	19.4	1.04
60S acidic ribosomal protein P0 OS=Mus musculus OX=10090 GN=Rplp0 PE=1 SV=3	24.2	23.4	1.04
40S ribosomal protein S12 OS=Mus musculus OX=10090 GN=Rps12 PE=1 SV=2	8.1	7.8	1.04
40S ribosomal protein S20 OS=Mus musculus OX=10090 GN=Rps20 PE=1 SV=1	16.1	15.6	1.04
40S ribosomal protein S24 OS=Mus musculus OX=10090 GN=Rps24 PE=1 SV=1	8.1	7.8	1.04
60S ribosomal protein L10 OS=Mus musculus OX=10090 GN=Rpl10 PE=1 SV=3	16.1	15.6	1.04
60S ribosomal protein L13a OS=Mus musculus OX=10090 GN=Rpl13a PE=1 SV=4	8.1	7.8	1.04
60S ribosomal protein L14 OS=Mus musculus OX=10090 GN=Rpl14 PE=1 SV=3	8.1	7.8	1.04
60S ribosomal protein L19 OS=Mus musculus OX=10090 GN=Rpl19 PE=1 SV=1	8.1	7.8	1.04
60S ribosomal protein L27a OS=Mus musculus OX=10090 GN=Rpl27a PE=1 SV=5	8.1	7.8	1.04
60S ribosomal protein L32 OS=Mus musculus OX=10090 GN=Rpl32 PE=1 SV=2	8.1	7.8	1.04
60S ribosomal protein L35 OS=Mus musculus OX=10090 GN=Rpl35 PE=1 SV=1	8.1	7.8	1.04
60S ribosomal protein L4 OS=Mus musculus OX=10090 GN=Rpl4 PE=1 SV=3	72.7	70.1	1.04
60S ribosomal protein L8 OS=Mus musculus OX=10090 GN=Rpl8 PE=1 SV=2	8.1	7.8	1.04
Cofilin-1 OS=Mus musculus OX=10090 GN=Cfl1 PE=1 SV=3	16.1	15.6	1.04
Deoxyuridine triphosphatase OS=Mus musculus OX=10090 GN=Dut PE=1 SV=1	8.1	7.8	1.04
DNA replication licensing factor MCM7 OS=Mus musculus OX=10090 GN=Mcm7 PE=1 SV=1	8.1	7.8	1.04

Dynein regulatory complex subunit 6 OS=Mus musculus OX=10090 GN=Fbxl13 PE=1 SV=2	8.1	7.8	1.04
Eukaryotic translation initiation factor 4B OS=Mus musculus OX=10090 GN=Eif4b PE=1 SV=1	8.1	7.8	1.04
Histone H3.3 OS=Mus musculus OX=10090 GN=H3f3a PE=1 SV=2	16.1	15.6	1.04
Importin subunit beta-1 OS=Mus musculus OX=10090 GN=Kpnb1 PE=1 SV=2	8.1	7.8	1.04
Lamin-B2 OS=Mus musculus OX=10090 GN=Lmnb2 PE=1 SV=2	8.1	7.8	1.04
Nebulette (Fragment) OS=Mus musculus OX=10090 GN=Nebl PE=1 SV=1	8.1	7.8	1.04
Phosphoribosyl pyrophosphate synthase-associated protein 2 OS=Mus musculus OX=10090 GN=Prpsap2 PE=1 SV=1	8.1	7.8	1.04
Platelet factor 4 OS=Mus musculus OX=10090 GN=Pf4 PE=1 SV=1	8.1	7.8	1.04
rRNA 2'-O-methyltransferase fibrillarin OS=Mus musculus OX=10090 GN=Fbl PE=1 SV=2	16.1	15.6	1.04
T-complex protein 1 subunit epsilon OS=Mus musculus OX=10090 GN=Cct5 PE=1 SV=1	8.1	7.8	1.04
Translationally-controlled tumor protein OS=Mus musculus OX=10090 GN=Tpt1 PE=1 SV=1	8.1	7.8	1.04
ATP-dependent RNA helicase DDX3X OS=Mus musculus OX=10090 GN=Ddx3x PE=1 SV=3	20.3	19.5	1.04
Putative ATP-dependent RNA helicase Pl10 OS=Mus musculus OX=10090 GN=D1Pas1 PE=1 SV=1	20.3	19.5	1.04
ATP synthase subunit beta, mitochondrial OS=Mus musculus OX=10090 GN=Atp5f1b PE=1 SV=2	12.2	11.8	1.04
60S ribosomal protein L11 OS=Mus musculus OX=10090 GN=Rpl11 PE=1 SV=4	4.1	4.0	1.04
Complement component 7 OS=Mus musculus OX=10090 GN=C7 PE=1 SV=2	4.1	4.0	1.04
Far upstream element-binding protein 2 OS=Mus musculus OX=10090 GN=Khsrp PE=1 SV=2	4.1	4.0	1.04
Fructose-bisphosphate aldolase OS=Mus musculus OX=10090 GN=Aldoart2 PE=1 SV=1	4.1	4.0	1.04
High mobility group protein B1 OS=Mus musculus OX=10090 GN=Hmgb1 PE=1 SV=2	4.1	4.0	1.04
Metalloproteinase inhibitor 3 OS=Mus musculus OX=10090 GN=Timp3 PE=1 SV=1	4.1	4.0	1.04
Nucleolar RNA helicase 2 OS=Mus musculus OX=10090 GN=Ddx21 PE=1 SV=3	4.1	4.0	1.04
Predicted gene 4788 OS=Mus musculus OX=10090 GN=Gm4788 PE=1 SV=1	4.1	4.0	1.04
Solute carrier family 12 member 4 OS=Mus musculus OX=10090 GN=Slc12a4 PE=1 SV=2	4.1	4.0	1.04
tRNA (cytosine(34)-C(5))-methyltransferase OS=Mus musculus OX=10090 GN=Nsun2 PE=1 SV=2	4.1	4.0	1.04

Histone H4 OS=Mus musculus OX=10090 GN=Hist1h4a PE=1 SV=2	52.8	50.5	1.04
Pregnancy zone protein OS=Mus musculus OX=10090 GN=Pzp PE=1 SV=3	20.3	19.4	1.05
40S ribosomal protein S3 OS=Mus musculus OX=10090 GN=Rps3 PE=1 SV=1	40.6	38.6	1.05
Nucleolin OS=Mus musculus OX=10090 GN=Ncl PE=1 SV=2	24.4	23.2	1.05
Glutathione peroxidase 3 OS=Mus musculus OX=10090 GN=Gpx3 PE=1 SV=2	8.1	7.6	1.06
Plectin OS=Mus musculus OX=10090 GN=Plec PE=1 SV=3	8.3	7.8	1.06
Glyceraldehyde-3-phosphate dehydrogenase OS=Mus musculus OX=10090 GN=Gapdh PE=1 SV=2	101.0	93.8	1.08
40S ribosomal protein SA OS=Mus musculus OX=10090 GN=Rpsa PE=1 SV=4	4.1	3.8	1.08
C-1-tetrahydrofolate synthase, cytoplasmic OS=Mus musculus OX=10090 GN=Mthfd1 PE=1 SV=1	4.1	3.8	1.08
Cell growth-regulating nucleolar protein OS=Mus musculus OX=10090 GN=Lyar PE=1 SV=2	4.1	3.8	1.08
Complement component 1, s subcomponent 1 OS=Mus musculus OX=10090 GN=C1s1 PE=1 SV=1	4.1	3.8	1.08
Elongation factor 1-beta OS=Mus musculus OX=10090 GN=Eef1b PE=1 SV=5	4.1	3.8	1.08
N-alpha-acetyltransferase 50 OS=Mus musculus OX=10090 GN=Naa50 PE=1 SV=1	4.1	3.8	1.08
Peroxiredoxin-2 OS=Mus musculus OX=10090 GN=Prdx2 PE=1 SV=3	8.3	7.6	1.08
T-complex protein 1 subunit alpha OS=Mus musculus OX=10090 GN=Tcp1 PE=1 SV=3	4.1	3.8	1.08
T-complex protein 1 subunit beta OS=Mus musculus OX=10090 GN=Cct2 PE=1 SV=4	4.1	3.8	1.08
Elongin-B OS=Mus musculus OX=10090 GN=Elob PE=1 SV=1	60.4	54.7	1.11
Trypsin OS=Sus scrofa PE=1 SV=1	742.5	661.2	1.12
CD5 antigen-like OS=Mus musculus OX=10090 GN=Cd5l PE=1 SV=3	96.7	85.5	1.13
60S ribosomal protein L7 OS=Mus musculus OX=10090 GN=Rpl7 PE=1 SV=2	48.4	42.8	1.13
Mannan-binding lectin serine protease 1 OS=Mus musculus OX=10090 GN=Masp1 PE=1 SV=2	84.7	73.8	1.15
60S ribosomal protein L6 OS=Mus musculus OX=10090 GN=Rpl6 PE=1 SV=3	40.4	35.1	1.15
Fibronectin OS=Mus musculus OX=10090 GN=Fn1 PE=1 SV=1	390.3	339.3	1.15
Predicted gene 7298 OS=Mus musculus OX=10090 GN=Gm7298 PE=4 SV=1	35.8	31.2	1.15

GTP-binding nuclear protein Ran OS=Mus musculus OX=10090 GN=1700009N14Rik PE=2 SV=1	32.3	27.3	1.18
Peroxiredoxin-1 OS=Mus musculus OX=10090 GN=Prdx1 PE=1 SV=1	64.6	54.7	1.18
40S ribosomal protein S8 OS=Mus musculus OX=10090 GN=Rps8 PE=1 SV=2	28.3	23.5	1.21
60S ribosomal protein L18a OS=Mus musculus OX=10090 GN=Rpl18a PE=1 SV=1	28.3	23.5	1.21
Elongation factor 1-alpha 1 OS=Mus musculus OX=10090 GN=Eef1a1 PE=1 SV=3	84.9	70.2	1.21
60S ribosomal protein L23a OS=Mus musculus OX=10090 GN=Rpl23a PE=1 SV=1	28.3	23.4	1.21
Fascin OS=Mus musculus OX=10090 GN=Fscn1 PE=1 SV=4	28.3	23.4	1.21
60S ribosomal protein L7a OS=Mus musculus OX=10090 GN=Rpl7a PE=1 SV=2	76.4	62.5	1.22
Nucleophosmin OS=Mus musculus OX=10090 GN=Npm1 PE=1 SV=1	80.9	66.0	1.23
40S ribosomal protein S28 OS=Mus musculus OX=10090 GN=Rps28 PE=1 SV=1	24.2	19.5	1.24
60S ribosomal protein L28 OS=Mus musculus OX=10090 GN=Rpl28 PE=1 SV=2	48.4	39.1	1.24
Serum albumin OS=Mus musculus OX=10090 GN=Alb PE=1 SV=3	44.1	35.0	1.26
Eukaryotic initiation factor 4A-I OS=Mus musculus OX=10090 GN=Eif4a1 PE=1 SV=1	88.8	70.2	1.26
Probable ATP-dependent RNA helicase DDX5 OS=Mus musculus OX=10090 GN=Ddx5 PE=1 SV=2	20.1	15.7	1.28
Clusterin OS=Mus musculus OX=10090 GN=Clu PE=1 SV=1	169.7	132.4	1.28
Filamin-A OS=Mus musculus OX=10090 GN=Flna PE=1 SV=5	60.4	47.0	1.29
40S ribosomal protein S19 OS=Mus musculus OX=10090 GN=Rps19 PE=1 SV=3	20.3	15.7	1.29
60S ribosomal protein L12 OS=Mus musculus OX=10090 GN=Rpl12 PE=1 SV=2	20.1	15.6	1.29
Heterogeneous nuclear ribonucleoprotein K OS=Mus musculus OX=10090 GN=Hnrnpk PE=1 SV=1	20.1	15.6	1.29
60S ribosomal protein L17 OS=Mus musculus OX=10090 GN=Rpl17 PE=1 SV=3	20.3	15.6	1.30
Murinoglobulin-1 OS=Mus musculus OX=10090 GN=Mug1 PE=1 SV=3	100.0	74.1	1.35
60S ribosomal protein L26 OS=Mus musculus OX=10090 GN=Rpl26 PE=1 SV=1	16.1	11.8	1.37
Eukaryotic translation initiation factor 5A-2 OS=Mus musculus OX=10090 GN=Eif5a2 PE=1 SV=3	16.1	11.8	1.37
Alpha-1-antitrypsin 1-5 OS=Mus musculus OX=10090 GN=Serpina1e PE=1 SV=1	16.1	11.6	1.39

Heterogeneous nuclear ribonucleoprotein U OS=Mus musculus OX=10090 GN=Hnrnpu PE=1 SV=1	48.4	34.8	1.39
Beta-casein OS=Bos taurus GN=CSN2 PE=1 SV=2	16.3	11.6	1.41
60S ribosomal protein L27 OS=Mus musculus OX=10090 GN=Rpl27 PE=1 SV=2	44.5	31.3	1.42
Protein-L-isoaspartate O-methyltransferase domain-containing protein 2 OS=Mus musculus OX=10090 GN=Pcmtd2 PE=2 SV=1	28.3	19.4	1.46
40S ribosomal protein S4, X isoform OS=Mus musculus OX=10090 GN=Rps4x PE=1 SV=2	52.8	35.1	1.50
Heterogeneous nuclear ribonucleoprotein A3 OS=Mus musculus OX=10090 GN=Hnrnpa3 PE=1 SV=1	52.8	35.0	1.51
Protein-L-isoaspartate(D-aspartate) O-methyltransferase OS=Mus musculus OX=10090 GN=Pcmt1 PE=1 SV=3	12.2	7.9	1.54
60S ribosomal protein L36 OS=Mus musculus OX=10090 GN=Rpl36 PE=1 SV=1	24.2	15.7	1.54
Peroxiredoxin-6 OS=Mus musculus OX=10090 GN=Prdx6 PE=1 SV=3	24.2	15.7	1.54
Predicted gene 29423 OS=Mus musculus OX=10090 GN=Gm29423 PE=4 SV=1	12.0	7.8	1.54
60 kDa heat shock protein, mitochondrial OS=Mus musculus OX=10090 GN=Hspd1 PE=1 SV=1	24.2	15.6	1.55
Tubulin beta-5 chain OS=Mus musculus OX=10090 GN=Tubb5 PE=1 SV=1	36.2	23.2	1.56
Elongation factor 1-gamma OS=Mus musculus OX=10090 GN=Eef1g PE=1 SV=3	36.6	23.4	1.57
Thioredoxin OS=Mus musculus OX=10090 GN=Txn PE=1 SV=3	12.2	7.8	1.57
60S ribosomal protein L10a OS=Mus musculus OX=10090 GN=Rpl10a PE=1 SV=3	12.4	7.8	1.59
Tubulin alpha-4A chain OS=Mus musculus OX=10090 GN=Tuba4a PE=1 SV=1	44.1	27.3	1.61
60S ribosomal protein L15 OS=Mus musculus OX=10090 GN=Rpl15 PE=1 SV=4	32.5	19.5	1.66
60S ribosomal protein L13 OS=Mus musculus OX=10090 GN=Rpl13 PE=1 SV=3	20.3	11.8	1.72
Cysteine and glycine-rich protein 2 OS=Mus musculus OX=10090 GN=Csrp2 PE=1 SV=3	20.3	11.8	1.72
Prothrombin OS=Mus musculus OX=10090 GN=F2 PE=1 SV=1	20.3	11.6	1.75
Tubulin alpha-3 chain OS=Mus musculus OX=10090 GN=Tuba3a PE=1 SV=1	48.0	27.3	1.76
Alpha-enolase OS=Mus musculus OX=10090 GN=Eno1 PE=1 SV=3	28.2	15.6	1.81
60S acidic ribosomal protein P2 OS=Mus musculus OX=10090 GN=Rplp2 PE=1 SV=3	36.4	19.5	1.86
Tubulin beta-2B chain OS=Mus musculus OX=10090 GN=Tubb2b PE=1 SV=1	36.2	19.4	1.87

T-complex protein 1 subunit delta OS=Mus musculus OX=10090 GN=Cct4 PE=1 SV=3	16.0	7.9	2.01
60S ribosomal protein L29 OS=Mus musculus OX=10090 GN=Rpl29 PE=1 SV=2	8.1	4.0	2.03
Histone-lysine N-methyltransferase EZH1 OS=Mus musculus OX=10090 GN=Ezh1 PE=1 SV=1	8.1	4.0	2.03
Inter alpha-trypsin inhibitor, heavy chain 4 OS=Mus musculus OX=10090 GN=Itih4 PE=1 SV=2	8.1	4.0	2.03
Zinc finger and BTB domain-containing 12 OS=Mus musculus OX=10090 GN=Zbtb12 PE=2 SV=1	16.1	7.9	2.03
60S ribosomal protein L21 OS=Mus musculus OX=10090 GN=Rpl21 PE=1 SV=3	7.9	3.8	2.06
Myb-binding protein 1A OS=Mus musculus OX=10090 GN=Mybbp1a PE=1 SV=2	40.6	19.5	2.07
Multifunctional protein ADE2 OS=Mus musculus OX=10090 GN=Paics PE=1 SV=4	24.4	11.8	2.08
Receptor of activated protein C kinase 1 OS=Mus musculus OX=10090 GN=Rack1 PE=1 SV=3	8.3	4.0	2.08
TATA-box-binding protein-associated factor 15 (Fragment) OS=Mus musculus OX=10090 GN=Taf15 PE=1 SV=1	8.3	4.0	2.08
40S ribosomal protein S23 OS=Mus musculus OX=10090 GN=Rps23 PE=1 SV=3	16.3	7.8	2.10
Alpha-S1-casein OS=Bos taurus GN=CSN1S1 PE=1 SV=2	8.1	3.8	2.11
DnaJ homolog subfamily B member 1 OS=Mus musculus OX=10090 GN=Dnajb1 PE=1 SV=3	8.1	3.8	2.11
Lamin-B1 OS=Mus musculus OX=10090 GN=Lmnb1 PE=1 SV=3	8.1	3.8	2.11
NHP2-like protein 1 OS=Mus musculus OX=10090 GN=Snu13 PE=1 SV=4	8.1	3.8	2.11
Peptidyl-prolyl cis-trans isomerase FKBP4 OS=Mus musculus OX=10090 GN=Fkbp4 PE=1 SV=5	8.1	3.8	2.11
Serine/arginine-rich splicing factor 2 OS=Mus musculus OX=10090 GN=Srsf2 PE=1 SV=4	8.3	3.8	2.17
Complement C4-B OS=Mus musculus OX=10090 GN=C4b PE=1 SV=3	269.5	119.9	2.25
ADP/ATP translocase 2 OS=Mus musculus OX=10090 GN=Slc25a5 PE=1 SV=3	36.4	15.7	2.32
Bifunctional glutamate/proline--tRNA ligase OS=Mus musculus OX=10090 GN=Eprs PE=1 SV=4	24.2	7.8	3.11
Serum albumin OS=Bos taurus GN=ALB PE=1 SV=4	24.2	7.8	3.11
40S ribosomal protein S15 OS=Mus musculus OX=10090 GN=Rps15 PE=1 SV=2	12.0	3.8	3.15
Triosephosphate isomerase OS=Mus musculus OX=10090 GN=Tpi1 PE=1 SV=4	12.0	3.8	3.15
Heterogeneous nuclear ribonucleoprotein A1 OS=Mus musculus OX=10090 GN=Hnrnpa1 PE=1 SV=2	24.6	7.8	3.16

Lysyl endopeptidase OS=Pseudomonas aeruginosa (strain ATCC 15692 / PAO1 / 1C / PRS 101 / LMG 12228) GN=prpL PE=1 SV=1	141.7	38.9	3.64
MCG124046 OS=Mus musculus OX=10090 GN=Prss1 PE=1 SV=1	16.0	4.0	4.02
Alpha-1-antitrypsin 1-1 OS=Mus musculus OX=10090 GN=Serpina1a PE=1 SV=1	16.1	4.0	4.07
High mobility group protein B2 OS=Mus musculus OX=10090 GN=Hmgb2 PE=1 SV=3	16.1	4.0	4.07
Dihydrolipoyllysine-residue succinyltransferase component of 2-oxoglutarate dehydrogenase complex, mitochondrial OS=Mus musculus OX=10090 GN=Dlst PE=1 SV=1	20.7	3.8	5.41
Elongation factor 1-delta OS=Mus musculus OX=10090 GN=Eef1d PE=1 SV=3	24.4	4.0	6.15
[Pyruvate dehydrogenase (acetyl-transferring)] kinase isozyme 1, mitochondrial OS=Mus musculus OX=10090 GN=Pdk1 PE=1 SV=2	12.4	0	
116 kDa U5 small nuclear ribonucleoprotein component OS=Mus musculus OX=10090 GN=Eftud2 PE=1 SV=1	4.1	0	
2-oxoglutarate dehydrogenase, mitochondrial OS=Mus musculus OX=10090 GN=Ogdh PE=1 SV=3	4.1	0	
40S ribosomal protein S21 OS=Mus musculus OX=10090 GN=Rps21 PE=1 SV=1	4.1	0	
40S ribosomal protein S27-like OS=Mus musculus OX=10090 GN=Rps27l PE=1 SV=3	4.1	0	
40S ribosomal protein S29 OS=Mus musculus OX=10090 GN=Rps29 PE=1 SV=2	4.1	0	
60S ribosomal protein L29 OS=Mus musculus OX=10090 GN=Gm3550 PE=3 SV=1	8.1	0	
60S ribosomal protein L36a OS=Mus musculus OX=10090 GN=Rpl36a PE=3 SV=2	12.2	0	
60S ribosomal protein L37a OS=Mus musculus OX=10090 GN=Rpl37a PE=1 SV=2	4.1	0	
60S ribosomal protein L5 OS=Mus musculus OX=10090 GN=Rpl5 PE=1 SV=3	4.1	0	
ABI gene family member 3 OS=Mus musculus OX=10090 GN=Abi3 PE=1 SV=3	16.5	0	
Activated RNA polymerase II transcriptional coactivator p15 OS=Mus musculus OX=10090 GN=Sub1 PE=1 SV=3	4.1	0	
Acyl-coenzyme A thioesterase 13 OS=Mus musculus OX=10090 GN=Acot13 PE=1 SV=1	8.1	0	
Adenosylhomocysteinase OS=Mus musculus OX=10090 GN=Ahcy PE=1 SV=3	8.1	0	
Ankyrin-2 OS=Mus musculus OX=10090 GN=Ank2 PE=1 SV=2	12.0	0	

Apolipoprotein A-IV OS=Mus musculus OX=10090 GN=Apoa4 PE=1 SV=3	4.1	0
ATP-binding cassette sub-family E member 1 OS=Mus musculus OX=10090 GN=Abce1 PE=1 SV=1	12.4	0
ATP-citrate synthase OS=Mus musculus OX=10090 GN=Acly PE=1 SV=1	3.9	0
Bromodomain adjacent to zinc finger domain, 2B (Fragment) OS=Mus musculus OX=10090 GN=Baz2b PE=1 SV=1	3.9	0
Chromatin target of PRMT1 protein OS=Mus musculus OX=10090 GN=Chtop PE=1 SV=2	4.1	0
Chromobox protein homolog 3 OS=Mus musculus OX=10090 GN=Cbx3 PE=1 SV=2	4.1	0
Complement C1q subcomponent subunit B OS=Mus musculus OX=10090 GN=C1qb PE=1 SV=2	4.1	0
Cystatin-B OS=Mus musculus OX=10090 GN=Cstb PE=1 SV=1	16.1	0
Cysteine-rich protein 3 OS=Mus musculus OX=10090 GN=Crip3 PE=2 SV=1	3.9	0
Cytochrome c oxidase subunit 4 isoform 1, mitochondrial OS=Mus musculus OX=10090 GN=Cox4i1 PE=1 SV=2	4.1	0
Cytochrome c oxidase subunit NDUFA4 OS=Mus musculus OX=10090 GN=Ndufa4 PE=1 SV=2	3.9	0
Cytoplasmic dynein 1 heavy chain 1 OS=Mus musculus OX=10090 GN=Dync1h1 PE=1 SV=2	3.9	0
Cytoplasmic FMR1-interacting protein 2 OS=Mus musculus OX=10090 GN=Cyfip2 PE=1 SV=2	12.4	0
Cytoplasmic FMR1-interacting protein OS=Mus musculus OX=10090 GN=Cyfip1 PE=1 SV=1	12.4	0
Deoxyhypusine synthase OS=Mus musculus OX=10090 GN=Dhps PE=1 SV=2	12.0	0
DNA mismatch repair protein Msh2 OS=Mus musculus OX=10090 GN=Msh2 PE=1 SV=1	4.1	0
DnaJ heat shock protein family (Hsp40) member C13 OS=Mus musculus OX=10090 GN=Dnajc13 PE=1 SV=1	3.9	0
E3 ubiquitin-protein ligase AMFR OS=Mus musculus OX=10090 GN=Amfr PE=1 SV=2	16.5	0
FERM domain-containing protein 7 OS=Mus musculus OX=10090 GN=Frmd7 PE=1 SV=1	4.1	0
Filamin-B OS=Mus musculus OX=10090 GN=Flnb PE=1 SV=3	7.9	0
Gap junction beta-5 protein OS=Mus musculus OX=10090 GN=Gjb5 PE=2 SV=1	3.9	0
Glucose-6-phosphate 1-dehydrogenase X OS=Mus musculus OX=10090 GN=G6pdx PE=1 SV=3	8.1	0

Glycerol-3-phosphate dehydrogenase, mitochondrial OS=Mus musculus OX=10090 GN=Gpd2 PE=1 SV=2	3.9	0
Glycine dehydrogenase (decarboxylating), mitochondrial OS=Mus musculus OX=10090 GN=Gldc PE=1 SV=1	3.9	0
Golgin subfamily A member 3 OS=Mus musculus OX=10090 GN=Golga3 PE=1 SV=3	4.1	0
Heat shock 70 kDa protein 1-like OS=Mus musculus OX=10090 GN=Hspa1l PE=1 SV=4	8.3	0
Heterogeneous nuclear ribonucleoprotein F OS=Mus musculus OX=10090 GN=Hnrnpf PE=1 SV=3	12.0	0
Heterogeneous nuclear ribonucleoproteins A2/B1 OS=Mus musculus OX=10090 GN=Hnrnpa2b1 PE=1 SV=2	12.2	0
Histone H2B type 3-B OS=Mus musculus OX=10090 GN=Hist3h2bb PE=1 SV=3	12.4	0
Histone-lysine N-methyltransferase Smyd1 OS=Mus musculus OX=10090 GN=Smyd1 PE=1 SV=3	3.9	0
Inhibitor of carbonic anhydrase OS=Mus musculus OX=10090 GN=Ica PE=1 SV=1	4.1	0
Isocitrate dehydrogenase [NAD] subunit, mitochondrial OS=Mus musculus OX=10090 GN=Idh3b PE=1 SV=1	4.1	0
KH domain-containing, RNA-binding, signal transduction-associated protein 1 OS=Mus musculus OX=10090 GN=Khdrbs1 PE=1 SV=2	3.9	0
Kinesin-like protein KIF11 OS=Mus musculus OX=10090 GN=Kif11 PE=1 SV=1	12.4	0
Leucine-rich repeat-containing 10B OS=Mus musculus OX=10090 GN=Lrrc10b PE=4 SV=1	3.9	0
MARCKS-related protein OS=Mus musculus OX=10090 GN=Marcksl1 PE=1 SV=2	3.9	0
Methionine--tRNA ligase, cytoplasmic OS=Mus musculus OX=10090 GN=Mars PE=1 SV=1	4.1	0
Myotubularin-related protein 2 OS=Mus musculus OX=10090 GN=Mtmr2 PE=1 SV=3	4.1	0
Nck-associated protein 1 OS=Mus musculus OX=10090 GN=Nckap1 PE=1 SV=2	16.5	0
Nebulin OS=Mus musculus OX=10090 GN=Neb PE=1 SV=1	8.3	0
Neurofascin OS=Mus musculus OX=10090 GN=Nfasc PE=1 SV=1	3.9	0
Ogdhl protein OS=Mus musculus OX=10090 GN=Ogdhl PE=1 SV=1	4.1	0
Oxidation resistance protein 1 OS=Mus musculus OX=10090 GN=Oxr1 PE=1 SV=3	3.9	0
Pachytene checkpoint protein 2 homolog OS=Mus musculus OX=10090 GN=Trip13 PE=1 SV=1	12.4	0

Peptidyl-prolyl cis-trans isomerase OS=Mus musculus OX=10090 GN=Gm12728 PE=1 SV=1	4.1	0
Plasma serine protease inhibitor OS=Mus musculus OX=10090 GN=Serpina5 PE=1 SV=2	4.1	0
Pluripotency-associated transcript 25 OS=Mus musculus OX=10090 GN=Platr25 PE=4 SV=1	4.1	0
Polyubiquitin-B OS=Mus musculus OX=10090 GN=Ubb PE=2 SV=1	4.1	0
Pre-mRNA-splicing factor ATP-dependent RNA helicase DHX15 OS=Mus musculus OX=10090 GN=Dhx15 PE=1 SV=2	4.1	0
Protein AMBP OS=Mus musculus OX=10090 GN=Ambp PE=1 SV=2	3.9	0
Protein ATP1B4 OS=Mus musculus OX=10090 GN=Atp1b4 PE=1 SV=1	4.1	0
Protein broad-minded OS=Mus musculus OX=10090 GN=Tbc1d32 PE=1 SV=2	4.1	0
Protein FAM49B (Fragment) OS=Mus musculus OX=10090 GN=Fam49b PE=1 SV=1	4.1	0
Regulator of sex-limitation candidate 2 OS=Mus musculus OX=10090 GN=Zfp85 PE=2 SV=1	4.1	0
RNA-binding protein EWS OS=Mus musculus OX=10090 GN=Ewsr1 PE=1 SV=2	4.1	0
RNA-binding protein FUS OS=Mus musculus OX=10090 GN=Fus PE=1 SV=1	12.4	0
RuvB-like 2 OS=Mus musculus OX=10090 GN=Ruvbl2 PE=1 SV=3	4.1	0
Ryanodine receptor 3 OS=Mus musculus OX=10090 GN=Ryr3 PE=1 SV=1	16.0	0
sp|95620|STREP_Streptavidin	4.1	0
T-complex protein 1 subunit gamma OS=Mus musculus OX=10090 GN=Cct3 PE=1 SV=1	4.1	0
Terminal uridylyltransferase 7 OS=Mus musculus OX=10090 GN=Tut7 PE=1 SV=1	4.1	0
Transitional endoplasmic reticulum ATPase OS=Mus musculus OX=10090 GN=Vcp PE=1 SV=4	12.4	0
tRNA-splicing ligase RtcB homolog OS=Mus musculus OX=10090 GN=Rtcb PE=1 SV=1	4.1	0
Tubulin-specific chaperone D OS=Mus musculus OX=10090 GN=Tbcd PE=1 SV=1	3.9	0
Twinfilin-1 OS=Mus musculus OX=10090 GN=Twf1 PE=1 SV=2	24.8	0
Ubiquitin-conjugating enzyme E2 D3 OS=Mus musculus OX=10090 GN=Ube2d3 PE=1 SV=1	8.1	0
Ubiquitin-conjugating enzyme E2 N OS=Mus musculus OX=10090 GN=Ube2n PE=1 SV=1	4.1	0
Ubiquitin-like modifier-activating enzyme 1 OS=Mus musculus OX=10090 GN=Uba1 PE=1 SV=1	3.9	0
Valine--tRNA ligase OS=Mus musculus OX=10090 GN=Vars PE=1 SV=1	32.9	0

Wiskott-Aldrich syndrome protein family member 2 OS=Mus musculus OX=10090 GN=Wasf2 PE=1 SV=1	12.4	0
Zinc finger protein 106 OS=Mus musculus OX=10090 GN=Zfp106 PE=1 SV=1	4.1	0
Zinc finger protein 990 OS=Mus musculus OX=10090 GN=Zfp990 PE=1 SV=1	4.1	0
Zinc finger protein OS=Mus musculus OX=10090 GN=Gm5141 PE=4 SV=1	4.1	0
Zinc finger with KRAB and SCAN domains 16 OS=Mus musculus OX=10090 GN=Zkscan16 PE=1 SV=1	4.1	0

Meng et al., Supplementary table 3

	Antigens	Control (C)	Immunized (I)	Ratio (C/I)
	14-3-3 protein theta OS=Mus musculus OX=10090 GN=Ywhaq PE=1 SV=1	0	3.3	0
	14-3-3 protein zeta/delta OS=Mus musculus OX=10090 GN=Ywhaz PE=1 SV=1	0	3.3	0
	40S ribosomal protein SA OS=Mus musculus OX=10090 GN=Rpsa PE=1 SV=1	0	3.3	0
	Endoplasmin OS=Mus musculus OX=10090 GN=Hsp90b1 PE=1 SV=2	0	3.3	0
	Heat shock 70 kDa protein 1A OS=Mus musculus OX=10090 GN=Hspa1a PE=1 SV=2	0	3.3	0
	Heat shock 70 kDa protein 1-like OS=Mus musculus OX=10090 GN=Hspa1l PE=1 SV=4	0	3.3	0
	Heat shock-related 70 kDa protein 2 OS=Mus musculus OX=10090 GN=Hspa2 PE=1 SV=2	0	3.3	0
	Histone H1.1 OS=Mus musculus OX=10090 GN=Hist1h1a PE=1 SV=2	0	3.3	0
*	Keratin, type I cytoskeletal 14 OS=Mus musculus OX=10090 GN=Krt14 PE=1 SV=2	0	42.8	0
*	Keratin, type II cytoskeletal 6A OS=Mus musculus OX=10090 GN=Krt6a PE=1 SV=3	0	52.6	0
*	Keratin, type II cytoskeletal 73 OS=Mus musculus OX=10090 GN=Krt73 PE=1 SV=1	0	23.0	0
*	Keratin, type II cytoskeletal 75 OS=Mus musculus OX=10090 GN=Krt75 PE=1 SV=1	0	29.6	0
*	Keratin, type II cytoskeletal 79 OS=Mus musculus OX=10090 GN=Krt79 PE=1 SV=2	0	29.6	0
	MCG1050941 OS=Mus musculus OX=10090 GN=Gm5414 PE=1 SV=1	0	9.9	0
	Tubulin beta-3 chain OS=Mus musculus OX=10090 GN=Tubb3 PE=1 SV=1	0	6.6	0
	Tubulin beta-4A chain OS=Mus musculus OX=10090 GN=Tubb4a PE=1 SV=3	0	6.6	0
	3-hydroxyisobutyrate dehydrogenase, mitochondrial OS=Mus musculus OX=10090 GN=Hibadh PE=1 SV=1	0	3.3	0
	40S ribosomal protein S13 OS=Mus musculus OX=10090 GN=Rps13 PE=1 SV=2	0	3.3	0
	40S ribosomal protein S14 OS=Mus musculus OX=10090 GN=Rps14 PE=1 SV=3	0	6.6	0
	40S ribosomal protein S19 (Fragment) OS=Mus musculus OX=10090 GN=Rps19 PE=1 SV=8	0	3.3	0
	40S ribosomal protein S25 OS=Mus musculus OX=10090 GN=Rps25 PE=1 SV=1	0	3.3	0
	40S ribosomal protein S7 OS=Mus musculus OX=10090 GN=Rps7 PE=2 SV=1	0	3.3	0
	4F2 cell-surface antigen heavy chain OS=Mus musculus OX=10090 GN=Slc3a2 PE=1 SV=1	0	3.3	0

60S ribosomal protein L26 OS=Mus musculus OX=10090 GN=Rpl26 PE=1 SV=1	0	3.3	0
Aldehyde dehydrogenase family 16 member A1 OS=Mus musculus OX=10090 GN=Aldh16a1 PE=1 SV=2	0	3.3	0
Alpha-2-macroglobulin-P OS=Mus musculus OX=10090 GN=A2m PE=2 SV=2	0	6.6	0
Amine oxidase OS=Mus musculus OX=10090 GN=4930438A08Rik PE=3 SV=3	0	3.3	0
ATP-dependent RNA helicase A OS=Mus musculus OX=10090 GN=Dhx9 PE=1 SV=2	0	6.6	0
Cation-independent mannose-6-phosphate receptor OS=Mus musculus OX=10090 GN=Igf2r PE=1 SV=1	0	3.3	0
Cilia and flagella-associated protein 43 OS=Mus musculus OX=10090 GN=Cfap43 PE=1 SV=1	0	3.3	0
Cluster of 14-3-3 protein theta OS=Mus musculus OX=10090 GN=Ywhaq PE=1 SV=1 (sp|P68254|1433T_MOUSE)	0	3.3	0
Complement C1q subcomponent subunit A OS=Mus musculus OX=10090 GN=C1qa PE=1 SV=2	0	26.3	0
Complement C1q subcomponent subunit C OS=Mus musculus OX=10090 GN=C1qc PE=1 SV=2	0	9.9	0
Complement C1r-A subcomponent OS=Mus musculus OX=10090 GN=C1ra PE=1 SV=1	0	3.3	0
Complement factor H OS=Mus musculus OX=10090 GN=Cfh PE=1 SV=2	0	3.3	0
Desmoplakin OS=Mus musculus OX=10090 GN=Dsp PE=1 SV=1	0	3.3	0
Exportin-2 OS=Mus musculus OX=10090 GN=Cse1l PE=1 SV=1	0	3.3	0
Glyceraldehyde-3-phosphate dehydrogenase OS=Mus musculus OX=10090 GN=Gapdh PE=1 SV=2	0	6.6	0
Histidine-rich glycoprotein OS=Mus musculus OX=10090 GN=Hrg PE=1 SV=2	0	3.3	0
Inter alpha-trypsin inhibitor, heavy chain 4 OS=Mus musculus OX=10090 GN=Itih4 PE=1 SV=2	0	3.3	0
Inter-alpha-trypsin inhibitor heavy chain H1 OS=Mus musculus OX=10090 GN=Itih1 PE=1 SV=2	0	3.3	0
Inter-alpha-trypsin inhibitor heavy chain H3 OS=Mus musculus OX=10090 GN=Itih3 PE=1 SV=3	0	13.2	0
Laminin subunit alpha-3 OS=Mus musculus OX=10090 GN=Lama3 PE=1 SV=4	0	3.3	0
L-lactate dehydrogenase A chain OS=Mus musculus OX=10090 GN=Ldha PE=1 SV=3	0	3.3	0
MAP7 domain-containing protein 1 OS=Mus musculus OX=10090 GN=Map7d1 PE=1 SV=1	0	3.3	0
Plectin OS=Mus musculus OX=10090 GN=Plec PE=1 SV=3	0	3.3	0
Predicted gene 29423 OS=Mus musculus OX=10090 GN=Gm29423 PE=4 SV=1	0	3.3	0

	Predicted gene 8225 OS=Mus musculus OX=10090 GN=Gm8225 PE=3 SV=1	0	3.3	0
	Probable JmjC domain-containing histone demethylation protein 2C OS=Mus musculus OX=10090 GN=Jmjd1c PE=1 SV=3	0	3.3	0
	Prohibitin OS=Mus musculus OX=10090 GN=Phb PE=1 SV=1	0	3.3	0
	Prohibitin-2 OS=Mus musculus OX=10090 GN=Phb2 PE=1 SV=1	0	9.9	0
	Solute carrier family 2, facilitated glucose transporter member 3 OS=Mus musculus OX=10090 GN=Slc2a3 PE=1 SV=1	0	3.3	0
	T-complex protein 1 subunit beta OS=Mus musculus OX=10090 GN=Cct2 PE=1 SV=4	0	3.3	0
	Testis-specific gene 10 protein OS=Mus musculus OX=10090 GN=Tsga10 PE=1 SV=1	0	3.3	0
	Thrombospondin-1 OS=Mus musculus OX=10090 GN=Thbs1 PE=1 SV=1	0	3.3	0
	Tripeptidyl-peptidase 2 OS=Mus musculus OX=10090 GN=Tpp2 PE=1 SV=3	0	19.7	0
*	Keratin, type I cytoskeletal 42 OS=Mus musculus OX=10090 GN=Krt42 PE=1 SV=1	6.6	39.5	0.17
	Tubulin beta-5 chain OS=Mus musculus OX=10090 GN=Tubb5 PE=1 SV=1	3.3	16.5	0.20
	Cluster of Tubulin beta-4A chain OS=Mus musculus OX=10090 GN=Tubb4a PE=1 SV=3 (sp|Q9D6F9|TBB4A_MOUSE)	3.3	16.5	0.20
	Complement C1q subcomponent subunit B OS=Mus musculus OX=10090 GN=C1qb PE=1 SV=2	6.6	23.0	0.29
*	Keratin, type II cytoskeletal 6B OS=Mus musculus OX=10090 GN=Krt6b PE=1 SV=3	13.2	42.8	0.31
	40S ribosomal protein S3a OS=Mus musculus OX=10090 GN=Rps3a PE=1 SV=3	3.3	9.9	0.33
	Cofilin-1 OS=Mus musculus OX=10090 GN=Cfl1 PE=1 SV=3	3.3	9.9	0.33
	Elongation factor 1-alpha 1 OS=Mus musculus OX=10090 GN=Eef1a1 PE=1 SV=3	3.3	9.9	0.33
	Inter-alpha-trypsin inhibitor heavy chain H2 OS=Mus musculus OX=10090 GN=Itih2 PE=1 SV=1	3.3	9.9	0.33
	Plasminogen activator inhibitor 1 RNA-binding protein OS=Mus musculus OX=10090 GN=Serbp1 PE=1 SV=2	3.3	9.9	0.33
*	Keratin, type I cytoskeletal 16 OS=Mus musculus OX=10090 GN=Krt16 PE=1 SV=3	16.5	42.8	0.39
	Tubulin alpha-1C chain OS=Mus musculus OX=10090 GN=Tuba1c PE=1 SV=1	6.6	16.5	0.40
*	Keratin, type I cytoskeletal 17 OS=Mus musculus OX=10090 GN=Krt17 PE=1 SV=3	13.2	29.6	0.45
	Gelsolin OS=Mus musculus OX=10090 GN=Gsn PE=1 SV=3	3.3	6.6	0.50

	Heat shock cognate 71 kDa protein OS=Mus musculus OX=10090 GN=Hspa8 PE=1 SV=1	6.6	13.2	0.50
*	Keratin, type I cytoskeletal 13 OS=Mus musculus OX=10090 GN=Krt13 PE=1 SV=2	13.2	26.3	0.50
*	Keratin, type I cytoskeletal 20 OS=Mus musculus OX=10090 GN=Krt20 PE=1 SV=1	3.3	6.6	0.50
*	Keratin, type II cytoskeletal 72 OS=Mus musculus OX=10090 GN=Krt72 PE=3 SV=1	6.6	13.2	0.50
	40S ribosomal protein S18 OS=Mus musculus OX=10090 GN=Rps18 PE=3 SV=1	3.3	6.6	0.50
	40S ribosomal protein S4, X isoform OS=Mus musculus OX=10090 GN=Rps4x PE=1 SV=2	6.6	13.2	0.50
	Cluster of Gelsolin OS=Mus musculus OX=10090 GN=Gsn PE=1 SV=3 (sp|P13020|GELS_MOUSE)	3.3	6.6	0.50
	Cluster of Heat shock cognate 71 kDa protein OS=Mus musculus OX=10090 GN=Hspa8 PE=1 SV=1 (sp|P63017|HSP7C_MOUSE)	6.6	13.2	0.50
	Cytochrome P450 3A16 OS=Mus musculus OX=10090 GN=Cyp3a16 PE=2 SV=2	3.3	6.6	0.50
	Pyruvate kinase PKM OS=Mus musculus OX=10090 GN=Pkm PE=1 SV=4	3.3	6.6	0.50
*	Keratin, type II cytoskeletal 2 epidermal OS=Mus musculus OX=10090 GN=Krt2 PE=1 SV=1	23.1	42.8	0.54
*	Keratin 15, isoform CRA_a OS=Mus musculus OX=10090 GN=Krt15 PE=1 SV=1	19.8	36.2	0.55
*	Keratin, type II cytoskeletal 5 OS=Mus musculus OX=10090 GN=Krt5 PE=1 SV=1	26.4	42.8	0.62
	Beta-globin OS=Mus musculus OX=10090 GN=Hbb-bs PE=1 SV=1	16.5	26.3	0.63
	Alpha-enolase OS=Mus musculus OX=10090 GN=Eno1 PE=1 SV=1	6.6	9.9	0.67
*	Keratin, type II cytoskeletal 8 OS=Mus musculus OX=10090 GN=Krt8 PE=1 SV=4	13.2	19.7	0.67
	Cluster of Alpha-enolase OS=Mus musculus OX=10090 GN=Eno1 PE=1 SV=1 (tr|Q6PHC1|Q6PHC1_MOUSE)	6.6	9.9	0.67
	Ryanodine receptor 3 OS=Mus musculus OX=10090 GN=Ryr3 PE=1 SV=1	6.6	9.9	0.67
	Clusterin OS=Mus musculus OX=10090 GN=Clu PE=1 SV=1	36.3	52.6	0.69
	Fibronectin OS=Mus musculus OX=10090 GN=Fn1 PE=1 SV=4	29.7	42.8	0.70
*	Keratin, type II cytoskeletal 1b OS=Mus musculus OX=10090 GN=Krt77 PE=1 SV=1	23.1	32.9	0.70
	Serum albumin OS=Bos taurus GN=ALB PE=1 SV=4	23.1	32.9	0.70
	Cluster of Serum albumin OS=Bos taurus GN=ALB PE=1 SV=4 (sp|P02769|ALBU_BOVIN)	23.1	32.9	0.70
	Histone H2B type 3-B OS=Mus musculus OX=10090 GN=Hist3h2bb PE=1 SV=3	16.5	23.0	0.72

	Cluster of Actin, cytoplasmic 2 OS=Mus musculus OX=10090 GN=Actg1 PE=1 SV=1 (sp|P63260|ACTG_MOUSE)	33.0	46.1	0.72
	Major vault protein OS=Mus musculus OX=10090 GN=Mvp PE=1 SV=4	26.4	36.2	0.73
	Histone H2B type 1-F/J/L OS=Mus musculus OX=10090 GN=Hist1h2bf PE=1 SV=2	19.8	26.3	0.75
	Cluster of Histone H2B type 3-B OS=Mus musculus OX=10090 GN=Hist3h2bb PE=1 SV=3 (sp|Q8CGP0|H2B3B_MOUSE)	19.8	26.3	0.75
*	Keratin 78 OS=Mus musculus OX=10090 GN=Krt78 PE=1 SV=1	9.9	13.2	0.75
	Actin, cytoplasmic 2 OS=Mus musculus OX=10090 GN=Actg1 PE=1 SV=1	33.0	42.8	0.77
	Clathrin heavy chain 1 OS=Mus musculus OX=10090 GN=Cltc PE=1 SV=3	26.4	32.9	0.80
*	Keratin, type II cytoskeletal 71 OS=Mus musculus OX=10090 GN=Krt71 PE=1 SV=1	16.5	19.7	0.84
	Histone H4 OS=Mus musculus OX=10090 GN=Hist1h4a PE=1 SV=2	26.4	29.6	0.89
	Actin, alpha cardiac muscle 1 OS=Mus musculus OX=10090 GN=Actc1 PE=1 SV=1	29.7	32.9	0.90
	Mannose-binding protein C OS=Mus musculus OX=10090 GN=Mbl2 PE=1 SV=2	36.3	39.5	0.92
	CD5 antigen-like OS=Mus musculus OX=10090 GN=Cd5l PE=1 SV=3	59.5	62.5	0.95
	Heat shock protein HSP 90-beta OS=Mus musculus OX=10090 GN=Hsp90ab1 PE=1 SV=3	3.3	3.3	1
	Histone H1.3 OS=Mus musculus OX=10090 GN=Hist1h1d PE=1 SV=2	3.3	3.3	1
	Murinoglobulin-1 OS=Mus musculus OX=10090 GN=Mug1 PE=1 SV=3	19.8	19.7	1
	Predicted gene 7298 OS=Mus musculus OX=10090 GN=Gm7298 PE=4 SV=1	9.9	9.9	1
	Tubulin beta-1 chain OS=Mus musculus OX=10090 GN=Tubb1 PE=1 SV=1	3.3	3.3	1
	40S ribosomal protein S16 OS=Mus musculus OX=10090 GN=Rps16 PE=1 SV=4	6.6	6.6	1
	40S ribosomal protein S20 OS=Mus musculus OX=10090 GN=Rps20 PE=1 SV=1	9.9	9.9	1
	40S ribosomal protein S28 OS=Mus musculus OX=10090 GN=Rps28 PE=1 SV=1	6.6	6.6	1
	40S ribosomal protein S30 OS=Mus musculus OX=10090 GN=Fau PE=1 SV=1	6.6	6.6	1
	60S ribosomal protein L11 OS=Mus musculus OX=10090 GN=Rpl11 PE=1 SV=4	3.3	3.3	1
	60S ribosomal protein L35 OS=Mus musculus OX=10090 GN=Rpl35 PE=1 SV=1	3.3	3.3	1
	Ankyrin-2 OS=Mus musculus OX=10090 GN=Ank2 PE=1 SV=2	3.3	3.3	1

	ATP-sensitive inward rectifier potassium channel 10 OS=Mus musculus OX=10090 GN=Kcnj10 PE=1 SV=1	3.3	3.3	1
	Cluster of Heat shock protein HSP 90-beta OS=Mus musculus OX=10090 GN=Hsp90ab1 PE=1 SV=3 (sp|P11499|HS90B_MOUSE)	3.3	3.3	1
	Cluster of Histone H1.3 OS=Mus musculus OX=10090 GN=Hist1h1d PE=1 SV=2 (sp|P43277|H13_MOUSE)	3.3	3.3	1
	Cluster of Murinoglobulin-1 OS=Mus musculus OX=10090 GN=Mug1 PE=1 SV=3 (sp|P28665|MUG1_MOUSE)	19.8	19.7	1
	Cytochrome b-c1 complex subunit Rieske, mitochondrial OS=Mus musculus OX=10090 GN=Uqcrfs1 PE=1 SV=1	3.3	3.3	1
	Epithelial splicing regulatory protein 2 OS=Mus musculus OX=10090 GN=Esrp2 PE=1 SV=1	3.3	3.3	1
	GTP-binding nuclear protein Ran OS=Mus musculus OX=10090 GN=Ran PE=1 SV=3	3.3	3.3	1
	Hemoglobin subunit alpha OS=Mus musculus OX=10090 GN=Hba PE=1 SV=2	16.5	16.5	1
	Histone H2A type 1-B OS=Mus musculus OX=10090 GN=Hist1h2ab PE=1 SV=1	6.6	6.6	1
	Histone H3.3C OS=Mus musculus OX=10090 GN=H3f3c PE=3 SV=3	3.3	3.3	1
	Importin subunit alpha-3 OS=Mus musculus OX=10090 GN=Kpna4 PE=1 SV=1	3.3	3.3	1
*	Keratin, type II cytoskeletal 2 oral OS=Mus musculus OX=10090 GN=Krt76 PE=1 SV=1	3.3	3.3	1
	Mannan-binding lectin serine protease 1 OS=Mus musculus OX=10090 GN=Masp1 PE=1 SV=2	33.0	32.9	1
	Mannan-binding lectin serine protease 2 OS=Mus musculus OX=10090 GN=Masp2 PE=1 SV=1	33.0	32.9	1
	Oxidation resistance protein 1 OS=Mus musculus OX=10090 GN=Oxr1 PE=1 SV=1	3.3	3.3	1
	Rho GTPase-activating protein 23 OS=Mus musculus OX=10090 GN=Arhgap23 PE=1 SV=2	3.3	3.3	1
	Serum albumin OS=Mus musculus OX=10090 GN=Alb PE=1 SV=3	9.9	9.9	1
	Transcription regulator protein BACH1 OS=Mus musculus OX=10090 GN=Bach1 PE=1 SV=1	3.3	3.3	1
	Transforming protein RhoA OS=Mus musculus OX=10090 GN=Rhoa PE=1 SV=1	3.3	3.3	1
	Ubiquitin carboxyl-terminal hydrolase 12 OS=Mus musculus OX=10090 GN=Usp12 PE=2 SV=2	3.3	3.3	1
	Zinc finger CCCH type-containing 7B OS=Mus musculus OX=10090 GN=Zc3h7b PE=1 SV=1	3.3	3.3	1
	Complement C3 OS=Mus musculus OX=10090 GN=C3 PE=1 SV=3	1212.4	1178.0	1.03
	Cationic trypsin OS=Bos taurus PE=1 SV=3	79.3	69.1	1.15
	Murinoglobulin-2 OS=Mus musculus OX=10090 GN=Mug2 PE=1 SV=2	13.2	9.9	1.34
	Beta-actin-like protein 2 OS=Mus musculus OX=10090 GN=Actbl2 PE=1 SV=1	23.1	16.5	1.41

	Stress-70 protein, mitochondrial OS=Mus musculus OX=10090 GN=Hspa9 PE=1 SV=3	9.9	6.6	1.51
	Complement C4-B OS=Mus musculus OX=10090 GN=C4b PE=1 SV=3	145.4	79.0	1.84
	40S ribosomal protein SA OS=Mus musculus OX=10090 GN=Rpsa PE=1 SV=4	6.6	3.3	2.01
	ATP synthase subunit alpha, mitochondrial OS=Mus musculus OX=10090 GN=Atp5a1 PE=1 SV=1	6.6	3.3	2.01
	Cluster of 40S ribosomal protein SA OS=Mus musculus OX=10090 GN=Rpsa PE=1 SV=1 (tr|A0A1L1SUK3|A0A1L1SUK3_MOUSE)	6.6	3.3	2.01
	Destrin OS=Mus musculus OX=10090 GN=Dstn PE=1 SV=3	6.6	3.3	2.01
	MCG15081 OS=Mus musculus OX=10090 GN=Gm10334 PE=3 SV=1	6.6	3.3	2.01
	Tudor domain-containing protein 5 OS=Mus musculus OX=10090 GN=Tdrd5 PE=1 SV=3	9.9	3.3	3.01
	ADP/ATP translocase 2 OS=Mus musculus OX=10090 GN=Slc25a5 PE=1 SV=3	3.3	0	
	Basement membrane-specific heparan sulfate proteoglycan core protein OS=Mus musculus OX=10090 GN=Hspg2 PE=1 SV=1	3.3	0	
	Basement membrane-specific heparan sulfate proteoglycan core protein OS=Mus musculus OX=10090 GN=Hspg2 PE=1 SV=1	3.3	0	
*	Keratin, type I cytoskeletal 27 OS=Mus musculus OX=10090 GN=Krt27 PE=1 SV=1	13.2	0	
*	Keratin, type II cytoskeletal 1 OS=Mus musculus OX=10090 GN=Krt1 PE=1 SV=4	29.7	0	
	40S ribosomal protein S12 OS=Mus musculus OX=10090 GN=Rps12 PE=1 SV=2	3.3	0	
	40S ribosomal protein S3 OS=Mus musculus OX=10090 GN=Rps3 PE=1 SV=1	3.3	0	
	60S ribosomal protein L29 OS=Mus musculus OX=10090 GN=Gm5218 PE=3 SV=1	3.3	0	
	AF4/FMR2 family member 1 OS=Mus musculus OX=10090 GN=Aff1 PE=1 SV=2	3.3	0	
	ATM interactor OS=Mus musculus OX=10090 GN=Atmin PE=2 SV=2	3.3	0	
	Cluster of ADP/ATP translocase 2 OS=Mus musculus OX=10090 GN=Slc25a5 PE=1 SV=3 (sp|P51881|ADT2_MOUSE)	3.3	0	
	Cluster of Basement membrane-specific heparan sulfate proteoglycan core protein OS=Mus musculus OX=10090 GN=Hspg2 PE=1 SV=1 (sp|Q05793|PGBM_MOUSE)	3.3	0	
	Dihydrolipoyllysine-residue acetyltransferase component of pyruvate dehydrogenase complex, mitochondrial OS=Mus musculus OX=10090 GN=Dlat PE=1 SV=2	6.6	0	
	Endoplasmic reticulum chaperone BiP OS=Mus musculus OX=10090 GN=Hspa5 PE=1 SV=3	3.3	0	

Leucine-rich repeat-containing protein 7 (Fragment) OS=Mus musculus OX=10090 GN=Lrrc7 PE=1 SV=1	6.6	0
Maestro heat-like repeat family member 6 OS=Mus musculus OX=10090 GN=Mroh6 PE=4 SV=1	3.3	0
M-phase phosphoprotein 8 OS=Mus musculus OX=10090 GN=Mphosph8 PE=1 SV=1	3.3	0
Na(+)/H(+) exchange regulatory cofactor NHE-RF1 OS=Mus musculus OX=10090 GN=Slc9a3r1 PE=1 SV=3	3.3	0
Neutrophil collagenase OS=Mus musculus OX=10090 GN=Mmp8 PE=2 SV=2	3.3	0
Pre-mRNA-processing factor 19 OS=Mus musculus OX=10090 GN=Prpf19 PE=1 SV=1	6.6	0
Properdin OS=Mus musculus OX=10090 GN=Cfp PE=2 SV=2	3.3	0
Protein FAM208B OS=Mus musculus OX=10090 GN=Fam208b PE=1 SV=2	3.3	0
Receptor-interacting serine/threonine-protein kinase 4 OS=Mus musculus OX=10090 GN=Ripk4 PE=4 SV=1	3.3	0
RIKEN cDNA D830044I16 gene OS=Mus musculus OX=10090 GN=D830044I16Rik PE=4 SV=1	3.3	0
Single-pass membrane and coiled-coil domain-containing protein 3 OS=Mus musculus OX=10090 GN=Smco3 PE=2 SV=1	3.3	0
Stomatin-like protein 2, mitochondrial OS=Mus musculus OX=10090 GN=Stoml2 PE=1 SV=1	3.3	0
Tyrosine-protein kinase BAZ1B OS=Mus musculus OX=10090 GN=Baz1b PE=1 SV=2	3.3	0

Meng et al., Supplementary table 4

	Antigens	Ratio(Control/Immunized) Cell-based	Exosome-based
	14-3-3 protein theta OS=Mus musculus OX=10090 GN=Ywhaq PE=1 SV=1	0	0
	14-3-3 protein zeta/delta OS=Mus musculus OX=10090 GN=Ywhaz PE=1 SV=1	0	0
	40S ribosomal protein S7 OS=Mus musculus OX=10090 GN=Rps7 PE=2 SV=1	0	0
	Complement factor H OS=Mus musculus OX=10090 GN=Cfh PE=1 SV=2	0	0
	Desmoplakin OS=Mus musculus OX=10090 GN=Dsp PE=1 SV=1	0	0
	Inter-alpha-trypsin inhibitor heavy chain H1 OS=Mus musculus OX=10090 GN=Itih1 PE=1 SV=2	0	0
*	Keratin, type II cytoskeletal 72 OS=Mus musculus OX=10090 GN=Krt72 PE=3 SV=1	0	0.50
*	Keratin, type II cytoskeletal 73 OS=Mus musculus OX=10090 GN=Krt73 PE=1 SV=1	0.29	0
*	Keratin 78 OS=Mus musculus OX=10090 GN=Krt78 PE=1 SV=1	0.34	0.75
*	Keratin, type II cytoskeletal 79 OS=Mus musculus OX=10090 GN=Krt79 PE=1 SV=2	0.41	0
*	Keratin, type II cytoskeletal 1b OS=Mus musculus OX=10090 GN=Krt77 PE=1 SV=1	0.46	0.70
*	Keratin, type II cytoskeletal 6A OS=Mus musculus OX=10090 GN=Krt6a PE=1 SV=3	0.49	0
	Thrombospondin-1 OS=Mus musculus OX=10090 GN=Thbs1 PE=1 SV=1	0.53	0
*	Keratin, type II cytoskeletal 5 OS=Mus musculus OX=10090 GN=Krt5 PE=1 SV=1	0.53	0.62
*	Keratin 15, isoform CRA_a OS=Mus musculus OX=10090 GN=Krt15 PE=1 SV=1	0.54	0.55
*	Keratin, type II cytoskeletal 2 epidermal OS=Mus musculus OX=10090 GN=Krt2 PE=1 SV=1	0.57	0.54
*	Keratin, type I cytoskeletal 16 OS=Mus musculus OX=10090 GN=Krt16 PE=1 SV=3	0.58	0.39
*	Keratin, type II cytoskeletal 8 OS=Mus musculus OX=10090 GN=Krt8 PE=1 SV=4	0.67	0.67
	40S ribosomal protein S3a OS=Mus musculus OX=10090 GN=Rps3a PE=1 SV=3	0.69	0.33
	40S ribosomal protein S25 OS=Mus musculus OX=10090 GN=Rps25 PE=1 SV=1	0.70	0
	Beta-globin OS=Mus musculus OX=10090 GN=Hbb-bs PE=1 SV=1	0.74	0.63
	Gelsolin OS=Mus musculus OX=10090 GN=Gsn PE=1 SV=3	0.83	0.50

	Plasminogen activator inhibitor 1 RNA-binding protein OS=Mus musculus OX=10090 GN=Serbp1 PE=1 SV=2	0.83	0.33
	40S ribosomal protein S18 OS=Mus musculus OX=10090 GN=Rps18 PE=3 SV=1	0.83	0.50
*	Keratin, type I cytoskeletal 17 OS=Mus musculus OX=10090 GN=Krt17 PE=1 SV=3	0.85	0.45
	40S ribosomal protein S14 OS=Mus musculus OX=10090 GN=Rps14 PE=1 SV=3	0.89	0
	Actin, cytoplasmic 2 OS=Mus musculus OX=10090 GN=Actg1 PE=1 SV=1	0.90	0.77
*	Keratin, type I cytoskeletal 42 OS=Mus musculus OX=10090 GN=Krt42 PE=1 SV=1	0.91	0.17
	Mannose-binding protein C OS=Mus musculus OX=10090 GN=Mbl2 PE=1 SV=2	0.91	0.92
	Pyruvate kinase PKM OS=Mus musculus OX=10090 GN=Pkm PE=1 SV=4	0.91	0.50
	Heat shock cognate 71 kDa protein OS=Mus musculus OX=10090 GN=Hspa8 PE=1 SV=1	0.91	0.50

Identified proteins (2698)	Molecular weight	Protein abundance
(E3-independent) E2 ubiquitin-conjugating enzyme UBE2O OS=Mus musculus OX=10090 GN=Ube2o PE=1 SV=3	141 kDa	4
0610038D11Rik protein OS=Mus musculus OX=10090 GN=Trmt112 PE=1 SV=1	12 kDa	1
1,2-dihydroxy-3-keto-5-methylthiopentene dioxygenase OS=Mus musculus OX=10090 GN=Adi1 PE=1 SV=1	22 kDa	5
1,5-anhydro-D-fructose reductase OS=Mus musculus OX=10090 GN=Akr1e2 PE=1 SV=1	34 kDa	3
10 kDa heat shock protein, mitochondrial OS=Mus musculus OX=10090 GN=Hspe1 PE=1 SV=2	11 kDa	53
116 kDa U5 small nuclear ribonucleoprotein component OS=Mus musculus OX=10090 GN=Eftud2 PE=1 SV=1	109 kDa	37
130kDa Protein 4.1B MEF cell isoform OS=Mus musculus OX=10090 GN=Epb41l3 PE=1 SV=1	98 kDa	2
14 kDa phosphohistidine phosphatase OS=Mus musculus OX=10090 GN=Phpt1 PE=1 SV=1	14 kDa	6
14-3-3 protein beta/alpha OS=Mus musculus OX=10090 GN=Ywhab PE=1 SV=3	28 kDa	51
14-3-3 protein epsilon OS=Mus musculus OX=10090 GN=Ywhae PE=1 SV=1	29 kDa	109
14-3-3 protein eta OS=Mus musculus OX=10090 GN=Ywhah PE=1 SV=2	28 kDa	34
14-3-3 protein gamma OS=Mus musculus OX=10090 GN=Ywhag PE=1 SV=2	28 kDa	47
14-3-3 protein theta (Fragment) OS=Mus musculus OX=10090 GN=Ywhaq PE=1 SV=1	34 kDa	62
14-3-3 protein theta OS=Mus musculus OX=10090 GN=Ywhaq PE=1 SV=1	28 kDa	66
14-3-3 protein zeta/delta OS=Mus musculus OX=10090 GN=Ywhaz PE=1 SV=1	28 kDa	87
182 kDa tankyrase-1-binding protein OS=Mus musculus OX=10090 GN=Tnks1bp1 PE=1 SV=2	182 kDa	18
1-phosphatidylinositol 4,5-bisphosphate phosphodiesterase beta-3 OS=Mus musculus OX=10090 GN=Plcb3 PE=1 SV=2	139 kDa	2
2,4-dienoyl-CoA reductase, mitochondrial OS=Mus musculus OX=10090 GN=Decr1 PE=1 SV=1	36 kDa	5
26S proteasome non-ATPase regulatory subunit 1 OS=Mus musculus OX=10090 GN=Psmd1 PE=1 SV=1	106 kDa	41
26S proteasome non-ATPase regulatory subunit 11 OS=Mus musculus OX=10090 GN=Psmd11 PE=1 SV=3	47 kDa	23
26S proteasome non-ATPase regulatory subunit 12 OS=Mus musculus OX=10090 GN=Psmd12 PE=1 SV=4	53 kDa	17
26S proteasome non-ATPase regulatory subunit 13 OS=Mus musculus OX=10090 GN=Psmd13 PE=1 SV=1	43 kDa	9
26S proteasome non-ATPase regulatory subunit 14 OS=Mus musculus OX=10090 GN=Psmd14 PE=1 SV=2	35 kDa	11

26S proteasome non-ATPase regulatory subunit 2 OS=Mus musculus OX=10090 GN=Psmd2 PE=1 SV=1	100 kDa	44
26S proteasome non-ATPase regulatory subunit 3 OS=Mus musculus OX=10090 GN=Psmd3 PE=1 SV=3	61 kDa	21
26S proteasome non-ATPase regulatory subunit 4 (Fragment) OS=Mus musculus OX=10090 GN=Psmd4 PE=1 SV=1	21 kDa	6
26S proteasome non-ATPase regulatory subunit 4 OS=Mus musculus OX=10090 GN=Psmd4 PE=1 SV=1	41 kDa	21
26S proteasome non-ATPase regulatory subunit 5 OS=Mus musculus OX=10090 GN=Psmd5 PE=1 SV=4	56 kDa	6
26S proteasome non-ATPase regulatory subunit 6 OS=Mus musculus OX=10090 GN=Psmd6 PE=1 SV=1	46 kDa	16
26S proteasome non-ATPase regulatory subunit 7 OS=Mus musculus OX=10090 GN=Psmd7 PE=1 SV=2	37 kDa	12
26S proteasome non-ATPase regulatory subunit 8 OS=Mus musculus OX=10090 GN=Psmd8 PE=1 SV=2	40 kDa	4
26S proteasome non-ATPase regulatory subunit 9 OS=Mus musculus OX=10090 GN=Psmd9 PE=1 SV=1	25 kDa	13
26S proteasome regulatory subunit 10B OS=Mus musculus OX=10090 GN=Psmc6 PE=1 SV=1	44 kDa	22
26S proteasome regulatory subunit 4 OS=Mus musculus OX=10090 GN=Psmc1 PE=1 SV=1	49 kDa	24
26S proteasome regulatory subunit 6A (Fragment) OS=Mus musculus OX=10090 GN=Psmc3 PE=1 SV=6	34 kDa	12
26S proteasome regulatory subunit 6A OS=Mus musculus OX=10090 GN=Psmc3 PE=1 SV=1	50 kDa	21
26S proteasome regulatory subunit 6A OS=Mus musculus OX=10090 GN=Psmc3 PE=1 SV=2	50 kDa	21
26S proteasome regulatory subunit 6B OS=Mus musculus OX=10090 GN=Psmc4 PE=1 SV=1	44 kDa	20
26S proteasome regulatory subunit 7 OS=Mus musculus OX=10090 GN=Psmc2 PE=1 SV=5	49 kDa	25
26S proteasome regulatory subunit 8 OS=Mus musculus OX=10090 GN=Psmc5 PE=1 SV=1	46 kDa	26
28 kDa heat- and acid-stable phosphoprotein OS=Mus musculus OX=10090 GN=Pdap1 PE=1 SV=1	21 kDa	21
28S ribosomal protein S18b, mitochondrial (Fragment) OS=Mus musculus OX=10090 GN=Mrps18b PE=1 SV=1	22 kDa	1
28S ribosomal protein S22, mitochondrial OS=Mus musculus OX=10090 GN=Mrps22 PE=1 SV=1	41 kDa	1
28S ribosomal protein S23, mitochondrial (Fragment) OS=Mus musculus OX=10090 GN=Mrps23 PE=1 SV=1	13 kDa	1
28S ribosomal protein S24, mitochondrial OS=Mus musculus OX=10090 GN=Mrps24 PE=1 SV=1	19 kDa	1
28S ribosomal protein S26, mitochondrial OS=Mus musculus OX=10090 GN=Mrps26 PE=1 SV=1	23 kDa	1
28S ribosomal protein S31, mitochondrial OS=Mus musculus OX=10090 GN=Mrps31 PE=1 SV=1	44 kDa	1

28S ribosomal protein S36, mitochondrial OS=Mus musculus OX=10090 GN=Mrps36 PE=1 SV=1	11 kDa	5
28S ribosomal protein S5, mitochondrial OS=Mus musculus OX=10090 GN=Mrps5 PE=1 SV=1	48 kDa	2
28S ribosomal protein S6, mitochondrial OS=Mus musculus OX=10090 GN=Mrps6 PE=1 SV=3	14 kDa	1
28S ribosomal protein S9, mitochondrial (Fragment) OS=Mus musculus OX=10090 GN=Mrps9 PE=1 SV=1	15 kDa	1
2-amino-3-ketobutyrate coenzyme A ligase, mitochondrial OS=Mus musculus OX=10090 GN=Gcat PE=1 SV=1	41 kDa	2
2-aminoethanethiol dioxygenase OS=Mus musculus OX=10090 GN=Ado PE=1 SV=2	28 kDa	3
2'-deoxynucleoside 5'-phosphate N-hydrolase 1 OS=Mus musculus OX=10090 GN=Dnph1 PE=1 SV=2	19 kDa	4
2-iminobutanoate/2-iminopropanoate deaminase OS=Mus musculus OX=10090 GN=Rida PE=1 SV=3	14 kDa	4
2-oxoglutarate dehydrogenase, mitochondrial OS=Mus musculus OX=10090 GN=Ogdh PE=1 SV=3	116 kDa	3
3'(2'),5'-bisphosphate nucleotidase 1 OS=Mus musculus OX=10090 GN=Bpnt1 PE=1 SV=1	35 kDa	9
39S ribosomal protein L12, mitochondrial OS=Mus musculus OX=10090 GN=Mrpl12 PE=1 SV=2	22 kDa	2
39S ribosomal protein L28, mitochondrial OS=Mus musculus OX=10090 GN=Mrpl28 PE=1 SV=3	30 kDa	1
39S ribosomal protein L4, mitochondrial (Fragment) OS=Mus musculus OX=10090 GN=Mrpl4 PE=1 SV=1	25 kDa	1
39S ribosomal protein L46, mitochondrial OS=Mus musculus OX=10090 GN=Mrpl46 PE=1 SV=1	32 kDa	1
39S ribosomal protein L49, mitochondrial OS=Mus musculus OX=10090 GN=Mrpl49 PE=1 SV=1	19 kDa	1
39S ribosomal protein L50, mitochondrial OS=Mus musculus OX=10090 GN=Mrpl50 PE=1 SV=2	18 kDa	2
3-hydroxyacyl-CoA dehydrogenase type-2 OS=Mus musculus OX=10090 GN=Hsd17b10 PE=1 SV=1	28 kDa	29
3-hydroxyisobutyrate dehydrogenase, mitochondrial OS=Mus musculus OX=10090 GN=Hibadh PE=1 SV=1	35 kDa	11
3-ketoacyl-CoA thiolase A, peroxisomal OS=Mus musculus OX=10090 GN=Acaa1a PE=1 SV=1	44 kDa	5
3-ketoacyl-CoA thiolase B, peroxisomal OS=Mus musculus OX=10090 GN=Acaa1b PE=1 SV=1	44 kDa	3
3-ketoacyl-CoA thiolase, mitochondrial OS=Mus musculus OX=10090 GN=Acaa2 PE=1 SV=3	42 kDa	10
40S ribosomal protein S10 OS=Mus musculus OX=10090 GN=Rps10 PE=1 SV=1	19 kDa	18
40S ribosomal protein S11 (Fragment) OS=Mus musculus OX=10090 GN=Rps11 PE=1 SV=1	16 kDa	12
40S ribosomal protein S11 OS=Mus musculus OX=10090 GN=Rps11 PE=1 SV=3	18 kDa	18
40S ribosomal protein S12 OS=Mus musculus OX=10090 GN=Rps12 PE=1 SV=1	15 kDa	39

40S ribosomal protein S13 OS=Mus musculus OX=10090 GN=Rps13 PE=1 SV=2	17 kDa	15
40S ribosomal protein S14 OS=Mus musculus OX=10090 GN=Rps14 PE=1 SV=3	16 kDa	22
40S ribosomal protein S15 OS=Mus musculus OX=10090 GN=Rps15 PE=1 SV=1	14 kDa	11
40S ribosomal protein S15a (Fragment) OS=Mus musculus OX=10090 GN=Rps15a PE=1 SV=1	12 kDa	9
40S ribosomal protein S16 OS=Mus musculus OX=10090 GN=Rps16 PE=1 SV=4	16 kDa	19
40S ribosomal protein S17 OS=Mus musculus OX=10090 GN=Rps17 PE=1 SV=2	16 kDa	19
40S ribosomal protein S18 OS=Mus musculus OX=10090 GN=Rps18 PE=1 SV=3	18 kDa	30
40S ribosomal protein S19 OS=Mus musculus OX=10090 GN=Rps19 PE=1 SV=3	16 kDa	28
40S ribosomal protein S2 OS=Mus musculus OX=10090 GN=Rps2 PE=1 SV=3	31 kDa	36
40S ribosomal protein S20 OS=Mus musculus OX=10090 GN=Rps20 PE=1 SV=1	13 kDa	13
40S ribosomal protein S21 OS=Mus musculus OX=10090 GN=Rps21 PE=1 SV=1	9 kDa	37
40S ribosomal protein S23 OS=Mus musculus OX=10090 GN=Rps23 PE=1 SV=3	16 kDa	13
40S ribosomal protein S24 OS=Mus musculus OX=10090 GN=Rps24 PE=1 SV=1	15 kDa	10
40S ribosomal protein S25 OS=Mus musculus OX=10090 GN=Rps25 PE=1 SV=1	10 kDa	13
40S ribosomal protein S26 OS=Mus musculus OX=10090 GN=Rps26 PE=1 SV=3	13 kDa	9
40S ribosomal protein S27 (Fragment) OS=Mus musculus OX=10090 GN=Rps27 PE=1 SV=1	9 kDa	9
40S ribosomal protein S28 OS=Mus musculus OX=10090 GN=Rps28 PE=1 SV=1	8 kDa	17
40S ribosomal protein S29 OS=Mus musculus OX=10090 GN=Rps29 PE=1 SV=2	7 kDa	2
40S ribosomal protein S3 OS=Mus musculus OX=10090 GN=Rps3 PE=1 SV=1	27 kDa	50
40S ribosomal protein S30 OS=Mus musculus OX=10090 GN=Fau PE=1 SV=1	14 kDa	11
40S ribosomal protein S3a OS=Mus musculus OX=10090 GN=Rps3a PE=1 SV=3	30 kDa	50
40S ribosomal protein S4, X isoform OS=Mus musculus OX=10090 GN=Rps4x PE=1 SV=2	30 kDa	37
40S ribosomal protein S5 OS=Mus musculus OX=10090 GN=Rps5 PE=1 SV=1	23 kDa	29
40S ribosomal protein S6 OS=Mus musculus OX=10090 GN=Rps6 PE=1 SV=1	29 kDa	31
40S ribosomal protein S7 OS=Mus musculus OX=10090 GN=Rps7 PE=2 SV=1	22 kDa	29

40S ribosomal protein S8 OS=Mus musculus OX=10090 GN=Rps8 PE=1 SV=2	24 kDa	30
40S ribosomal protein S9 OS=Mus musculus OX=10090 GN=Rps9 PE=1 SV=3	23 kDa	18
40S ribosomal protein SA OS=Mus musculus OX=10090 GN=Rpsa PE=1 SV=4	33 kDa	27
4F2 cell-surface antigen heavy chain OS=Mus musculus OX=10090 GN=Slc3a2 PE=1 SV=1	58 kDa	24
4-trimethylaminobutyraldehyde dehydrogenase OS=Mus musculus OX=10090 GN=Aldh9a1 PE=1 SV=1	54 kDa	20
5'(3')-deoxyribonucleotidase, cytosolic type OS=Mus musculus OX=10090 GN=Nt5c PE=1 SV=1	23 kDa	9
5'-3' exoribonuclease 2 OS=Mus musculus OX=10090 GN=Xrn2 PE=1 SV=1	109 kDa	15
55 kDa erythrocyte membrane protein OS=Mus musculus OX=10090 GN=Mpp1 PE=1 SV=1	51 kDa	1
60 kDa heat shock protein, mitochondrial OS=Mus musculus OX=10090 GN=Hspd1 PE=1 SV=1	61 kDa	165
60 kDa SS-A/Ro ribonucleoprotein OS=Mus musculus OX=10090 GN=Trove2 PE=1 SV=1	60 kDa	6
60S acidic ribosomal protein P0 OS=Mus musculus OX=10090 GN=Rplp0 PE=1 SV=3	34 kDa	23
60S acidic ribosomal protein P1 OS=Mus musculus OX=10090 GN=Rplp1 PE=1 SV=1	11 kDa	3
60S acidic ribosomal protein P2 OS=Mus musculus OX=10090 GN=Rplp2 PE=1 SV=3	12 kDa	11
60S ribosomal protein L10 (Fragment) OS=Mus musculus OX=10090 GN=Rpl10 PE=1 SV=1	23 kDa	26
60S ribosomal protein L12 OS=Mus musculus OX=10090 GN=Rpl12 PE=1 SV=2	18 kDa	23
60S ribosomal protein L13 OS=Mus musculus OX=10090 GN=Rpl13 PE=1 SV=3	24 kDa	24
60S ribosomal protein L13a OS=Mus musculus OX=10090 GN=Rpl13a PE=1 SV=4	23 kDa	15
60S ribosomal protein L14 OS=Mus musculus OX=10090 GN=Rpl14 PE=1 SV=3	24 kDa	18
60S ribosomal protein L15 OS=Mus musculus OX=10090 GN=Rpl15 PE=2 SV=4	24 kDa	13
60S ribosomal protein L17 OS=Mus musculus OX=10090 GN=Rpl17 PE=1 SV=1	21 kDa	18
60S ribosomal protein L18 OS=Mus musculus OX=10090 GN=Rpl18 PE=1 SV=3	22 kDa	21
60S ribosomal protein L18a (Fragment) OS=Mus musculus OX=10090 GN=Rpl18a PE=1 SV=1	25 kDa	16
60S ribosomal protein L21 OS=Mus musculus OX=10090 GN=Rpl21 PE=1 SV=1	19 kDa	12
60S ribosomal protein L22 OS=Mus musculus OX=10090 GN=Rpl22 PE=1 SV=2	15 kDa	13
60S ribosomal protein L23 OS=Mus musculus OX=10090 GN=Rpl23 PE=1 SV=1	15 kDa	19

60S ribosomal protein L23a OS=Mus musculus OX=10090 GN=Rpl23a PE=1 SV=1	18 kDa	19
60S ribosomal protein L24 OS=Mus musculus OX=10090 GN=Rpl24 PE=1 SV=2	18 kDa	11
60S ribosomal protein L26 OS=Mus musculus OX=10090 GN=Rpl26 PE=1 SV=1	17 kDa	19
60S ribosomal protein L27 OS=Mus musculus OX=10090 GN=Rpl27 PE=1 SV=2	16 kDa	19
60S ribosomal protein L27a OS=Mus musculus OX=10090 GN=Rpl27a PE=1 SV=5	17 kDa	14
60S ribosomal protein L28 OS=Mus musculus OX=10090 GN=Rpl28 PE=1 SV=2	16 kDa	9
60S ribosomal protein L29 OS=Mus musculus OX=10090 GN=Gm17669 PE=3 SV=1	17 kDa	5
60S ribosomal protein L29 OS=Mus musculus OX=10090 GN=Gm3550 PE=3 SV=1	18 kDa	3
60S ribosomal protein L3 OS=Mus musculus OX=10090 GN=Rpl3 PE=1 SV=3	46 kDa	40
60S ribosomal protein L30 OS=Mus musculus OX=10090 GN=Rpl30 PE=1 SV=2	13 kDa	25
60S ribosomal protein L31 OS=Mus musculus OX=10090 GN=Rpl31 PE=1 SV=1	14 kDa	8
60S ribosomal protein L32 OS=Mus musculus OX=10090 GN=Rpl32 PE=1 SV=2	16 kDa	15
60S ribosomal protein L34 OS=Mus musculus OX=10090 GN=Rpl34 PE=1 SV=2	13 kDa	10
60S ribosomal protein L35 OS=Mus musculus OX=10090 GN=Rpl35 PE=1 SV=1	15 kDa	10
60S ribosomal protein L35a OS=Mus musculus OX=10090 GN=Rpl35a PE=1 SV=2	13 kDa	5
60S ribosomal protein L36 OS=Mus musculus OX=10090 GN=Rpl36 PE=1 SV=1	12 kDa	13
60S ribosomal protein L36 OS=Mus musculus OX=10090 GN=Rpl36 PE=1 SV=2	12 kDa	6
60S ribosomal protein L36a OS=Mus musculus OX=10090 GN=Rpl36a PE=1 SV=2	12 kDa	6
60S ribosomal protein L37a OS=Mus musculus OX=10090 GN=Rpl37a PE=1 SV=2	10 kDa	11
60S ribosomal protein L38 OS=Mus musculus OX=10090 GN=Rpl38 PE=1 SV=3	8 kDa	14
60S ribosomal protein L39 OS=Mus musculus OX=10090 GN=Rpl39 PE=1 SV=2	6 kDa	4
60S ribosomal protein L4 OS=Mus musculus OX=10090 GN=Rpl4 PE=1 SV=3	47 kDa	58
60S ribosomal protein L5 OS=Mus musculus OX=10090 GN=Rpl5 PE=1 SV=3	34 kDa	37
60S ribosomal protein L6 OS=Mus musculus OX=10090 GN=Rpl6 PE=1 SV=3	34 kDa	27
60S ribosomal protein L7 OS=Mus musculus OX=10090 GN=Rpl7 PE=1 SV=2	31 kDa	39

60S ribosomal protein L7a OS=Mus musculus OX=10090 GN=Rpl7a PE=1 SV=2	30 kDa	42
60S ribosomal protein L8 OS=Mus musculus OX=10090 GN=Rpl8 PE=1 SV=2	28 kDa	17
60S ribosomal protein L9 (Fragment) OS=Mus musculus OX=10090 GN=Rpl9 PE=1 SV=1	22 kDa	8
6-phosphofructo-2-kinase/fructose-2,6-bisphosphatase 4 OS=Mus musculus OX=10090 GN=Pfkfb4 PE=2 SV=4	54 kDa	1
6-phosphogluconate dehydrogenase, decarboxylating OS=Mus musculus OX=10090 GN=Pgd PE=1 SV=3	53 kDa	30
6-phosphogluconolactonase OS=Mus musculus OX=10090 GN=Pgls PE=1 SV=1	27 kDa	24
6-pyruvoyl tetrahydrobiopterin synthase OS=Mus musculus OX=10090 GN=Pts PE=1 SV=1	9 kDa	1
Abscission/NoCut checkpoint regulator OS=Mus musculus OX=10090 GN=Zfyve19 PE=1 SV=1	36 kDa	1
Abscission/NoCut checkpoint regulator OS=Mus musculus OX=10090 GN=Zfyve19 PE=1 SV=2	43 kDa	1
Acetyl-CoA acetyltransferase, cytosolic OS=Mus musculus OX=10090 GN=Acat2 PE=1 SV=2	41 kDa	5
Acetyl-CoA acetyltransferase, mitochondrial OS=Mus musculus OX=10090 GN=Acat1 PE=1 SV=1	45 kDa	14
Acetyl-CoA carboxylase 1 OS=Mus musculus OX=10090 GN=Acaca PE=1 SV=1	265 kDa	1
Acid ceramidase OS=Mus musculus OX=10090 GN=Asah1 PE=1 SV=1	45 kDa	12
Acid sphingomyelinase-like phosphodiesterase 3b OS=Mus musculus OX=10090 GN=Smpdl3b PE=1 SV=1	52 kDa	4
Acidic leucine-rich nuclear phosphoprotein 32 family member A OS=Mus musculus OX=10090 GN=Anp32a PE=1 SV=1	29 kDa	30
Acidic leucine-rich nuclear phosphoprotein 32 family member B OS=Mus musculus OX=10090 GN=Anp32b PE=1 SV=1	31 kDa	12
Acidic leucine-rich nuclear phosphoprotein 32 family member E OS=Mus musculus OX=10090 GN=Anp32e PE=1 SV=2	30 kDa	4
Aconitate hydratase, mitochondrial OS=Mus musculus OX=10090 GN=Aco2 PE=1 SV=1	85 kDa	34
Acp1 protein OS=Mus musculus OX=10090 GN=Acp1 PE=1 SV=1	18 kDa	2
Actin, alpha cardiac muscle 1 OS=Mus musculus OX=10090 GN=Actc1 PE=1 SV=1	42 kDa	289
Actin, alpha skeletal muscle OS=Mus musculus OX=10090 GN=Acta1 PE=1 SV=1	42 kDa	289
Actin, cytoplasmic 1 OS=Mus musculus OX=10090 GN=Actb PE=1 SV=1	42 kDa	557
Actin, cytoplasmic 2 OS=Mus musculus OX=10090 GN=Actg1 PE=1 SV=1	42 kDa	551

Actin-like protein 6A OS=Mus musculus OX=10090 GN=Actl6a PE=1 SV=2	47 kDa	8
Actin-like protein 6B OS=Mus musculus OX=10090 GN=Actl6b PE=1 SV=1	47 kDa	3
Actin-related protein 10 OS=Mus musculus OX=10090 GN=Actr10 PE=1 SV=2	46 kDa	4
Actin-related protein 2 OS=Mus musculus OX=10090 GN=Actr2 PE=1 SV=1	45 kDa	34
Actin-related protein 2/3 complex subunit 1A OS=Mus musculus OX=10090 GN=Arpc1a PE=1 SV=1	42 kDa	1
Actin-related protein 2/3 complex subunit 1B OS=Mus musculus OX=10090 GN=Arpc1b PE=1 SV=4	41 kDa	37
Actin-related protein 2/3 complex subunit 2 OS=Mus musculus OX=10090 GN=Arpc2 PE=1 SV=3	34 kDa	21
Actin-related protein 2/3 complex subunit 3 OS=Mus musculus OX=10090 GN=Arpc3 PE=1 SV=3	21 kDa	14
Actin-related protein 2/3 complex subunit 4 OS=Mus musculus OX=10090 GN=Arpc4 PE=1 SV=3	20 kDa	16
Actin-related protein 2/3 complex subunit 5 OS=Mus musculus OX=10090 GN=Arpc5 PE=1 SV=3	16 kDa	6
Actin-related protein 2/3 complex subunit 5-like protein OS=Mus musculus OX=10090 GN=Arpc5l PE=1 SV=1	17 kDa	1
Actin-related protein 3 OS=Mus musculus OX=10090 GN=Actr3 PE=1 SV=3	47 kDa	51
Actin-related protein 3B OS=Mus musculus OX=10090 GN=Actr3b PE=1 SV=1	48 kDa	9
Activated RNA polymerase II transcriptional coactivator p15 OS=Mus musculus OX=10090 GN=Sub1 PE=1 SV=3	14 kDa	23
Activating transcription factor 7-interacting protein 1 OS=Mus musculus OX=10090 GN=Atf7ip PE=1 SV=1	139 kDa	1
Activator of 90 kDa heat shock protein ATPase homolog 1 OS=Mus musculus OX=10090 GN=Ahsa1 PE=1 SV=2	38 kDa	11
Activator of basal transcription 1 OS=Mus musculus OX=10090 GN=Abt1 PE=2 SV=1	31 kDa	1
Activity-dependent neuroprotector homeobox protein OS=Mus musculus OX=10090 GN=Adnp PE=1 SV=2	124 kDa	1
Acylamino-acid-releasing enzyme (Fragment) OS=Mus musculus OX=10090 GN=Apeh PE=1 SV=1	80 kDa	8
Acyl-CoA dehydrogenase family member 9, mitochondrial OS=Mus musculus OX=10090 GN=Acad9 PE=1 SV=2	69 kDa	5
Acyl-CoA-binding domain-containing protein 5 OS=Mus musculus OX=10090 GN=Acbd5 PE=4 SV=1	58 kDa	1
Acyl-CoA-binding domain-containing protein 6 OS=Mus musculus OX=10090 GN=Acbd6 PE=1 SV=2	31 kDa	1
Acyl-CoA-binding protein OS=Mus musculus OX=10090 GN=Dbi PE=1 SV=2	10 kDa	13
Acyl-coenzyme A thioesterase 13 OS=Mus musculus OX=10090 GN=Acot13 PE=1 SV=1	15 kDa	2
Acyl-coenzyme A thioesterase 2, mitochondrial OS=Mus musculus OX=10090 GN=Acot2 PE=1 SV=2	50 kDa	2

Acyl-coenzyme A thioesterase 8 OS=Mus musculus OX=10090 GN=Acot8 PE=1 SV=1	30 kDa	1
Acyl-coenzyme A thioesterase 9, mitochondrial OS=Mus musculus OX=10090 GN=Acot9 PE=1 SV=1	51 kDa	7
Acyl-protein thioesterase 1 OS=Mus musculus OX=10090 GN=Lypla1 PE=1 SV=1	25 kDa	4
Acyl-protein thioesterase 2 OS=Mus musculus OX=10090 GN=Lypla2 PE=1 SV=1	25 kDa	2
Acylpyruvase FAHD1, mitochondrial OS=Mus musculus OX=10090 GN=Fahd1 PE=1 SV=2	25 kDa	2
Adapter molecule crk OS=Mus musculus OX=10090 GN=Crk PE=1 SV=1	34 kDa	6
Adaptin ear-binding coat-associated protein 1 OS=Mus musculus OX=10090 GN=Necap1 PE=1 SV=2	30 kDa	1
Adaptin ear-binding coat-associated protein 2 OS=Mus musculus OX=10090 GN=Necap2 PE=1 SV=1	29 kDa	2
Adenine phosphoribosyltransferase OS=Mus musculus OX=10090 GN=Aprt PE=1 SV=2	20 kDa	28
Adenosine deaminase OS=Mus musculus OX=10090 GN=Ada PE=1 SV=3	40 kDa	5
Adenosine kinase OS=Mus musculus OX=10090 GN=Adk PE=1 SV=2	40 kDa	5
Adenosylhomocysteinase OS=Mus musculus OX=10090 GN=Ahcy PE=1 SV=3	48 kDa	53
Adenylate kinase 2, mitochondrial OS=Mus musculus OX=10090 GN=Ak2 PE=1 SV=5	26 kDa	13
Adenylate kinase isoenzyme 1 OS=Mus musculus OX=10090 GN=Ak1 PE=1 SV=1	22 kDa	6
Adenylosuccinate lyase OS=Mus musculus OX=10090 GN=Adsl PE=1 SV=2	55 kDa	28
Adenylosuccinate synthetase isozyme 1 OS=Mus musculus OX=10090 GN=Adssl1 PE=1 SV=1	53 kDa	7
Adenylosuccinate synthetase isozyme 2 OS=Mus musculus OX=10090 GN=Adss PE=1 SV=2	50 kDa	30
Adenylyl cyclase-associated protein 1 OS=Mus musculus OX=10090 GN=Cap1 PE=1 SV=4	52 kDa	90
Adipocyte plasma membrane-associated protein OS=Mus musculus OX=10090 GN=Apmap PE=1 SV=1	46 kDa	4
ADP/ATP translocase 1 OS=Mus musculus OX=10090 GN=Slc25a4 PE=1 SV=4	33 kDa	17
ADP/ATP translocase 2 OS=Mus musculus OX=10090 GN=Slc25a5 PE=1 SV=3	33 kDa	23
ADP-dependent glucokinase OS=Mus musculus OX=10090 GN=Adpgk PE=1 SV=1	54 kDa	6
ADP-ribosyl cyclase/cyclic ADP-ribose hydrolase 1 OS=Mus musculus OX=10090 GN=Cd38 PE=1 SV=2	34 kDa	1
ADP-ribosylation factor 1 OS=Mus musculus OX=10090 GN=Arf1 PE=1 SV=2	21 kDa	13
ADP-ribosylation factor 4 OS=Mus musculus OX=10090 GN=Arf4 PE=1 SV=2	20 kDa	5

ADP-ribosylation factor 5 OS=Mus musculus OX=10090 GN=Arf5 PE=1 SV=2	21 kDa	4
ADP-ribosylation factor 6 OS=Mus musculus OX=10090 GN=Arf6 PE=1 SV=2	20 kDa	1
ADP-ribosylation factor GTPase-activating protein 1 OS=Mus musculus OX=10090 GN=Arfgap1 PE=1 SV=2	45 kDa	2
ADP-ribosylation factor GTPase-activating protein 2 OS=Mus musculus OX=10090 GN=Arfgap2 PE=1 SV=1	57 kDa	4
ADP-ribosylation factor-interacting protein 1 OS=Mus musculus OX=10090 GN=Arfip1 PE=1 SV=1	42 kDa	6
ADP-ribosylation factor-like protein 1 OS=Mus musculus OX=10090 GN=Arl1 PE=1 SV=1	20 kDa	1
ADP-ribosylation factor-like protein 2 OS=Mus musculus OX=10090 GN=Arl2 PE=1 SV=1	21 kDa	3
ADP-ribosylation factor-like protein 2-binding protein OS=Mus musculus OX=10090 GN=Arl2bp PE=1 SV=1	19 kDa	1
ADP-ribosylation factor-like protein 3 OS=Mus musculus OX=10090 GN=Arl3 PE=1 SV=1	20 kDa	7
ADP-ribosylation factor-like protein 6-interacting protein 1 OS=Mus musculus OX=10090 GN=Arl6ip1 PE=1 SV=1	23 kDa	2
ADP-ribosylation factor-like protein 8B OS=Mus musculus OX=10090 GN=Arl8b PE=1 SV=1	16 kDa	8
ADP-sugar pyrophosphatase OS=Mus musculus OX=10090 GN=Nudt5 PE=1 SV=1	24 kDa	5
Adrenodoxin, mitochondrial OS=Mus musculus OX=10090 GN=Fdx1 PE=1 SV=1	20 kDa	3
Afadin OS=Mus musculus OX=10090 GN=Afdn PE=1 SV=2	205 kDa	1
AH receptor-interacting protein OS=Mus musculus OX=10090 GN=Aip PE=1 SV=1	38 kDa	3
AHNAK nucleoprotein (desmoyokin) OS=Mus musculus OX=10090 GN=Ahnak PE=1 SV=1	604 kDa	898
AHNAK nucleoprotein 2 (Fragment) OS=Mus musculus OX=10090 GN=Ahnak2 PE=1 SV=1	166 kDa	35
AHNAK nucleoprotein 2 (Fragment) OS=Mus musculus OX=10090 GN=Ahnak2 PE=1 SV=1	115 kDa	28
AHNAK nucleoprotein 2 (Fragment) OS=Mus musculus OX=10090 GN=Ahnak2 PE=1 SV=8	183 kDa	29
AHNAK nucleoprotein 2 OS=Mus musculus OX=10090 GN=Ahnak2 PE=1 SV=1	83 kDa	8
A-kinase anchor protein 2 OS=Mus musculus OX=10090 GN=Akap2 PE=1 SV=3	99 kDa	12
A-kinase anchor protein 8 OS=Mus musculus OX=10090 GN=Akap8 PE=1 SV=1	76 kDa	7
Alanine aminotransferase 2 OS=Mus musculus OX=10090 GN=Gpt2 PE=1 SV=1	58 kDa	1
Alanine--tRNA ligase, cytoplasmic OS=Mus musculus OX=10090 GN=Aars PE=1 SV=1	107 kDa	15
Alcohol dehydrogenase [NADP(+)] OS=Mus musculus OX=10090 GN=Akr1a1 PE=1 SV=3	37 kDa	4

Alcohol dehydrogenase class-3 OS=Mus musculus OX=10090 GN=Adh5 PE=1 SV=3	40 kDa	25
Aldehyde dehydrogenase family 16 member A1 OS=Mus musculus OX=10090 GN=Aldh16a1 PE=1 SV=1	85 kDa	7
Aldehyde dehydrogenase, mitochondrial OS=Mus musculus OX=10090 GN=Aldh2 PE=1 SV=1	57 kDa	18
Aldo-keto reductase family 1, member B10 (aldose reductase) OS=Mus musculus OX=10090 GN=Akr1b10 PE=1 SV=1	33 kDa	3
Aldose 1-epimerase OS=Mus musculus OX=10090 GN=Galm PE=1 SV=1	38 kDa	1
Aldose reductase OS=Mus musculus OX=10090 GN=Akr1b1 PE=1 SV=3	36 kDa	15
Aldose reductase-related protein 2 OS=Mus musculus OX=10090 GN=Akr1b8 PE=1 SV=2	36 kDa	5
Alkylglycerone-phosphate synthase OS=Mus musculus OX=10090 GN=Agps PE=1 SV=1	56 kDa	1
alpha-1,2-Mannosidase (Fragment) OS=Mus musculus OX=10090 GN=Man1a PE=1 SV=1	82 kDa	1
Alpha-1,6-mannosyl-glycoprotein 2-beta-N-acetylglucosaminyltransferase OS=Mus musculus OX=10090 GN=Mgat2 PE=1 SV=1	51 kDa	1
Alpha-2-macroglobulin receptor-associated protein OS=Mus musculus OX=10090 GN=Lrpap1 PE=1 SV=1	42 kDa	5
Alpha-actinin-1 OS=Mus musculus OX=10090 GN=Actn1 PE=1 SV=1	103 kDa	162
Alpha-actinin-2 OS=Mus musculus OX=10090 GN=Actn2 PE=1 SV=2	104 kDa	19
Alpha-actinin-3 OS=Mus musculus OX=10090 GN=Actn3 PE=2 SV=1	103 kDa	11
Alpha-actinin-4 OS=Mus musculus OX=10090 GN=Actn4 PE=1 SV=1	105 kDa	169
Alpha-aminoadipic semialdehyde dehydrogenase OS=Mus musculus OX=10090 GN=Aldh7a1 PE=1 SV=4	59 kDa	5
Alpha-centractin OS=Mus musculus OX=10090 GN=Actr1a PE=1 SV=1	43 kDa	9
AlphaCstF-64 variant 4 OS=Mus musculus OX=10090 GN=Cstf2 PE=1 SV=1	59 kDa	5
Alpha-endosulfine OS=Mus musculus OX=10090 GN=Ensa PE=1 SV=1	13 kDa	2
Alpha-enolase OS=Mus musculus OX=10090 GN=Eno1 PE=1 SV=3	47 kDa	217
Alpha-ketoglutarate-dependent dioxygenase alkB homolog 3 OS=Mus musculus OX=10090 GN=Alkbh3 PE=1 SV=1	33 kDa	1
Alpha-ketoglutarate-dependent dioxygenase FTO OS=Mus musculus OX=10090 GN=Fto PE=1 SV=1	58 kDa	6
Alpha-mannosidase 2 OS=Mus musculus OX=10090 GN=Man2a1 PE=1 SV=2	132 kDa	11
Alpha-N-acetylgalactosaminidase OS=Mus musculus OX=10090 GN=Naga PE=1 SV=2	47 kDa	1

Alpha-N-acetylglucosaminidase OS=Mus musculus OX=10090 GN=Naglu PE=1 SV=1	83 kDa	5
Alpha-soluble NSF attachment protein OS=Mus musculus OX=10090 GN=Napa PE=1 SV=1	33 kDa	4
Alpha-taxilin OS=Mus musculus OX=10090 GN=Txlna PE=1 SV=1	62 kDa	5
Amidophosphoribosyltransferase OS=Mus musculus OX=10090 GN=Ppat PE=1 SV=1	57 kDa	7
Amine oxidase [flavin-containing] A OS=Mus musculus OX=10090 GN=Maoa PE=1 SV=3	60 kDa	7
Amino acid transporter OS=Mus musculus OX=10090 GN=Slc1a5 PE=1 SV=1	58 kDa	3
Aminoacyl tRNA synthase complex-interacting multifunctional protein 1 OS=Mus musculus OX=10090 GN=Aimp1 PE=1 SV=2	34 kDa	11
Aminoacyl tRNA synthase complex-interacting multifunctional protein 2 OS=Mus musculus OX=10090 GN=Aimp2 PE=1 SV=2	35 kDa	2
Aminoacylase-1 OS=Mus musculus OX=10090 GN=Acy1 PE=1 SV=1	46 kDa	3
Aminopeptidase B OS=Mus musculus OX=10090 GN=Rnpep PE=1 SV=2	72 kDa	1
AMP deaminase OS=Mus musculus OX=10090 GN=Ampd2 PE=1 SV=1	95 kDa	5
Amyloid beta A4 precursor protein-binding family B member 1-interacting protein OS=Mus musculus OX=10090 GN=Apbb1ip PE=1 SV=2	74 kDa	2
AN1-type zinc finger protein 5 OS=Mus musculus OX=10090 GN=Zfand5 PE=1 SV=1	23 kDa	3
Anamorsin OS=Mus musculus OX=10090 GN=Ciapin1 PE=1 SV=1	33 kDa	3
Angio-associated migratory protein OS=Mus musculus OX=10090 GN=Aamp PE=1 SV=1	47 kDa	9
Anillin OS=Mus musculus OX=10090 GN=Anln PE=1 SV=2	123 kDa	5
Ankycorbin OS=Mus musculus OX=10090 GN=Rai14 PE=4 SV=1	106 kDa	5
Annexin A1 OS=Mus musculus OX=10090 GN=Anxa1 PE=1 SV=2	39 kDa	84
Annexin A2 OS=Mus musculus OX=10090 GN=Anxa2 PE=1 SV=2	39 kDa	44
Annexin A3 OS=Mus musculus OX=10090 GN=Anxa3 PE=1 SV=4	36 kDa	10
Annexin A4 OS=Mus musculus OX=10090 GN=Anxa4 PE=1 SV=4	36 kDa	2
Annexin A5 OS=Homo sapiens GN=ANXA5 PE=1 SV=2	36 kDa	23
Annexin A5 OS=Mus musculus OX=10090 GN=Anxa5 PE=1 SV=1	36 kDa	55
Annexin OS=Mus musculus OX=10090 GN=Anxa11 PE=1 SV=1	48 kDa	2

Annexin OS=Mus musculus OX=10090 GN=Anxa7 PE=1 SV=1	52 kDa	2
AP-1 complex subunit beta-1 OS=Mus musculus OX=10090 GN=Ap1b1 PE=1 SV=2	104 kDa	18
AP-1 complex subunit gamma-1 OS=Mus musculus OX=10090 GN=Ap1g1 PE=1 SV=3	91 kDa	2
AP-1 complex subunit mu-1 OS=Mus musculus OX=10090 GN=Ap1m1 PE=1 SV=3	49 kDa	4
AP-1 complex subunit sigma-1A OS=Mus musculus OX=10090 GN=Ap1s1 PE=1 SV=1	19 kDa	1
AP-2 complex subunit alpha-1 OS=Mus musculus OX=10090 GN=Ap2a1 PE=1 SV=1	108 kDa	14
AP-2 complex subunit alpha-2 OS=Mus musculus OX=10090 GN=Ap2a2 PE=1 SV=2	104 kDa	14
AP-2 complex subunit beta OS=Mus musculus OX=10090 GN=Ap2b1 PE=1 SV=1	105 kDa	26
AP-2 complex subunit mu OS=Mus musculus OX=10090 GN=Ap2m1 PE=1 SV=1	50 kDa	4
AP2-associated protein kinase 1 OS=Mus musculus OX=10090 GN=Aak1 PE=1 SV=2	103 kDa	1
AP-3 complex subunit delta-1 OS=Mus musculus OX=10090 GN=Ap3d1 PE=1 SV=1	135 kDa	1
AP-3 complex subunit sigma-1 OS=Mus musculus OX=10090 GN=Ap3s1 PE=1 SV=2	22 kDa	1
Apolipoprotein B receptor OS=Mus musculus OX=10090 GN=Apobr PE=1 SV=1	103 kDa	14
Apoptosis inhibitor 5 OS=Mus musculus OX=10090 GN=Api5 PE=1 SV=2	57 kDa	15
Apoptosis regulator BAX (Fragment) OS=Mus musculus OX=10090 GN=Bax PE=1 SV=1	13 kDa	16
Apoptosis-inducing factor 1, mitochondrial OS=Mus musculus OX=10090 GN=Aifm1 PE=1 SV=1	67 kDa	16
Apoptotic chromatin condensation inducer in the nucleus OS=Mus musculus OX=10090 GN=Acin1 PE=1 SV=3	151 kDa	7
Aprataxin and PNK-like factor OS=Mus musculus OX=10090 GN=Aplf PE=1 SV=2	55 kDa	1
Arf-GAP domain and FG repeat-containing protein 1 OS=Mus musculus OX=10090 GN=Agfg1 PE=1 SV=1	54 kDa	4
Arginine/serine-rich protein PNISR OS=Mus musculus OX=10090 GN=Pnisr PE=1 SV=1	93 kDa	1
Arginine-rich, mutated in early stage tumors, isoform CRA_b OS=Mus musculus OX=10090 GN=Manf PE=1 SV=1	20 kDa	29
Arginine--tRNA ligase, cytoplasmic OS=Mus musculus OX=10090 GN=Rars PE=1 SV=2	76 kDa	15
Argininosuccinate lyase OS=Mus musculus OX=10090 GN=Asl PE=1 SV=1	52 kDa	5
Arginyl-tRNA--protein transferase 1 OS=Mus musculus OX=10090 GN=Ate1 PE=1 SV=1	58 kDa	1
Arpin OS=Mus musculus OX=10090 GN=Arpin PE=1 SV=1	25 kDa	5

Arsenite methyltransferase OS=Mus musculus OX=10090 GN=As3mt PE=1 SV=2	42 kDa	1
Arylsulfatase A OS=Mus musculus OX=10090 GN=Arsa PE=1 SV=2	54 kDa	5
Arylsulfatase B OS=Mus musculus OX=10090 GN=Arsb PE=1 SV=1	60 kDa	2
Asparagine synthetase [glutamine-hydrolyzing] OS=Mus musculus OX=10090 GN=Asns PE=1 SV=3	64 kDa	5
Asparagine--tRNA ligase, cytoplasmic OS=Mus musculus OX=10090 GN=Nars PE=1 SV=2	64 kDa	17
Aspartate aminotransferase, cytoplasmic OS=Mus musculus OX=10090 GN=Got1 PE=1 SV=3	46 kDa	35
Aspartate aminotransferase, mitochondrial OS=Mus musculus OX=10090 GN=Got2 PE=1 SV=1	47 kDa	81
Aspartate--tRNA ligase, cytoplasmic OS=Mus musculus OX=10090 GN=Dars PE=1 SV=2	57 kDa	44
Aspartyl aminopeptidase OS=Mus musculus OX=10090 GN=Dnpep PE=1 SV=2	52 kDa	21
Aspartyl/asparaginyl beta-hydroxylase OS=Mus musculus OX=10090 GN=Asph PE=1 SV=1	81 kDa	11
Aspartyl/asparaginyl beta-hydroxylase OS=Mus musculus OX=10090 GN=Asph PE=1 SV=1	26 kDa	3
Astrocytic phosphoprotein PEA-15 OS=Mus musculus OX=10090 GN=Pea15 PE=1 SV=1	15 kDa	1
Ataxin-10 OS=Mus musculus OX=10090 GN=Atxn10 PE=1 SV=2	54 kDa	4
Ataxin-1-like OS=Mus musculus OX=10090 GN=Atxn1l PE=1 SV=1	73 kDa	1
Ataxin-2 OS=Mus musculus OX=10090 GN=Atxn2 PE=1 SV=1	136 kDa	2
Ataxin-2-like protein OS=Mus musculus OX=10090 GN=Atxn2l PE=1 SV=1	115 kDa	15
ATP synthase F(0) complex subunit B1, mitochondrial OS=Mus musculus OX=10090 GN=Atp5f1 PE=1 SV=1	29 kDa	3
ATP synthase protein 8 OS=Mus musculus OX=10090 GN=Mtatp8 PE=1 SV=1	8 kDa	2
ATP synthase subunit alpha, mitochondrial OS=Mus musculus OX=10090 GN=Atp5a1 PE=1 SV=1	60 kDa	106
ATP synthase subunit beta, mitochondrial OS=Mus musculus OX=10090 GN=Atp5b PE=1 SV=2	56 kDa	153
ATP synthase subunit d, mitochondrial (Fragment) OS=Mus musculus OX=10090 GN=Atp5h PE=1 SV=1	16 kDa	14
ATP synthase subunit d, mitochondrial OS=Mus musculus OX=10090 GN=Atp5h PE=1 SV=3	19 kDa	20
ATP synthase subunit delta, mitochondrial OS=Mus musculus OX=10090 GN=Atp5d PE=1 SV=1	18 kDa	5
ATP synthase subunit e, mitochondrial OS=Mus musculus OX=10090 GN=Atp5i PE=1 SV=2	8 kDa	4
ATP synthase subunit epsilon, mitochondrial OS=Mus musculus OX=10090 GN=Atp5e PE=1 SV=2	6 kDa	4

ATP synthase subunit f, mitochondrial OS=Mus musculus OX=10090 GN=Atp5j2 PE=1 SV=3	10 kDa	5
ATP synthase subunit g, mitochondrial OS=Mus musculus OX=10090 GN=Atp5l PE=1 SV=1	11 kDa	2
ATP synthase subunit gamma, mitochondrial OS=Mus musculus OX=10090 GN=Atp5c1 PE=1 SV=1	33 kDa	30
ATP synthase subunit O, mitochondrial OS=Mus musculus OX=10090 GN=Atp5o PE=1 SV=1	23 kDa	9
ATP synthase-coupling factor 6, mitochondrial OS=Mus musculus OX=10090 GN=Atp5j PE=1 SV=1	12 kDa	3
ATPase Asna1 OS=Mus musculus OX=10090 GN=Asna1 PE=1 SV=2	39 kDa	4
ATPase inhibitor, mitochondrial OS=Mus musculus OX=10090 GN=Atpif1 PE=1 SV=2	12 kDa	11
ATP-binding cassette sub-family E member 1 OS=Mus musculus OX=10090 GN=Abce1 PE=1 SV=1	67 kDa	31
ATP-binding cassette sub-family F member 1 OS=Mus musculus OX=10090 GN=Abcf1 PE=1 SV=1	95 kDa	4
ATP-binding cassette sub-family F member 2 OS=Mus musculus OX=10090 GN=Abcf2 PE=1 SV=1	72 kDa	4
ATP-citrate synthase OS=Mus musculus OX=10090 GN=Acly PE=1 SV=1	120 kDa	10
ATP-dependent (S)-NAD(P)H-hydrate dehydratase OS=Mus musculus OX=10090 GN=Naxd PE=1 SV=1	35 kDa	2
ATP-dependent 6-phosphofructokinase, liver type OS=Mus musculus OX=10090 GN=Pfkl PE=1 SV=4	85 kDa	17
ATP-dependent 6-phosphofructokinase, muscle type OS=Mus musculus OX=10090 GN=Pfkm PE=1 SV=3	85 kDa	4
ATP-dependent 6-phosphofructokinase, platelet type OS=Mus musculus OX=10090 GN=Pfkp PE=1 SV=1	85 kDa	6
ATP-dependent Clp protease ATP-binding subunit clpX-like, mitochondrial OS=Mus musculus OX=10090 GN=Clpx PE=1 SV=2	69 kDa	2
ATP-dependent Clp protease proteolytic subunit, mitochondrial OS=Mus musculus OX=10090 GN=Clpp PE=1 SV=1	30 kDa	14
ATP-dependent RNA helicase A OS=Mus musculus OX=10090 GN=Dhx9 PE=1 SV=1	150 kDa	18
ATP-dependent RNA helicase DDX1 OS=Mus musculus OX=10090 GN=Ddx1 PE=1 SV=1	83 kDa	4
ATP-dependent RNA helicase DDX18 OS=Mus musculus OX=10090 GN=Ddx18 PE=1 SV=1	74 kDa	2
ATP-dependent RNA helicase DDX19A OS=Mus musculus OX=10090 GN=Ddx19a PE=1 SV=2	54 kDa	11
ATP-dependent RNA helicase DDX25 OS=Mus musculus OX=10090 GN=Ddx25 PE=1 SV=2	55 kDa	1
ATP-dependent RNA helicase DDX39A OS=Mus musculus OX=10090 GN=Ddx39a PE=1 SV=1	49 kDa	22
ATP-dependent RNA helicase DDX3X OS=Mus musculus OX=10090 GN=Ddx3x PE=1 SV=3	73 kDa	20

ATP-dependent RNA helicase DDX3Y OS=Mus musculus OX=10090 GN=Ddx3y PE=1 SV=2	73 kDa	16
ATP-dependent RNA helicase DDX4 OS=Mus musculus OX=10090 GN=Ddx4 PE=1 SV=1	79 kDa	4
ATP-dependent RNA helicase DHX8 (Fragment) OS=Mus musculus OX=10090 GN=Dhx8 PE=1 SV=8	134 kDa	1
AT-rich interactive domain-containing protein 1A OS=Mus musculus OX=10090 GN=Arid1a PE=1 SV=1	242 kDa	1
Atrophin-1 OS=Mus musculus OX=10090 GN=Atn1 PE=1 SV=1	124 kDa	1
Autophagy-related protein 16-1 OS=Mus musculus OX=10090 GN=Atg16l1 PE=1 SV=1	68 kDa	1
BAG family molecular chaperone regulator 2 OS=Mus musculus OX=10090 GN=Bag2 PE=1 SV=1	23 kDa	2
BAG family molecular chaperone regulator 3 OS=Mus musculus OX=10090 GN=Bag3 PE=1 SV=2	62 kDa	27
Band 4.1-like protein 1 OS=Mus musculus OX=10090 GN=Epb41l1 PE=1 SV=1	98 kDa	3
Band 4.1-like protein 2 OS=Mus musculus OX=10090 GN=Epb41l2 PE=1 SV=2	110 kDa	15
Band 4.1-like protein 3 (Fragment) OS=Mus musculus OX=10090 GN=Epb41l3 PE=1 SV=1	95 kDa	2
Basement membrane-specific heparan sulfate proteoglycan core protein OS=Mus musculus OX=10090 GN=Hspg2 PE=1 SV=1	469 kDa	10
Basic leucine zipper and W2 domain-containing protein 1 OS=Mus musculus OX=10090 GN=Bzw1 PE=1 SV=1	51 kDa	18
Basic leucine zipper and W2 domain-containing protein 2 OS=Mus musculus OX=10090 GN=Bzw2 PE=1 SV=1	48 kDa	9
Basigin (Fragment) OS=Mus musculus OX=10090 GN=Bsg PE=1 SV=1	22 kDa	18
Basigin OS=Mus musculus OX=10090 GN=Bsg PE=1 SV=2	42 kDa	17
BC035947 protein OS=Mus musculus OX=10090 GN=BC035947 PE=1 SV=1	77 kDa	1
B-cell CLL/lymphoma 7 protein family member C OS=Mus musculus OX=10090 GN=Bcl7c PE=1 SV=1	26 kDa	1
B-cell receptor-associated protein 29 OS=Mus musculus OX=10090 GN=Bcap29 PE=1 SV=1	28 kDa	2
B-cell receptor-associated protein 31 OS=Mus musculus OX=10090 GN=Bcap31 PE=1 SV=4	28 kDa	26
Bcl-2 homologous antagonist/killer OS=Mus musculus OX=10090 GN=Bak1 PE=1 SV=3	23 kDa	4
Bcl-2-associated transcription factor 1 OS=Mus musculus OX=10090 GN=Bclaf1 PE=1 SV=1	86 kDa	4
Bcl-2-like protein 13 OS=Mus musculus OX=10090 GN=Bcl2l13 PE=1 SV=2	47 kDa	1
Beta-2-microglobulin OS=Mus musculus OX=10090 GN=B2m PE=1 SV=2	14 kDa	1
Beta-actin-like protein 2 OS=Mus musculus OX=10090 GN=Actbl2 PE=1 SV=1	42 kDa	129

Beta-enolase OS=Mus musculus OX=10090 GN=Eno3 PE=1 SV=3	47 kDa	39
Beta-galactosidase OS=Mus musculus OX=10090 GN=Glb1 PE=1 SV=1	73 kDa	8
Beta-glucuronidase OS=Mus musculus OX=10090 GN=Gusb PE=1 SV=2	74 kDa	8
Beta-hexosaminidase subunit alpha OS=Mus musculus OX=10090 GN=Hexa PE=1 SV=2	61 kDa	2
Beta-hexosaminidase subunit beta OS=Mus musculus OX=10090 GN=Hexb PE=1 SV=2	61 kDa	3
Bifunctional 3'-phosphoadenosine 5'-phosphosulfate synthase 1 OS=Mus musculus OX=10090 GN=Papss1 PE=1 SV=1	71 kDa	14
Bifunctional glutamate/proline--tRNA ligase OS=Mus musculus OX=10090 GN=Eprs PE=1 SV=4	170 kDa	46
Bifunctional methylenetetrahydrofolate dehydrogenase/cyclohydrolase, mitochondrial OS=Mus musculus OX=10090 GN=Mthfd2 PE=1 SV=1	38 kDa	7
Bifunctional purine biosynthesis protein PURH OS=Mus musculus OX=10090 GN=Atic PE=1 SV=2	64 kDa	31
Biliverdin reductase A OS=Mus musculus OX=10090 GN=Blvra PE=1 SV=1	34 kDa	1
Bin1 protein OS=Mus musculus OX=10090 GN=Bin1 PE=1 SV=1	53 kDa	16
Biogenesis of lysosome-related organelles complex 1 subunit 2 OS=Mus musculus OX=10090 GN=Bloc1s2 PE=1 SV=1	16 kDa	1
Biorientation of chromosomes in cell division protein 1-like 1 OS=Mus musculus OX=10090 GN=Bod1l PE=1 SV=1	327 kDa	2
Bis(5'-nucleosyl)-tetraphosphatase [asymmetrical] OS=Mus musculus OX=10090 GN=Nudt2 PE=1 SV=3	17 kDa	2
Bleomycin hydrolase OS=Mus musculus OX=10090 GN=Blmh PE=1 SV=1	53 kDa	21
BMS1 homolog, ribosome assembly protein (Yeast) OS=Mus musculus OX=10090 GN=Bms1 PE=1 SV=1	145 kDa	1
BolA-like protein 1 OS=Mus musculus OX=10090 GN=Bola1 PE=1 SV=1	14 kDa	2
BolA-like protein 2 OS=Mus musculus OX=10090 GN=Bola2 PE=1 SV=1	10 kDa	1
Brain acid soluble protein 1 OS=Mus musculus OX=10090 GN=Basp1 PE=1 SV=3	22 kDa	2
Brain-specific angiogenesis inhibitor 1-associated protein 2 OS=Mus musculus OX=10090 GN=Baiap2 PE=1 SV=1	58 kDa	7
Branched-chain-amino-acid aminotransferase OS=Mus musculus OX=10090 GN=Bcat2 PE=1 SV=1	43 kDa	2
Branched-chain-amino-acid aminotransferase, cytosolic (Fragment) OS=Mus musculus OX=10090 GN=Bcat1 PE=1 SV=1	20 kDa	2
Branched-chain-amino-acid aminotransferase, cytosolic OS=Mus musculus OX=10090 GN=Bcat1 PE=1 SV=2	43 kDa	13

BRCA2 and CDKN1A-interacting protein OS=Mus musculus OX=10090 GN=Bccip PE=1 SV=1	36 kDa	1
Breast cancer anti-estrogen resistance protein 1 OS=Mus musculus OX=10090 GN=Bcar1 PE=1 SV=2	94 kDa	1
BRI3-binding protein OS=Mus musculus OX=10090 GN=Bri3bp PE=1 SV=1	28 kDa	1
BRISC and BRCA1-A complex member 1 (Fragment) OS=Mus musculus OX=10090 GN=Babam1 PE=1 SV=1	13 kDa	1
BRISC and BRCA1-A complex member 2 OS=Mus musculus OX=10090 GN=Babam2 PE=1 SV=2	44 kDa	5
BRISC complex subunit Abraxas 2 OS=Mus musculus OX=10090 GN=Abraxas2 PE=1 SV=1	47 kDa	4
BRO1 domain-containing protein BROX OS=Mus musculus OX=10090 GN=Brox PE=1 SV=1	46 kDa	1
Bromodomain-containing protein 3 OS=Mus musculus OX=10090 GN=Brd3 PE=1 SV=2	80 kDa	2
Bromodomain-containing protein 7 OS=Mus musculus OX=10090 GN=Brd7 PE=1 SV=1	74 kDa	1
BSD domain-containing protein 1 OS=Mus musculus OX=10090 GN=Bsdc1 PE=1 SV=1	47 kDa	1
BTB/POZ domain-containing protein KCTD5 OS=Mus musculus OX=10090 GN=Kctd5 PE=1 SV=1	26 kDa	1
BUB3-interacting and GLEBS motif-containing protein ZNF207 OS=Mus musculus OX=10090 GN=Znf207 PE=1 SV=1	53 kDa	5
C-1-tetrahydrofolate synthase, cytoplasmic OS=Mus musculus OX=10090 GN=Mthfd1 PE=1 SV=4	101 kDa	16
CAAX prenyl protease 1 homolog OS=Mus musculus OX=10090 GN=Zmpste24 PE=1 SV=2	55 kDa	1
CAD protein OS=Mus musculus OX=10090 GN=Cad PE=1 SV=1	236 kDa	41
Calcineurin subunit B type 1 OS=Mus musculus OX=10090 GN=Ppp3r1 PE=1 SV=3	19 kDa	1
Calcium homeostasis endoplasmic reticulum protein OS=Mus musculus OX=10090 GN=Cherp PE=1 SV=1	106 kDa	1
Calcium/calmodulin-dependent protein kinase type 1 OS=Mus musculus OX=10090 GN=Camk1 PE=1 SV=1	42 kDa	2
Calcium/calmodulin-dependent protein kinase type 1D OS=Mus musculus OX=10090 GN=Camk1d PE=1 SV=2	43 kDa	2
Calcium/calmodulin-dependent protein kinase type II subunit delta OS=Mus musculus OX=10090 GN=Camk2d PE=1 SV=1	60 kDa	12
Calcium-binding mitochondrial carrier protein Aralar1 OS=Mus musculus OX=10090 GN=Slc25a12 PE=1 SV=1	75 kDa	1
Calcium-binding mitochondrial carrier protein Aralar2 OS=Mus musculus OX=10090 GN=Slc25a13 PE=1 SV=1	74 kDa	1
Calcium-binding mitochondrial carrier protein SCaMC-1 OS=Mus musculus OX=10090 GN=Slc25a24 PE=1 SV=1	53 kDa	7
Calcium-regulated heat stable protein 1 OS=Mus musculus OX=10090 GN=Carhsp1 PE=1 SV=1	16 kDa	4

Calcium-transporting ATPase OS=Mus musculus OX=10090 GN=Atp2b3 PE=1 SV=1	134 kDa	2
Calcyclin-binding protein OS=Mus musculus OX=10090 GN=Cacybp PE=1 SV=1	27 kDa	27
Caldesmon 1 OS=Mus musculus OX=10090 GN=Cald1 PE=1 SV=1	60 kDa	83
Caldesmon 1 OS=Mus musculus OX=10090 GN=Cald1 PE=1 SV=1	89 kDa	76
Calmodulin-1 OS=Mus musculus OX=10090 GN=Calm1 PE=1 SV=1	17 kDa	15
Calmodulin-like protein 3 (Fragment) OS=Mus musculus OX=10090 GN=Calml3 PE=1 SV=1	13 kDa	6
Calnexin OS=Mus musculus OX=10090 GN=Canx PE=1 SV=1	67 kDa	52
Calpain small subunit 1 OS=Mus musculus OX=10090 GN=Capns1 PE=1 SV=1	28 kDa	26
Calpain-1 catalytic subunit OS=Mus musculus OX=10090 GN=Capn1 PE=1 SV=1	82 kDa	9
Calpain-2 catalytic subunit OS=Mus musculus OX=10090 GN=Capn2 PE=1 SV=4	80 kDa	43
Calpastatin OS=Mus musculus OX=10090 GN=Cast PE=1 SV=1	81 kDa	35
Calpastatin OS=Mus musculus OX=10090 GN=Cast PE=1 SV=2	85 kDa	35
Calponin OS=Mus musculus OX=10090 GN=Cnn3 PE=1 SV=1	31 kDa	11
Calponin-2 OS=Mus musculus OX=10090 GN=Cnn2 PE=1 SV=1	33 kDa	7
Calponin-3 OS=Mus musculus OX=10090 GN=Cnn3 PE=1 SV=1	36 kDa	13
Calreticulin OS=Mus musculus OX=10090 GN=Calr PE=1 SV=1	48 kDa	55
Calumenin OS=Mus musculus OX=10090 GN=Calu PE=1 SV=1	37 kDa	25
Calumenin OS=Mus musculus OX=10090 GN=Calu PE=1 SV=1	37 kDa	28
cAMP-dependent protein kinase catalytic subunit beta OS=Mus musculus OX=10090 GN=Prkacb PE=1 SV=2	41 kDa	2
cAMP-dependent protein kinase type I-alpha regulatory subunit OS=Mus musculus OX=10090 GN=Prkar1a PE=1 SV=3	43 kDa	9
cAMP-dependent protein kinase type II-alpha regulatory subunit OS=Mus musculus OX=10090 GN=Prkar2a PE=1 SV=2	45 kDa	1
cAMP-regulated phosphoprotein 19 OS=Mus musculus OX=10090 GN=Arpp19 PE=1 SV=2	12 kDa	10
cAMP-responsive element modulator OS=Mus musculus OX=10090 GN=Crem PE=1 SV=1	12 kDa	1
CAP-Gly domain-containing linker protein 1 (Fragment) OS=Mus musculus OX=10090 GN=Clip1 PE=1 SV=1	136 kDa	2

CAP-Gly domain-containing linker protein 1 OS=Mus musculus OX=10090 GN=Clip1 PE=1 SV=1	156 kDa	5
CAP-Gly domain-containing linker protein 2 OS=Mus musculus OX=10090 GN=Clip2 PE=1 SV=2	116 kDa	5
Capping protein (Actin filament) muscle Z-line, alpha 1 OS=Mus musculus OX=10090 GN=Capza1 PE=1 SV=1	33 kDa	19
Capping protein (Actin filament) muscle Z-line, beta, isoform CRA_a OS=Mus musculus OX=10090 GN=Capzb PE=1 SV=1	29 kDa	62
Capping protein (Actin filament), gelsolin-like OS=Mus musculus OX=10090 GN=Capg PE=1 SV=1	39 kDa	32
Caprin-1 OS=Mus musculus OX=10090 GN=Caprin1 PE=1 SV=2	78 kDa	26
Carboxypeptidase OS=Mus musculus OX=10090 GN=Ctsa PE=1 SV=1	56 kDa	11
Carboxypeptidase Q OS=Mus musculus OX=10090 GN=Cpq PE=1 SV=1	52 kDa	5
Casein kinase II subunit alpha OS=Mus musculus OX=10090 GN=Csnk2a1 PE=1 SV=2	45 kDa	7
Casein kinase II subunit alpha' OS=Mus musculus OX=10090 GN=Csnk2a2 PE=1 SV=1	41 kDa	2
Casein kinase II subunit beta OS=Mus musculus OX=10090 GN=Csnk2b PE=1 SV=1	25 kDa	3
Casitas B-lineage lymphoma b OS=Mus musculus OX=10090 GN=Cblb PE=1 SV=1	105 kDa	1
Caspase-3 OS=Mus musculus OX=10090 GN=Casp3 PE=1 SV=1	31 kDa	17
Caspase-3 OS=Mus musculus OX=10090 GN=Casp3 PE=1 SV=1	15 kDa	6
Caspase-6 OS=Mus musculus OX=10090 GN=Casp6 PE=1 SV=1	32 kDa	3
Caspase-8 OS=Mus musculus OX=10090 GN=Casp8 PE=1 SV=1	58 kDa	2
Catalase OS=Mus musculus OX=10090 GN=Cat PE=1 SV=4	60 kDa	2
Catechol O-methyltransferase OS=Mus musculus OX=10090 GN=Comt PE=1 SV=2	29 kDa	2
Catenin alpha-1 OS=Mus musculus OX=10090 GN=Ctnna1 PE=1 SV=1	100 kDa	6
Catenin alpha-2 OS=Mus musculus OX=10090 GN=Ctnna2 PE=1 SV=3	105 kDa	1
Catenin beta-1 OS=Mus musculus OX=10090 GN=Ctnnb1 PE=1 SV=1	85 kDa	1
Catenin delta-1 OS=Mus musculus OX=10090 GN=Ctnnd1 PE=1 SV=2	105 kDa	1
Cathepsin B OS=Mus musculus OX=10090 GN=Ctsb PE=1 SV=2	37 kDa	51
Cathepsin D OS=Mus musculus OX=10090 GN=Ctsd PE=1 SV=1	45 kDa	7

Cathepsin L1 OS=Mus musculus OX=10090 GN=Ctsl PE=1 SV=2	38 kDa	19
Cathepsin Z OS=Mus musculus OX=10090 GN=Ctsz PE=1 SV=1	34 kDa	13
Cation-dependent mannose-6-phosphate receptor OS=Mus musculus OX=10090 GN=M6pr PE=1 SV=1	31 kDa	11
Cationic trypsin OS=Bos taurus PE=1 SV=3	26 kDa	45
Cation-independent mannose-6-phosphate receptor OS=Mus musculus OX=10090 GN=Igf2r PE=1 SV=1	274 kDa	31
Caveolae-associated protein 1 OS=Mus musculus OX=10090 GN=Cavin1 PE=1 SV=1	44 kDa	46
Caveolae-associated protein 2 OS=Mus musculus OX=10090 GN=Cavin2 PE=1 SV=3	47 kDa	10
Caveolin-1 OS=Mus musculus OX=10090 GN=Cav1 PE=1 SV=1	21 kDa	10
Caveolin-2 OS=Mus musculus OX=10090 GN=Cav2 PE=1 SV=1	18 kDa	2
Cbx3 protein OS=Mus musculus OX=10090 GN=Cbx3 PE=1 SV=1	21 kDa	28
CCR4-NOT transcription complex subunit 1 OS=Mus musculus OX=10090 GN=Cnot1 PE=1 SV=1	180 kDa	1
CCR4-NOT transcription complex subunit 3 OS=Mus musculus OX=10090 GN=Cnot3 PE=1 SV=1	82 kDa	1
CCR4-NOT transcription complex subunit 9 OS=Mus musculus OX=10090 GN=Cnot9 PE=1 SV=1	34 kDa	1
CD109 antigen OS=Mus musculus OX=10090 GN=Cd109 PE=1 SV=1	162 kDa	8
CD151 antigen OS=Mus musculus OX=10090 GN=Cd151 PE=1 SV=2	28 kDa	3
CD166 antigen OS=Mus musculus OX=10090 GN=Alcam PE=1 SV=3	65 kDa	6
CD2 antigen cytoplasmic tail-binding protein 2 OS=Mus musculus OX=10090 GN=Cd2bp2 PE=1 SV=1	38 kDa	2
CD276 antigen OS=Mus musculus OX=10090 GN=Cd276 PE=1 SV=1	34 kDa	2
CD2-associated protein OS=Mus musculus OX=10090 GN=Cd2ap PE=1 SV=3	70 kDa	4
CD44 antigen OS=Mus musculus OX=10090 GN=Cd44 PE=1 SV=1	72 kDa	31
CD81 antigen OS=Mus musculus OX=10090 GN=Cd81 PE=1 SV=2	26 kDa	1
CD9 antigen OS=Mus musculus OX=10090 GN=Cd9 PE=1 SV=2	25 kDa	5
CDGSH iron sulfur domain 3 OS=Mus musculus OX=10090 GN=Cisd3 PE=1 SV=1	15 kDa	1
CDGSH iron-sulfur domain-containing protein 1 OS=Mus musculus OX=10090 GN=Cisd1 PE=1 SV=1	12 kDa	3
CDGSH iron-sulfur domain-containing protein 2 OS=Mus musculus OX=10090 GN=Cisd2 PE=1 SV=1	15 kDa	1

CDK-activating kinase assembly factor MAT1 OS=Mus musculus OX=10090 GN=Mnat1 PE=1 SV=2	36 kDa	4
CDKN2A-interacting protein OS=Mus musculus OX=10090 GN=Cdkn2aip PE=1 SV=1	60 kDa	1
Cell adhesion molecule 1 OS=Mus musculus OX=10090 GN=Cadm1 PE=1 SV=2	50 kDa	1
Cell cycle and apoptosis regulator protein 2 OS=Mus musculus OX=10090 GN=Ccar2 PE=1 SV=2	103 kDa	3
Cell cycle checkpoint control protein RAD9A OS=Mus musculus OX=10090 GN=Rad9a PE=1 SV=1	42 kDa	1
Cell cycle control protein 50B OS=Mus musculus OX=10090 GN=Tmem30b PE=2 SV=1	39 kDa	1
Cell division control protein 42 homolog OS=Mus musculus OX=10090 GN=Cdc42 PE=1 SV=2	21 kDa	27
Cell division cycle 5-like protein OS=Mus musculus OX=10090 GN=Cdc5l PE=1 SV=2	92 kDa	2
Cell division cycle and apoptosis regulator protein 1 OS=Mus musculus OX=10090 GN=Ccar1 PE=1 SV=1	132 kDa	7
Cell division cycle protein 20 homolog OS=Mus musculus OX=10090 GN=Cdc20 PE=1 SV=2	55 kDa	3
Cell growth-regulating nucleolar protein OS=Mus musculus OX=10090 GN=Lyar PE=1 SV=2	44 kDa	4
Cellular nucleic acid-binding protein OS=Mus musculus OX=10090 GN=Cnbp PE=1 SV=1	19 kDa	22
Cellular nucleic acid-binding protein OS=Mus musculus OX=10090 GN=Cnbp PE=1 SV=2	20 kDa	20
Centromere protein V OS=Mus musculus OX=10090 GN=Cenpv PE=1 SV=2	28 kDa	2
Centromere/kinetochore protein zw10 homolog OS=Mus musculus OX=10090 GN=Zw10 PE=1 SV=3	88 kDa	1
Centrosomal protein of 170 kDa OS=Mus musculus OX=10090 GN=Cep170 PE=1 SV=2	175 kDa	5
Cerebellar degeneration-related protein 2-like OS=Mus musculus OX=10090 GN=Cdr2l PE=1 SV=1	53 kDa	1
CGG triplet repeat-binding protein 1 OS=Mus musculus OX=10090 GN=Cggbp1 PE=1 SV=1	19 kDa	2
Charged multivesicular body protein 1a OS=Mus musculus OX=10090 GN=Chmp1a PE=1 SV=1	22 kDa	2
Charged multivesicular body protein 2a OS=Mus musculus OX=10090 GN=Chmp2a PE=1 SV=1	25 kDa	1
Charged multivesicular body protein 2b OS=Mus musculus OX=10090 GN=Chmp2b PE=1 SV=1	24 kDa	2
Charged multivesicular body protein 4b OS=Mus musculus OX=10090 GN=Chmp4b PE=1 SV=2	25 kDa	11
Charged multivesicular body protein 5 OS=Mus musculus OX=10090 GN=Chmp5 PE=1 SV=1	25 kDa	6
Charged multivesicular body protein 6 (Fragment) OS=Mus musculus OX=10090 GN=Chmp6 PE=1 SV=1	4 kDa	1
Chloride channel, nucleotide-sensitive, 1A OS=Mus musculus OX=10090 GN=Clns1a PE=1 SV=1	27 kDa	9

Chloride intracellular channel protein 1 OS=Mus musculus OX=10090 GN=Clic1 PE=1 SV=3	27 kDa	16
Chloride intracellular channel protein 4 OS=Mus musculus OX=10090 GN=Clic4 PE=1 SV=3	29 kDa	1
Choline-phosphate cytidylyltransferase A OS=Mus musculus OX=10090 GN=Pcyt1a PE=1 SV=1	42 kDa	2
Choline-phosphate cytidylyltransferase B OS=Mus musculus OX=10090 GN=Pcyt1b PE=1 SV=2	42 kDa	1
Chromatin accessibility complex protein 1 OS=Mus musculus OX=10090 GN=Chrac1 PE=1 SV=1	14 kDa	1
Chromatin assembly factor 1 subunit B OS=Mus musculus OX=10090 GN=Chaf1b PE=1 SV=1	63 kDa	2
Chromatin target of PRMT1 protein OS=Mus musculus OX=10090 GN=Chtop PE=1 SV=1	22 kDa	1
Chromobox protein homolog 1 OS=Mus musculus OX=10090 GN=Cbx1 PE=1 SV=1	21 kDa	6
Chromobox protein homolog 5 OS=Mus musculus OX=10090 GN=Cbx5 PE=1 SV=1	22 kDa	6
Chromodomain-helicase-DNA-binding protein 4 OS=Mus musculus OX=10090 GN=Chd4 PE=1 SV=1	218 kDa	10
Chromodomain-helicase-DNA-binding protein 5 OS=Mus musculus OX=10090 GN=Chd5 PE=1 SV=1	223 kDa	3
Chromosome-associated kinesin KIF4 OS=Mus musculus OX=10090 GN=Kif4 PE=1 SV=3	140 kDa	1
Chymotrypsinogen A OS=Bos taurus PE=1 SV=1	26 kDa	2
Cilia- and flagella-associated protein 20 OS=Mus musculus OX=10090 GN=Cfap20 PE=1 SV=1	23 kDa	2
Citramalyl-CoA lyase, mitochondrial OS=Mus musculus OX=10090 GN=Clybl PE=1 SV=2	38 kDa	1
Citrate synthase, mitochondrial OS=Mus musculus OX=10090 GN=Cs PE=1 SV=1	52 kDa	42
C-Jun-amino-terminal kinase-interacting protein 4 OS=Mus musculus OX=10090 GN=Spag9 PE=1 SV=2	146 kDa	11
Clathrin coat assembly protein AP180 OS=Mus musculus OX=10090 GN=Snap91 PE=1 SV=2	86 kDa	1
Clathrin heavy chain 1 OS=Mus musculus OX=10090 GN=Cltc PE=1 SV=3	192 kDa	8
Clathrin interactor 1 OS=Mus musculus OX=10090 GN=Clint1 PE=1 SV=1	70 kDa	7
Clathrin light chain OS=Mus musculus OX=10090 GN=Clta PE=1 SV=1	23 kDa	13
Cleavage and polyadenylation specificity factor subunit 1 OS=Mus musculus OX=10090 GN=Cpsf1 PE=1 SV=1	161 kDa	2
Cleavage and polyadenylation specificity factor subunit 2 OS=Mus musculus OX=10090 GN=Cpsf2 PE=1 SV=1	88 kDa	1
Cleavage and polyadenylation specificity factor subunit 3 OS=Mus musculus OX=10090 GN=Cpsf3 PE=1 SV=2	78 kDa	2
Cleavage and polyadenylation specificity factor subunit 5 OS=Mus musculus OX=10090 GN=Nudt21 PE=1 SV=1	26 kDa	9

Cleavage and polyadenylation specificity factor subunit 6 OS=Mus musculus OX=10090 GN=Cpsf6 PE=1 SV=1	59 kDa	4
Cleavage and polyadenylation specificity factor subunit 7 OS=Mus musculus OX=10090 GN=Cpsf7 PE=1 SV=2	52 kDa	3
Cleavage stimulation factor subunit 1 OS=Mus musculus OX=10090 GN=Cstf1 PE=1 SV=1	48 kDa	3
Cleavage stimulation factor subunit 2 tau variant OS=Mus musculus OX=10090 GN=Cstf2t PE=1 SV=2	66 kDa	1
Clustered mitochondria protein homolog OS=Mus musculus OX=10090 GN=Cluh PE=1 SV=1	152 kDa	6
Clusterin OS=Mus musculus OX=10090 GN=Clu PE=1 SV=1	52 kDa	1
c-Myc-binding protein OS=Mus musculus OX=10090 GN=Mycbp PE=1 SV=5	12 kDa	2
Coatomer subunit alpha OS=Mus musculus OX=10090 GN=Copa PE=1 SV=2	138 kDa	21
Coatomer subunit beta OS=Mus musculus OX=10090 GN=Copb1 PE=1 SV=1	107 kDa	18
Coatomer subunit beta' OS=Mus musculus OX=10090 GN=Copb2 PE=1 SV=2	102 kDa	27
Coatomer subunit delta OS=Mus musculus OX=10090 GN=Arcn1 PE=1 SV=2	57 kDa	23
Coatomer subunit epsilon OS=Mus musculus OX=10090 GN=Cope PE=1 SV=3	35 kDa	2
Coatomer subunit gamma-1 OS=Mus musculus OX=10090 GN=Copg1 PE=1 SV=1	98 kDa	21
Coatomer subunit gamma-2 OS=Mus musculus OX=10090 GN=Copg2 PE=1 SV=1	98 kDa	3
Coatomer subunit zeta-1 OS=Mus musculus OX=10090 GN=Copz1 PE=1 SV=1	20 kDa	3
COBW domain-containing protein 1 OS=Mus musculus OX=10090 GN=Cbwd1 PE=1 SV=1	44 kDa	1
Cofilin-1 OS=Mus musculus OX=10090 GN=Cfl1 PE=1 SV=3	19 kDa	104
Cofilin-2 OS=Mus musculus OX=10090 GN=Cfl2 PE=1 SV=1	19 kDa	15
Coiled-coil domain-containing protein 115 OS=Mus musculus OX=10090 GN=Ccdc115 PE=1 SV=1	20 kDa	1
Coiled-coil domain-containing protein 124 OS=Mus musculus OX=10090 GN=Ccdc124 PE=1 SV=1	25 kDa	2
Coiled-coil domain-containing protein 189 (Fragment) OS=Mus musculus OX=10090 GN=Ccdc189 PE=4 SV=1	18 kDa	1
Coiled-coil domain-containing protein 43 OS=Mus musculus OX=10090 GN=Ccdc43 PE=1 SV=1	25 kDa	2
Coiled-coil domain-containing protein 47 OS=Mus musculus OX=10090 GN=Ccdc47 PE=1 SV=2	56 kDa	3
Coiled-coil domain-containing protein 50 OS=Mus musculus OX=10090 GN=Ccdc50 PE=1 SV=1	35 kDa	2
Coiled-coil domain-containing protein 51 OS=Mus musculus OX=10090 GN=Ccdc51 PE=1 SV=1	45 kDa	1

Coiled-coil domain-containing protein 58 OS=Mus musculus OX=10090 GN=Ccdc58 PE=1 SV=1	16 kDa	3
Coiled-coil domain-containing protein 58 OS=Mus musculus OX=10090 GN=Ccdc58 PE=1 SV=1	17 kDa	4
Coiled-coil domain-containing protein 6 OS=Mus musculus OX=10090 GN=Ccdc6 PE=1 SV=1	53 kDa	3
Coiled-coil domain-containing protein 90B, mitochondrial OS=Mus musculus OX=10090 GN=Ccdc90b PE=1 SV=1	30 kDa	1
Coiled-coil-helix-coiled-coil-helix domain-containing protein 7 OS=Mus musculus OX=10090 GN=Chchd7 PE=1 SV=1	7 kDa	1
Cold shock domain-containing protein E1 OS=Mus musculus OX=10090 GN=Csde1 PE=1 SV=1	89 kDa	4
Collagen alpha-1(III) chain OS=Mus musculus OX=10090 GN=Col3a1 PE=1 SV=4	139 kDa	3
Collagen alpha-1(IV) chain OS=Mus musculus OX=10090 GN=Col4a1 PE=1 SV=1	149 kDa	1
Collagen alpha-2(IV) chain OS=Mus musculus OX=10090 GN=Col4a2 PE=1 SV=4	167 kDa	1
Collagen triple helix repeat-containing protein 1 OS=Mus musculus OX=10090 GN=Cthrc1 PE=2 SV=2	26 kDa	1
Collectin-12 OS=Mus musculus OX=10090 GN=Colec12 PE=1 SV=1	81 kDa	1
COMM domain-containing protein 1 OS=Mus musculus OX=10090 GN=Commd1 PE=1 SV=2	21 kDa	1
Complement component 1 Q subcomponent-binding protein, mitochondrial OS=Mus musculus OX=10090 GN=C1qbp PE=1 SV=1	31 kDa	28
Condensin complex subunit 2 OS=Mus musculus OX=10090 GN=Ncaph PE=1 SV=1	82 kDa	1
Constitutive coactivator of PPAR-gamma-like protein 1 OS=Mus musculus OX=10090 GN=FAM120A PE=1 SV=2	122 kDa	3
COP9 signalosome complex subunit 1 OS=Mus musculus OX=10090 GN=Gps1 PE=1 SV=1	53 kDa	10
COP9 signalosome complex subunit 1 OS=Mus musculus OX=10090 GN=Gps1 PE=1 SV=1	55 kDa	10
COP9 signalosome complex subunit 2 OS=Mus musculus OX=10090 GN=Cops2 PE=1 SV=1	52 kDa	15
COP9 signalosome complex subunit 3 OS=Mus musculus OX=10090 GN=Cops3 PE=1 SV=3	48 kDa	5
COP9 signalosome complex subunit 4 OS=Mus musculus OX=10090 GN=Cops4 PE=1 SV=1	46 kDa	15
COP9 signalosome complex subunit 5 OS=Mus musculus OX=10090 GN=Cops5 PE=1 SV=3	38 kDa	6
COP9 signalosome complex subunit 6 OS=Mus musculus OX=10090 GN=Cops6 PE=1 SV=1	36 kDa	7
COP9 signalosome complex subunit 7a OS=Mus musculus OX=10090 GN=Cops7a PE=1 SV=2	30 kDa	8
COP9 signalosome complex subunit 7b OS=Mus musculus OX=10090 GN=Cops7b PE=1 SV=1	30 kDa	2

COP9 signalosome complex subunit 8 OS=Mus musculus OX=10090 GN=Cops8 PE=1 SV=1	23 kDa	6
Copine-1 OS=Mus musculus OX=10090 GN=Cpne1 PE=1 SV=1	59 kDa	6
Copine-2 OS=Mus musculus OX=10090 GN=Cpne2 PE=1 SV=1	61 kDa	1
Copine-3 OS=Mus musculus OX=10090 GN=Cpne3 PE=1 SV=2	60 kDa	1
Copine-5 OS=Mus musculus OX=10090 GN=Cpne5 PE=1 SV=1	66 kDa	1
Copper chaperone for superoxide dismutase OS=Mus musculus OX=10090 GN=Ccs PE=1 SV=1	29 kDa	1
Copper transport protein ATOX1 OS=Mus musculus OX=10090 GN=Atox1 PE=1 SV=1	7 kDa	7
Cordon-bleu protein-like 1 OS=Mus musculus OX=10090 GN=Cobll1 PE=1 SV=2	137 kDa	11
Core histone macro-H2A.1 OS=Mus musculus OX=10090 GN=H2afy PE=1 SV=3	40 kDa	3
Core-binding factor subunit beta OS=Mus musculus OX=10090 GN=Cbfb PE=1 SV=1	22 kDa	2
Coronin-1B OS=Mus musculus OX=10090 GN=Coro1b PE=1 SV=1	54 kDa	6
Coronin-1C OS=Mus musculus OX=10090 GN=Coro1c PE=1 SV=2	53 kDa	29
Costars family protein ABRACL (Fragment) OS=Mus musculus OX=10090 GN=Abracl PE=1 SV=1	6 kDa	3
COX assembly mitochondrial protein 2 homolog OS=Mus musculus OX=10090 GN=Cmc2 PE=3 SV=1	9 kDa	2
COX assembly mitochondrial protein homolog OS=Mus musculus OX=10090 GN=Cmc1 PE=1 SV=1	13 kDa	1
Craniofacial development protein 1 OS=Mus musculus OX=10090 GN=Cfdp1 PE=1 SV=1	33 kDa	10
CREB-binding protein OS=Mus musculus OX=10090 GN=Crebbp PE=1 SV=3	265 kDa	1
Crk-like protein OS=Mus musculus OX=10090 GN=Crkl PE=1 SV=2	34 kDa	6
C-terminal-binding protein 2 OS=Mus musculus OX=10090 GN=Ctbp2 PE=1 SV=2	49 kDa	2
CTP synthase 1 OS=Mus musculus OX=10090 GN=Ctps1 PE=1 SV=2	67 kDa	7
CTP synthase 2 OS=Mus musculus OX=10090 GN=Ctps2 PE=1 SV=1	66 kDa	1
CTTNBP2 N-terminal-like protein OS=Mus musculus OX=10090 GN=Cttnbp2nl PE=1 SV=1	70 kDa	1
C-type mannose receptor 2 OS=Mus musculus OX=10090 GN=Mrc2 PE=1 SV=3	167 kDa	1
Cullin-1 OS=Mus musculus OX=10090 GN=Cul1 PE=1 SV=1	90 kDa	8
Cullin-3 OS=Mus musculus OX=10090 GN=Cul3 PE=1 SV=1	89 kDa	1

Cullin-4B OS=Mus musculus OX=10090 GN=Cul4b PE=1 SV=1	111 kDa	2
CXADR-like membrane protein OS=Mus musculus OX=10090 GN=Clmp PE=1 SV=1	41 kDa	1
Cyclic AMP-dependent transcription factor ATF-1 OS=Mus musculus OX=10090 GN=Atf1 PE=1 SV=1	29 kDa	1
Cyclic AMP-dependent transcription factor ATF-7 OS=Mus musculus OX=10090 GN=Atf7 PE=2 SV=1	45 kDa	1
Cyclic AMP-responsive element-binding protein 1 OS=Mus musculus OX=10090 GN=Creb1 PE=1 SV=1	37 kDa	3
Cyclin-A2 OS=Mus musculus OX=10090 GN=Ccna2 PE=1 SV=2	47 kDa	1
Cyclin-dependent kinase 1 OS=Mus musculus OX=10090 GN=Cdk1 PE=1 SV=3	34 kDa	5
Cyclin-dependent kinase 12 OS=Mus musculus OX=10090 GN=Cdk12 PE=1 SV=2	164 kDa	2
Cyclin-dependent kinase 13 OS=Mus musculus OX=10090 GN=Cdk13 PE=1 SV=3	165 kDa	2
Cyclin-dependent kinase 16 OS=Mus musculus OX=10090 GN=Cdk16 PE=1 SV=1	56 kDa	2
Cyclin-dependent kinase 2 OS=Mus musculus OX=10090 GN=Cdk2 PE=1 SV=2	39 kDa	3
Cyclin-dependent kinase 6 OS=Mus musculus OX=10090 GN=Cdk6 PE=1 SV=2	37 kDa	4
Cyclin-dependent kinase 9 OS=Mus musculus OX=10090 GN=Cdk9 PE=1 SV=1	43 kDa	3
Cyclin-dependent kinase inhibitor 1 OS=Mus musculus OX=10090 GN=Cdkn1a PE=1 SV=4	18 kDa	1
Cyclin-dependent kinases regulatory subunit 1 OS=Mus musculus OX=10090 GN=Cks1b PE=3 SV=1	10 kDa	4
Cyclin-dependent kinases regulatory subunit OS=Mus musculus OX=10090 GN=Cks1brt PE=2 SV=1	12 kDa	3
Cyclin-K OS=Mus musculus OX=10090 GN=Ccnk PE=1 SV=3	61 kDa	2
Cystathionine gamma-lyase OS=Mus musculus OX=10090 GN=Cth PE=1 SV=1	44 kDa	1
Cystatin (Fragment) OS=Mus musculus OX=10090 GN=Cst3 PE=1 SV=1	11 kDa	1
Cystatin-B OS=Mus musculus OX=10090 GN=Cstb PE=1 SV=1	11 kDa	16
Cysteine and glycine-rich protein 1 OS=Mus musculus OX=10090 GN=Csrp1 PE=1 SV=3	21 kDa	13
Cysteine and histidine-rich domain-containing protein 1 OS=Mus musculus OX=10090 GN=Chordc1 PE=1 SV=1	37 kDa	11
Cysteine sulfinic acid decarboxylase OS=Mus musculus OX=10090 GN=Csad PE=1 SV=1	55 kDa	1
Cysteine-rich with EGF-like domain protein 2 OS=Mus musculus OX=10090 GN=Creld2 PE=1 SV=1	38 kDa	5
Cysteine--tRNA ligase, cytoplasmic OS=Mus musculus OX=10090 GN=Cars PE=1 SV=2	95 kDa	9

Cytidine deaminase OS=Mus musculus OX=10090 GN=Cda PE=1 SV=2	16 kDa	8
Cytochrome b5 OS=Mus musculus OX=10090 GN=Cyb5a PE=1 SV=2	15 kDa	4
Cytochrome b5 reductase 1, isoform CRA_a (Fragment) OS=Mus musculus OX=10090 GN=Cyb5r1 PE=1 SV=1	19 kDa	3
Cytochrome b5 type B OS=Mus musculus OX=10090 GN=Cyb5b PE=1 SV=1	16 kDa	8
Cytochrome b-c1 complex subunit 1, mitochondrial OS=Mus musculus OX=10090 GN=Uqcrc1 PE=1 SV=2	53 kDa	2
Cytochrome b-c1 complex subunit 2, mitochondrial OS=Mus musculus OX=10090 GN=Uqcrc2 PE=1 SV=1	48 kDa	1
Cytochrome b-c1 complex subunit 6, mitochondrial OS=Mus musculus OX=10090 GN=Uqcrh PE=1 SV=2	10 kDa	2
Cytochrome b-c1 complex subunit 7 OS=Mus musculus OX=10090 GN=Uqcrb PE=1 SV=1	14 kDa	3
Cytochrome b-c1 complex subunit 8 OS=Mus musculus OX=10090 GN=Uqcrq PE=1 SV=3	10 kDa	1
Cytochrome b-c1 complex subunit 9 OS=Mus musculus OX=10090 GN=Uqcr10 PE=1 SV=1	7 kDa	1
Cytochrome b-c1 complex subunit Rieske, mitochondrial OS=Mus musculus OX=10090 GN=Uqcrfs1 PE=1 SV=1	29 kDa	7
Cytochrome c oxidase assembly factor 3 homolog, mitochondrial OS=Mus musculus OX=10090 GN=Coa3 PE=1 SV=1	12 kDa	2
Cytochrome c oxidase assembly factor 6 homolog OS=Mus musculus OX=10090 GN=Coa6 PE=1 SV=1	9 kDa	2
Cytochrome c oxidase assembly factor 7 OS=Mus musculus OX=10090 GN=Coa7 PE=1 SV=1	26 kDa	1
Cytochrome c oxidase assembly protein COX20, mitochondrial OS=Mus musculus OX=10090 GN=Cox20 PE=1 SV=1	13 kDa	1
Cytochrome c oxidase subunit 2 OS=Mus musculus OX=10090 GN=Mtco2 PE=1 SV=1	26 kDa	4
Cytochrome c oxidase subunit 4 isoform 1, mitochondrial OS=Mus musculus OX=10090 GN=Cox4i1 PE=1 SV=2	20 kDa	24
Cytochrome c oxidase subunit 5A, mitochondrial OS=Mus musculus OX=10090 GN=Cox5a PE=1 SV=2	16 kDa	7
Cytochrome c oxidase subunit 5B, mitochondrial OS=Mus musculus OX=10090 GN=Cox5b PE=1 SV=1	14 kDa	10
Cytochrome c oxidase subunit 6B1 OS=Mus musculus OX=10090 GN=Cox6b1 PE=1 SV=2	10 kDa	12
Cytochrome c oxidase subunit 6C OS=Mus musculus OX=10090 GN=Cox6c PE=1 SV=3	8 kDa	9
Cytochrome c oxidase subunit NDUFA4 OS=Mus musculus OX=10090 GN=Ndufa4 PE=1 SV=1	6 kDa	4
Cytochrome c, somatic OS=Mus musculus OX=10090 GN=Cycs PE=1 SV=2	12 kDa	27
Cytoplasmic aconitate hydratase OS=Mus musculus OX=10090 GN=Aco1 PE=1 SV=3	98 kDa	18

Cytoplasmic dynein 1 intermediate chain 2 OS=Mus musculus OX=10090 GN=Dync1i2 PE=1 SV=1	71 kDa	15
Cytoplasmic dynein 1 light intermediate chain 2 (Fragment) OS=Mus musculus OX=10090 GN=Dync1li2 PE=1 SV=1	45 kDa	2
Cytoplasmic tRNA 2-thiolation protein 2 OS=Mus musculus OX=10090 GN=Ctu2 PE=1 SV=1	56 kDa	1
Cytoskeleton-associated protein 4 OS=Mus musculus OX=10090 GN=Ckap4 PE=1 SV=2	64 kDa	59
Cytoskeleton-associated protein 5 OS=Mus musculus OX=10090 GN=Ckap5 PE=1 SV=1	224 kDa	1
Cytosol aminopeptidase OS=Mus musculus OX=10090 GN=Lap3 PE=1 SV=3	56 kDa	16
Cytosolic acyl coenzyme A thioester hydrolase OS=Mus musculus OX=10090 GN=Acot7 PE=1 SV=2	43 kDa	16
Cytosolic Fe-S cluster assembly factor NUBP1 OS=Mus musculus OX=10090 GN=Nubp1 PE=1 SV=1	34 kDa	6
Cytosolic non-specific dipeptidase OS=Mus musculus OX=10090 GN=Cndp2 PE=1 SV=1	53 kDa	12
D-3-phosphoglycerate dehydrogenase OS=Mus musculus OX=10090 GN=Phgdh PE=1 SV=3	57 kDa	40
D-aminoacyl-tRNA deacylase 1 OS=Mus musculus OX=10090 GN=Dtd1 PE=1 SV=2	23 kDa	3
DAZ-associated protein 1 OS=Mus musculus OX=10090 GN=Dazap1 PE=1 SV=2	43 kDa	11
DCN1-like protein 5 OS=Mus musculus OX=10090 GN=Dcun1d5 PE=1 SV=1	28 kDa	1
DDB1- and CUL4-associated factor 13 OS=Mus musculus OX=10090 GN=Dcaf13 PE=2 SV=2	51 kDa	1
DDB1- and CUL4-associated factor 7 OS=Mus musculus OX=10090 GN=Dcaf7 PE=1 SV=1	39 kDa	2
DDB1- and CUL4-associated factor 8 OS=Mus musculus OX=10090 GN=Dcaf8 PE=1 SV=1	66 kDa	9
D-dopachrome decarboxylase OS=Mus musculus OX=10090 GN=Ddt PE=1 SV=3	13 kDa	4
DDRGK domain-containing protein 1 OS=Mus musculus OX=10090 GN=Ddrgk1 PE=1 SV=2	36 kDa	3
Deaminated glutathione amidase OS=Mus musculus OX=10090 GN=Nit1 PE=1 SV=2	36 kDa	4
Death-associated protein 1 OS=Mus musculus OX=10090 GN=Dap PE=1 SV=3	11 kDa	9
Delta(3,5)-Delta(2,4)-dienoyl-CoA isomerase, mitochondrial (Fragment) OS=Mus musculus OX=10090 GN=Ech1 PE=1 SV=1	19 kDa	2
Delta(3,5)-Delta(2,4)-dienoyl-CoA isomerase, mitochondrial OS=Mus musculus OX=10090 GN=Ech1 PE=1 SV=1	36 kDa	2
Delta-1-pyrroline-5-carboxylate synthase OS=Mus musculus OX=10090 GN=Aldh18a1 PE=1 SV=2	87 kDa	8
Delta-aminolevulinic acid dehydratase OS=Mus musculus OX=10090 GN=Alad PE=1 SV=1	36 kDa	20

Density-regulated protein OS=Mus musculus OX=10090 GN=Denr PE=1 SV=1	22 kDa	9
Deoxycytidine kinase OS=Mus musculus OX=10090 GN=Dck PE=1 SV=1	30 kDa	1
Deoxycytidylate deaminase OS=Mus musculus OX=10090 GN=Dctd PE=2 SV=1	20 kDa	1
Deoxyguanosine kinase, mitochondrial (Fragment) OS=Mus musculus OX=10090 GN=Dguok PE=1 SV=1	24 kDa	1
Deoxyhypusine hydroxylase OS=Mus musculus OX=10090 GN=Dohh PE=1 SV=2	33 kDa	1
Deoxyhypusine synthase OS=Mus musculus OX=10090 GN=Dhps PE=1 SV=2	41 kDa	1
Deoxynucleoside triphosphate triphosphohydrolase SAMHD1 OS=Mus musculus OX=10090 GN=Samhd1 PE=1 SV=1	76 kDa	18
Deoxynucleoside triphosphate triphosphohydrolase SAMHD1 OS=Mus musculus OX=10090 GN=Samhd1 PE=1 SV=2	73 kDa	19
Deoxynucleotidyltransferase terminal-interacting protein 2 OS=Mus musculus OX=10090 GN=Dnttip2 PE=1 SV=1	84 kDa	1
Deoxyribose-phosphate aldolase (Fragment) OS=Mus musculus OX=10090 GN=Dera PE=1 SV=1	11 kDa	1
Deoxyuridine triphosphatase OS=Mus musculus OX=10090 GN=Dut PE=1 SV=1	17 kDa	19
Deoxyuridine triphosphatase OS=Mus musculus OX=10090 GN=Dut PE=1 SV=1	21 kDa	18
Destrin OS=Mus musculus OX=10090 GN=Dstn PE=1 SV=3	19 kDa	23
DET1- and DDB1-associated protein 1 OS=Mus musculus OX=10090 GN=Dda1 PE=1 SV=1	11 kDa	1
Developmentally-regulated GTP-binding protein 1 OS=Mus musculus OX=10090 GN=Drg1 PE=1 SV=1	41 kDa	2
Developmentally-regulated GTP-binding protein 2 OS=Mus musculus OX=10090 GN=Drg2 PE=1 SV=1	41 kDa	2
D-glutamate cyclase, mitochondrial OS=Mus musculus OX=10090 GN=Dglucy PE=1 SV=1	70 kDa	15
Digestive organ expansion factor homolog OS=Mus musculus OX=10090 GN=Diexf PE=1 SV=1	73 kDa	1
Dihydrofolate reductase OS=Mus musculus OX=10090 GN=Dhfr PE=1 SV=3	22 kDa	2
Dihydrolipoyl dehydrogenase, mitochondrial OS=Mus musculus OX=10090 GN=Dld PE=1 SV=2	54 kDa	46
Dihydrolipoyllysine-residue acetyltransferase component of pyruvate dehydrogenase complex, mitochondrial OS=Mus musculus OX=10090 GN=Dlat PE=1 SV=2	68 kDa	14
Dihydrolipoyllysine-residue succinyltransferase component of 2-oxoglutarate dehydrogenase complex, mitochondrial OS=Mus musculus OX=10090 GN=Dlst PE=1 SV=1	49 kDa	6
Dihydroorotate dehydrogenase (quinone), mitochondrial (Fragment) OS=Mus musculus OX=10090 GN=Dhodh PE=1 SV=1	11 kDa	2

Dihydropteridine reductase OS=Mus musculus OX=10090 GN=Qdpr PE=1 SV=1	22 kDa	2
Dihydropyrimidinase-related protein 2 OS=Mus musculus OX=10090 GN=Dpysl2 PE=1 SV=2	62 kDa	84
Dihydropyrimidinase-related protein 3 OS=Mus musculus OX=10090 GN=Dpysl3 PE=1 SV=1	74 kDa	50
Dipeptidyl peptidase 1 OS=Mus musculus OX=10090 GN=Ctsc PE=1 SV=1	52 kDa	2
Dipeptidyl peptidase 2 OS=Mus musculus OX=10090 GN=Dpp7 PE=1 SV=2	56 kDa	4
Dipeptidyl peptidase 3 OS=Mus musculus OX=10090 GN=Dpp3 PE=1 SV=2	83 kDa	6
Dipeptidyl peptidase 9 OS=Mus musculus OX=10090 GN=Dpp9 PE=1 SV=2	98 kDa	3
Diphosphoinositol polyphosphate phosphohydrolase 1 OS=Mus musculus OX=10090 GN=Nudt3 PE=1 SV=1	16 kDa	2
Diphosphomevalonate decarboxylase OS=Mus musculus OX=10090 GN=Mvd PE=1 SV=2	44 kDa	1
Diphthine methyltransferase OS=Mus musculus OX=10090 GN=Dph7 PE=2 SV=1	53 kDa	1
Disabled homolog 2 OS=Mus musculus OX=10090 GN=Dab2 PE=1 SV=1	82 kDa	6
Disintegrin and metalloproteinase domain-containing protein 10 OS=Mus musculus OX=10090 GN=Adam10 PE=1 SV=2	84 kDa	2
Disintegrin and metalloproteinase domain-containing protein 17 OS=Mus musculus OX=10090 GN=Adam17 PE=1 SV=3	93 kDa	2
Disintegrin and metalloproteinase domain-containing protein 9 OS=Mus musculus OX=10090 GN=Adam9 PE=1 SV=1	94 kDa	2
Disks large homolog 1 OS=Mus musculus OX=10090 GN=Dlg1 PE=1 SV=1	92 kDa	3
DNA (cytosine-5)-methyltransferase 1 OS=Mus musculus OX=10090 GN=Dnmt1 PE=1 SV=5	183 kDa	3
DNA damage-binding protein 1 OS=Mus musculus OX=10090 GN=Ddb1 PE=1 SV=2	127 kDa	68
DNA ligase 1 OS=Mus musculus OX=10090 GN=Lig1 PE=1 SV=2	102 kDa	4
DNA ligase OS=Mus musculus OX=10090 GN=Lig3 PE=1 SV=1	106 kDa	1
DNA mismatch repair protein Msh2 OS=Mus musculus OX=10090 GN=Msh2 PE=1 SV=1	104 kDa	3
DNA mismatch repair protein Msh6 OS=Mus musculus OX=10090 GN=Msh6 PE=1 SV=3	151 kDa	4
DNA polymerase epsilon subunit 3 OS=Mus musculus OX=10090 GN=Pole3 PE=1 SV=1	17 kDa	1
DNA repair protein RAD50 OS=Mus musculus OX=10090 GN=Rad50 PE=1 SV=1	154 kDa	1
DNA repair protein XRCC1 OS=Mus musculus OX=10090 GN=Xrcc1 PE=1 SV=2	69 kDa	1
DNA replication complex GINS protein PSF1 OS=Mus musculus OX=10090 GN=Gins1 PE=1 SV=1	23 kDa	1

DNA replication licensing factor MCM2 OS=Mus musculus OX=10090 GN=Mcm2 PE=1 SV=3	102 kDa	23
DNA replication licensing factor MCM3 OS=Mus musculus OX=10090 GN=Mcm3 PE=1 SV=2	92 kDa	19
DNA replication licensing factor MCM4 OS=Mus musculus OX=10090 GN=Mcm4 PE=1 SV=1	97 kDa	13
DNA replication licensing factor MCM5 OS=Mus musculus OX=10090 GN=Mcm5 PE=1 SV=1	82 kDa	11
DNA replication licensing factor MCM6 OS=Mus musculus OX=10090 GN=Mcm6 PE=1 SV=1	93 kDa	19
DNA replication licensing factor MCM7 OS=Mus musculus OX=10090 GN=Mcm7 PE=1 SV=1	81 kDa	14
DNA topoisomerase 1 OS=Mus musculus OX=10090 GN=Top1 PE=1 SV=2	91 kDa	5
DNA topoisomerase 2-alpha OS=Mus musculus OX=10090 GN=Top2a PE=1 SV=2	173 kDa	9
DNA topoisomerase 2-beta OS=Mus musculus OX=10090 GN=Top2b PE=1 SV=2	182 kDa	3
DNA-(apurinic or apyrimidinic site) lyase OS=Mus musculus OX=10090 GN=Apex1 PE=1 SV=2	35 kDa	9
DNA-directed RNA polymerase I subunit RPA1 OS=Mus musculus OX=10090 GN=Polr1a PE=1 SV=2	194 kDa	2
DNA-directed RNA polymerase I subunit RPA12 OS=Mus musculus OX=10090 GN=Znrd1 PE=1 SV=1	14 kDa	1
DNA-directed RNA polymerase I subunit RPA34 OS=Mus musculus OX=10090 GN=Cd3eap PE=1 SV=2	43 kDa	1
DNA-directed RNA polymerase II subunit RPB2 OS=Mus musculus OX=10090 GN=Polr2b PE=1 SV=2	134 kDa	2
DNA-directed RNA polymerase II subunit RPB7 OS=Mus musculus OX=10090 GN=Polr2g PE=1 SV=1	19 kDa	4
DNA-directed RNA polymerase III subunit RPC3 OS=Mus musculus OX=10090 GN=Polr3c PE=1 SV=1	61 kDa	1
DNA-directed RNA polymerase subunit OS=Mus musculus OX=10090 GN=Polr2i PE=1 SV=1	12 kDa	2
DNA-directed RNA polymerases I and III subunit RPAC1 (Fragment) OS=Mus musculus OX=10090 GN=Polr1c PE=1 SV=2	29 kDa	1
DNA-directed RNA polymerases I, II, and III subunit RPABC1 OS=Mus musculus OX=10090 GN=Polr2e PE=1 SV=1	25 kDa	1
DNA-directed RNA polymerases I, II, and III subunit RPABC3 OS=Mus musculus OX=10090 GN=Polr2h PE=1 SV=3	17 kDa	4
DnaJ homolog subfamily A member 1 OS=Mus musculus OX=10090 GN=Dnaja1 PE=1 SV=1	45 kDa	14
DnaJ homolog subfamily A member 2 OS=Mus musculus OX=10090 GN=Dnaja2 PE=1 SV=1	46 kDa	8
DnaJ homolog subfamily B member 11 OS=Mus musculus OX=10090 GN=Dnajb11 PE=1 SV=1	41 kDa	2

DnaJ homolog subfamily C member 10 OS=Mus musculus OX=10090 GN=Dnajc10 PE=1 SV=2	91 kDa	3
DnaJ homolog subfamily C member 2 OS=Mus musculus OX=10090 GN=Dnajc2 PE=1 SV=2	72 kDa	2
DnaJ homolog subfamily C member 8 OS=Mus musculus OX=10090 GN=Dnajc8 PE=1 SV=2	30 kDa	13
DnaJ homolog subfamily C member 9 OS=Mus musculus OX=10090 GN=Dnajc9 PE=1 SV=2	30 kDa	2
Docking protein 1 OS=Mus musculus OX=10090 GN=Dok1 PE=1 SV=2	52 kDa	1
Dolichol-phosphate mannosyltransferase subunit 3 OS=Mus musculus OX=10090 GN=Dpm3 PE=1 SV=1	10 kDa	1
Dolichyl-diphosphooligosaccharide--protein glycosyltransferase 48 kDa subunit OS=Mus musculus OX=10090 GN=Ddost PE=1 SV=2	49 kDa	4
Dolichyl-diphosphooligosaccharide--protein glycosyltransferase subunit 1 OS=Mus musculus OX=10090 GN=Rpn1 PE=1 SV=1	69 kDa	12
Dolichyl-diphosphooligosaccharide--protein glycosyltransferase subunit 2 OS=Mus musculus OX=10090 GN=Rpn2 PE=1 SV=1	68 kDa	7
Dolichyl-diphosphooligosaccharide--protein glycosyltransferase subunit DAD1 OS=Mus musculus OX=10090 GN=Dad1 PE=1 SV=3	12 kDa	2
Double-strand break repair protein MRE11 OS=Mus musculus OX=10090 GN=Mre11 PE=1 SV=1	80 kDa	1
Double-strand-break repair protein rad21 homolog OS=Mus musculus OX=10090 GN=Rad21 PE=1 SV=3	72 kDa	1
Dr1-associated corepressor OS=Mus musculus OX=10090 GN=Drap1 PE=1 SV=1	23 kDa	1
Drebrin-like protein OS=Mus musculus OX=10090 GN=Dbnl PE=1 SV=2	49 kDa	12
Dual specificity mitogen-activated protein kinase kinase 1 OS=Mus musculus OX=10090 GN=Map2k1 PE=1 SV=2	43 kDa	1
Dual specificity protein phosphatase OS=Mus musculus OX=10090 GN=Dusp9 PE=1 SV=1	49 kDa	7
Dynactin 6, isoform CRA_b OS=Mus musculus OX=10090 GN=Dctn6 PE=1 SV=1	20 kDa	2
Dynactin subunit 1 OS=Mus musculus OX=10090 GN=Dctn1 PE=1 SV=1	127 kDa	9
Dynactin subunit 2 OS=Mus musculus OX=10090 GN=Dctn2 PE=1 SV=3	44 kDa	25
Dynactin subunit 4 OS=Mus musculus OX=10090 GN=Dctn4 PE=1 SV=1	53 kDa	4
Dynamin-1 OS=Mus musculus OX=10090 GN=Dnm1 PE=1 SV=1	94 kDa	1
Dynamin-2 OS=Mus musculus OX=10090 GN=Dnm2 PE=1 SV=2	98 kDa	1
Dynein light chain 1, cytoplasmic OS=Mus musculus OX=10090 GN=Dynll1 PE=1 SV=1	10 kDa	9

Dynein light chain roadblock-type 1 OS=Mus musculus OX=10090 GN=Dynlrb1 PE=1 SV=3	11 kDa	4
Dynein light chain Tctex-type 1 OS=Mus musculus OX=10090 GN=Dynlt1 PE=1 SV=1	12 kDa	1
Dystroglycan OS=Mus musculus OX=10090 GN=Dag1 PE=1 SV=4	97 kDa	2
E3 SUMO-protein ligase RanBP2 OS=Mus musculus OX=10090 GN=Ranbp2 PE=1 SV=2	341 kDa	3
E3 ubiquitin/ISG15 ligase TRIM25 OS=Mus musculus OX=10090 GN=Trim25 PE=1 SV=1	71 kDa	1
E3 ubiquitin-protein ligase ARIH1 OS=Mus musculus OX=10090 GN=Arih1 PE=1 SV=3	64 kDa	7
E3 ubiquitin-protein ligase BRE1A (Fragment) OS=Mus musculus OX=10090 GN=Rnf20 PE=1 SV=1	20 kDa	1
E3 ubiquitin-protein ligase CBL OS=Mus musculus OX=10090 GN=Cbl PE=1 SV=1	98 kDa	3
E3 ubiquitin-protein ligase HECTD1 OS=Mus musculus OX=10090 GN=Hectd1 PE=1 SV=1	289 kDa	1
E3 ubiquitin-protein ligase HUWE1 OS=Mus musculus OX=10090 GN=Huwe1 PE=1 SV=1	483 kDa	4
E3 ubiquitin-protein ligase KCMF1 OS=Mus musculus OX=10090 GN=Kcmf1 PE=1 SV=1	42 kDa	1
E3 ubiquitin-protein ligase NEDD4 OS=Mus musculus OX=10090 GN=Nedd4 PE=1 SV=3	103 kDa	17
E3 ubiquitin-protein ligase NEDD4-like OS=Mus musculus OX=10090 GN=Nedd4l PE=1 SV=2	115 kDa	2
E3 ubiquitin-protein ligase PPP1R11 OS=Mus musculus OX=10090 GN=Ppp1r11 PE=1 SV=1	15 kDa	6
E3 ubiquitin-protein ligase Praja-2 OS=Mus musculus OX=10090 GN=Pja2 PE=1 SV=2	78 kDa	1
E3 ubiquitin-protein ligase RBX1 OS=Mus musculus OX=10090 GN=Rbx1 PE=1 SV=1	12 kDa	10
E3 ubiquitin-protein ligase RING1 OS=Mus musculus OX=10090 GN=Ring1 PE=1 SV=2	43 kDa	1
E3 ubiquitin-protein ligase RING2 OS=Mus musculus OX=10090 GN=Rnf2 PE=1 SV=1	38 kDa	1
E3 ubiquitin-protein ligase RNF114 OS=Mus musculus OX=10090 GN=Rnf114 PE=1 SV=2	26 kDa	4
E3 ubiquitin-protein ligase RNF144B OS=Mus musculus OX=10090 GN=Rnf144b PE=2 SV=2	33 kDa	1
E3 ubiquitin-protein ligase TRIM56 OS=Mus musculus OX=10090 GN=Trim56 PE=1 SV=1	80 kDa	1
E3 ubiquitin-protein ligase UBR5 OS=Mus musculus OX=10090 GN=Ubr5 PE=4 SV=1	309 kDa	3
E3 ubiquitin-protein ligase UHRF1 OS=Mus musculus OX=10090 GN=Uhrf1 PE=1 SV=2	88 kDa	3
E3 ubiquitin-protein ligase ZNF598 OS=Mus musculus OX=10090 GN=Znf598 PE=1 SV=1	99 kDa	1
Early endosome antigen 1 OS=Mus musculus OX=10090 GN=Eea1 PE=1 SV=2	161 kDa	27

Echinoderm microtubule-associated protein-like 1 OS=Mus musculus OX=10090 GN=Eml1 PE=1 SV=1	88 kDa	1
Echinoderm microtubule-associated protein-like 2 OS=Mus musculus OX=10090 GN=Eml2 PE=1 SV=1	91 kDa	5
Echinoderm microtubule-associated protein-like 4 OS=Mus musculus OX=10090 GN=Eml4 PE=1 SV=1	110 kDa	4
Eef1d protein OS=Mus musculus OX=10090 GN=Eef1d PE=1 SV=1	31 kDa	32
EF-hand domain-containing protein D1 OS=Mus musculus OX=10090 GN=Efhd1 PE=1 SV=1	27 kDa	2
EF-hand domain-containing protein D2 OS=Mus musculus OX=10090 GN=Efhd2 PE=1 SV=1	27 kDa	18
EH domain-binding protein 1-like protein 1 OS=Mus musculus OX=10090 GN=Ehbp1l1 PE=1 SV=1	185 kDa	1
EKC/KEOPS complex subunit Lage3 OS=Mus musculus OX=10090 GN=Lage3 PE=1 SV=1	12 kDa	2
ELAV-like protein 1 OS=Mus musculus OX=10090 GN=Elavl1 PE=1 SV=2	36 kDa	7
Electron transfer flavoprotein subunit alpha, mitochondrial OS=Mus musculus OX=10090 GN=Etfa PE=1 SV=2	35 kDa	19
Electron transfer flavoprotein subunit beta OS=Mus musculus OX=10090 GN=Etfb PE=1 SV=3	28 kDa	13
ELKS/Rab6-interacting/CAST family member 1 OS=Mus musculus OX=10090 GN=Erc1 PE=1 SV=1	128 kDa	1
Elongation factor 1-alpha 1 OS=Mus musculus OX=10090 GN=Eef1a1 PE=1 SV=3	50 kDa	225
Elongation factor 1-beta OS=Mus musculus OX=10090 GN=Eef1b PE=1 SV=5	25 kDa	23
Elongation factor 1-delta (Fragment) OS=Mus musculus OX=10090 GN=Eef1d PE=1 SV=1	27 kDa	39
Elongation factor 1-delta OS=Mus musculus OX=10090 GN=Eef1d PE=1 SV=1	29 kDa	34
Elongation factor 1-gamma OS=Mus musculus OX=10090 GN=Eef1g PE=1 SV=3	50 kDa	89
Elongation factor 2 OS=Mus musculus OX=10090 GN=Eef2 PE=1 SV=2	95 kDa	308
Elongation factor G, mitochondrial OS=Mus musculus OX=10090 GN=Gfm1 PE=1 SV=1	84 kDa	1
Elongation factor Tu, mitochondrial OS=Mus musculus OX=10090 GN=Tufm PE=1 SV=1	50 kDa	7
Elongation factor-like GTPase 1 OS=Mus musculus OX=10090 GN=Efl1 PE=1 SV=1	126 kDa	1
Elongator complex protein 1 OS=Mus musculus OX=10090 GN=Elp1 PE=1 SV=2	150 kDa	3
Elongator complex protein 2 OS=Mus musculus OX=10090 GN=Elp2 PE=1 SV=1	93 kDa	1
Elongator complex protein 4 OS=Mus musculus OX=10090 GN=Elp4 PE=1 SV=2	46 kDa	2
Elongin-B OS=Mus musculus OX=10090 GN=Elob PE=1 SV=1	13 kDa	11

Elongin-C OS=Mus musculus OX=10090 GN=Eloc PE=1 SV=1	15 kDa	9
Embigin OS=Mus musculus OX=10090 GN=Emb PE=1 SV=2	37 kDa	7
Emerin OS=Mus musculus OX=10090 GN=Emd PE=1 SV=1	19 kDa	2
Ena/VASP-like protein OS=Mus musculus OX=10090 GN=Evl PE=1 SV=2	44 kDa	1
Endophilin-B1 OS=Mus musculus OX=10090 GN=Sh3glb1 PE=1 SV=1	44 kDa	1
Endophilin-B2 OS=Mus musculus OX=10090 GN=Sh3glb2 PE=1 SV=1	45 kDa	4
Endoplasmic reticulum aminopeptidase 1 OS=Mus musculus OX=10090 GN=Erap1 PE=1 SV=2	107 kDa	1
Endoplasmic reticulum chaperone BiP OS=Mus musculus OX=10090 GN=Hspa5 PE=1 SV=3	72 kDa	224
Endoplasmic reticulum junction formation protein lunapark OS=Mus musculus OX=10090 GN=Lnpk PE=1 SV=1	48 kDa	8
Endoplasmic reticulum metallopeptidase 1 OS=Mus musculus OX=10090 GN=Ermp1 PE=1 SV=2	100 kDa	12
Endoplasmic reticulum resident protein 29 OS=Mus musculus OX=10090 GN=Erp29 PE=1 SV=2	29 kDa	10
Endoplasmic reticulum resident protein 44 OS=Mus musculus OX=10090 GN=Erp44 PE=1 SV=1	47 kDa	7
Endoplasmic reticulum-Golgi intermediate compartment protein 1 OS=Mus musculus OX=10090 GN=Ergic1 PE=1 SV=1	33 kDa	1
Endoplasmin (Fragment) OS=Mus musculus OX=10090 GN=Hsp90b1 PE=1 SV=1	43 kDa	83
Endoplasmin OS=Mus musculus OX=10090 GN=Hsp90b1 PE=1 SV=2	92 kDa	146
Endoribonuclease LACTB2 OS=Mus musculus OX=10090 GN=Lactb2 PE=1 SV=1	33 kDa	10
Endothelial differentiation-related factor 1 OS=Mus musculus OX=10090 GN=Edf1 PE=1 SV=1	16 kDa	4
Enhancer of mRNA-decapping protein 4 OS=Mus musculus OX=10090 GN=Edc4 PE=1 SV=1	151 kDa	2
Enhancer of rudimentary homolog OS=Mus musculus OX=10090 GN=Erh PE=1 SV=1	12 kDa	13
Enoyl-[acyl-carrier-protein] reductase, mitochondrial OS=Mus musculus OX=10090 GN=Mecr PE=1 SV=2	40 kDa	2
Enoyl-CoA delta isomerase 1, mitochondrial OS=Mus musculus OX=10090 GN=Eci1 PE=1 SV=2	32 kDa	12
Enoyl-CoA hydratase domain-containing protein 3, mitochondrial OS=Mus musculus OX=10090 GN=Echdc3 PE=1 SV=1	32 kDa	1
Enoyl-CoA hydratase, mitochondrial OS=Mus musculus OX=10090 GN=Echs1 PE=1 SV=1	31 kDa	9
Epidermal growth factor receptor kinase substrate 8 OS=Mus musculus OX=10090 GN=Eps8 PE=1 SV=2	92 kDa	1

Epidermal growth factor receptor substrate 15-like 1 OS=Mus musculus OX=10090 GN=Eps15l1 PE=1 SV=3	99 kDa	6
Epididymis-specific alpha-mannosidase OS=Mus musculus OX=10090 GN=Man2b2 PE=1 SV=2	116 kDa	2
Epithelial membrane protein 2 OS=Mus musculus OX=10090 GN=Emp2 PE=1 SV=1	20 kDa	1
Epsin-2 OS=Mus musculus OX=10090 GN=Epn2 PE=1 SV=1	63 kDa	6
Epsin-3 OS=Mus musculus OX=10090 GN=Epn3 PE=1 SV=1	68 kDa	1
ER membrane protein complex subunit 2 OS=Mus musculus OX=10090 GN=Emc2 PE=1 SV=1	35 kDa	1
ER membrane protein complex subunit 6 OS=Mus musculus OX=10090 GN=Emc6 PE=1 SV=1	12 kDa	1
Erbb2ip protein OS=Mus musculus OX=10090 GN=Erbin PE=1 SV=1	145 kDa	1
ERC protein 2 OS=Mus musculus OX=10090 GN=Erc2 PE=1 SV=2	111 kDa	1
ERO1-like protein alpha OS=Mus musculus OX=10090 GN=Ero1a PE=1 SV=2	54 kDa	13
ES1 protein homolog, mitochondrial OS=Mus musculus OX=10090 GN=D10Jhu81e PE=1 SV=1	28 kDa	5
Ethanolamine-phosphate cytidylyltransferase OS=Mus musculus OX=10090 GN=Pcyt2 PE=1 SV=1	43 kDa	1
Ethylmalonyl-CoA decarboxylase (Fragment) OS=Mus musculus OX=10090 GN=Echdc1 PE=1 SV=1	18 kDa	1
Eukaryotic initiation factor 4A-I OS=Mus musculus OX=10090 GN=Eif4a1 PE=1 SV=1	46 kDa	56
Eukaryotic initiation factor 4A-III OS=Mus musculus OX=10090 GN=Eif4a3 PE=1 SV=3	47 kDa	19
Eukaryotic peptide chain release factor GTP-binding subunit ERF3A OS=Mus musculus OX=10090 GN=Gspt1 PE=1 SV=2	69 kDa	24
Eukaryotic peptide chain release factor subunit 1 OS=Mus musculus OX=10090 GN=Etf1 PE=1 SV=4	49 kDa	6
Eukaryotic translation elongation factor 1 epsilon-1 OS=Mus musculus OX=10090 GN=Eef1e1 PE=1 SV=1	20 kDa	2
Eukaryotic translation initiation factor 1 OS=Mus musculus OX=10090 GN=Eif1 PE=1 SV=2	13 kDa	9
Eukaryotic translation initiation factor 1A, X-chromosomal OS=Mus musculus OX=10090 GN=Eif1ax PE=2 SV=3	16 kDa	3
Eukaryotic translation initiation factor 2 subunit 1 OS=Mus musculus OX=10090 GN=Eif2s1 PE=1 SV=3	36 kDa	25
Eukaryotic translation initiation factor 2 subunit 2 (Fragment) OS=Mus musculus OX=10090 GN=Eif2s2 PE=1 SV=1	14 kDa	5
Eukaryotic translation initiation factor 2 subunit 2 OS=Mus musculus OX=10090 GN=Eif2s2 PE=1 SV=1	38 kDa	20
Eukaryotic translation initiation factor 2 subunit 3, X-linked OS=Mus musculus OX=10090 GN=Eif2s3x PE=1 SV=2	51 kDa	37

Eukaryotic translation initiation factor 2A OS=Mus musculus OX=10090 GN=Eif2a PE=1 SV=2	64 kDa	16
Eukaryotic translation initiation factor 2B, subunit 3 OS=Mus musculus OX=10090 GN=Eif2b3 PE=1 SV=1	50 kDa	2
Eukaryotic translation initiation factor 3 subunit A OS=Mus musculus OX=10090 GN=Eif3a PE=1 SV=5	162 kDa	20
Eukaryotic translation initiation factor 3 subunit B OS=Mus musculus OX=10090 GN=Eif3b PE=1 SV=1	91 kDa	35
Eukaryotic translation initiation factor 3 subunit C OS=Mus musculus OX=10090 GN=Eif3c PE=1 SV=1	106 kDa	16
Eukaryotic translation initiation factor 3 subunit D OS=Mus musculus OX=10090 GN=Eif3d PE=1 SV=2	64 kDa	5
Eukaryotic translation initiation factor 3 subunit E OS=Mus musculus OX=10090 GN=Eif3e PE=1 SV=1	52 kDa	4
Eukaryotic translation initiation factor 3 subunit F OS=Mus musculus OX=10090 GN=Eif3f PE=1 SV=2	38 kDa	5
Eukaryotic translation initiation factor 3 subunit G OS=Mus musculus OX=10090 GN=Eif3g PE=1 SV=2	36 kDa	9
Eukaryotic translation initiation factor 3 subunit H OS=Mus musculus OX=10090 GN=Eif3h PE=1 SV=1	40 kDa	21
Eukaryotic translation initiation factor 3 subunit I OS=Mus musculus OX=10090 GN=Eif3i PE=1 SV=1	36 kDa	7
Eukaryotic translation initiation factor 3 subunit J-A OS=Mus musculus OX=10090 GN=Eif3j1 PE=2 SV=1	29 kDa	14
Eukaryotic translation initiation factor 3 subunit J-B OS=Mus musculus OX=10090 GN=Eif3j2 PE=1 SV=1	29 kDa	12
Eukaryotic translation initiation factor 3 subunit K OS=Mus musculus OX=10090 GN=Eif3k PE=1 SV=1	25 kDa	5
Eukaryotic translation initiation factor 4 gamma 1 OS=Mus musculus OX=10090 GN=Eif4g1 PE=1 SV=1	175 kDa	33
Eukaryotic translation initiation factor 4 gamma 2 OS=Mus musculus OX=10090 GN=Eif4g2 PE=1 SV=1	98 kDa	1
Eukaryotic translation initiation factor 4 gamma 3 OS=Mus musculus OX=10090 GN=Eif4g3 PE=1 SV=1	193 kDa	3
Eukaryotic translation initiation factor 4B OS=Mus musculus OX=10090 GN=Eif4b PE=1 SV=1	69 kDa	52
Eukaryotic translation initiation factor 4E OS=Mus musculus OX=10090 GN=Eif4e PE=1 SV=1	25 kDa	7
Eukaryotic translation initiation factor 4E-binding protein 1 OS=Mus musculus OX=10090 GN=Eif4ebp1 PE=1 SV=3	12 kDa	3
Eukaryotic translation initiation factor 4E-binding protein 2 OS=Mus musculus OX=10090 GN=Eif4ebp2 PE=1 SV=1	13 kDa	2
Eukaryotic translation initiation factor 4H OS=Mus musculus OX=10090 GN=Eif4h PE=1 SV=3	27 kDa	20
Eukaryotic translation initiation factor 5 OS=Mus musculus OX=10090 GN=Eif5 PE=1 SV=1	49 kDa	15
Eukaryotic translation initiation factor 5A-1 OS=Mus musculus OX=10090 GN=Eif5a PE=1 SV=2	17 kDa	61
Eukaryotic translation initiation factor 5B OS=Mus musculus OX=10090 GN=Eif5b PE=1 SV=2	138 kDa	11

Eukaryotic translation initiation factor 6 OS=Mus musculus OX=10090 GN=Eif6 PE=1 SV=2	27 kDa	19
Exosome complex component MTR3 OS=Mus musculus OX=10090 GN=Exosc6 PE=1 SV=1	28 kDa	2
Exosome complex component RRP4 OS=Mus musculus OX=10090 GN=Exosc2 PE=1 SV=1	33 kDa	3
Exosome complex component RRP40 OS=Mus musculus OX=10090 GN=Exosc3 PE=1 SV=3	30 kDa	1
Exosome complex component RRP41 OS=Mus musculus OX=10090 GN=Exosc4 PE=1 SV=3	26 kDa	1
Exosome complex component RRP43 (Fragment) OS=Mus musculus OX=10090 GN=Exosc8 PE=1 SV=1	14 kDa	4
Exosome complex component RRP45 OS=Mus musculus OX=10090 GN=Exosc9 PE=1 SV=1	49 kDa	5
Exosome complex component RRP46 OS=Mus musculus OX=10090 GN=Exosc5 PE=1 SV=1	25 kDa	2
Exosome complex exonuclease RRP42 OS=Mus musculus OX=10090 GN=Exosc7 PE=1 SV=2	32 kDa	3
Exosome RNA helicase MTR4 OS=Mus musculus OX=10090 GN=Mtrex PE=1 SV=1	118 kDa	8
Exportin-1 OS=Mus musculus OX=10090 GN=Xpo1 PE=1 SV=1	123 kDa	5
Exportin-2 OS=Mus musculus OX=10090 GN=Cse1l PE=1 SV=1	104 kDa	9
Ezrin OS=Mus musculus OX=10090 GN=Ezr PE=1 SV=3	69 kDa	43
FACT complex subunit SSRP1 (Fragment) OS=Mus musculus OX=10090 GN=Ssrp1 PE=1 SV=1	72 kDa	8
F-actin-capping protein subunit alpha-2 OS=Mus musculus OX=10090 GN=Capza2 PE=1 SV=3	33 kDa	22
F-actin-capping protein subunit beta OS=Mus musculus OX=10090 GN=Capzb PE=1 SV=3	31 kDa	56
FAD-linked sulfhydryl oxidase ALR OS=Mus musculus OX=10090 GN=Gfer PE=1 SV=2	23 kDa	3
Family with sequence similarity 104, member A OS=Mus musculus OX=10090 GN=Fam104a PE=1 SV=1	20 kDa	1
Far upstream element (FUSE)-binding protein 3 OS=Mus musculus OX=10090 GN=Fubp3 PE=1 SV=1	61 kDa	8
Far upstream element-binding protein 1 OS=Mus musculus OX=10090 GN=Fubp1 PE=1 SV=1	67 kDa	39
Far upstream element-binding protein 2 OS=Mus musculus OX=10090 GN=Khsrp PE=1 SV=2	77 kDa	92
Farnesyl pyrophosphate synthase OS=Mus musculus OX=10090 GN=Fdps PE=1 SV=1	41 kDa	3
FAS-associated factor 1 OS=Mus musculus OX=10090 GN=Faf1 PE=1 SV=2	74 kDa	3
FAS-associated factor 2 OS=Mus musculus OX=10090 GN=Faf2 PE=1 SV=2	52 kDa	2
Fascin OS=Mus musculus OX=10090 GN=Fscn1 PE=1 SV=4	55 kDa	27

Fatty acid synthase OS=Mus musculus OX=10090 GN=Fasn PE=1 SV=1	272 kDa	52
Fatty acid-binding protein, epidermal OS=Mus musculus OX=10090 GN=Fabp5 PE=1 SV=3	15 kDa	15
Fatty aldehyde dehydrogenase OS=Mus musculus OX=10090 GN=Aldh3a2 PE=1 SV=2	54 kDa	6
F-box only protein 6 (Fragment) OS=Mus musculus OX=10090 GN=Fbxo6 PE=1 SV=1	27 kDa	1
F-box-like/WD repeat-containing protein TBL1X OS=Mus musculus OX=10090 GN=Tbl1x PE=1 SV=2	57 kDa	4
F-box-like/WD repeat-containing protein TBL1XR1 OS=Mus musculus OX=10090 GN=Tbl1xr1 PE=1 SV=1	56 kDa	4
FERM, ARHGEF and pleckstrin domain-containing protein 1 OS=Mus musculus OX=10090 GN=Farp1 PE=1 SV=1	119 kDa	4
Fermitin family homolog 2 OS=Mus musculus OX=10090 GN=Fermt2 PE=1 SV=1	78 kDa	4
Ferredoxin-2, mitochondrial OS=Mus musculus OX=10090 GN=Fdx2 PE=1 SV=1	19 kDa	1
Ferritin heavy chain OS=Mus musculus OX=10090 GN=Fth1 PE=1 SV=2	21 kDa	8
Ferritin light chain 1 OS=Mus musculus OX=10090 GN=Ftl1 PE=1 SV=2	21 kDa	6
Ferrochelatase, mitochondrial OS=Mus musculus OX=10090 GN=Fech PE=1 SV=2	47 kDa	4
FH1/FH2 domain-containing protein 1 OS=Mus musculus OX=10090 GN=Fhod1 PE=1 SV=3	130 kDa	1
Fibronectin OS=Mus musculus OX=10090 GN=Fn1 PE=1 SV=1	263 kDa	9
Filamin, alpha OS=Mus musculus OX=10090 GN=Flna PE=1 SV=1	280 kDa	204
Filamin-B OS=Mus musculus OX=10090 GN=Flnb PE=1 SV=3	278 kDa	106
Filamin-binding LIM protein 1 OS=Mus musculus OX=10090 GN=Fblim1 PE=1 SV=2	41 kDa	1
Filamin-C OS=Mus musculus OX=10090 GN=Flnc PE=1 SV=3	291 kDa	166
Flap endonuclease 1 OS=Mus musculus OX=10090 GN=Fen1 PE=1 SV=1	43 kDa	7
Flotillin-2 OS=Mus musculus OX=10090 GN=Flot2 PE=1 SV=2	47 kDa	1
Formin-binding protein 1 OS=Mus musculus OX=10090 GN=Fnbp1 PE=1 SV=1	71 kDa	2
Formin-binding protein 4 OS=Mus musculus OX=10090 GN=Fnbp4 PE=1 SV=1	116 kDa	5
Four and a half LIM domains protein 2 OS=Mus musculus OX=10090 GN=Fhl2 PE=1 SV=1	32 kDa	4
Four and a half LIM domains protein 3 OS=Mus musculus OX=10090 GN=Fhl3 PE=1 SV=2	32 kDa	14
Fragile X mental retardation syndrome-related protein 1 OS=Mus musculus OX=10090 GN=Fxr1 PE=1 SV=1	64 kDa	3

Fructose-bisphosphate aldolase A OS=Mus musculus OX=10090 GN=Aldoa PE=1 SV=2	39 kDa	171
Fumarate hydratase, mitochondrial OS=Mus musculus OX=10090 GN=Fh PE=1 SV=3	54 kDa	10
Fumarylacetoacetase OS=Mus musculus OX=10090 GN=Fah PE=1 SV=2	46 kDa	11
FUN14 domain-containing protein 2 OS=Mus musculus OX=10090 GN=Fundc2 PE=1 SV=1	17 kDa	2
G patch domain-containing protein 11 OS=Mus musculus OX=10090 GN=Gpatch11 PE=1 SV=2	31 kDa	1
G patch domain-containing protein 8 OS=Mus musculus OX=10090 GN=Gpatch8 PE=1 SV=1	165 kDa	1
Galactokinase OS=Mus musculus OX=10090 GN=Galk1 PE=1 SV=2	42 kDa	16
Galectin OS=Mus musculus OX=10090 GN=Lgals9 PE=1 SV=1	40 kDa	1
Galectin-1 OS=Mus musculus OX=10090 GN=Lgals1 PE=1 SV=3	15 kDa	172
Galectin-3 OS=Mus musculus OX=10090 GN=Lgals3 PE=1 SV=3	28 kDa	43
Gamma-glutamylcyclotransferase OS=Mus musculus OX=10090 GN=Ggct PE=1 SV=1	21 kDa	1
Gap junction alpha-1 protein OS=Mus musculus OX=10090 GN=Gja1 PE=1 SV=2	43 kDa	1
Gatad2b protein OS=Mus musculus OX=10090 GN=Gatad2b PE=1 SV=1	64 kDa	4
GDP-fucose protein O-fucosyltransferase 1 OS=Mus musculus OX=10090 GN=Pofut1 PE=1 SV=1	40 kDa	2
GDP-L-fucose synthase OS=Mus musculus OX=10090 GN=Tsta3 PE=1 SV=3	36 kDa	2
GDP-mannose 4,6 dehydratase OS=Mus musculus OX=10090 GN=Gmds PE=1 SV=1	42 kDa	8
Gelsolin (Fragment) OS=Mus musculus OX=10090 GN=Gsn PE=1 SV=1	28 kDa	6
Gelsolin OS=Mus musculus OX=10090 GN=Gsn PE=1 SV=3	86 kDa	26
General transcription factor IIE subunit 1 OS=Mus musculus OX=10090 GN=Gtf2e1 PE=1 SV=1	50 kDa	1
General transcription factor IIE subunit 2 (Fragment) OS=Mus musculus OX=10090 GN=Gtf2e2 PE=1 SV=1	14 kDa	1
General transcription factor IIF subunit 1 OS=Mus musculus OX=10090 GN=Gtf2f1 PE=1 SV=2	57 kDa	5
General transcription factor II-I (Fragment) OS=Mus musculus OX=10090 GN=Gtf2i PE=1 SV=2	50 kDa	1
General transcription factor II-I OS=Mus musculus OX=10090 GN=Gtf2i PE=1 SV=3	112 kDa	3
General transcription factor II-I repeat domain-containing protein 2 OS=Mus musculus OX=10090 GN=Gtf2ird2 PE=2 SV=1	105 kDa	1

General vesicular transport factor p115 OS=Mus musculus OX=10090 GN=Uso1 PE=1 SV=2	107 kDa	1
Gephyrin OS=Mus musculus OX=10090 GN=Gphn PE=1 SV=2	83 kDa	4
Geranylgeranyl pyrophosphate synthase OS=Mus musculus OX=10090 GN=Ggps1 PE=1 SV=1	35 kDa	2
Glia maturation factor beta (Fragment) OS=Mus musculus OX=10090 GN=Gmfb PE=4 SV=1	18 kDa	4
Glia maturation factor beta OS=Mus musculus OX=10090 GN=Gmfb PE=1 SV=3	17 kDa	5
Glucocorticoid modulatory element-binding protein 1 OS=Mus musculus OX=10090 GN=Gmeb1 PE=1 SV=2	61 kDa	1
Glucocorticoid receptor OS=Mus musculus OX=10090 GN=Nr3c1 PE=1 SV=1	87 kDa	1
Glucosamine 6-phosphate N-acetyltransferase (Fragment) OS=Mus musculus OX=10090 GN=Gnpnat1 PE=4 SV=1	15 kDa	1
Glucosamine-6-phosphate isomerase 1 OS=Mus musculus OX=10090 GN=Gnpda1 PE=1 SV=3	33 kDa	4
Glucosamine-6-phosphate isomerase OS=Mus musculus OX=10090 GN=Gnpda2 PE=1 SV=1	31 kDa	1
Glucose-6-phosphate 1-dehydrogenase OS=Mus musculus OX=10090 GN=G6pd2 PE=1 SV=1	59 kDa	1
Glucose-6-phosphate 1-dehydrogenase X OS=Mus musculus OX=10090 GN=G6pdx PE=1 SV=3	59 kDa	7
Glucose-6-phosphate isomerase OS=Mus musculus OX=10090 GN=Gpi PE=1 SV=4	63 kDa	80
Glucosidase 2 subunit beta OS=Mus musculus OX=10090 GN=Prkcsh PE=1 SV=1	59 kDa	28
Glucosylceramidase OS=Mus musculus OX=10090 GN=Gba PE=1 SV=1	58 kDa	4
Glutamate dehydrogenase 1, mitochondrial OS=Mus musculus OX=10090 GN=Glud1 PE=1 SV=1	61 kDa	70
Glutamine amidotransferase-like class 1 domain-containing protein 1 OS=Mus musculus OX=10090 GN=Gatd1 PE=1 SV=1	23 kDa	1
Glutamine synthetase OS=Mus musculus OX=10090 GN=Glul PE=1 SV=6	42 kDa	6
Glutamine--fructose-6-phosphate aminotransferase [isomerizing] 1 OS=Mus musculus OX=10090 GN=Gfpt1 PE=1 SV=3	79 kDa	1
Glutamine--tRNA ligase OS=Mus musculus OX=10090 GN=Qars PE=1 SV=1	88 kDa	5
Glutaredoxin-1 OS=Mus musculus OX=10090 GN=Glrx PE=1 SV=1	9 kDa	5
Glutaredoxin-3 OS=Mus musculus OX=10090 GN=Glrx3 PE=1 SV=1	38 kDa	4
Glutaredoxin-related protein 5, mitochondrial OS=Mus musculus OX=10090 GN=Glrx5 PE=1 SV=2	16 kDa	3
Glutathione reductase, mitochondrial OS=Mus musculus OX=10090 GN=Gsr PE=1 SV=3	54 kDa	19

Glutathione S-transferase (Fragment) OS=Mus musculus OX=10090 GN=Gstm6 PE=1 SV=1	18 kDa	2
Glutathione S-transferase Mu 1 OS=Mus musculus OX=10090 GN=Gstm1 PE=1 SV=2	29 kDa	17
Glutathione S-transferase Mu 2 OS=Mus musculus OX=10090 GN=Gstm2 PE=1 SV=2	26 kDa	17
Glutathione S-transferase omega-1 OS=Mus musculus OX=10090 GN=Gsto1 PE=1 SV=2	27 kDa	34
Glutathione S-transferase OS=Mus musculus OX=10090 GN=Gstm2 PE=1 SV=1	22 kDa	14
Glutathione S-transferase P 1 OS=Mus musculus OX=10090 GN=Gstp1 PE=1 SV=2	24 kDa	12
Glutathione synthetase OS=Mus musculus OX=10090 GN=Gss PE=1 SV=1	52 kDa	1
Glyceraldehyde-3-phosphate dehydrogenase (Fragment) OS=Mus musculus OX=10090 GN=Gapdh PE=1 SV=1	6 kDa	29
Glyceraldehyde-3-phosphate dehydrogenase OS=Mus musculus OX=10090 GN=Gapdh PE=1 SV=2	36 kDa	218
Glyceraldehyde-3-phosphate dehydrogenase OS=Mus musculus OX=10090 GN=Gapdhs PE=1 SV=1	47 kDa	19
Glyceraldehyde-3-phosphate dehydrogenase OS=Mus musculus OX=10090 GN=Gm3839 PE=1 SV=1	36 kDa	189
Glycerol-3-phosphate dehydrogenase, mitochondrial OS=Mus musculus OX=10090 GN=Gpd2 PE=1 SV=2	81 kDa	37
Glycerol-3-phosphate phosphatase OS=Mus musculus OX=10090 GN=Pgp PE=1 SV=1	35 kDa	13
Glycine amidinotransferase, mitochondrial OS=Mus musculus OX=10090 GN=Gatm PE=1 SV=1	48 kDa	13
Glycine cleavage system H protein, mitochondrial OS=Mus musculus OX=10090 GN=Gcsh PE=1 SV=2	19 kDa	1
Glycine--tRNA ligase OS=Mus musculus OX=10090 GN=Gars PE=1 SV=1	82 kDa	45
Glycogen phosphorylase, brain form OS=Mus musculus OX=10090 GN=Pygb PE=1 SV=3	97 kDa	18
Glycogen phosphorylase, liver form OS=Mus musculus OX=10090 GN=Pygl PE=1 SV=4	97 kDa	2
Glycogenin-1 OS=Mus musculus OX=10090 GN=Gyg1 PE=1 SV=3	37 kDa	2
Glycolipid transfer protein OS=Mus musculus OX=10090 GN=Gltp PE=1 SV=1	20 kDa	1
Glycosylated lysosomal membrane protein OS=Mus musculus OX=10090 GN=Glmp PE=1 SV=1	44 kDa	1
Glycylpeptide N-tetradecanoyltransferase 1 OS=Mus musculus OX=10090 GN=Nmt1 PE=1 SV=1	57 kDa	18
Glycylpeptide N-tetradecanoyltransferase 2 OS=Mus musculus OX=10090 GN=Nmt2 PE=1 SV=1	60 kDa	10
Glyoxalase domain-containing protein 4 OS=Mus musculus OX=10090 GN=Glod4 PE=1 SV=1	33 kDa	12
Glyoxalase domain-containing protein 4 OS=Mus musculus OX=10090 GN=Glod4 PE=1 SV=1	8 kDa	3

Glyoxylate reductase/hydroxypyruvate reductase OS=Mus musculus OX=10090 GN=Grhpr PE=1 SV=1	35 kDa	7
GMP reductase 1 OS=Mus musculus OX=10090 GN=Gmpr PE=1 SV=1	37 kDa	2
GMP reductase 2 OS=Mus musculus OX=10090 GN=Gmpr2 PE=1 SV=2	38 kDa	6
GMP synthase [glutamine-hydrolyzing] OS=Mus musculus OX=10090 GN=Gmps PE=1 SV=2	77 kDa	7
Golgi apparatus protein 1 (Fragment) OS=Mus musculus OX=10090 GN=Glg1 PE=1 SV=1	132 kDa	5
Golgi autoantigen, golgin subfamily b, macrogolgin 1 OS=Mus musculus OX=10090 GN=Golgb1 PE=1 SV=1	370 kDa	7
Golgin subfamily A member 1 OS=Mus musculus OX=10090 GN=Golga1 PE=1 SV=2	87 kDa	1
Golgin subfamily A member 2 OS=Mus musculus OX=10090 GN=Golga2 PE=1 SV=1	116 kDa	2
Golgin subfamily A member 3 OS=Mus musculus OX=10090 GN=Golga3 PE=1 SV=1	163 kDa	2
Golgin subfamily A member 5 OS=Mus musculus OX=10090 GN=Golga5 PE=1 SV=2	82 kDa	1
Granulins (Fragment) OS=Mus musculus OX=10090 GN=Grn PE=1 SV=1	36 kDa	5
Granulins OS=Mus musculus OX=10090 GN=Grn PE=1 SV=2	63 kDa	11
GRB10-interacting GYF protein 2 OS=Mus musculus OX=10090 GN=Gigyf2 PE=1 SV=2	149 kDa	3
Gremlin-1 OS=Mus musculus OX=10090 GN=Grem1 PE=2 SV=1	21 kDa	1
GRIP and coiled-coil domain containing 2 OS=Mus musculus OX=10090 GN=Gcc2 PE=1 SV=1	195 kDa	2
Group XIIA secretory phospholipase A2 (Fragment) OS=Mus musculus OX=10090 GN=Pla2g12a PE=1 SV=1	14 kDa	1
Group XV phospholipase A2 OS=Mus musculus OX=10090 GN=Pla2g15 PE=1 SV=1	47 kDa	1
Growth arrest and DNA damage-inducible proteins-interacting protein 1 OS=Mus musculus OX=10090 GN=Gadd45gip1 PE=1 SV=1	26 kDa	1
Growth factor receptor-bound protein 2 OS=Mus musculus OX=10090 GN=Grb2 PE=1 SV=1	24 kDa	5
Growth hormone-inducible transmembrane protein OS=Mus musculus OX=10090 GN=Ghitm PE=1 SV=1	37 kDa	3
GrpE protein homolog 1, mitochondrial OS=Mus musculus OX=10090 GN=Grpel1 PE=1 SV=1	24 kDa	10
GTPase KRas OS=Mus musculus OX=10090 GN=Kras PE=1 SV=1	22 kDa	5
GTPase NRas (Fragment) OS=Mus musculus OX=10090 GN=Nras PE=1 SV=1	21 kDa	6
GTPase-activating protein and VPS9 domain-containing protein 1 OS=Mus musculus OX=10090 GN=Gapvd1 PE=1 SV=2	162 kDa	2

GTP-binding nuclear protein Ran OS=Mus musculus OX=10090 GN=Ran PE=1 SV=3	24 kDa	41
GTP-binding nuclear protein Ran, testis-specific isoform OS=Mus musculus OX=10090 GN=Rasl2-9 PE=2 SV=1	24 kDa	17
GTP-binding protein 1 OS=Mus musculus OX=10090 GN=Gtpbp1 PE=1 SV=2	72 kDa	2
GTP-binding protein Rheb OS=Mus musculus OX=10090 GN=Rheb PE=1 SV=1	20 kDa	2
GTP-binding protein SAR1a OS=Mus musculus OX=10090 GN=Sar1a PE=1 SV=1	22 kDa	3
Guanine nucleotide exchange factor MSS4 OS=Mus musculus OX=10090 GN=Rabif PE=1 SV=1	14 kDa	1
Guanine nucleotide-binding protein G(i) subunit alpha-2 (Fragment) OS=Mus musculus OX=10090 GN=Gnai2 PE=1 SV=1	18 kDa	2
Guanine nucleotide-binding protein G(i) subunit alpha-2 OS=Mus musculus OX=10090 GN=Gnai2 PE=1 SV=5	40 kDa	4
Guanine nucleotide-binding protein G(I)/G(S)/G(T) subunit beta-1 OS=Mus musculus OX=10090 GN=Gnb1 PE=1 SV=3	37 kDa	16
Guanine nucleotide-binding protein G(I)/G(S)/G(T) subunit beta-2 OS=Mus musculus OX=10090 GN=Gnb2 PE=1 SV=3	37 kDa	12
Guanine nucleotide-binding protein G(s) subunit alpha isoforms XLas OS=Mus musculus OX=10090 GN=Gnas PE=1 SV=1	122 kDa	5
Guanine nucleotide-binding protein G(t) subunit alpha-1 OS=Mus musculus OX=10090 GN=Gnat1 PE=1 SV=3	40 kDa	2
Guanine nucleotide-binding protein-like 3 OS=Mus musculus OX=10090 GN=Gnl3 PE=1 SV=2	61 kDa	4
H/ACA ribonucleoprotein complex subunit (Fragment) OS=Mus musculus OX=10090 GN=Gar1 PE=1 SV=1	20 kDa	2
H/ACA ribonucleoprotein complex subunit 2 OS=Mus musculus OX=10090 GN=Nhp2 PE=1 SV=1	17 kDa	1
H/ACA ribonucleoprotein complex subunit 4 OS=Mus musculus OX=10090 GN=Dkc1 PE=1 SV=4	57 kDa	8
H-2 class I histocompatibility antigen, D-B alpha chain OS=Mus musculus OX=10090 GN=H2-D1 PE=1 SV=2	41 kDa	1
H-2 class I histocompatibility antigen, Q7 alpha chain OS=Mus musculus OX=10090 GN=H2-Q7 PE=3 SV=2	35 kDa	1
Haloacid dehalogenase-like hydrolase domain-containing protein 2 OS=Mus musculus OX=10090 GN=Hdhd2 PE=1 SV=2	29 kDa	3
HAUS augmin-like complex subunit 7 OS=Mus musculus OX=10090 GN=Haus7 PE=1 SV=2	41 kDa	1
HCLS1-binding protein 3 OS=Mus musculus OX=10090 GN=Hs1bp3 PE=1 SV=2	44 kDa	3
HD domain-containing protein 2 OS=Mus musculus OX=10090 GN=Hddc2 PE=1 SV=1	23 kDa	1
Heat shock 70 kDa protein 14 OS=Mus musculus OX=10090 GN=Hspa14 PE=1 SV=2	55 kDa	3

Heat shock 70 kDa protein 1-like OS=Mus musculus OX=10090 GN=Hspa1l PE=1 SV=4	71 kDa	21
Heat shock 70 kDa protein 4 OS=Mus musculus OX=10090 GN=Hspa4 PE=1 SV=1	94 kDa	40
Heat shock 70 kDa protein 4L OS=Mus musculus OX=10090 GN=Hspa4l PE=1 SV=2	94 kDa	4
Heat shock cognate 71 kDa protein OS=Mus musculus OX=10090 GN=Hspa8 PE=1 SV=1	71 kDa	258
Heat shock protein 105 kDa OS=Mus musculus OX=10090 GN=Hsph1 PE=1 SV=1	92 kDa	16
Heat shock protein 105 kDa OS=Mus musculus OX=10090 GN=Hsph1 PE=1 SV=2	96 kDa	17
Heat shock protein 75 kDa, mitochondrial OS=Mus musculus OX=10090 GN=Trap1 PE=1 SV=1	80 kDa	10
Heat shock protein beta-8 OS=Mus musculus OX=10090 GN=Hspb8 PE=1 SV=1	22 kDa	4
Heat shock protein HSP 90-alpha OS=Mus musculus OX=10090 GN=Hsp90aa1 PE=1 SV=4	85 kDa	216
Heat shock protein HSP 90-beta OS=Mus musculus OX=10090 GN=Hsp90ab1 PE=1 SV=3	83 kDa	290
Heme oxygenase 2 OS=Mus musculus OX=10090 GN=Hmox2 PE=1 SV=1	36 kDa	6
HemK methyltransferase family member 2, isoform CRA_c OS=Mus musculus OX=10090 GN=N6amt1 PE=1 SV=1	23 kDa	1
Heparan sulfate 2-O-sulfotransferase 1 OS=Mus musculus OX=10090 GN=Hs2st1 PE=1 SV=2	42 kDa	1
Hepatocyte growth factor-regulated tyrosine kinase substrate (Fragment) OS=Mus musculus OX=10090 GN=Hgs PE=1 SV=1	28 kDa	4
Hepatocyte growth factor-regulated tyrosine kinase substrate OS=Mus musculus OX=10090 GN=Hgs PE=1 SV=1	86 kDa	4
Hepatoma-derived growth factor OS=Mus musculus OX=10090 GN=Hdgf PE=1 SV=2	26 kDa	35
Hepatoma-derived growth factor-related protein 2 OS=Mus musculus OX=10090 GN=Hdgfl2 PE=1 SV=1	74 kDa	15
Heterochromatin protein 1-binding protein 3 OS=Mus musculus OX=10090 GN=Hp1bp3 PE=1 SV=1	61 kDa	5
Heterogeneous nuclear ribonucleoprotein A/B OS=Mus musculus OX=10090 GN=Hnrnpab PE=1 SV=1	36 kDa	28
Heterogeneous nuclear ribonucleoprotein A0 OS=Mus musculus OX=10090 GN=Hnrnpa0 PE=1 SV=1	31 kDa	6
Heterogeneous nuclear ribonucleoprotein A1 OS=Mus musculus OX=10090 GN=Hnrnpa1 PE=1 SV=1	39 kDa	29
Heterogeneous nuclear ribonucleoprotein A3 OS=Mus musculus OX=10090 GN=Hnrnpa3 PE=1 SV=1	34 kDa	38
Heterogeneous nuclear ribonucleoprotein D, isoform CRA_a OS=Mus musculus OX=10090 GN=Hnrnpd PE=1 SV=1	36 kDa	25
Heterogeneous nuclear ribonucleoprotein D0 OS=Mus musculus OX=10090 GN=Hnrnpd PE=1 SV=2	38 kDa	26

Heterogeneous nuclear ribonucleoprotein D-like OS=Mus musculus OX=10090 GN=Hnrnpdl PE=1 SV=1	46 kDa	13
Heterogeneous nuclear ribonucleoprotein F OS=Mus musculus OX=10090 GN=Hnrnpf PE=1 SV=3	46 kDa	15
Heterogeneous nuclear ribonucleoprotein H OS=Mus musculus OX=10090 GN=Hnrnph1 PE=1 SV=3	49 kDa	18
Heterogeneous nuclear ribonucleoprotein H2 OS=Mus musculus OX=10090 GN=Hnrnph2 PE=1 SV=1	49 kDa	11
Heterogeneous nuclear ribonucleoprotein K OS=Mus musculus OX=10090 GN=Hnrnpk PE=1 SV=1	51 kDa	44
Heterogeneous nuclear ribonucleoprotein L (Fragment) OS=Mus musculus OX=10090 GN=Hnrnpl PE=1 SV=1	67 kDa	22
Heterogeneous nuclear ribonucleoprotein L-like OS=Mus musculus OX=10090 GN=Hnrnpll PE=1 SV=3	64 kDa	6
Heterogeneous nuclear ribonucleoprotein M OS=Mus musculus OX=10090 GN=Hnrnpm PE=1 SV=3	78 kDa	36
Heterogeneous nuclear ribonucleoprotein Q OS=Mus musculus OX=10090 GN=Syncrip PE=1 SV=1	63 kDa	23
Heterogeneous nuclear ribonucleoprotein R OS=Mus musculus OX=10090 GN=Hnrnpr PE=1 SV=1	60 kDa	13
Heterogeneous nuclear ribonucleoprotein U OS=Mus musculus OX=10090 GN=Hnrnpu PE=1 SV=1	88 kDa	40
Heterogeneous nuclear ribonucleoprotein U-like protein 1 OS=Mus musculus OX=10090 GN=Hnrnpul1 PE=1 SV=1	96 kDa	3
Heterogeneous nuclear ribonucleoprotein U-like protein 2 OS=Mus musculus OX=10090 GN=Hnrnpul2 PE=1 SV=2	85 kDa	4
Heterogeneous nuclear ribonucleoproteins A2/B1 OS=Mus musculus OX=10090 GN=Hnrnpa2b1 PE=1 SV=2	37 kDa	45
Heterogeneous nuclear ribonucleoproteins C1/C2 OS=Mus musculus OX=10090 GN=Hnrnpc PE=1 SV=1	34 kDa	19
Hexokinase 1, isoform CRA_f OS=Mus musculus OX=10090 GN=Hk1 PE=1 SV=1	102 kDa	6
High affinity cationic amino acid transporter 1 OS=Mus musculus OX=10090 GN=Slc7a1 PE=1 SV=1	67 kDa	1
High mobility group nucleosome-binding domain-containing protein 5 OS=Mus musculus OX=10090 GN=Hmgn5 PE=1 SV=2	45 kDa	6
High mobility group protein B1 OS=Mus musculus OX=10090 GN=Hmgb1 PE=1 SV=2	25 kDa	113
High mobility group protein B2 OS=Mus musculus OX=10090 GN=Hmgb2 PE=1 SV=3	24 kDa	67
High mobility group protein B3 OS=Mus musculus OX=10090 GN=Hmgb3 PE=1 SV=3	23 kDa	7
High mobility group protein HMG-I/HMG-Y OS=Mus musculus OX=10090 GN=Hmga1 PE=1 SV=4	12 kDa	42
High mobility group protein HMGI-C OS=Mus musculus OX=10090 GN=Hmga2 PE=1 SV=1	12 kDa	29
HIRA-interacting protein 3 OS=Mus musculus OX=10090 GN=Hirip3 PE=1 SV=1	65 kDa	5

Histidine triad nucleotide-binding protein 1 OS=Mus musculus OX=10090 GN=Hint1 PE=1 SV=3	14 kDa	11
Histidine--tRNA ligase, cytoplasmic OS=Mus musculus OX=10090 GN=Hars PE=1 SV=2	57 kDa	20
Histidyl-tRNA synthetase-like, isoform CRA_a OS=Mus musculus OX=10090 GN=Hars2 PE=1 SV=1	48 kDa	5
Histocompatibility 13 (Fragment) OS=Mus musculus OX=10090 GN=H13 PE=1 SV=1	38 kDa	3
Histone acetyltransferase KAT7 OS=Mus musculus OX=10090 GN=Kat7 PE=1 SV=1	71 kDa	1
Histone acetyltransferase type B catalytic subunit OS=Mus musculus OX=10090 GN=Hat1 PE=1 SV=1	50 kDa	5
Histone deacetylase 4 OS=Mus musculus OX=10090 GN=Hdac4 PE=1 SV=1	119 kDa	2
Histone deacetylase 6 (Fragment) OS=Mus musculus OX=10090 GN=Hdac6 PE=1 SV=1	110 kDa	1
Histone deacetylase complex subunit SAP18 OS=Mus musculus OX=10090 GN=Sap18 PE=1 SV=1	20 kDa	2
Histone deacetylase OS=Mus musculus OX=10090 GN=Gm10093 PE=3 SV=1	55 kDa	8
Histone deacetylase OS=Mus musculus OX=10090 GN=Hdac2 PE=1 SV=1	55 kDa	7
Histone deacetylase OS=Mus musculus OX=10090 GN=Hdac7 PE=1 SV=1	92 kDa	1
Histone H1.0 OS=Mus musculus OX=10090 GN=H1f0 PE=2 SV=4	21 kDa	8
Histone H1.1 OS=Mus musculus OX=10090 GN=Hist1h1a PE=1 SV=2	22 kDa	10
Histone H1.2 OS=Mus musculus OX=10090 GN=Hist1h1c PE=1 SV=2	21 kDa	32
Histone H1.3 OS=Mus musculus OX=10090 GN=Hist1h1d PE=1 SV=2	22 kDa	33
Histone H1.4 OS=Mus musculus OX=10090 GN=Hist1h1e PE=1 SV=2	22 kDa	38
Histone H1.5 OS=Mus musculus OX=10090 GN=Hist1h1b PE=1 SV=2	23 kDa	20
Histone H2A OS=Mus musculus OX=10090 GN=H2afj PE=1 SV=1	14 kDa	32
Histone H2A type 2-A OS=Mus musculus OX=10090 GN=Hist2h2aa1 PE=1 SV=3	14 kDa	31
Histone H2A type 2-B OS=Mus musculus OX=10090 GN=Hist2h2ab PE=1 SV=3	14 kDa	11
Histone H2A.V OS=Mus musculus OX=10090 GN=H2afv PE=1 SV=3	14 kDa	13
Histone H2B type 1-B OS=Mus musculus OX=10090 GN=Hist1h2bb PE=1 SV=3	14 kDa	89
Histone H3.2 OS=Mus musculus OX=10090 GN=Hist2h3c1 PE=1 SV=1	20 kDa	9
Histone H4 OS=Mus musculus OX=10090 GN=Hist1h4a PE=1 SV=2	11 kDa	100

Histone-arginine methyltransferase CARM1 OS=Mus musculus OX=10090 GN=Carm1 PE=1 SV=2	66 kDa	2
Histone-binding protein RBBP4 OS=Mus musculus OX=10090 GN=Rbbp4 PE=1 SV=5	48 kDa	25
Histone-binding protein RBBP7 OS=Mus musculus OX=10090 GN=Rbbp7 PE=1 SV=1	48 kDa	35
Histone-lysine N-methyltransferase setd3 OS=Mus musculus OX=10090 GN=Setd3 PE=1 SV=1	67 kDa	2
HN1-like protein OS=Mus musculus OX=10090 GN=AY358078 PE=2 SV=1	53 kDa	6
Homeobox protein cut-like 1 OS=Mus musculus OX=10090 GN=Cux1 PE=1 SV=3	166 kDa	3
Homeobox protein Rhox5 OS=Mus musculus OX=10090 GN=Rhox5 PE=1 SV=1	23 kDa	4
Host cell factor 1 OS=Mus musculus OX=10090 GN=Hcfc1 PE=1 SV=1	215 kDa	32
Hsc70-interacting protein OS=Mus musculus OX=10090 GN=St13 PE=1 SV=1	42 kDa	27
Hsp70-binding protein 1 OS=Mus musculus OX=10090 GN=Hspbp1 PE=1 SV=1	39 kDa	3
Hsp90 co-chaperone Cdc37 OS=Mus musculus OX=10090 GN=Cdc37 PE=1 SV=1	45 kDa	34
Huntingtin-interacting protein 1 OS=Mus musculus OX=10090 GN=Hip1 PE=1 SV=2	115 kDa	2
Hyaluronan mediated motility receptor OS=Mus musculus OX=10090 GN=Hmmr PE=1 SV=4	92 kDa	1
Hydroxyacyl-coenzyme A dehydrogenase, mitochondrial OS=Mus musculus OX=10090 GN=Hadh PE=1 SV=2	34 kDa	5
Hydroxyacylglutathione hydrolase, mitochondrial (Fragment) OS=Mus musculus OX=10090 GN=Hagh PE=1 SV=1	26 kDa	4
Hydroxymethylglutaryl-CoA lyase, mitochondrial OS=Mus musculus OX=10090 GN=Hmgcl PE=1 SV=2	34 kDa	6
Hypoxanthine-guanine phosphoribosyltransferase OS=Mus musculus OX=10090 GN=Hprt1 PE=1 SV=3	25 kDa	14
Hypoxia up-regulated protein 1 OS=Mus musculus OX=10090 GN=Hyou1 PE=1 SV=1	111 kDa	21
IgG receptor FcRn large subunit p51 OS=Mus musculus OX=10090 GN=Fcgrt PE=1 SV=1	40 kDa	1
Immunoglobulin-binding protein 1 OS=Mus musculus OX=10090 GN=Igbp1 PE=1 SV=1	39 kDa	3
Immunoglobulin-binding protein 1b OS=Mus musculus OX=10090 GN=Igbp1b PE=1 SV=1	39 kDa	1
Importin subunit alpha-1 OS=Mus musculus OX=10090 GN=Kpna2 PE=1 SV=2	58 kDa	18
Importin subunit alpha-3 OS=Mus musculus OX=10090 GN=Kpna4 PE=1 SV=1	68 kDa	1
Importin subunit alpha-3 OS=Mus musculus OX=10090 GN=Kpna4 PE=1 SV=1	58 kDa	2
Importin subunit alpha-4 OS=Mus musculus OX=10090 GN=Kpna3 PE=1 SV=1	58 kDa	1

Importin subunit beta-1 OS=Mus musculus OX=10090 GN=Kpnb1 PE=1 SV=2	97 kDa	32
Importin-4 OS=Mus musculus OX=10090 GN=Ipo4 PE=1 SV=1	119 kDa	1
Importin-5 OS=Mus musculus OX=10090 GN=Ipo5 PE=1 SV=3	124 kDa	24
Importin-7 OS=Mus musculus OX=10090 GN=Ipo7 PE=1 SV=2	119 kDa	2
Inactive tyrosine-protein kinase 7 OS=Mus musculus OX=10090 GN=Ptk7 PE=1 SV=1	118 kDa	6
Inhibitor of nuclear factor kappa-B kinase-interacting protein OS=Mus musculus OX=10090 GN=Ikbip PE=1 SV=1	42 kDa	5
Inorganic pyrophosphatase 2, mitochondrial OS=Mus musculus OX=10090 GN=Ppa2 PE=1 SV=1	38 kDa	6
Inorganic pyrophosphatase OS=Mus musculus OX=10090 GN=Ppa1 PE=1 SV=1	33 kDa	19
Inosine triphosphate pyrophosphatase OS=Mus musculus OX=10090 GN=Itpa PE=1 SV=2	22 kDa	7
Inosine-5'-monophosphate dehydrogenase 1 (Fragment) OS=Mus musculus OX=10090 GN=Impdh1 PE=1 SV=1	25 kDa	2
Inosine-5'-monophosphate dehydrogenase 2 (Fragment) OS=Mus musculus OX=10090 GN=Impdh2 PE=1 SV=1	34 kDa	13
Inosine-5'-monophosphate dehydrogenase 2 OS=Mus musculus OX=10090 GN=Impdh2 PE=1 SV=2	56 kDa	23
Inositol monophosphatase 1 OS=Mus musculus OX=10090 GN=Impa1 PE=1 SV=1	30 kDa	18
Inositol monophosphatase 2 OS=Mus musculus OX=10090 GN=Impa2 PE=1 SV=1	32 kDa	6
Inositol polyphosphate 1-phosphatase OS=Mus musculus OX=10090 GN=Inpp1 PE=1 SV=2	43 kDa	1
Inositol-3-phosphate synthase 1 OS=Mus musculus OX=10090 GN=Isyna1 PE=1 SV=1	61 kDa	3
Insulin-degrading enzyme (Fragment) OS=Mus musculus OX=10090 GN=Ide PE=1 SV=1	114 kDa	16
Insulin-degrading enzyme OS=Mus musculus OX=10090 GN=Ide PE=1 SV=1	118 kDa	14
Insulin-like growth factor 2 mRNA-binding protein 2 OS=Mus musculus OX=10090 GN=Igf2bp2 PE=1 SV=1	66 kDa	10
Insulin-like growth factor 2 mRNA-binding protein 3 OS=Mus musculus OX=10090 GN=Igf2bp3 PE=1 SV=1	64 kDa	4
Insulin-like growth factor-binding protein 4 OS=Mus musculus OX=10090 GN=Igfbp4 PE=1 SV=1	18 kDa	2
Integrin alpha-1 OS=Mus musculus OX=10090 GN=Itga1 PE=1 SV=2	131 kDa	1
Integrin alpha-3 OS=Mus musculus OX=10090 GN=Itga3 PE=1 SV=1	117 kDa	1
Integrin alpha-5 OS=Mus musculus OX=10090 GN=Itga5 PE=1 SV=3	115 kDa	5
Integrin alpha-6 (Fragment) OS=Mus musculus OX=10090 GN=Itga6 PE=1 SV=1	75 kDa	15

Integrin alpha-6 OS=Mus musculus OX=10090 GN=Itga6 PE=1 SV=3	122 kDa	21
Integrin beta-1 OS=Mus musculus OX=10090 GN=Itgb1 PE=1 SV=1	88 kDa	55
Integrin beta-7 OS=Mus musculus OX=10090 GN=Itgb7 PE=1 SV=2	87 kDa	1
Integrin-linked protein kinase (Fragment) OS=Mus musculus OX=10090 GN=Ilk PE=1 SV=2	34 kDa	1
Interferon regulatory factor 2-binding protein 2 OS=Mus musculus OX=10090 GN=Irf2bp2 PE=1 SV=1	59 kDa	9
Interferon regulatory factor 2-binding protein-like OS=Mus musculus OX=10090 GN=Irf2bpl PE=1 SV=1	81 kDa	2
Interferon-induced transmembrane protein 3 OS=Mus musculus OX=10090 GN=Ifitm3 PE=1 SV=1	15 kDa	8
Interferon-inducible protein AIM2 OS=Mus musculus OX=10090 GN=Aim2 PE=1 SV=2	40 kDa	1
Interleukin enhancer-binding factor 2 OS=Mus musculus OX=10090 GN=Ilf2 PE=1 SV=1	43 kDa	3
Interleukin enhancer-binding factor 3 OS=Mus musculus OX=10090 GN=Ilf3 PE=1 SV=1	97 kDa	3
Inverted formin-2 OS=Mus musculus OX=10090 GN=Inf2 PE=1 SV=1	138 kDa	1
Isoamyl acetate-hydrolyzing esterase 1 homolog (Fragment) OS=Mus musculus OX=10090 GN=Iah1 PE=1 SV=1	18 kDa	2
Isocitrate dehydrogenase [NAD] subunit gamma 1, mitochondrial OS=Mus musculus OX=10090 GN=Idh3g PE=1 SV=1	43 kDa	1
Isocitrate dehydrogenase [NAD] subunit, mitochondrial OS=Mus musculus OX=10090 GN=Idh3a PE=1 SV=1	42 kDa	18
Isocitrate dehydrogenase [NAD] subunit, mitochondrial OS=Mus musculus OX=10090 GN=Idh3b PE=1 SV=1	42 kDa	2
Isocitrate dehydrogenase [NADP] cytoplasmic OS=Mus musculus OX=10090 GN=Idh1 PE=1 SV=2	47 kDa	9
Isocitrate dehydrogenase [NADP], mitochondrial OS=Mus musculus OX=10090 GN=Idh2 PE=1 SV=3	51 kDa	6
Isoleucine--tRNA ligase, cytoplasmic OS=Mus musculus OX=10090 GN=Iars PE=1 SV=2	144 kDa	9
Isoleucine--tRNA ligase, mitochondrial OS=Mus musculus OX=10090 GN=Iars2 PE=1 SV=1	113 kDa	13
Isopentenyl-diphosphate Delta-isomerase 1 OS=Mus musculus OX=10090 GN=Idi1 PE=1 SV=1	26 kDa	1
Isovaleryl-CoA dehydrogenase, mitochondrial OS=Mus musculus OX=10090 GN=Ivd PE=1 SV=1	46 kDa	3
IST1 homolog OS=Mus musculus OX=10090 GN=Ist1 PE=1 SV=1	39 kDa	1
Jumonji domain containing 1B OS=Mus musculus OX=10090 GN=Kdm3b PE=1 SV=1	191 kDa	1
Jupiter microtubule associated homolog 1 OS=Mus musculus OX=10090 GN=JPT1 PE=1 SV=3	16 kDa	7

	Jupiter microtubule associated homolog 2 OS=Mus musculus OX=10090 GN=Jpt2 PE=1 SV=1	20 kDa	22
	KDEL motif-containing protein 1 OS=Mus musculus OX=10090 GN=Kdelc1 PE=1 SV=1	15 kDa	1
	Kelch domain-containing protein 4 OS=Mus musculus OX=10090 GN=Klhdc4 PE=1 SV=1	61 kDa	8
*	Keratin 15, isoform CRA_a OS=Mus musculus OX=10090 GN=Krt15 PE=1 SV=1	49 kDa	9
*	Keratin 78 OS=Mus musculus OX=10090 GN=Krt78 PE=1 SV=1	112 kDa	6
*	Keratin, type I cytoskeletal 10 OS=Homo sapiens GN=KRT10 PE=1 SV=6	60 kDa	37
*	Keratin, type I cytoskeletal 16 OS=Mus musculus OX=10090 GN=Krt16 PE=1 SV=3	52 kDa	5
*	Keratin, type I cytoskeletal 17 OS=Mus musculus OX=10090 GN=Krt17 PE=1 SV=3	48 kDa	8
*	Keratin, type I cytoskeletal 18 OS=Mus musculus OX=10090 GN=Krt18 PE=1 SV=5	48 kDa	3
*	Keratin, type I cytoskeletal 42 OS=Mus musculus OX=10090 GN=Krt42 PE=1 SV=1	50 kDa	3
*	Keratin, type I cytoskeletal 9 OS=Homo sapiens GN=KRT9 PE=1 SV=3	62 kDa	38
*	Keratin, type II cytoskeletal 1 OS=Homo sapiens GN=KRT1 PE=1 SV=6	66 kDa	72
*	Keratin, type II cytoskeletal 1b OS=Mus musculus OX=10090 GN=Krt77 PE=1 SV=1	61 kDa	8
*	Keratin, type II cytoskeletal 2 epidermal OS=Homo sapiens GN=KRT2 PE=1 SV=2	66 kDa	27
*	Keratin, type II cytoskeletal 2 epidermal OS=Mus musculus OX=10090 GN=Krt2 PE=1 SV=1	71 kDa	11
*	Keratin, type II cytoskeletal 2 oral OS=Mus musculus OX=10090 GN=Krt76 PE=1 SV=1	63 kDa	7
*	Keratin, type II cytoskeletal 5 OS=Mus musculus OX=10090 GN=Krt5 PE=1 SV=1	62 kDa	13
*	Keratin, type II cytoskeletal 6A OS=Mus musculus OX=10090 GN=Krt6a PE=1 SV=3	59 kDa	10
*	Keratin, type II cytoskeletal 6B OS=Mus musculus OX=10090 GN=Krt6b PE=1 SV=3	60 kDa	9
*	Keratin, type II cytoskeletal 7 OS=Mus musculus OX=10090 GN=Krt7 PE=1 SV=1	51 kDa	6
*	Keratin, type II cytoskeletal 74 OS=Mus musculus OX=10090 GN=Krt74 PE=3 SV=1	55 kDa	6
*	Keratin, type II cytoskeletal 75 OS=Mus musculus OX=10090 GN=Krt75 PE=1 SV=1	60 kDa	8
*	Keratin, type II cytoskeletal 79 OS=Mus musculus OX=10090 GN=Krt79 PE=1 SV=2	58 kDa	8
*	Keratin, type II cytoskeletal 8 OS=Mus musculus OX=10090 GN=Krt8 PE=1 SV=4	55 kDa	13

KH domain-containing, RNA-binding, signal transduction-associated protein 1 OS=Mus musculus OX=10090 GN=Khdrbs1 PE=1 SV=2	48 kDa	5
Kinectin OS=Mus musculus OX=10090 GN=Ktn1 PE=1 SV=1	147 kDa	11
Kinesin heavy chain isoform 5A OS=Mus musculus OX=10090 GN=Kif5a PE=1 SV=3	117 kDa	2
Kinesin light chain 1 OS=Mus musculus OX=10090 GN=Klc1 PE=1 SV=1	62 kDa	6
Kinesin light chain 1 OS=Mus musculus OX=10090 GN=Klc1 PE=1 SV=3	61 kDa	3
Kinesin light chain 2 OS=Mus musculus OX=10090 GN=Klc2 PE=1 SV=1	68 kDa	4
Kinesin light chain 4 OS=Mus musculus OX=10090 GN=Klc4 PE=1 SV=1	69 kDa	2
Kinesin-1 heavy chain OS=Mus musculus OX=10090 GN=Kif5b PE=1 SV=3	110 kDa	9
Kinesin-like protein KIF2C OS=Mus musculus OX=10090 GN=Kif2c PE=1 SV=1	81 kDa	1
Kinesin-like protein OS=Mus musculus OX=10090 GN=Kif2a PE=1 SV=1	84 kDa	1
KxDL motif-containing protein 1 OS=Mus musculus OX=10090 GN=Kxd1 PE=1 SV=1	27 kDa	81
Kynurenine--oxoglutarate transaminase 3 (Fragment) OS=Mus musculus OX=10090 GN=Kyat3 PE=1 SV=1	22 kDa	1
Lactadherin OS=Mus musculus OX=10090 GN=Mfge8 PE=1 SV=3	51 kDa	15
Lactoylglutathione lyase OS=Mus musculus OX=10090 GN=Glo1 PE=1 SV=3	21 kDa	8
Lamina-associated polypeptide 2, isoforms alpha/zeta OS=Mus musculus OX=10090 GN=Tmpo PE=1 SV=4	75 kDa	20
Lamina-associated polypeptide 2, isoforms beta/delta/epsilon/gamma OS=Mus musculus OX=10090 GN=Tmpo PE=1 SV=4	50 kDa	26
Lamin-B receptor OS=Mus musculus OX=10090 GN=Lbr PE=1 SV=2	71 kDa	3
Lamin-B1 OS=Mus musculus OX=10090 GN=Lmnb1 PE=1 SV=3	67 kDa	27
Lamin-B2 OS=Mus musculus OX=10090 GN=Lmnb2 PE=1 SV=1	69 kDa	4
Laminin subunit alpha-5 OS=Mus musculus OX=10090 GN=Lama5 PE=1 SV=4	404 kDa	7
Laminin subunit beta-1 OS=Mus musculus OX=10090 GN=Lamb1 PE=1 SV=1	202 kDa	26
Laminin subunit gamma-1 OS=Mus musculus OX=10090 GN=Lamc1 PE=1 SV=1	177 kDa	12
Laminin subunit gamma-1 OS=Mus musculus OX=10090 GN=Lamc1 PE=1 SV=2	177 kDa	10
Lanosterol synthase OS=Mus musculus OX=10090 GN=Lss PE=1 SV=2	83 kDa	1

La-related protein 1 OS=Mus musculus OX=10090 GN=Larp1 PE=1 SV=3	121 kDa	4
La-related protein 4B (Fragment) OS=Mus musculus OX=10090 GN=Larp4b PE=4 SV=6	4 kDa	1
La-related protein 7 OS=Mus musculus OX=10090 GN=Larp7 PE=1 SV=2	65 kDa	5
Large neutral amino acids transporter small subunit 1 OS=Mus musculus OX=10090 GN=Slc7a5 PE=1 SV=2	56 kDa	9
Large proline-rich protein BAG6 OS=Mus musculus OX=10090 GN=Bag6 PE=1 SV=1	121 kDa	12
Lariat debranching enzyme OS=Mus musculus OX=10090 GN=Dbr1 PE=1 SV=2	62 kDa	1
Latent-transforming growth factor beta-binding protein 1 (Fragment) OS=Mus musculus OX=10090 GN=Ltbp1 PE=1 SV=8	107 kDa	1
Latent-transforming growth factor beta-binding protein 3 OS=Mus musculus OX=10090 GN=Ltbp3 PE=1 SV=1	134 kDa	3
Latent-transforming growth factor beta-binding protein 3 OS=Mus musculus OX=10090 GN=Ltbp3 PE=1 SV=3	136 kDa	3
Latexin OS=Mus musculus OX=10090 GN=Lxn PE=1 SV=2	25 kDa	2
Legumain OS=Mus musculus OX=10090 GN=Lgmn PE=1 SV=1	49 kDa	1
Leucine rich repeat (In FLII) interacting protein 1, isoform CRA_e OS=Mus musculus OX=10090 GN=Lrrfip1 PE=1 SV=1	49 kDa	5
Leucine rich repeat containing 40, isoform CRA_a OS=Mus musculus OX=10090 GN=Lrrc40 PE=1 SV=1	68 kDa	1
Leucine-rich repeat flightless-interacting protein 1 (Fragment) OS=Mus musculus OX=10090 GN=Lrrfip1 PE=1 SV=1	65 kDa	5
Leucine-rich repeat flightless-interacting protein 1 OS=Mus musculus OX=10090 GN=Lrrfip1 PE=1 SV=2	79 kDa	24
Leucine-rich repeat flightless-interacting protein 2 (Fragment) OS=Mus musculus OX=10090 GN=Lrrfip2 PE=1 SV=1	17 kDa	1
Leucine-rich repeat flightless-interacting protein 2 (Fragment) OS=Mus musculus OX=10090 GN=Lrrfip2 PE=1 SV=4	20 kDa	3
Leucine-rich repeat flightless-interacting protein 2 OS=Mus musculus OX=10090 GN=Lrrfip2 PE=1 SV=1	47 kDa	2
Leucine-rich repeat-containing protein 47 OS=Mus musculus OX=10090 GN=Lrrc47 PE=1 SV=1	64 kDa	15
Leucine-rich repeat-containing protein 59 OS=Mus musculus OX=10090 GN=Lrrc59 PE=1 SV=1	35 kDa	21
Leucine-rich repeats and immunoglobulin-like domains protein 1 OS=Mus musculus OX=10090 GN=Lrig1 PE=1 SV=2	119 kDa	1
Leucine--tRNA ligase, cytoplasmic OS=Mus musculus OX=10090 GN=Lars PE=1 SV=2	134 kDa	11

Leucyl-cystinyl aminopeptidase OS=Mus musculus OX=10090 GN=Lnpep PE=1 SV=1	117 kDa	9
Leukocyte surface antigen CD47 OS=Mus musculus OX=10090 GN=Cd47 PE=1 SV=2	33 kDa	3
Leukotriene A-4 hydrolase OS=Mus musculus OX=10090 GN=Lta4h PE=1 SV=4	69 kDa	28
LIM and senescent cell antigen-like-containing domain protein OS=Mus musculus OX=10090 GN=Lims1 PE=1 SV=1	38 kDa	6
LIM and SH3 domain protein 1 OS=Mus musculus OX=10090 GN=Lasp1 PE=1 SV=1	30 kDa	34
LIM domain and actin-binding protein 1 OS=Mus musculus OX=10090 GN=Lima1 PE=1 SV=3	84 kDa	11
LIM domain-containing protein 2 OS=Mus musculus OX=10090 GN=Limd2 PE=1 SV=1	14 kDa	1
Lipoamide acyltransferase component of branched-chain alpha-keto acid dehydrogenase complex, mitochondrial OS=Mus musculus OX=10090 GN=Dbt PE=1 SV=2	53 kDa	1
Lipoma-preferred partner homolog OS=Mus musculus OX=10090 GN=Lpp PE=1 SV=1	66 kDa	11
Liprin-beta-1 OS=Mus musculus OX=10090 GN=Ppfibp1 PE=1 SV=3	109 kDa	5
L-lactate dehydrogenase (Fragment) OS=Mus musculus OX=10090 GN=Ldhb PE=1 SV=1	21 kDa	12
L-lactate dehydrogenase A chain OS=Mus musculus OX=10090 GN=Ldha PE=1 SV=3	36 kDa	154
Lon protease homolog, mitochondrial OS=Mus musculus OX=10090 GN=Lonp1 PE=1 SV=2	106 kDa	13
Long-chain-fatty-acid--CoA ligase 5 OS=Mus musculus OX=10090 GN=Acsl5 PE=1 SV=1	76 kDa	1
Long-chain-specific acyl-CoA dehydrogenase, mitochondrial OS=Mus musculus OX=10090 GN=Acadl PE=1 SV=1	48 kDa	33
Low density lipoprotein receptor-related protein 1 OS=Mus musculus OX=10090 GN=Lrp1 PE=1 SV=1	505 kDa	34
Low-density lipoprotein receptor OS=Mus musculus OX=10090 GN=Ldlr PE=1 SV=2	95 kDa	5
LRP chaperone MESD OS=Mus musculus OX=10090 GN=Mesd PE=1 SV=1	25 kDa	13
Luc7-like protein 3 OS=Mus musculus OX=10090 GN=Luc7l3 PE=1 SV=1	51 kDa	7
Lupus La protein homolog OS=Mus musculus OX=10090 GN=Ssb PE=1 SV=1	48 kDa	29
LYR motif-containing protein 4 OS=Mus musculus OX=10090 GN=Lyrm4 PE=1 SV=1	11 kDa	1
Lys-63-specific deubiquitinase BRCC36 (Fragment) OS=Mus musculus OX=10090 GN=Brcc3 PE=1 SV=1	18 kDa	1
Lysine--tRNA ligase OS=Mus musculus OX=10090 GN=Kars PE=1 SV=1	68 kDa	48
Lysophosphatidic acid phosphatase type 6 OS=Mus musculus OX=10090 GN=Acp6 PE=1 SV=1	48 kDa	1

Lysosomal alpha-glucosidase OS=Mus musculus OX=10090 GN=Gaa PE=1 SV=2	106 kDa	9
Lysosomal alpha-mannosidase OS=Mus musculus OX=10090 GN=Man2b1 PE=1 SV=4	115 kDa	5
Lysosome membrane protein 2 OS=Mus musculus OX=10090 GN=Scarb2 PE=1 SV=3	54 kDa	3
Lysosome-associated membrane glycoprotein 1 OS=Mus musculus OX=10090 GN=Lamp1 PE=1 SV=2	44 kDa	14
Lysosome-associated membrane glycoprotein 2 OS=Mus musculus OX=10090 GN=Lamp2 PE=1 SV=2	46 kDa	10
m7GpppX diphosphatase OS=Mus musculus OX=10090 GN=Dcps PE=1 SV=1	39 kDa	17
Macrophage erythroblast attacher OS=Mus musculus OX=10090 GN=Maea PE=1 SV=1	45 kDa	1
Macrophage migration inhibitory factor OS=Mus musculus OX=10090 GN=Mif PE=1 SV=2	13 kDa	16
Mago nashi protein OS=Mus musculus OX=10090 GN=Magohb PE=1 SV=1	17 kDa	1
Major vault protein OS=Mus musculus OX=10090 GN=Mvp PE=1 SV=1	97 kDa	15
Malate dehydrogenase, cytoplasmic OS=Mus musculus OX=10090 GN=Mdh1 PE=1 SV=3	37 kDa	32
Malate dehydrogenase, mitochondrial OS=Mus musculus OX=10090 GN=Mdh2 PE=1 SV=3	36 kDa	77
Malectin OS=Mus musculus OX=10090 GN=Mlec PE=1 SV=2	32 kDa	1
Malignant T-cell-amplified sequence 1 OS=Mus musculus OX=10090 GN=Mcts1 PE=1 SV=1	21 kDa	8
Mammalian ependymin-related protein 1 OS=Mus musculus OX=10090 GN=Epdr1 PE=1 SV=1	25 kDa	4
Mannose-1-phosphate guanyltransferase alpha OS=Mus musculus OX=10090 GN=Gmppa PE=1 SV=1	46 kDa	1
Mannose-1-phosphate guanyltransferase beta OS=Mus musculus OX=10090 GN=Gmppb PE=1 SV=1	40 kDa	2
Mannose-6-phosphate isomerase OS=Mus musculus OX=10090 GN=Mpi PE=1 SV=1	47 kDa	5
Mannosyl-oligosaccharide glucosidase OS=Mus musculus OX=10090 GN=Mogs PE=1 SV=1	92 kDa	8
Mapk-regulated corepressor-interacting protein 1 OS=Mus musculus OX=10090 GN=Mcrip1 PE=1 SV=1	11 kDa	4
MARCKS-related protein OS=Mus musculus OX=10090 GN=Marcksl1 PE=1 SV=2	20 kDa	5
Matrin-3 OS=Mus musculus OX=10090 GN=Matr3 PE=1 SV=1	95 kDa	17
Matrix metalloproteinase OS=Mus musculus OX=10090 GN=Mmp14 PE=1 SV=1	64 kDa	1
Max protein OS=Mus musculus OX=10090 GN=Max PE=1 SV=1	17 kDa	2
MCG10134, isoform CRA_c OS=Mus musculus OX=10090 GN=Tpd52 PE=1 SV=1	18 kDa	7

MCG115964 OS=Mus musculus OX=10090 GN=Wdr70 PE=1 SV=1	73 kDa	4
MCG119397 OS=Mus musculus OX=10090 GN=Gm9774 PE=4 SV=1	42 kDa	5
MCG12127, isoform CRA_e OS=Mus musculus OX=10090 GN=Lsm7 PE=1 SV=1	9 kDa	2
MCG123888 OS=Mus musculus OX=10090 GN=Mkl2 PE=1 SV=1	119 kDa	1
MCG125586 OS=Mus musculus OX=10090 GN=Rhox2d PE=1 SV=1	21 kDa	2
MCG126099, isoform CRA_b OS=Mus musculus OX=10090 GN=Ppp1r2 PE=1 SV=1	22 kDa	4
MCG129810, isoform CRA_c OS=Mus musculus OX=10090 GN=Pdxdc1 PE=1 SV=1	87 kDa	1
MCG130490 OS=Mus musculus OX=10090 GN=Ppp1r12b PE=1 SV=1	111 kDa	1
MCG130675 OS=Mus musculus OX=10090 GN=Zbed5 PE=2 SV=1	84 kDa	3
MCG13402, isoform CRA_a OS=Mus musculus OX=10090 GN=Ptbp1 PE=1 SV=1	57 kDa	19
MCG140437, isoform CRA_d OS=Mus musculus OX=10090 GN=Myh2 PE=1 SV=1	223 kDa	3
MCG1407, isoform CRA_a OS=Mus musculus OX=10090 GN=Rpf2 PE=1 SV=1	31 kDa	1
MCG14602, isoform CRA_b (Fragment) OS=Mus musculus OX=10090 GN=Ddx27 PE=1 SV=1	34 kDa	1
MCG16685, isoform CRA_d OS=Mus musculus OX=10090 GN=Wtap PE=1 SV=1	44 kDa	2
MCG5603 OS=Mus musculus OX=10090 GN=Ndufa11 PE=1 SV=1	15 kDa	2
MCG6846, isoform CRA_c OS=Mus musculus OX=10090 GN=Prpsap1 PE=1 SV=1	42 kDa	4
MCG7614, isoform CRA_c OS=Mus musculus OX=10090 GN=Srsf5 PE=1 SV=1	31 kDa	3
Mediator of DNA damage checkpoint protein 1 OS=Mus musculus OX=10090 GN=Mdc1 PE=1 SV=1	185 kDa	1
Mediator of RNA polymerase II transcription subunit 8 OS=Mus musculus OX=10090 GN=Med8 PE=1 SV=1	14 kDa	2
Medium-chain specific acyl-CoA dehydrogenase, mitochondrial OS=Mus musculus OX=10090 GN=Acadm PE=1 SV=1	46 kDa	3
Melanoma inhibitory activity protein 2 OS=Mus musculus OX=10090 GN=Mia2 PE=1 SV=1	87 kDa	3
Melanoma inhibitory activity protein 2 OS=Mus musculus OX=10090 GN=Mia2 PE=1 SV=2	157 kDa	3
Membrane magnesium transporter 1 OS=Mus musculus OX=10090 GN=Mmgt1 PE=1 SV=1	15 kDa	1
Membrane-associated progesterone receptor component 1 OS=Mus musculus OX=10090 GN=Pgrmc1 PE=1 SV=4	22 kDa	4

Membrane-associated progesterone receptor component 2 OS=Mus musculus OX=10090 GN=Pgrmc2 PE=1 SV=2	23 kDa	5
Metalloreductase STEAP3 OS=Mus musculus OX=10090 GN=Steap3 PE=1 SV=1	55 kDa	6
Metallothionein-1 OS=Mus musculus OX=10090 GN=Mt1 PE=1 SV=1	6 kDa	18
Metallothionein-2 OS=Mus musculus OX=10090 GN=Mt2 PE=1 SV=2	6 kDa	23
Metastasis-associated protein MTA1 OS=Mus musculus OX=10090 GN=Mta1 PE=1 SV=1	79 kDa	4
Metastasis-associated protein MTA2 OS=Mus musculus OX=10090 GN=Mta2 PE=1 SV=1	75 kDa	9
Metaxin-1 OS=Mus musculus OX=10090 GN=Mtx1 PE=1 SV=2	48 kDa	1
Methionine adenosyltransferase 2 subunit beta OS=Mus musculus OX=10090 GN=Mat2b PE=1 SV=1	37 kDa	3
Methionine aminopeptidase 1 OS=Mus musculus OX=10090 GN=Metap1 PE=1 SV=1	43 kDa	6
Methionine aminopeptidase 2 OS=Mus musculus OX=10090 GN=Metap2 PE=1 SV=1	53 kDa	1
Methionine sulfoxide reductase B3 OS=Mus musculus OX=10090 GN=Msrb3 PE=1 SV=1	20 kDa	1
Methionine--tRNA ligase, cytoplasmic OS=Mus musculus OX=10090 GN=Mars PE=1 SV=1	102 kDa	8
Methyl-CpG-binding domain protein 3 OS=Mus musculus OX=10090 GN=Mbd3 PE=1 SV=1	31 kDa	1
Methylcrotonoyl-CoA carboxylase beta chain, mitochondrial OS=Mus musculus OX=10090 GN=Mccc2 PE=1 SV=1	61 kDa	1
Methylglutaconyl-CoA hydratase, mitochondrial OS=Mus musculus OX=10090 GN=Auh PE=1 SV=1	33 kDa	1
Methylosome protein 50 OS=Mus musculus OX=10090 GN=Wdr77 PE=1 SV=1	37 kDa	9
Methylthioribose-1-phosphate isomerase OS=Mus musculus OX=10090 GN=Mri1 PE=1 SV=1	39 kDa	6
Methylthioribulose-1-phosphate dehydratase OS=Mus musculus OX=10090 GN=Apip PE=1 SV=1	27 kDa	1
MHC class II regulatory factor RFX1 OS=Mus musculus OX=10090 GN=Rfx1 PE=1 SV=1	98 kDa	1
MICAL-like protein 2 OS=Mus musculus OX=10090 GN=Micall2 PE=1 SV=1	99 kDa	3
MICOS complex subunit Mic19 OS=Mus musculus OX=10090 GN=Chchd3 PE=1 SV=1	26 kDa	11
MICOS complex subunit MIC60 OS=Mus musculus OX=10090 GN=Immt PE=1 SV=1	76 kDa	4
MICOS complex subunit MIC60 OS=Mus musculus OX=10090 GN=Immt PE=1 SV=1	54 kDa	2
MICOS complex subunit OS=Mus musculus OX=10090 GN=Chchd6 PE=1 SV=1	26 kDa	2
Microfibrillar-associated protein 1A OS=Mus musculus OX=10090 GN=Mfap1a PE=1 SV=1	52 kDa	4

Microtubule-associated protein (Fragment) OS=Mus musculus OX=10090 GN=Map4 PE=1 SV=1	153 kDa	30
Microtubule-associated protein 1B OS=Mus musculus OX=10090 GN=Map1b PE=1 SV=2	270 kDa	32
Microtubule-associated protein 1S OS=Mus musculus OX=10090 GN=Map1s PE=1 SV=2	103 kDa	14
Microtubule-associated protein 2 (Fragment) OS=Mus musculus OX=10090 GN=Map2 PE=1 SV=1	67 kDa	1
Microtubule-associated protein 4 OS=Mus musculus OX=10090 GN=Map4 PE=1 SV=3	117 kDa	67
Microtubule-associated protein OS=Mus musculus OX=10090 GN=Map4 PE=1 SV=3	98 kDa	33
Microtubule-associated protein RP/EB family member 1 OS=Mus musculus OX=10090 GN=Mapre1 PE=1 SV=3	30 kDa	20
MIP18 family protein FAM96A OS=Mus musculus OX=10090 GN=Fam96a PE=1 SV=1	18 kDa	1
Mitochondrial 10-formyltetrahydrofolate dehydrogenase OS=Mus musculus OX=10090 GN=Aldh1l2 PE=1 SV=2	102 kDa	3
Mitochondrial 2-oxoglutarate/malate carrier protein OS=Mus musculus OX=10090 GN=Slc25a11 PE=1 SV=3	34 kDa	1
Mitochondrial amidoxime reducing component 2 OS=Mus musculus OX=10090 GN=Marc2 PE=1 SV=1	38 kDa	3
Mitochondrial antiviral-signaling protein OS=Mus musculus OX=10090 GN=Mavs PE=1 SV=1	53 kDa	4
Mitochondrial carrier homolog 2 OS=Mus musculus OX=10090 GN=Mtch2 PE=1 SV=1	32 kDa	1
Mitochondrial fission 1 protein OS=Mus musculus OX=10090 GN=Fis1 PE=1 SV=1	17 kDa	12
Mitochondrial fission factor OS=Mus musculus OX=10090 GN=Mff PE=1 SV=1	36 kDa	2
Mitochondrial import inner membrane translocase subunit Tim13 OS=Mus musculus OX=10090 GN=Timm13 PE=1 SV=1	10 kDa	5
Mitochondrial import inner membrane translocase subunit Tim13 OS=Mus musculus OX=10090 GN=Timm13 PE=1 SV=1	9 kDa	2
Mitochondrial import receptor subunit TOM20 homolog OS=Mus musculus OX=10090 GN=Tomm20 PE=1 SV=1	11 kDa	2
Mitochondrial import receptor subunit TOM34 OS=Mus musculus OX=10090 GN=Tomm34 PE=1 SV=1	34 kDa	4
Mitochondrial import receptor subunit TOM40 homolog OS=Mus musculus OX=10090 GN=Tomm40 PE=1 SV=3	38 kDa	1
Mitochondrial import receptor subunit TOM70 OS=Mus musculus OX=10090 GN=Tomm70 PE=1 SV=2	68 kDa	4
Mitochondrial proton/calcium exchanger protein OS=Mus musculus OX=10090 GN=Letm1 PE=1 SV=1	83 kDa	3
Mitochondrial-processing peptidase subunit alpha OS=Mus musculus OX=10090 GN=Pmpca PE=1 SV=1	58 kDa	6
Mitochondrial-processing peptidase subunit beta OS=Mus musculus OX=10090 GN=Pmpcb PE=1 SV=1	55 kDa	6

Mitogen-activated protein kinase 1 OS=Mus musculus OX=10090 GN=Mapk1 PE=1 SV=3	41 kDa	17
Mitogen-activated protein kinase kinase kinase 20 OS=Mus musculus OX=10090 GN=Map3k20 PE=1 SV=1	92 kDa	1
Mitogen-activated protein kinase OS=Mus musculus OX=10090 GN=Mapk3 PE=1 SV=1	42 kDa	6
Mitotic checkpoint protein BUB3 OS=Mus musculus OX=10090 GN=Bub3 PE=1 SV=1	37 kDa	20
Mitotic spindle assembly checkpoint protein MAD2A OS=Mus musculus OX=10090 GN=Mad2l1 PE=1 SV=2	24 kDa	2
Mixed lineage kinase domain-like protein OS=Mus musculus OX=10090 GN=Mlkl PE=1 SV=1	54 kDa	3
MKI67 FHA domain-interacting nucleolar phosphoprotein OS=Mus musculus OX=10090 GN=Nifk PE=1 SV=1	36 kDa	3
MLV-related proviral Env polyprotein OS=Mus musculus OX=10090 PE=1 SV=3	70 kDa	5
MOB kinase activator 1A OS=Mus musculus OX=10090 GN=Mob1a PE=2 SV=3	25 kDa	3
Moesin OS=Mus musculus OX=10090 GN=Msn PE=1 SV=3	68 kDa	103
Monocarboxylate transporter 1 OS=Mus musculus OX=10090 GN=Slc16a1 PE=1 SV=1	53 kDa	2
Monofunctional C1-tetrahydrofolate synthase, mitochondrial OS=Mus musculus OX=10090 GN=Mthfd1l PE=1 SV=2	106 kDa	17
Mortality factor 4-like protein 2 (Fragment) OS=Mus musculus OX=10090 GN=Morf4l2 PE=1 SV=1	24 kDa	1
mRNA cap guanine-N7 methyltransferase OS=Mus musculus OX=10090 GN=Rnmt PE=1 SV=1	53 kDa	7
Multifunctional protein ADE2 OS=Mus musculus OX=10090 GN=Paics PE=1 SV=4	47 kDa	41
Multiple inositol polyphosphate phosphatase 1 OS=Mus musculus OX=10090 GN=Minpp1 PE=1 SV=3	55 kDa	2
Murinoglobulin-1 OS=Mus musculus OX=10090 GN=Mug1 PE=1 SV=3	165 kDa	1
Muscleblind-like protein 1 OS=Mus musculus OX=10090 GN=Mbnl1 PE=1 SV=1	42 kDa	6
Muscleblind-like protein 2 OS=Mus musculus OX=10090 GN=Mbnl2 PE=2 SV=2	40 kDa	5
Myb-binding protein 1A OS=Mus musculus OX=10090 GN=Mybbp1a PE=1 SV=2	152 kDa	37
Myc box-dependent-interacting protein 1 OS=Mus musculus OX=10090 GN=Bin1 PE=1 SV=1	64 kDa	16
Myelin expression factor 2 OS=Mus musculus OX=10090 GN=Myef2 PE=1 SV=1	60 kDa	2
Myeloid-associated differentiation marker OS=Mus musculus OX=10090 GN=Myadm PE=1 SV=2	35 kDa	3
Myeloid-derived growth factor OS=Mus musculus OX=10090 GN=Mydgf PE=1 SV=1	18 kDa	10
Myoferlin OS=Mus musculus OX=10090 GN=Myof PE=1 SV=2	233 kDa	20

Myosin light chain 1/3, skeletal muscle isoform OS=Mus musculus OX=10090 GN=Myl1 PE=1 SV=1	18 kDa	5
Myosin light polypeptide 6 OS=Mus musculus OX=10090 GN=Myl6 PE=1 SV=1	17 kDa	26
Myosin regulatory light chain 12B OS=Mus musculus OX=10090 GN=Myl12b PE=1 SV=2	20 kDa	17
Myosin, heavy chain 15 OS=Mus musculus OX=10090 GN=Myh15 PE=1 SV=1	222 kDa	3
Myosin, heavy polypeptide 13, skeletal muscle OS=Mus musculus OX=10090 GN=Myh13 PE=1 SV=1	224 kDa	3
Myosin-10 OS=Mus musculus OX=10090 GN=Myh10 PE=1 SV=2	229 kDa	39
Myosin-11 OS=Mus musculus OX=10090 GN=Myh11 PE=1 SV=1	223 kDa	24
Myosin-3 OS=Mus musculus OX=10090 GN=Myh3 PE=2 SV=2	224 kDa	3
Myosin-4 OS=Mus musculus OX=10090 GN=Myh4 PE=2 SV=1	223 kDa	3
Myosin-7 OS=Mus musculus OX=10090 GN=Myh7 PE=2 SV=1	223 kDa	3
Myosin-7B OS=Mus musculus OX=10090 GN=Myh7b PE=3 SV=1	222 kDa	3
Myosin-8 OS=Mus musculus OX=10090 GN=Myh8 PE=2 SV=2	223 kDa	3
Myosin-9 OS=Mus musculus OX=10090 GN=Myh9 PE=1 SV=4	226 kDa	271
Myotrophin OS=Mus musculus OX=10090 GN=Mtpn PE=1 SV=2	13 kDa	4
N(4)-(beta-N-acetylglucosaminyl)-L-asparaginase OS=Mus musculus OX=10090 GN=Aga PE=1 SV=1	37 kDa	9
N(G),N(G)-dimethylarginine dimethylaminohydrolase 1 OS=Mus musculus OX=10090 GN=Ddah1 PE=1 SV=3	31 kDa	4
Na(+)/H(+) exchange regulatory cofactor NHE-RF OS=Mus musculus OX=10090 GN=Slc9a3r2 PE=1 SV=1	37 kDa	1
Na(+)/H(+) exchange regulatory cofactor NHE-RF1 OS=Mus musculus OX=10090 GN=Slc9a3r1 PE=1 SV=3	39 kDa	8
N-acetyl-D-glucosamine kinase OS=Mus musculus OX=10090 GN=Nagk PE=1 SV=3	37 kDa	3
N-acetylglucosamine-6-phosphate deacetylase (Fragment) OS=Mus musculus OX=10090 GN=Amdhd2 PE=1 SV=8	22 kDa	1
N-acetylglucosamine-6-sulfatase OS=Mus musculus OX=10090 GN=Gns PE=1 SV=1	61 kDa	14
NAD kinase 2, mitochondrial OS=Mus musculus OX=10090 GN=Nadk2 PE=1 SV=2	51 kDa	1
NAD(P)H dehydrogenase [quinone] 1 OS=Mus musculus OX=10090 GN=Nqo1 PE=1 SV=3	31 kDa	2
NAD(P)H dehydrogenase, quinone 2, isoform CRA_b OS=Mus musculus OX=10090 GN=Nqo2 PE=1 SV=1	21 kDa	4
NAD(P)H-hydrate epimerase OS=Mus musculus OX=10090 GN=Naxe PE=1 SV=1	31 kDa	6

NAD-dependent malic enzyme, mitochondrial OS=Mus musculus OX=10090 GN=Me2 PE=1 SV=1	66 kDa	9
NAD-dependent protein deacylase sirtuin-5, mitochondrial OS=Mus musculus OX=10090 GN=Sirt5 PE=1 SV=1	32 kDa	1
NADH dehydrogenase [ubiquinone] 1 alpha subcomplex assembly factor 2 OS=Mus musculus OX=10090 GN=Ndufaf2 PE=1 SV=1	20 kDa	4
NADH dehydrogenase [ubiquinone] 1 alpha subcomplex assembly factor 8 OS=Mus musculus OX=10090 GN=Ndufaf8 PE=1 SV=1	9 kDa	1
NADH dehydrogenase [ubiquinone] 1 alpha subcomplex subunit 12 OS=Mus musculus OX=10090 GN=Ndufa12 PE=1 SV=2	17 kDa	1
NADH dehydrogenase [ubiquinone] 1 alpha subcomplex subunit 13 OS=Mus musculus OX=10090 GN=Ndufa13 PE=1 SV=3	17 kDa	1
NADH dehydrogenase [ubiquinone] 1 alpha subcomplex subunit 2 OS=Mus musculus OX=10090 GN=Ndufa2 PE=1 SV=3	11 kDa	2
NADH dehydrogenase [ubiquinone] 1 alpha subcomplex subunit 5 OS=Mus musculus OX=10090 GN=Ndufa5 PE=1 SV=3	13 kDa	1
NADH dehydrogenase [ubiquinone] 1 alpha subcomplex subunit 6 OS=Mus musculus OX=10090 GN=Ndufa6 PE=1 SV=1	15 kDa	2
NADH dehydrogenase [ubiquinone] 1 alpha subcomplex subunit 7 OS=Mus musculus OX=10090 GN=Ndufa7 PE=1 SV=3	13 kDa	2
NADH dehydrogenase [ubiquinone] 1 alpha subcomplex subunit 8 OS=Mus musculus OX=10090 GN=Ndufa8 PE=1 SV=3	20 kDa	3
NADH dehydrogenase [ubiquinone] 1 beta subcomplex subunit 10 (Fragment) OS=Mus musculus OX=10090 GN=Ndufb10 PE=1 SV=1	18 kDa	1
NADH dehydrogenase [ubiquinone] 1 beta subcomplex subunit 11, mitochondrial OS=Mus musculus OX=10090 GN=Ndufb11 PE=1 SV=2	17 kDa	1
NADH dehydrogenase [ubiquinone] 1 subunit C2 OS=Mus musculus OX=10090 GN=Ndufc2 PE=1 SV=1	14 kDa	1
NADH dehydrogenase [ubiquinone] flavoprotein 1, mitochondrial OS=Mus musculus OX=10090 GN=Ndufv1 PE=1 SV=1	50 kDa	2
NADH dehydrogenase [ubiquinone] flavoprotein 2, mitochondrial OS=Mus musculus OX=10090 GN=Ndufv2 PE=1 SV=2	27 kDa	8
NADH dehydrogenase [ubiquinone] iron-sulfur protein 3, mitochondrial OS=Mus musculus OX=10090 GN=Ndufs3 PE=1 SV=2	30 kDa	5

NADH dehydrogenase [ubiquinone] iron-sulfur protein 4, mitochondrial OS=Mus musculus OX=10090 GN=Ndufs4 PE=1 SV=1	20 kDa	2
NADH dehydrogenase [ubiquinone] iron-sulfur protein 6, mitochondrial OS=Mus musculus OX=10090 GN=Ndufs6 PE=1 SV=1	14 kDa	3
NADH dehydrogenase [ubiquinone] iron-sulfur protein 7, mitochondrial OS=Mus musculus OX=10090 GN=Ndufs7 PE=1 SV=1	25 kDa	1
NADH dehydrogenase [ubiquinone] iron-sulfur protein 8, mitochondrial OS=Mus musculus OX=10090 GN=Ndufs8 PE=1 SV=1	24 kDa	1
NADH-cytochrome b5 reductase 3 (Fragment) OS=Mus musculus OX=10090 GN=Cyb5r3 PE=1 SV=6	16 kDa	6
NADH-ubiquinone oxidoreductase 75 kDa subunit, mitochondrial OS=Mus musculus OX=10090 GN=Ndufs1 PE=1 SV=2	80 kDa	1
NADP-dependent malic enzyme OS=Mus musculus OX=10090 GN=Me1 PE=1 SV=2	64 kDa	12
NADPH:adrenodoxin oxidoreductase, mitochondrial OS=Mus musculus OX=10090 GN=Fdxr PE=1 SV=1	54 kDa	1
NADPH--cytochrome P450 reductase OS=Mus musculus OX=10090 GN=Por PE=1 SV=1	73 kDa	5
N-alpha-acetyltransferase 20 OS=Mus musculus OX=10090 GN=Naa20 PE=1 SV=1	20 kDa	2
N-alpha-acetyltransferase 25, NatB auxiliary subunit OS=Mus musculus OX=10090 GN=Naa25 PE=1 SV=1	112 kDa	1
Nap1l4 protein OS=Mus musculus OX=10090 GN=Nap1l4 PE=1 SV=1	44 kDa	23
Nardilysin, N-arginine dibasic convertase, NRD convertase 1 OS=Mus musculus OX=10090 GN=Nrd1 PE=1 SV=1	141 kDa	16
Nascent polypeptide-associated complex subunit alpha, muscle-specific form OS=Mus musculus OX=10090 GN=Naca PE=1 SV=2	220 kDa	30
NEDD8 OS=Homo sapiens GN=NEDD8 PE=1 SV=1	9 kDa	3
NEDD8-activating enzyme E1 catalytic subunit OS=Mus musculus OX=10090 GN=Uba3 PE=1 SV=2	52 kDa	4
NEDD8-activating enzyme E1 regulatory subunit OS=Mus musculus OX=10090 GN=Nae1 PE=1 SV=1	58 kDa	5
NEDD8-conjugating enzyme Ubc12 (Fragment) OS=Mus musculus OX=10090 GN=Ube2m PE=1 SV=1	20 kDa	2
Negative elongation factor E (Fragment) OS=Mus musculus OX=10090 GN=Nelfe PE=1 SV=1	26 kDa	1
Negative regulator of P-body association OS=Mus musculus OX=10090 GN=Nbdy PE=4 SV=1	7 kDa	1
Nestin OS=Mus musculus OX=10090 GN=Nes PE=1 SV=1	207 kDa	16
Neudesin OS=Mus musculus OX=10090 GN=Nenf PE=1 SV=1	19 kDa	3
Neural Wiskott-Aldrich syndrome protein OS=Mus musculus OX=10090 GN=Wasl PE=1 SV=1	54 kDa	1

Neurofilament heavy polypeptide OS=Mus musculus OX=10090 GN=Nefh PE=1 SV=3	117 kDa	2
Neurolysin, mitochondrial OS=Mus musculus OX=10090 GN=Nln PE=1 SV=1	80 kDa	5
Neuronal pentraxin receptor OS=Mus musculus OX=10090 GN=Nptxr PE=1 SV=2	52 kDa	1
Neuronal pentraxin-1 OS=Mus musculus OX=10090 GN=Nptx1 PE=1 SV=1	47 kDa	2
Neuropilin-2 OS=Mus musculus OX=10090 GN=Nrp2 PE=1 SV=2	105 kDa	1
Neuroplastin (Fragment) OS=Mus musculus OX=10090 GN=Nptn PE=1 SV=1	24 kDa	7
Neutral alpha-glucosidase AB OS=Mus musculus OX=10090 GN=Ganab PE=1 SV=1	107 kDa	67
Neutral cholesterol ester hydrolase 1 OS=Mus musculus OX=10090 GN=Nceh1 PE=1 SV=1	46 kDa	5
NF-kappa-B-activating protein OS=Mus musculus OX=10090 GN=Nkap PE=2 SV=1	47 kDa	1
NFU1 iron-sulfur cluster scaffold homolog, mitochondrial OS=Mus musculus OX=10090 GN=Nfu1 PE=1 SV=1	29 kDa	2
NHL repeat-containing protein 2 OS=Mus musculus OX=10090 GN=Nhlrc2 PE=1 SV=1	78 kDa	3
NHP2-like protein 1 OS=Mus musculus OX=10090 GN=Snu13 PE=1 SV=4	14 kDa	9
Nicastrin OS=Mus musculus OX=10090 GN=Ncstn PE=1 SV=3	78 kDa	5
Nicotinamide phosphoribosyltransferase OS=Mus musculus OX=10090 GN=Nampt PE=1 SV=1	55 kDa	16
Nidogen-1 OS=Mus musculus OX=10090 GN=Nid1 PE=1 SV=2	137 kDa	4
NIF3-like protein 1 OS=Mus musculus OX=10090 GN=Nif3l1 PE=1 SV=4	42 kDa	8
Nodal modulator 1 OS=Mus musculus OX=10090 GN=Nomo1 PE=1 SV=1	133 kDa	5
Non-histone chromosomal protein HMG-14 OS=Mus musculus OX=10090 GN=Hmgn1 PE=1 SV=2	10 kDa	12
Non-histone chromosomal protein HMG-17 OS=Mus musculus OX=10090 GN=Hmgn2 PE=1 SV=1	10 kDa	27
Non-POU domain-containing octamer-binding protein OS=Mus musculus OX=10090 GN=Nono PE=1 SV=3	55 kDa	19
Non-specific lipid-transfer protein OS=Mus musculus OX=10090 GN=Scp2 PE=1 SV=3	59 kDa	8
Non-specific serine/threonine protein kinase OS=Mus musculus OX=10090 GN=Pak1 PE=1 SV=1	61 kDa	5
Non-structural maintenance of chromosomes element 3 homolog OS=Mus musculus OX=10090 GN=Nsmce3 PE=1 SV=1	31 kDa	1
Notchless protein homolog 1 OS=Mus musculus OX=10090 GN=Nle1 PE=1 SV=4	53 kDa	9

NPC intracellular cholesterol transporter 1 OS=Mus musculus OX=10090 GN=Npc1 PE=1 SV=2	143 kDa	1
NPC intracellular cholesterol transporter 2 OS=Mus musculus OX=10090 GN=Npc2 PE=1 SV=1	16 kDa	11
NSFL1 cofactor p47 OS=Mus musculus OX=10090 GN=Nsfl1c PE=1 SV=1	41 kDa	31
Nuclear autoantigenic sperm protein OS=Mus musculus OX=10090 GN=Nasp PE=1 SV=1	84 kDa	23
Nuclear distribution protein nudE homolog 1 OS=Mus musculus OX=10090 GN=Nde1 PE=1 SV=1	39 kDa	1
Nuclear export mediator factor Nemf OS=Mus musculus OX=10090 GN=Nemf PE=1 SV=2	121 kDa	5
Nuclear factor 1 (Fragment) OS=Mus musculus OX=10090 GN=Nfix PE=1 SV=1	53 kDa	2
Nuclear factor 1 OS=Mus musculus OX=10090 GN=Nfic PE=1 SV=1	51 kDa	5
Nuclear fragile X mental retardation-interacting protein 2 OS=Mus musculus OX=10090 GN=Nufip2 PE=1 SV=1	76 kDa	1
Nuclear migration protein nudC OS=Mus musculus OX=10090 GN=Nudc PE=1 SV=1	38 kDa	35
Nuclear mitotic apparatus protein 1 OS=Mus musculus OX=10090 GN=Numa1 PE=1 SV=1	236 kDa	13
Nuclear pore complex protein Nup214 OS=Mus musculus OX=10090 GN=Nup214 PE=1 SV=2	213 kDa	3
Nuclear pore complex protein Nup50 OS=Mus musculus OX=10090 GN=Nup50 PE=1 SV=3	49 kDa	6
Nuclear pore complex protein Nup54 OS=Mus musculus OX=10090 GN=Nup54 PE=1 SV=1	56 kDa	1
Nuclear pore complex protein Nup88 OS=Mus musculus OX=10090 GN=Nup88 PE=1 SV=1	85 kDa	1
Nuclear pore complex-associated intranuclear coiled-coil protein TPR OS=Mus musculus OX=10090 GN=Tpr PE=1 SV=1	267 kDa	60
Nuclear pore glycoprotein p62 OS=Mus musculus OX=10090 GN=Nup62 PE=1 SV=2	53 kDa	3
Nuclear protein 1 OS=Mus musculus OX=10090 GN=Nupr1 PE=3 SV=1	9 kDa	1
Nuclear protein localization protein 4 homolog OS=Mus musculus OX=10090 GN=Nploc4 PE=1 SV=3	68 kDa	5
Nuclear receptor-binding protein OS=Mus musculus OX=10090 GN=Nrbp1 PE=1 SV=1	61 kDa	2
Nuclear transport factor 2 OS=Mus musculus OX=10090 GN=Nutf2 PE=1 SV=1	14 kDa	3
Nuclear ubiquitous casein and cyclin-dependent kinase substrate 1 OS=Mus musculus OX=10090 GN=Nucks1 PE=1 SV=1	26 kDa	15
Nuclear valosin-containing protein-like OS=Mus musculus OX=10090 GN=Nvl PE=1 SV=1	94 kDa	1
Nuclear-interacting partner of ALK OS=Mus musculus OX=10090 GN=Zc3hc1 PE=1 SV=1	51 kDa	2

Nuclease-sensitive element-binding protein 1 OS=Mus musculus OX=10090 GN=Ybx1 PE=1 SV=3	36 kDa	43
Nucleobindin-1 OS=Mus musculus OX=10090 GN=Nucb1 PE=1 SV=2	53 kDa	11
Nucleobindin-2 OS=Mus musculus OX=10090 GN=Nucb2 PE=1 SV=2	50 kDa	4
Nucleolar and coiled-body phosphoprotein 1 OS=Mus musculus OX=10090 GN=Nolc1 PE=1 SV=1	73 kDa	37
Nucleolar GTP-binding protein 1 OS=Mus musculus OX=10090 GN=Gtpbp4 PE=1 SV=3	74 kDa	2
Nucleolar protein 16 OS=Mus musculus OX=10090 GN=Nop16 PE=1 SV=1	21 kDa	2
Nucleolar protein 56 (Fragment) OS=Mus musculus OX=10090 GN=Nop56 PE=1 SV=1	24 kDa	6
Nucleolar protein 56 OS=Mus musculus OX=10090 GN=Nop56 PE=1 SV=2	64 kDa	8
Nucleolar protein 58 OS=Mus musculus OX=10090 GN=Nop58 PE=1 SV=1	60 kDa	12
Nucleolar RNA helicase 2 OS=Mus musculus OX=10090 GN=Ddx21 PE=1 SV=3	94 kDa	34
Nucleolar transcription factor 1 OS=Mus musculus OX=10090 GN=Ubtf PE=1 SV=1	89 kDa	2
Nucleolar transcription factor 1 OS=Mus musculus OX=10090 GN=Ubtf PE=1 SV=1	88 kDa	2
Nucleolin (Fragment) OS=Mus musculus OX=10090 GN=Ncl PE=1 SV=1	8 kDa	10
Nucleolin OS=Mus musculus OX=10090 GN=Ncl PE=1 SV=2	77 kDa	130
Nucleolysin TIA-1 OS=Mus musculus OX=10090 GN=Tia1 PE=1 SV=1	43 kDa	1
Nucleolysin TIAR OS=Mus musculus OX=10090 GN=Tial1 PE=1 SV=1	27 kDa	1
Nucleophosmin OS=Mus musculus OX=10090 GN=Npm1 PE=1 SV=1	33 kDa	84
Nucleoplasmin-3 OS=Mus musculus OX=10090 GN=Npm3 PE=1 SV=3	19 kDa	6
Nucleoporin 153 OS=Mus musculus OX=10090 GN=Nup153 PE=1 SV=1	152 kDa	1
Nucleoporin Nup37 OS=Mus musculus OX=10090 GN=Nup37 PE=1 SV=2	37 kDa	3
Nucleoporin Nup43 OS=Mus musculus OX=10090 GN=Nup43 PE=1 SV=2	42 kDa	3
Nucleoporin p58/p45 OS=Mus musculus OX=10090 GN=Nup58 PE=1 SV=1	59 kDa	1
Nucleoporin SEH1 OS=Mus musculus OX=10090 GN=Seh1l PE=2 SV=1	40 kDa	2
Nucleoredoxin OS=Mus musculus OX=10090 GN=Nxn PE=1 SV=1	48 kDa	2
Nucleoside diphosphate kinase A OS=Mus musculus OX=10090 GN=Nme1 PE=1 SV=1	17 kDa	80

Nucleoside diphosphate kinase OS=Mus musculus OX=10090 GN=Gm20390 PE=3 SV=1	30 kDa	99
Nucleoside diphosphate kinase, mitochondrial OS=Mus musculus OX=10090 GN=Nme4 PE=1 SV=1	21 kDa	1
Nucleosome assembly protein 1-like 1 OS=Mus musculus OX=10090 GN=Nap1l1 PE=1 SV=1	49 kDa	26
NudC domain-containing protein 1 OS=Mus musculus OX=10090 GN=Nudcd1 PE=1 SV=2	67 kDa	4
NudC domain-containing protein 2 OS=Mus musculus OX=10090 GN=Nudcd2 PE=1 SV=1	15 kDa	3
NudC domain-containing protein 3 (Fragment) OS=Mus musculus OX=10090 GN=Nudcd3 PE=1 SV=1	28 kDa	2
Obg-like ATPase 1 OS=Mus musculus OX=10090 GN=Ola1 PE=1 SV=1	45 kDa	2
OCIA domain-containing protein 1 OS=Mus musculus OX=10090 GN=Ociad1 PE=1 SV=1	28 kDa	3
Olfactory receptor OS=Mus musculus OX=10090 GN=Olfr1419 PE=2 SV=1	38 kDa	1
Oligoribonuclease, mitochondrial OS=Mus musculus OX=10090 GN=Rexo2 PE=1 SV=2	27 kDa	6
Oligosaccharyltransferase complex subunit OSTC OS=Mus musculus OX=10090 GN=Ostc PE=1 SV=1	17 kDa	1
Omega-amidase NIT2 OS=Mus musculus OX=10090 GN=Nit2 PE=1 SV=1	31 kDa	8
Opioid growth factor receptor OS=Mus musculus OX=10090 GN=Ogfr PE=1 SV=1	71 kDa	6
Ornithine aminotransferase, mitochondrial OS=Mus musculus OX=10090 GN=Oat PE=1 SV=1	48 kDa	24
Osteoclast-stimulating factor 1 OS=Mus musculus OX=10090 GN=Ostf1 PE=1 SV=2	24 kDa	1
Osteopontin OS=Mus musculus OX=10090 GN=Spp1 PE=1 SV=1	33 kDa	1
Oxygen-dependent coproporphyrinogen-III oxidase, mitochondrial OS=Mus musculus OX=10090 GN=Cpox PE=1 SV=2	50 kDa	4
Oxysterol-binding protein 1 OS=Mus musculus OX=10090 GN=Osbp PE=1 SV=3	89 kDa	2
p21-activated protein kinase-interacting protein 1 OS=Mus musculus OX=10090 GN=Pak1ip1 PE=1 SV=2	42 kDa	1
Pachytene checkpoint protein 2 homolog OS=Mus musculus OX=10090 GN=Trip13 PE=1 SV=1	48 kDa	7
Paired mesoderm homeobox protein 1 OS=Mus musculus OX=10090 GN=Prrx1 PE=1 SV=1	23 kDa	2
Paired mesoderm homeobox protein 2 OS=Mus musculus OX=10090 GN=Prrx2 PE=2 SV=1	20 kDa	1
Palmitoyl-protein thioesterase 1 OS=Mus musculus OX=10090 GN=Ppt1 PE=1 SV=2	34 kDa	3
Papillary Renal Cell carcinoma (translocation-associated) OS=Mus musculus OX=10090 GN=Prcc PE=1 SV=1	52 kDa	3

Paralemmin-1 OS=Mus musculus OX=10090 GN=Palm PE=1 SV=1	42 kDa	1
Paraspeckle component 1 OS=Mus musculus OX=10090 GN=Pspc1 PE=1 SV=1	59 kDa	1
Parathymosin OS=Mus musculus OX=10090 GN=Ptms PE=1 SV=3	11 kDa	15
Partner of Y14 and mago OS=Mus musculus OX=10090 GN=Pym1 PE=1 SV=2	23 kDa	5
Paxillin OS=Mus musculus OX=10090 GN=Pxn PE=1 SV=1	65 kDa	3
Paxillin OS=Mus musculus OX=10090 GN=Pxn PE=1 SV=1	47 kDa	1
PC4 and SFRS1-interacting protein OS=Mus musculus OX=10090 GN=Psip1 PE=1 SV=1	60 kDa	19
PDZ and LIM domain protein 1 OS=Mus musculus OX=10090 GN=Pdlim1 PE=1 SV=4	36 kDa	51
PDZ and LIM domain protein 2 OS=Mus musculus OX=10090 GN=Pdlim2 PE=1 SV=1	38 kDa	10
PDZ and LIM domain protein 5 OS=Mus musculus OX=10090 GN=Pdlim5 PE=1 SV=4	63 kDa	24
Pentatricopeptide repeat domain-containing protein 3, mitochondrial OS=Mus musculus OX=10090 GN=Ptcd3 PE=1 SV=2	78 kDa	1
Peptidyl-prolyl cis-trans isomerase A OS=Mus musculus OX=10090 GN=Ppia PE=1 SV=2	18 kDa	168
Peptidyl-prolyl cis-trans isomerase B OS=Mus musculus OX=10090 GN=Ppib PE=1 SV=2	24 kDa	15
Peptidyl-prolyl cis-trans isomerase C OS=Mus musculus OX=10090 GN=Ppic PE=1 SV=1	23 kDa	3
Peptidyl-prolyl cis-trans isomerase D OS=Mus musculus OX=10090 GN=Ppid PE=1 SV=3	41 kDa	11
Peptidyl-prolyl cis-trans isomerase F, mitochondrial OS=Mus musculus OX=10090 GN=Ppif PE=1 SV=1	22 kDa	2
Peptidyl-prolyl cis-trans isomerase FKBP10 OS=Mus musculus OX=10090 GN=Fkbp10 PE=1 SV=2	65 kDa	17
Peptidyl-prolyl cis-trans isomerase FKBP1A OS=Mus musculus OX=10090 GN=Fkbp1a PE=1 SV=2	12 kDa	20
Peptidyl-prolyl cis-trans isomerase FKBP2 OS=Mus musculus OX=10090 GN=Fkbp2 PE=1 SV=1	15 kDa	4
Peptidyl-prolyl cis-trans isomerase FKBP3 OS=Mus musculus OX=10090 GN=Fkbp3 PE=1 SV=2	25 kDa	35
Peptidyl-prolyl cis-trans isomerase FKBP4 OS=Mus musculus OX=10090 GN=Fkbp4 PE=1 SV=5	52 kDa	37
Peptidyl-prolyl cis-trans isomerase FKBP5 OS=Mus musculus OX=10090 GN=Fkbp5 PE=1 SV=1	51 kDa	2
Peptidyl-prolyl cis-trans isomerase FKBP9 OS=Mus musculus OX=10090 GN=Fkbp9 PE=1 SV=1	63 kDa	5
Peptidyl-prolyl cis-trans isomerase NIMA-interacting 1 OS=Mus musculus OX=10090 GN=Pin1 PE=1 SV=1	18 kDa	7
Peptidyl-prolyl cis-trans isomerase NIMA-interacting 4 OS=Mus musculus OX=10090 GN=Pin4 PE=1 SV=1	14 kDa	3

Peptidyl-prolyl cis-trans isomerase-like 1 OS=Mus musculus OX=10090 GN=Ppil1 PE=1 SV=1	18 kDa	3
Peptidyl-tRNA hydrolase 2, mitochondrial OS=Mus musculus OX=10090 GN=Ptrh2 PE=1 SV=1	20 kDa	4
Perilipin-3 OS=Mus musculus OX=10090 GN=Plin3 PE=1 SV=1	47 kDa	39
Peroxidasin homolog OS=Mus musculus OX=10090 GN=Pxdn PE=1 SV=1	144 kDa	1
Peroxiredoxin-1 OS=Mus musculus OX=10090 GN=Prdx1 PE=1 SV=1	22 kDa	81
Peroxiredoxin-2 OS=Mus musculus OX=10090 GN=Prdx2 PE=1 SV=3	22 kDa	35
Peroxiredoxin-4 OS=Mus musculus OX=10090 GN=Prdx4 PE=1 SV=1	31 kDa	40
Peroxiredoxin-5, mitochondrial OS=Mus musculus OX=10090 GN=Prdx5 PE=1 SV=2	22 kDa	31
Peroxiredoxin-6 OS=Mus musculus OX=10090 GN=Prdx6 PE=1 SV=1	25 kDa	55
Peroxiredoxin-6 OS=Mus musculus OX=10090 GN=Prdx6 PE=1 SV=1	9 kDa	3
Peroxisomal membrane protein PEX14 OS=Mus musculus OX=10090 GN=Pex14 PE=1 SV=1	41 kDa	6
Peroxisomal multifunctional enzyme type 2 OS=Mus musculus OX=10090 GN=Hsd17b4 PE=1 SV=3	79 kDa	15
Peroxisomal trans-2-enoyl-CoA reductase OS=Mus musculus OX=10090 GN=Pecr PE=1 SV=1	32 kDa	1
Persulfide dioxygenase ETHE1, mitochondrial OS=Mus musculus OX=10090 GN=Ethe1 PE=1 SV=2	28 kDa	8
Pescadillo homolog OS=Mus musculus OX=10090 GN=Pes1 PE=1 SV=1	68 kDa	4
PEST proteolytic signal-containing nuclear protein OS=Mus musculus OX=10090 GN=Pcnp PE=1 SV=1	19 kDa	21
Pet2 protein OS=Mus musculus OX=10090 GN=Pet2 PE=1 SV=1	83 kDa	1
PHD finger protein 23 (Fragment) OS=Mus musculus OX=10090 GN=Phf23 PE=1 SV=1	21 kDa	1
PHD finger protein 3 OS=Mus musculus OX=10090 GN=Phf3 PE=1 SV=1	226 kDa	2
PHD finger-like domain-containing protein 5A OS=Mus musculus OX=10090 GN=Phf5a PE=1 SV=1	12 kDa	2
Phenylalanine--tRNA ligase alpha subunit OS=Mus musculus OX=10090 GN=Farsa PE=1 SV=1	57 kDa	5
Phenylalanine--tRNA ligase beta subunit (Fragment) OS=Mus musculus OX=10090 GN=Farsb PE=1 SV=1	23 kDa	4
Phenylalanine--tRNA ligase beta subunit OS=Mus musculus OX=10090 GN=Farsb PE=1 SV=2	66 kDa	6
Phosducin-like protein (Fragment) OS=Mus musculus OX=10090 GN=Pdcl PE=1 SV=1	23 kDa	1
Phosducin-like protein 3 OS=Mus musculus OX=10090 GN=Pdcl3 PE=1 SV=1	28 kDa	1

Phosphatase and actin regulator 4 (Fragment) OS=Mus musculus OX=10090 GN=Phactr4 PE=1 SV=1	31 kDa	2
Phosphatase and actin regulator 4 OS=Mus musculus OX=10090 GN=Phactr4 PE=1 SV=2	77 kDa	4
Phosphate carrier protein, mitochondrial OS=Mus musculus OX=10090 GN=Slc25a3 PE=1 SV=1	40 kDa	14
Phosphatidylethanolamine-binding protein 1 OS=Mus musculus OX=10090 GN=Pebp1 PE=1 SV=3	21 kDa	43
Phosphatidylinositol 5-phosphate 4-kinase type-2 beta OS=Mus musculus OX=10090 GN=Pip4k2b PE=1 SV=1	47 kDa	3
Phosphatidylinositol transfer protein alpha isoform OS=Mus musculus OX=10090 GN=Pitpna PE=1 SV=1	32 kDa	1
Phosphatidylinositol transfer protein beta isoform OS=Mus musculus OX=10090 GN=Pitpnb PE=1 SV=2	31 kDa	2
Phosphatidylinositol-binding clathrin assembly protein (Fragment) OS=Mus musculus OX=10090 GN=Picalm PE=1 SV=1	57 kDa	2
Phosphatidylinositol-binding clathrin assembly protein (Fragment) OS=Mus musculus OX=10090 GN=Picalm PE=1 SV=1	10 kDa	1
Phosphoacetylglucosamine mutase OS=Mus musculus OX=10090 GN=Pgm3 PE=1 SV=1	59 kDa	6
Phosphoenolpyruvate carboxykinase [GTP], mitochondrial OS=Mus musculus OX=10090 GN=Pck2 PE=1 SV=1	73 kDa	19
Phosphoenolpyruvate carboxykinase, cytosolic [GTP] OS=Mus musculus OX=10090 GN=Pck1 PE=1 SV=1	69 kDa	1
Phosphofurin acidic cluster sorting protein 1 OS=Mus musculus OX=10090 GN=Pacs1 PE=1 SV=2	105 kDa	1
Phosphoglucomutase-1 OS=Mus musculus OX=10090 GN=Pgm1 PE=1 SV=4	61 kDa	34
Phosphoglucomutase-2 OS=Mus musculus OX=10090 GN=Pgm2 PE=1 SV=1	69 kDa	12
Phosphoglycerate kinase 1 OS=Mus musculus OX=10090 GN=Pgk1 PE=1 SV=4	45 kDa	76
Phosphoglycerate mutase 1 OS=Mus musculus OX=10090 GN=Pgam1 PE=1 SV=3	29 kDa	139
Phosphoglycerate mutase 2 OS=Mus musculus OX=10090 GN=Pgam2 PE=1 SV=3	29 kDa	41
Phospholipase A-2-activating protein OS=Mus musculus OX=10090 GN=Plaa PE=1 SV=4	87 kDa	12
Phosphomannomutase 2 OS=Mus musculus OX=10090 GN=Pmm2 PE=1 SV=1	28 kDa	1
Phosphopantothenate--cysteine ligase OS=Mus musculus OX=10090 GN=Ppcs PE=1 SV=1	34 kDa	1
Phosphoribosyl pyrophosphate synthase-associated protein 2 OS=Mus musculus OX=10090 GN=Prpsap2 PE=1 SV=1	41 kDa	9
Phosphoribosyl pyrophosphate synthetase 1-like 3 OS=Mus musculus OX=10090 GN=Prps1l3 PE=3 SV=1	35 kDa	18
Phosphoribosylformylglycinamidine synthase OS=Mus musculus OX=10090 GN=Pfas PE=1 SV=1	145 kDa	9

Phosphoserine aminotransferase OS=Mus musculus OX=10090 GN=Psat1 PE=1 SV=1	40 kDa	63
Phosphoserine phosphatase OS=Mus musculus OX=10090 GN=Psph PE=1 SV=1	25 kDa	13
Phosphotriesterase-related protein (Fragment) OS=Mus musculus OX=10090 GN=Pter PE=1 SV=2	36 kDa	2
Phostensin OS=Mus musculus OX=10090 GN=Ppp1r18 PE=1 SV=1	66 kDa	8
Pinin OS=Mus musculus OX=10090 GN=Pnn PE=1 SV=4	82 kDa	5
Pirin (Fragment) OS=Mus musculus OX=10090 GN=Pir PE=1 SV=1	28 kDa	1
PITH domain-containing protein 1 (Fragment) OS=Mus musculus OX=10090 GN=Pithd1 PE=1 SV=1	14 kDa	1
Pituitary tumor-transforming gene 1 protein-interacting protein (Fragment) OS=Mus musculus OX=10090 GN=Pttg1ip PE=1 SV=1	13 kDa	4
Plasma membrane calcium-transporting ATPase 1 OS=Mus musculus OX=10090 GN=Atp2b1 PE=1 SV=1	135 kDa	3
Plasminogen activator inhibitor 1 RNA-binding protein OS=Mus musculus OX=10090 GN=Serbp1 PE=1 SV=2	45 kDa	71
Plasminogen receptor (KT) OS=Mus musculus OX=10090 GN=Plgrkt PE=1 SV=1	17 kDa	2
Plastin-2 OS=Mus musculus OX=10090 GN=Lcp1 PE=1 SV=4	70 kDa	7
Plastin-3 (Fragment) OS=Mus musculus OX=10090 GN=Pls3 PE=1 SV=1	71 kDa	29
Platelet-activating factor acetylhydrolase IB subunit alpha OS=Mus musculus OX=10090 GN=Pafah1b1 PE=1 SV=2	47 kDa	23
Platelet-activating factor acetylhydrolase IB subunit beta (Fragment) OS=Mus musculus OX=10090 GN=Pafah1b2 PE=1 SV=1	22 kDa	6
Platelet-activating factor acetylhydrolase IB subunit gamma OS=Mus musculus OX=10090 GN=Pafah1b3 PE=1 SV=1	26 kDa	6
Plectin OS=Mus musculus OX=10090 GN=Plec PE=1 SV=1	499 kDa	46
Pleiotropic regulator 1 OS=Mus musculus OX=10090 GN=Plrg1 PE=1 SV=1	56 kDa	2
Plexin-B2 OS=Mus musculus OX=10090 GN=Plxnb2 PE=1 SV=1	206 kDa	4
Poliovirus receptor OS=Mus musculus OX=10090 GN=Pvr PE=1 SV=1	45 kDa	1
Poly [ADP-ribose] polymerase OS=Mus musculus OX=10090 GN=Parp1 PE=1 SV=1	113 kDa	7
Poly [ADP-ribose] polymerase OS=Mus musculus OX=10090 GN=Parp3 PE=1 SV=1	60 kDa	1
Poly A binding protein, cytoplasmic 5 OS=Mus musculus OX=10090 GN=Pabpc5 PE=2 SV=1	43 kDa	3
Poly(A)-specific ribonuclease PARN OS=Mus musculus OX=10090 GN=Parn PE=1 SV=1	72 kDa	1

Poly(rC)-binding protein 1 OS=Mus musculus OX=10090 GN=Pcbp1 PE=1 SV=1	37 kDa	33
Poly(rC)-binding protein 2 OS=Mus musculus OX=10090 GN=Pcbp2 PE=1 SV=1	38 kDa	24
Poly(rC)-binding protein 3 OS=Mus musculus OX=10090 GN=Pcbp3 PE=1 SV=3	39 kDa	17
Poly(U)-binding-splicing factor PUF60 OS=Mus musculus OX=10090 GN=Puf60 PE=1 SV=2	60 kDa	19
Polyadenylate-binding protein 1 OS=Mus musculus OX=10090 GN=Pabpc1 PE=1 SV=2	71 kDa	38
Polyadenylate-binding protein OS=Mus musculus OX=10090 GN=Pabpc1l PE=2 SV=1	67 kDa	4
Polyadenylate-binding protein OS=Mus musculus OX=10090 GN=Pabpc4 PE=1 SV=1	69 kDa	17
Polyadenylate-binding protein-interacting protein 1 OS=Mus musculus OX=10090 GN=Paip1 PE=1 SV=1	47 kDa	4
Polyadenylate-binding protein-interacting protein 1 OS=Mus musculus OX=10090 GN=Paip1 PE=1 SV=2	54 kDa	4
Polymerase delta-interacting protein 2 (Fragment) OS=Mus musculus OX=10090 GN=Poldip2 PE=1 SV=1	33 kDa	1
Polymerase delta-interacting protein 3 OS=Mus musculus OX=10090 GN=Poldip3 PE=1 SV=1	46 kDa	1
Polynucleotide 5'-hydroxyl-kinase NOL9 OS=Mus musculus OX=10090 GN=Nol9 PE=1 SV=1	81 kDa	1
Polypyrimidine tract-binding protein 3 OS=Mus musculus OX=10090 GN=Ptbp3 PE=1 SV=1	60 kDa	3
Polyribonucleotide nucleotidyltransferase 1, mitochondrial OS=Mus musculus OX=10090 GN=Pnpt1 PE=1 SV=1	86 kDa	1
Porphobilinogen deaminase OS=Mus musculus OX=10090 GN=Hmbs PE=1 SV=2	39 kDa	2
Ppm1b protein OS=Mus musculus OX=10090 GN=Ppm1b PE=1 SV=1	52 kDa	3
PRA1 family protein 3 OS=Mus musculus OX=10090 GN=Arl6ip5 PE=1 SV=2	22 kDa	4
Predicted gene 10036 OS=Mus musculus OX=10090 GN=Gm10036 PE=3 SV=1	20 kDa	16
Predicted gene 10439 OS=Mus musculus OX=10090 GN=Gm10439 PE=4 SV=1	51 kDa	1
Predicted gene 16381 OS=Mus musculus OX=10090 GN=Gm16381 PE=4 SV=1	9 kDa	1
Predicted gene 17087 OS=Mus musculus OX=10090 GN=Gm17087 PE=1 SV=1	18 kDa	19
Predicted gene 17190 OS=Mus musculus OX=10090 GN=Gm17190 PE=1 SV=1	38 kDa	4
Predicted gene 20425 OS=Mus musculus OX=10090 GN=Gm20425 PE=4 SV=1	108 kDa	2
Predicted gene 20498 OS=Mus musculus OX=10090 GN=Gm20498 PE=1 SV=1	20 kDa	1
Predicted gene 43738 OS=Mus musculus OX=10090 GN=Gm43738 PE=4 SV=1	95 kDa	3

Predicted gene 7075 OS=Mus musculus OX=10090 GN=Gm7075 PE=4 SV=1	13 kDa	1
Predicted gene 7298 OS=Mus musculus OX=10090 GN=Gm7298 PE=4 SV=1	165 kDa	1
Predicted gene 7324 OS=Mus musculus OX=10090 GN=Gm7324 PE=4 SV=1	42 kDa	8
Predicted gene 9803 OS=Mus musculus OX=10090 GN=Gm9803 PE=1 SV=1	11 kDa	3
Predicted gene, 27029 OS=Mus musculus OX=10090 GN=Gm27029 PE=4 SV=1	58 kDa	1
Predicted pseudogene 9242 OS=Mus musculus OX=10090 GN=Gm9242 PE=4 SV=1	37 kDa	45
Prefoldin subunit 1 OS=Mus musculus OX=10090 GN=Pfdn1 PE=1 SV=1	14 kDa	4
Prefoldin subunit 2 OS=Mus musculus OX=10090 GN=Pfdn2 PE=1 SV=2	17 kDa	9
Prefoldin subunit 3 OS=Mus musculus OX=10090 GN=Vbp1 PE=1 SV=2	22 kDa	10
Prefoldin subunit 4 OS=Mus musculus OX=10090 GN=Pfdn4 PE=1 SV=1	15 kDa	2
Prefoldin subunit 6 OS=Mus musculus OX=10090 GN=Pfdn6 PE=1 SV=1	14 kDa	2
Prelamin-A/C OS=Mus musculus OX=10090 GN=Lmna PE=1 SV=2	74 kDa	141
Pre-mRNA 3'-end-processing factor FIP1 OS=Mus musculus OX=10090 GN=Fip1l1 PE=1 SV=1	65 kDa	4
Pre-mRNA-processing factor 19 OS=Mus musculus OX=10090 GN=Prpf19 PE=1 SV=1	55 kDa	38
Pre-mRNA-processing factor 40 homolog A OS=Mus musculus OX=10090 GN=Prpf40a PE=1 SV=1	106 kDa	1
Pre-mRNA-processing-splicing factor 8 OS=Mus musculus OX=10090 GN=Prpf8 PE=1 SV=2	274 kDa	12
Pre-mRNA-splicing factor ATP-dependent RNA helicase DHX15 OS=Mus musculus OX=10090 GN=Dhx15 PE=1 SV=2	91 kDa	25
Pre-mRNA-splicing factor SYF1 OS=Mus musculus OX=10090 GN=Xab2 PE=1 SV=1	100 kDa	1
Prenylated Rab acceptor protein 1 OS=Mus musculus OX=10090 GN=Rabac1 PE=1 SV=1	21 kDa	1
Prenylcysteine oxidase OS=Mus musculus OX=10090 GN=Pcyox1 PE=1 SV=1	56 kDa	4
pre-rRNA processing protein FTSJ3 OS=Mus musculus OX=10090 GN=Ftsj3 PE=1 SV=1	96 kDa	2
Pre-rRNA-processing protein TSR1 homolog OS=Mus musculus OX=10090 GN=Tsr1 PE=1 SV=1	92 kDa	2
Presequence protease, mitochondrial OS=Mus musculus OX=10090 GN=Pitrm1 PE=1 SV=1	117 kDa	47
PRKC apoptosis WT1 regulator protein OS=Mus musculus OX=10090 GN=Pawr PE=1 SV=2	36 kDa	1

Probable 28S rRNA (cytosine-C(5))-methyltransferase OS=Mus musculus OX=10090 GN=Nop2 PE=1 SV=1	87 kDa	7
Probable aminopeptidase NPEPL1 OS=Mus musculus OX=10090 GN=Npepl1 PE=1 SV=1	56 kDa	4
Probable ATP-dependent RNA helicase DDX17 OS=Mus musculus OX=10090 GN=Ddx17 PE=1 SV=1	72 kDa	18
Probable ATP-dependent RNA helicase DDX27 OS=Mus musculus OX=10090 GN=Ddx27 PE=1 SV=3	86 kDa	3
Probable ATP-dependent RNA helicase DDX46 OS=Mus musculus OX=10090 GN=Ddx46 PE=1 SV=2	117 kDa	2
Probable ATP-dependent RNA helicase DDX47 (Fragment) OS=Mus musculus OX=10090 GN=Ddx47 PE=1 SV=1	42 kDa	1
Probable ATP-dependent RNA helicase DDX5 OS=Mus musculus OX=10090 GN=Ddx5 PE=1 SV=2	69 kDa	32
Probable ATP-dependent RNA helicase DDX56 OS=Mus musculus OX=10090 GN=Ddx56 PE=2 SV=1	61 kDa	1
Probable ATP-dependent RNA helicase DDX6 OS=Mus musculus OX=10090 GN=Ddx6 PE=1 SV=1	54 kDa	4
Probable cytosolic iron-sulfur protein assembly protein CIAO1 OS=Mus musculus OX=10090 GN=Ciao1 PE=1 SV=1	38 kDa	1
Probable dimethyladenosine transferase OS=Mus musculus OX=10090 GN=Dimt1 PE=2 SV=1	35 kDa	1
Probable global transcription activator SNF2L2 OS=Mus musculus OX=10090 GN=Smarca2 PE=1 SV=1	173 kDa	1
Probable RNA polymerase II nuclear localization protein SLC7A6OS OS=Mus musculus OX=10090 GN=Slc7a6os PE=1 SV=1	35 kDa	2
Probable RNA-binding protein EIF1AD OS=Mus musculus OX=10090 GN=Eif1ad PE=1 SV=2	20 kDa	2
Probable rRNA-processing protein EBP2 OS=Mus musculus OX=10090 GN=Ebna1bp2 PE=2 SV=1	35 kDa	1
Probable tRNA N6-adenosine threonylcarbamoyltransferase OS=Mus musculus OX=10090 GN=Osgep PE=1 SV=1	36 kDa	1
Probable UDP-sugar transporter protein SLC35A4 OS=Mus musculus OX=10090 GN=Slc35a4 PE=1 SV=1	11 kDa	2
Probable Xaa-Pro aminopeptidase 3 OS=Mus musculus OX=10090 GN=Xpnpep3 PE=1 SV=1	57 kDa	1
Processing of 1, ribonuclease P/MRP family, (S. cerevisiae) OS=Mus musculus OX=10090 GN=Pop1 PE=1 SV=1	117 kDa	4
Procollagen galactosyltransferase 1 OS=Mus musculus OX=10090 GN=Colgalt1 PE=1 SV=2	71 kDa	3
Procollagen galactosyltransferase 2 OS=Mus musculus OX=10090 GN=Colgalt2 PE=1 SV=1	55 kDa	1
Procollagen-lysine,2-oxoglutarate 5-dioxygenase 1 OS=Mus musculus OX=10090 GN=Plod1 PE=1 SV=1	84 kDa	3
Procollagen-lysine,2-oxoglutarate 5-dioxygenase 2 OS=Mus musculus OX=10090 GN=Plod2 PE=1 SV=1	87 kDa	2
Procollagen-lysine,2-oxoglutarate 5-dioxygenase 3 OS=Mus musculus OX=10090 GN=Plod3 PE=1 SV=1	85 kDa	2

Profilin-1 OS=Mus musculus OX=10090 GN=Pfn1 PE=1 SV=2	15 kDa	53
Programmed cell death 6-interacting protein OS=Mus musculus OX=10090 GN=Pdcd6ip PE=1 SV=3	96 kDa	5
Programmed cell death protein 10 OS=Mus musculus OX=10090 GN=Pdcd10 PE=1 SV=1	25 kDa	4
Programmed cell death protein 4 OS=Mus musculus OX=10090 GN=Pdcd4 PE=1 SV=1	52 kDa	1
Programmed cell death protein 5 OS=Mus musculus OX=10090 GN=Pdcd5 PE=1 SV=1	22 kDa	6
Programmed cell death protein 5 OS=Mus musculus OX=10090 GN=Pdcd5 PE=1 SV=3	14 kDa	11
Programmed cell death protein 6 OS=Mus musculus OX=10090 GN=Pdcd6 PE=1 SV=2	22 kDa	10
Prohibitin OS=Mus musculus OX=10090 GN=Phb PE=1 SV=1	30 kDa	7
Prohibitin-2 OS=Mus musculus OX=10090 GN=Phb2 PE=1 SV=1	33 kDa	12
Proliferating cell nuclear antigen OS=Mus musculus OX=10090 GN=Pcna PE=1 SV=2	29 kDa	48
Proliferation marker protein Ki-67 OS=Mus musculus OX=10090 GN=Mki67 PE=1 SV=1	351 kDa	1
Proliferation-associated protein 2G4 OS=Mus musculus OX=10090 GN=Pa2g4 PE=1 SV=3	44 kDa	46
Proline-, glutamic acid- and leucine-rich protein 1 OS=Mus musculus OX=10090 GN=Pelp1 PE=1 SV=2	118 kDa	1
Proline-rich AKT1 substrate 1 OS=Mus musculus OX=10090 GN=Akt1s1 PE=1 SV=1	27 kDa	1
Prolyl 3-hydroxylase 1 OS=Mus musculus OX=10090 GN=P3h1 PE=1 SV=1	84 kDa	1
Prolyl 4-hydroxylase subunit alpha-1 OS=Mus musculus OX=10090 GN=P4ha1 PE=1 SV=1	52 kDa	8
Prolyl 4-hydroxylase subunit alpha-1 OS=Mus musculus OX=10090 GN=P4ha1 PE=1 SV=2	61 kDa	9
Prolyl endopeptidase OS=Mus musculus OX=10090 GN=Prep PE=1 SV=1	81 kDa	12
Propionyl-CoA carboxylase alpha chain, mitochondrial OS=Mus musculus OX=10090 GN=Pcca PE=1 SV=2	80 kDa	1
Propionyl-CoA carboxylase beta chain, mitochondrial (Fragment) OS=Mus musculus OX=10090 GN=Pccb PE=1 SV=1	27 kDa	2
Prosaposin OS=Mus musculus OX=10090 GN=Psap PE=1 SV=1	61 kDa	7
Prostaglandin E synthase 3 OS=Mus musculus OX=10090 GN=Ptges3 PE=1 SV=1	15 kDa	8
Prostaglandin E synthase OS=Mus musculus OX=10090 GN=Ptges PE=1 SV=1	17 kDa	4
Prostaglandin G/H synthase 1 OS=Mus musculus OX=10090 GN=Ptgs1 PE=1 SV=1	69 kDa	9

Prostaglandin G/H synthase 2 OS=Mus musculus OX=10090 GN=Ptgs2 PE=1 SV=1	69 kDa	7
Prostaglandin reductase 1 OS=Mus musculus OX=10090 GN=Ptgr1 PE=1 SV=2	36 kDa	11
Prostaglandin reductase-3 OS=Mus musculus OX=10090 GN=Zadh2 PE=1 SV=1	41 kDa	3
Protease 1 OS=Achromobacter lyticus PE=1 SV=1	68 kDa	1
Proteasome activator complex subunit 1 (Fragment) OS=Mus musculus OX=10090 GN=Psme1 PE=1 SV=1	27 kDa	17
Proteasome activator complex subunit 2 OS=Mus musculus OX=10090 GN=Psme2 PE=1 SV=4	27 kDa	7
Proteasome activator complex subunit 3 OS=Mus musculus OX=10090 GN=Psme3 PE=1 SV=1	30 kDa	17
Proteasome assembly chaperone 1 OS=Mus musculus OX=10090 GN=Psmg1 PE=1 SV=1	33 kDa	6
Proteasome assembly chaperone 2 OS=Mus musculus OX=10090 GN=Psmg2 PE=1 SV=1	30 kDa	2
Proteasome assembly chaperone 3 OS=Mus musculus OX=10090 GN=Psmg3 PE=1 SV=1	13 kDa	2
Proteasome inhibitor PI31 subunit OS=Mus musculus OX=10090 GN=Psmf1 PE=1 SV=1	30 kDa	1
Proteasome subunit alpha type-1 OS=Mus musculus OX=10090 GN=Psma1 PE=1 SV=1	30 kDa	44
Proteasome subunit alpha type-2 OS=Mus musculus OX=10090 GN=Psma2 PE=1 SV=3	26 kDa	29
Proteasome subunit alpha type-3 OS=Mus musculus OX=10090 GN=Psma3 PE=1 SV=3	28 kDa	37
Proteasome subunit alpha type-4 OS=Mus musculus OX=10090 GN=Psma4 PE=1 SV=1	29 kDa	23
Proteasome subunit alpha type-5 OS=Mus musculus OX=10090 GN=Psma5 PE=1 SV=1	26 kDa	14
Proteasome subunit alpha type-6 OS=Mus musculus OX=10090 GN=Psma6 PE=1 SV=1	27 kDa	33
Proteasome subunit alpha type-7 OS=Mus musculus OX=10090 GN=Psma7 PE=1 SV=1	28 kDa	27
Proteasome subunit beta type-1 OS=Mus musculus OX=10090 GN=Psmb1 PE=1 SV=1	26 kDa	27
Proteasome subunit beta type-2 OS=Mus musculus OX=10090 GN=Psmb2 PE=1 SV=1	23 kDa	24
Proteasome subunit beta type-3 OS=Mus musculus OX=10090 GN=Psmb3 PE=1 SV=1	23 kDa	12
Proteasome subunit beta type-4 OS=Mus musculus OX=10090 GN=Psmb4 PE=1 SV=1	29 kDa	12
Proteasome subunit beta type-5 OS=Mus musculus OX=10090 GN=Psmb5 PE=1 SV=3	29 kDa	32
Proteasome subunit beta type-6 OS=Mus musculus OX=10090 GN=Psmb6 PE=1 SV=3	25 kDa	15
Proteasome subunit beta type-7 OS=Mus musculus OX=10090 GN=Psmb7 PE=1 SV=1	30 kDa	25

Protein AATF OS=Mus musculus OX=10090 GN=Aatf PE=1 SV=1	59 kDa	4
Protein ABHD14B OS=Mus musculus OX=10090 GN=Abhd14b PE=1 SV=1	22 kDa	4
Protein arginine N-methyltransferase 1 OS=Mus musculus OX=10090 GN=Prmt1 PE=1 SV=1	37 kDa	28
Protein arginine N-methyltransferase 3 OS=Mus musculus OX=10090 GN=Prmt3 PE=1 SV=2	60 kDa	1
Protein arginine N-methyltransferase 5 OS=Mus musculus OX=10090 GN=Prmt5 PE=1 SV=1	73 kDa	17
Protein arginine N-methyltransferase 7 OS=Mus musculus OX=10090 GN=Prmt7 PE=1 SV=1	78 kDa	2
Protein argonaute-2 OS=Mus musculus OX=10090 GN=Ago2 PE=1 SV=3	97 kDa	2
Protein argonaute-4 OS=Mus musculus OX=10090 GN=Ago4 PE=2 SV=2	97 kDa	1
Protein bicaudal D homolog 2 OS=Mus musculus OX=10090 GN=Bicd2 PE=1 SV=1	93 kDa	1
Protein BUD31 homolog OS=Mus musculus OX=10090 GN=Bud31 PE=1 SV=1	17 kDa	4
Protein canopy homolog 2 (Fragment) OS=Mus musculus OX=10090 GN=Cnpy2 PE=1 SV=1	15 kDa	13
Protein canopy homolog 3 OS=Mus musculus OX=10090 GN=Cnpy3 PE=1 SV=1	31 kDa	5
Protein canopy homolog 4 (Fragment) OS=Mus musculus OX=10090 GN=Cnpy4 PE=1 SV=1	18 kDa	2
Protein canopy homolog 4 OS=Mus musculus OX=10090 GN=Cnpy4 PE=1 SV=1	28 kDa	6
Protein CDV3 OS=Mus musculus OX=10090 GN=Cdv3 PE=1 SV=2	30 kDa	26
Protein DDI1 homolog 2 OS=Mus musculus OX=10090 GN=Ddi2 PE=1 SV=1	45 kDa	5
Protein DEK OS=Mus musculus OX=10090 GN=Dek PE=1 SV=1	43 kDa	16
Protein disulfide-isomerase A3 OS=Mus musculus OX=10090 GN=Pdia3 PE=1 SV=2	57 kDa	98
Protein disulfide-isomerase A4 OS=Mus musculus OX=10090 GN=Pdia4 PE=1 SV=1	72 kDa	66
Protein disulfide-isomerase A5 OS=Mus musculus OX=10090 GN=Pdia5 PE=1 SV=1	59 kDa	1
Protein disulfide-isomerase A6 OS=Mus musculus OX=10090 GN=Pdia6 PE=1 SV=1	49 kDa	57
Protein disulfide-isomerase OS=Mus musculus OX=10090 GN=P4hb PE=1 SV=2	57 kDa	161
Protein disulfide-isomerase TMX3 OS=Mus musculus OX=10090 GN=Tmx3 PE=1 SV=2	52 kDa	1
Protein Dr1 OS=Mus musculus OX=10090 GN=Dr1 PE=1 SV=1	19 kDa	3
Protein enabled homolog OS=Mus musculus OX=10090 GN=Enah PE=1 SV=1	84 kDa	6

Protein enabled homolog OS=Mus musculus OX=10090 GN=Enah PE=1 SV=2	62 kDa	6
Protein ERGIC-53 OS=Mus musculus OX=10090 GN=Lman1 PE=1 SV=1	58 kDa	4
Protein FAM107B OS=Mus musculus OX=10090 GN=Fam107b PE=1 SV=1	18 kDa	1
Protein FAM111A OS=Mus musculus OX=10090 GN=Fam111a PE=1 SV=1	65 kDa	1
Protein FAM114A2 OS=Mus musculus OX=10090 GN=Fam114a2 PE=1 SV=2	54 kDa	1
Protein FAM136A OS=Mus musculus OX=10090 GN=Fam136a PE=1 SV=1	16 kDa	2
Protein FAM162A OS=Mus musculus OX=10090 GN=Fam162a PE=1 SV=1	18 kDa	6
Protein FAM177A1 OS=Mus musculus OX=10090 GN=Fam177a1 PE=1 SV=1	24 kDa	1
Protein FAM185A OS=Mus musculus OX=10090 GN=Fam185a PE=2 SV=1	40 kDa	1
Protein FAM3C OS=Mus musculus OX=10090 GN=Fam3c PE=1 SV=1	25 kDa	1
Protein FAM49B OS=Mus musculus OX=10090 GN=Fam49b PE=1 SV=1	37 kDa	8
Protein FAM98B OS=Mus musculus OX=10090 GN=Fam98b PE=1 SV=1	45 kDa	1
Protein farnesyltransferase/geranylgeranyltransferase type-1 subunit alpha OS=Mus musculus OX=10090 GN=Fnta PE=1 SV=1	44 kDa	2
Protein flightless-1 homolog OS=Mus musculus OX=10090 GN=Flii PE=1 SV=1	145 kDa	5
Protein HEXIM1 OS=Mus musculus OX=10090 GN=Hexim1 PE=1 SV=1	40 kDa	3
Protein Hook homolog 3 OS=Mus musculus OX=10090 GN=Hook3 PE=1 SV=2	83 kDa	1
Protein incorporated later into tight junctions OS=Mus musculus OX=10090 GN=Tjap1 PE=1 SV=1	61 kDa	1
Protein IWS1 homolog OS=Mus musculus OX=10090 GN=Iws1 PE=1 SV=1	85 kDa	2
Protein kinase C and casein kinase substrate in neurons protein 2 OS=Mus musculus OX=10090 GN=Pacsin2 PE=1 SV=1	56 kDa	7
Protein kish OS=Mus musculus OX=10090 GN=Tmem167 PE=1 SV=1	6 kDa	1
Protein lin-7 homolog C OS=Mus musculus OX=10090 GN=Lin7c PE=1 SV=2	22 kDa	6
Protein LSM12 homolog OS=Mus musculus OX=10090 GN=Lsm12 PE=1 SV=1	22 kDa	4
Protein LYRIC OS=Mus musculus OX=10090 GN=Mtdh PE=1 SV=1	64 kDa	13
Protein LZIC OS=Mus musculus OX=10090 GN=Lzic PE=1 SV=1	22 kDa	2

Protein MAK16 homolog OS=Mus musculus OX=10090 GN=Mak16 PE=1 SV=1	35 kDa	1
Protein MANBAL OS=Mus musculus OX=10090 GN=Manbal PE=1 SV=1	9 kDa	1
Protein MEMO1 OS=Mus musculus OX=10090 GN=Memo1 PE=1 SV=1	34 kDa	7
Protein NDRG1 OS=Mus musculus OX=10090 GN=Ndrg1 PE=1 SV=1	43 kDa	1
Protein NDRG2 OS=Mus musculus OX=10090 GN=Ndrg2 PE=1 SV=1	41 kDa	3
Protein Niban OS=Mus musculus OX=10090 GN=Fam129a PE=1 SV=2	103 kDa	4
Protein O-GlcNAcase OS=Mus musculus OX=10090 GN=Mgea5 PE=1 SV=2	103 kDa	1
Protein O-glucosyltransferase 1 OS=Mus musculus OX=10090 GN=Poglut1 PE=1 SV=2	46 kDa	1
Protein OS-9 OS=Mus musculus OX=10090 GN=Os9 PE=1 SV=2	76 kDa	3
Protein PBDC1 OS=Mus musculus OX=10090 GN=Pbdc1 PE=1 SV=1	22 kDa	6
Protein pelota homolog OS=Mus musculus OX=10090 GN=Pelo PE=1 SV=3	43 kDa	1
Protein phosphatase 1 regulatory subunit 12A OS=Mus musculus OX=10090 GN=Ppp1r12a PE=1 SV=2	115 kDa	5
Protein phosphatase 1 regulatory subunit 14B OS=Mus musculus OX=10090 GN=Ppp1r14b PE=1 SV=2	16 kDa	5
Protein phosphatase 1 regulatory subunit 21 OS=Mus musculus OX=10090 GN=Ppp1r21 PE=1 SV=2	88 kDa	1
Protein phosphatase 1 regulatory subunit 7 OS=Mus musculus OX=10090 GN=Ppp1r7 PE=1 SV=2	41 kDa	13
Protein phosphatase 1A OS=Mus musculus OX=10090 GN=Ppm1a PE=1 SV=1	42 kDa	3
Protein phosphatase 1F OS=Mus musculus OX=10090 GN=Ppm1f PE=1 SV=1	50 kDa	1
Protein phosphatase 1G OS=Mus musculus OX=10090 GN=Ppm1g PE=1 SV=1	48 kDa	6
Protein phosphatase methylesterase 1 OS=Mus musculus OX=10090 GN=Ppme1 PE=1 SV=5	42 kDa	2
Protein PML OS=Mus musculus OX=10090 GN=Pml PE=1 SV=1	71 kDa	3
Protein PRRC1 OS=Mus musculus OX=10090 GN=Prrc1 PE=1 SV=1	46 kDa	3
Protein PRRC2A OS=Mus musculus OX=10090 GN=Prrc2a PE=1 SV=1	229 kDa	7
Protein PRRC2B OS=Mus musculus OX=10090 GN=Prrc2b PE=1 SV=1	161 kDa	2
Protein PRRC2C OS=Mus musculus OX=10090 GN=Prrc2c PE=1 SV=1	311 kDa	29
Protein prune homolog 2 OS=Mus musculus OX=10090 GN=Prune2 PE=1 SV=2	340 kDa	1

Protein quaking OS=Mus musculus OX=10090 GN=Qki PE=1 SV=1	38 kDa	2
Protein RCC2 OS=Mus musculus OX=10090 GN=Rcc2 PE=1 SV=1	56 kDa	18
Protein RER1 OS=Mus musculus OX=10090 GN=Rer1 PE=1 SV=1	23 kDa	4
Protein RRP5 homolog OS=Mus musculus OX=10090 GN=Pdcd11 PE=1 SV=2	208 kDa	1
Protein RTF2 homolog OS=Mus musculus OX=10090 GN=Rtfdc1 PE=1 SV=1	34 kDa	1
Protein S100-A10 OS=Mus musculus OX=10090 GN=S100a10 PE=1 SV=2	11 kDa	17
Protein S100-A11 OS=Mus musculus OX=10090 GN=S100a11 PE=1 SV=1	11 kDa	8
Protein S100-A13 OS=Mus musculus OX=10090 GN=S100a13 PE=1 SV=1	18 kDa	2
Protein S100-A4 (Fragment) OS=Mus musculus OX=10090 GN=S100a4 PE=1 SV=1	9 kDa	43
Protein S100-A6 OS=Mus musculus OX=10090 GN=S100a6 PE=1 SV=3	10 kDa	7
Protein scribble homolog OS=Mus musculus OX=10090 GN=Scrib PE=1 SV=2	174 kDa	1
Protein SEC13 homolog OS=Mus musculus OX=10090 GN=Sec13 PE=1 SV=3	36 kDa	5
Protein SET (Fragment) OS=Mus musculus OX=10090 GN=Set PE=1 SV=1	25 kDa	32
Protein SGT1 homolog OS=Mus musculus OX=10090 GN=Sugt1 PE=1 SV=3	38 kDa	10
Protein sprouty homolog 4 OS=Mus musculus OX=10090 GN=Spry4 PE=2 SV=1	33 kDa	1
Protein SREK1IP1 OS=Mus musculus OX=10090 GN=Srek1ip1 PE=1 SV=1	18 kDa	1
Protein transport protein Sec23A OS=Mus musculus OX=10090 GN=Sec23a PE=1 SV=1	83 kDa	4
Protein transport protein Sec23B OS=Mus musculus OX=10090 GN=Sec23b PE=1 SV=1	86 kDa	16
Protein transport protein Sec31A OS=Mus musculus OX=10090 GN=Sec31a PE=1 SV=2	134 kDa	12
Protein transport protein Sec61 subunit alpha isoform 2 OS=Mus musculus OX=10090 GN=Sec61a2 PE=1 SV=1	45 kDa	1
Protein transport protein Sec61 subunit beta OS=Mus musculus OX=10090 GN=Sec61b PE=1 SV=3	10 kDa	8
Protein transport protein Sec61 subunit gamma OS=Mus musculus OX=10090 GN=Sec61g PE=3 SV=1	8 kDa	1
Protein tyrosine phosphatase type IVA 2 OS=Mus musculus OX=10090 GN=Ptp4a2 PE=1 SV=1	19 kDa	1
Protein YIPF3 (Fragment) OS=Mus musculus OX=10090 GN=Yipf3 PE=1 SV=8	22 kDa	1
Protein/nucleic acid deglycase DJ-1 OS=Mus musculus OX=10090 GN=Park7 PE=1 SV=1	20 kDa	35

Protein-L-isoaspartate O-methyltransferase OS=Mus musculus OX=10090 GN=Pcmt1 PE=1 SV=1	30 kDa	1
Proteolipid protein 2 OS=Mus musculus OX=10090 GN=Plp2 PE=1 SV=1	17 kDa	3
Prothymosin alpha OS=Mus musculus OX=10090 GN=Ptma PE=1 SV=1	6 kDa	52
Prothymosin alpha OS=Mus musculus OX=10090 GN=Ptma PE=1 SV=2	12 kDa	95
Proto-oncogene c-Rel OS=Mus musculus OX=10090 GN=Rel PE=1 SV=1	65 kDa	1
Pseudopodium-enriched atypical kinase 1 OS=Mus musculus OX=10090 GN=Peak1 PE=1 SV=4	191 kDa	5
Pseudouridylate synthase 7 OS=Mus musculus OX=10090 GN=Pus7 PE=1 SV=1	75 kDa	7
Pumilio homolog 3 OS=Mus musculus OX=10090 GN=Pum3 PE=1 SV=1	73 kDa	1
Purine nucleoside phosphorylase OS=Mus musculus OX=10090 GN=Pnp PE=1 SV=1	32 kDa	17
Puromycin-sensitive aminopeptidase OS=Mus musculus OX=10090 GN=Npepps PE=1 SV=2	103 kDa	16
Putative 60S ribosomal protein L32' OS=Mus musculus OX=10090 GN=Rpl32-ps PE=5 SV=2	16 kDa	3
Putative ATP-dependent RNA helicase Pl10 OS=Mus musculus OX=10090 GN=D1Pas1 PE=1 SV=1	73 kDa	16
Putative D-tyrosyl-tRNA(Tyr) deacylase 2 OS=Mus musculus OX=10090 GN=Dtd2 PE=1 SV=1	18 kDa	1
Putative E3 ubiquitin-protein ligase UBR7 OS=Mus musculus OX=10090 GN=Ubr7 PE=1 SV=1	48 kDa	3
Putative hydrolase RBBP9 OS=Mus musculus OX=10090 GN=Rbbp9 PE=1 SV=2	21 kDa	3
Putative phospholipase B-like 2 OS=Mus musculus OX=10090 GN=Plbd2 PE=1 SV=2	66 kDa	3
Putative RNA-binding protein Luc7-like 1 OS=Mus musculus OX=10090 GN=Luc7l PE=1 SV=2	44 kDa	5
Putative RNA-binding protein Luc7-like 2 OS=Mus musculus OX=10090 GN=Luc7l2 PE=1 SV=1	47 kDa	11
Pygopus 2 OS=Mus musculus OX=10090 GN=Pygo2 PE=1 SV=1	38 kDa	1
Pyridoxal kinase OS=Mus musculus OX=10090 GN=Pdxk PE=1 SV=1	35 kDa	12
Pyridoxal phosphate homeostasis protein (Fragment) OS=Mus musculus OX=10090 GN=Plpbp PE=1 SV=1	36 kDa	2
Pyridoxal phosphate phosphatase OS=Mus musculus OX=10090 GN=Pdxp PE=1 SV=1	32 kDa	2
Pyrroline-5-carboxylate reductase 1, mitochondrial (Fragment) OS=Mus musculus OX=10090 GN=Pycr1 PE=1 SV=1	25 kDa	5
Pyrroline-5-carboxylate reductase 2 OS=Mus musculus OX=10090 GN=Pycr2 PE=1 SV=1	34 kDa	16

Pyrroline-5-carboxylate reductase 3 OS=Mus musculus OX=10090 GN=Pycr3 PE=1 SV=2	29 kDa	14
Pyruvate carboxylase OS=Mus musculus OX=10090 GN=Pcx PE=1 SV=1	130 kDa	1
Pyruvate dehydrogenase E1 component subunit alpha, somatic form, mitochondrial OS=Mus musculus OX=10090 GN=Pdha1 PE=1 SV=1	43 kDa	5
Pyruvate dehydrogenase E1 component subunit beta, mitochondrial OS=Mus musculus OX=10090 GN=Pdhb PE=1 SV=1	39 kDa	4
Pyruvate dehydrogenase protein X component, mitochondrial OS=Mus musculus OX=10090 GN=Pdhx PE=1 SV=1	54 kDa	4
Pyruvate kinase PKM OS=Mus musculus OX=10090 GN=Pkm PE=1 SV=4	58 kDa	342
Queuine tRNA-ribosyltransferase accessory subunit 2 OS=Mus musculus OX=10090 GN=Qtrt2 PE=1 SV=2	46 kDa	2
Queuine tRNA-ribosyltransferase catalytic subunit 1 OS=Mus musculus OX=10090 GN=Qtrt1 PE=1 SV=2	44 kDa	5
Quinone oxidoreductase OS=Mus musculus OX=10090 GN=Cryz PE=1 SV=1	35 kDa	1
Quinone oxidoreductase-like protein 1 OS=Mus musculus OX=10090 GN=Cryzl1 PE=1 SV=1	33 kDa	2
Rab GDP dissociation inhibitor (Fragment) OS=Mus musculus OX=10090 GN=Gdi2 PE=1 SV=1	36 kDa	18
Rab GDP dissociation inhibitor alpha OS=Mus musculus OX=10090 GN=Gdi1 PE=1 SV=3	51 kDa	20
Rab GDP dissociation inhibitor beta OS=Mus musculus OX=10090 GN=Gdi2 PE=1 SV=1	51 kDa	29
Rab GTPase-activating protein 1 OS=Mus musculus OX=10090 GN=Rabgap1 PE=1 SV=1	121 kDa	4
Rab GTPase-binding effector protein 1 OS=Mus musculus OX=10090 GN=Rabep1 PE=1 SV=1	90 kDa	1
Rab GTPase-binding effector protein 2 OS=Mus musculus OX=10090 GN=Rabep2 PE=1 SV=3	62 kDa	3
Rab proteins geranylgeranyltransferase component A 1 OS=Mus musculus OX=10090 GN=Chm PE=1 SV=1	74 kDa	1
RAB1A, member RAS oncogene family OS=Mus musculus OX=10090 GN=Rab1a PE=1 SV=1	22 kDa	28
Rab22B OS=Mus musculus OX=10090 GN=Rab31 PE=1 SV=1	21 kDa	4
Rab-like protein 6 OS=Mus musculus OX=10090 GN=Rabl6 PE=1 SV=2	80 kDa	1
RAC-alpha serine/threonine-protein kinase OS=Mus musculus OX=10090 GN=Akt1 PE=1 SV=2	56 kDa	1
RAD51-associated protein 1 OS=Mus musculus OX=10090 GN=Rad51ap1 PE=1 SV=1	36 kDa	1
Radixin OS=Mus musculus OX=10090 GN=Rdx PE=1 SV=3	69 kDa	59
Ragulator complex protein LAMTOR1 OS=Mus musculus OX=10090 GN=Lamtor1 PE=1 SV=1	16 kDa	3

Ragulator complex protein LAMTOR2 OS=Mus musculus OX=10090 GN=Lamtor2 PE=1 SV=1	13 kDa	1
Ragulator complex protein LAMTOR3 OS=Mus musculus OX=10090 GN=Lamtor3 PE=1 SV=1	14 kDa	2
RalBP1-associated Eps domain-containing protein 1 OS=Mus musculus OX=10090 GN=Reps1 PE=1 SV=1	77 kDa	5
Ran GTPase-activating protein 1 OS=Mus musculus OX=10090 GN=Rangap1 PE=1 SV=2	64 kDa	10
Ran-binding protein 3 OS=Mus musculus OX=10090 GN=Ranbp3 PE=1 SV=2	53 kDa	20
Ran-specific GTPase-activating protein OS=Mus musculus OX=10090 GN=Ranbp1 PE=1 SV=2	24 kDa	33
RAP1, GTP-GDP dissociation stimulator 1 OS=Mus musculus OX=10090 GN=Rap1gds1 PE=1 SV=1	61 kDa	3
Ras association (RalGDS/AF-6) and pleckstrin homology domains 1 OS=Mus musculus OX=10090 GN=Raph1 PE=1 SV=2	137 kDa	4
Ras GTPase-activating protein-binding protein 1 OS=Mus musculus OX=10090 GN=G3bp1 PE=1 SV=1	52 kDa	30
Ras GTPase-activating protein-binding protein 2 OS=Mus musculus OX=10090 GN=G3bp2 PE=1 SV=2	54 kDa	5
Ras GTPase-activating-like protein IQGAP1 OS=Mus musculus OX=10090 GN=Iqgap1 PE=1 SV=2	189 kDa	23
Ras GTPase-activating-like protein IQGAP2 OS=Mus musculus OX=10090 GN=Iqgap2 PE=1 SV=2	181 kDa	3
Ras suppressor protein 1 OS=Mus musculus OX=10090 GN=Rsu1 PE=1 SV=1	31 kDa	4
RAS-related C3 botulinum substrate 1, isoform CRA_a OS=Mus musculus OX=10090 GN=Rac1 PE=1 SV=1	23 kDa	29
Ras-related GTP-binding protein A OS=Mus musculus OX=10090 GN=Rraga PE=1 SV=1	37 kDa	3
Ras-related GTP-binding protein C OS=Mus musculus OX=10090 GN=Rragc PE=1 SV=1	44 kDa	7
Ras-related GTP-binding protein D OS=Mus musculus OX=10090 GN=Rragd PE=1 SV=1	32 kDa	2
Ras-related protein Rab-10 OS=Mus musculus OX=10090 GN=Rab10 PE=1 SV=1	23 kDa	20
Ras-related protein Rab-11B OS=Mus musculus OX=10090 GN=Rab11b PE=1 SV=3	24 kDa	14
Ras-related protein Rab-12 OS=Mus musculus OX=10090 GN=Rab12 PE=1 SV=1	32 kDa	7
Ras-related protein Rab-14 OS=Mus musculus OX=10090 GN=Rab14 PE=1 SV=3	24 kDa	22
Ras-related protein Rab-18 OS=Mus musculus OX=10090 GN=Rab18 PE=1 SV=2	23 kDa	5
Ras-related protein Rab-1B OS=Mus musculus OX=10090 GN=Rab1b PE=1 SV=1	22 kDa	18
Ras-related protein Rab-21 OS=Mus musculus OX=10090 GN=Rab21 PE=1 SV=4	24 kDa	4

Ras-related protein Rab-2A OS=Mus musculus OX=10090 GN=Rab2a PE=1 SV=1	24 kDa	15
Ras-related protein Rab-2B OS=Mus musculus OX=10090 GN=Rab2b PE=1 SV=1	21 kDa	7
Ras-related protein Rab-35 OS=Mus musculus OX=10090 GN=Rab35 PE=1 SV=1	23 kDa	11
Ras-related protein Rab-3A OS=Mus musculus OX=10090 GN=Rab3a PE=1 SV=1	25 kDa	6
Ras-related protein Rab-4A OS=Mus musculus OX=10090 GN=Rab4a PE=1 SV=2	24 kDa	6
Ras-related protein Rab-4B OS=Mus musculus OX=10090 GN=Rab4b PE=1 SV=2	24 kDa	7
Ras-related protein Rab-5A OS=Mus musculus OX=10090 GN=Rab5a PE=1 SV=1	24 kDa	5
Ras-related protein Rab-5B OS=Mus musculus OX=10090 GN=Rab5b PE=1 SV=1	24 kDa	9
Ras-related protein Rab-5C OS=Mus musculus OX=10090 GN=Rab5c PE=1 SV=1	25 kDa	14
Ras-related protein Rab-6A OS=Mus musculus OX=10090 GN=Rab6a PE=1 SV=4	24 kDa	19
Ras-related protein Rab-6B OS=Mus musculus OX=10090 GN=Rab6b PE=1 SV=1	23 kDa	15
Ras-related protein Rab-7a OS=Mus musculus OX=10090 GN=Rab7a PE=1 SV=2	23 kDa	24
Ras-related protein Rab-8A OS=Mus musculus OX=10090 GN=Rab8a PE=1 SV=2	24 kDa	16
Ras-related protein Rab-8B OS=Mus musculus OX=10090 GN=Rab8b PE=1 SV=1	24 kDa	15
Ras-related protein Ral-A OS=Mus musculus OX=10090 GN=Rala PE=1 SV=1	24 kDa	5
Ras-related protein Ral-B OS=Mus musculus OX=10090 GN=Ralb PE=1 SV=1	23 kDa	7
Ras-related protein Rap-1A OS=Mus musculus OX=10090 GN=Rap1a PE=1 SV=1	21 kDa	9
Ras-related protein Rap-1b OS=Mus musculus OX=10090 GN=Rap1b PE=1 SV=2	21 kDa	13
Ras-related protein Rap-2b OS=Mus musculus OX=10090 GN=Rap2b PE=1 SV=1	21 kDa	3
Ras-related protein R-Ras2 OS=Mus musculus OX=10090 GN=Rras2 PE=1 SV=1	23 kDa	1
Rcc1 protein OS=Mus musculus OX=10090 GN=Rcc1 PE=1 SV=1	46 kDa	2
Receptor expression-enhancing protein OS=Mus musculus OX=10090 GN=Reep5 PE=1 SV=1	21 kDa	11
Receptor of activated protein C kinase 1 OS=Mus musculus OX=10090 GN=Rack1 PE=1 SV=3	35 kDa	40
Recombining binding protein suppressor of hairless (Drosophila), isoform CRA_b OS=Mus musculus OX=10090 GN=Rbpj PE=1 SV=1	57 kDa	5

Regulation of nuclear pre-mRNA domain-containing protein 1B OS=Mus musculus OX=10090 GN=Rprd1b PE=1 SV=1	37 kDa	3
Regulator of G-protein signaling 10 OS=Mus musculus OX=10090 GN=Rgs10 PE=1 SV=1	21 kDa	1
Regulator of nonsense transcripts 1 OS=Mus musculus OX=10090 GN=Upf1 PE=1 SV=2	124 kDa	23
Remodeling and spacing factor 1 OS=Mus musculus OX=10090 GN=Rsf1 PE=1 SV=2	162 kDa	1
Replication factor C subunit 2 OS=Mus musculus OX=10090 GN=Rfc2 PE=1 SV=1	39 kDa	1
Replication factor C subunit 3 OS=Mus musculus OX=10090 GN=Rfc3 PE=1 SV=1	41 kDa	1
Replication factor C subunit 4 OS=Mus musculus OX=10090 GN=Rfc4 PE=1 SV=1	41 kDa	1
Replication protein A 14 kDa subunit OS=Mus musculus OX=10090 GN=Rpa3 PE=1 SV=1	14 kDa	1
Replication protein A 32 kDa subunit OS=Mus musculus OX=10090 GN=Rpa2 PE=1 SV=1	29 kDa	2
Replication protein A subunit OS=Mus musculus OX=10090 GN=Rpa1 PE=1 SV=2	71 kDa	8
REST corepressor 3 OS=Mus musculus OX=10090 GN=Rcor3 PE=1 SV=1	50 kDa	1
Reticulocalbin-1 OS=Mus musculus OX=10090 GN=Rcn1 PE=1 SV=1	38 kDa	6
Reticulon-3 OS=Mus musculus OX=10090 GN=Rtn3 PE=1 SV=2	104 kDa	5
Reticulon-4 OS=Mus musculus OX=10090 GN=Rtn4 PE=1 SV=2	127 kDa	45
Retinal dehydrogenase 2 OS=Mus musculus OX=10090 GN=Aldh1a2 PE=1 SV=2	57 kDa	1
Retinal rod rhodopsin-sensitive cGMP 3',5'-cyclic phosphodiesterase subunit delta OS=Mus musculus OX=10090 GN=Pde6d PE=1 SV=1	14 kDa	2
Retinoblastoma-binding protein 5 (Fragment) OS=Mus musculus OX=10090 GN=Rbbp5 PE=1 SV=1	17 kDa	2
Retinoblastoma-binding protein 5 OS=Mus musculus OX=10090 GN=Rbbp5 PE=1 SV=1	59 kDa	3
Retinoic acid early-inducible protein 1-alpha OS=Mus musculus OX=10090 GN=Raet1a PE=1 SV=1	29 kDa	2
Retinoid-inducible serine carboxypeptidase OS=Mus musculus OX=10090 GN=Scpep1 PE=1 SV=2	51 kDa	4
Rho GDP-dissociation inhibitor 1 OS=Mus musculus OX=10090 GN=Arhgdia PE=1 SV=3	23 kDa	65
Rho GTPase-activating protein 1 OS=Mus musculus OX=10090 GN=Arhgap1 PE=1 SV=1	54 kDa	6
Rho guanine nucleotide exchange factor 2 OS=Mus musculus OX=10090 GN=Arhgef2 PE=1 SV=1	109 kDa	2
Rho-associated protein kinase 2 (Fragment) OS=Mus musculus OX=10090 GN=Rock2 PE=1 SV=1	167 kDa	3

Rho-related GTP-binding protein RhoB OS=Mus musculus OX=10090 GN=Rhob PE=1 SV=1	22 kDa	3
Rho-related GTP-binding protein RhoC OS=Mus musculus OX=10090 GN=Rhoc PE=1 SV=1	17 kDa	15
Rho-related GTP-binding protein RhoC OS=Mus musculus OX=10090 GN=Rhoc PE=1 SV=2	22 kDa	18
Rho-related GTP-binding protein RhoG OS=Mus musculus OX=10090 GN=Rhog PE=1 SV=1	21 kDa	6
Ribonuclease H2 subunit B OS=Mus musculus OX=10090 GN=Rnaseh2b PE=1 SV=2	35 kDa	1
Ribonuclease inhibitor OS=Mus musculus OX=10090 GN=Rnh1 PE=1 SV=1	50 kDa	19
Ribonuclease T2-A OS=Mus musculus OX=10090 GN=Rnaset2a PE=1 SV=1	30 kDa	1
Ribonucleoside-diphosphate reductase large subunit OS=Mus musculus OX=10090 GN=Rrm1 PE=1 SV=2	90 kDa	3
Ribonucleoside-diphosphate reductase subunit M2 OS=Mus musculus OX=10090 GN=Rrm2 PE=1 SV=1	45 kDa	6
Ribose-5-phosphate isomerase OS=Mus musculus OX=10090 GN=Rpia PE=1 SV=2	32 kDa	3
Ribosomal L1 domain-containing protein 1 OS=Mus musculus OX=10090 GN=Rsl1d1 PE=1 SV=1	50 kDa	15
Ribosomal protein L19 OS=Mus musculus OX=10090 GN=Rpl19 PE=1 SV=1	23 kDa	14
Ribosomal protein OS=Mus musculus OX=10090 GN=Rpl10a PE=1 SV=1	25 kDa	29
Ribosomal RNA processing protein 1 homolog A OS=Mus musculus OX=10090 GN=Rrp1 PE=1 SV=2	55 kDa	7
Ribosomal RNA processing protein 1 homolog B OS=Mus musculus OX=10090 GN=Rrp1b PE=1 SV=2	81 kDa	1
Ribosome assembly factor mrt4 OS=Mus musculus OX=10090 GN=Mrto4 PE=1 SV=1	27 kDa	5
Ribosome biogenesis protein BOP1 OS=Mus musculus OX=10090 GN=Bop1 PE=1 SV=1	83 kDa	2
Ribosome biogenesis protein WDR12 OS=Mus musculus OX=10090 GN=Wdr12 PE=1 SV=1	47 kDa	8
Ribosome-binding protein 1 OS=Mus musculus OX=10090 GN=Rrbp1 PE=1 SV=1	158 kDa	43
Ribosyldihydronicotinamide dehydrogenase [quinone] OS=Homo sapiens GN=NQO2 PE=1 SV=5	26 kDa	1
Ribosyldihydronicotinamide dehydrogenase [quinone] OS=Mus musculus OX=10090 GN=Nqo2 PE=1 SV=3	26 kDa	5
RIKEN cDNA 0610037L13 gene OS=Mus musculus OX=10090 GN=0610037L13Rik PE=1 SV=1	16 kDa	4
RIKEN cDNA 4930430F08 gene (Fragment) OS=Mus musculus OX=10090 GN=4930430F08Rik PE=1 SV=1	25 kDa	1
RIKEN cDNA 4931406C07 gene OS=Mus musculus OX=10090 GN=4931406C07Rik PE=1 SV=1	31 kDa	5
RIKEN cDNA 5830416A07, isoform CRA_c OS=Mus musculus OX=10090 GN=Zc3h18 PE=1 SV=1	108 kDa	7

RING finger and CHY zinc finger domain-containing protein 1 OS=Mus musculus OX=10090 GN=Rchy1 PE=1 SV=1	30 kDa	1
RING finger protein 214 OS=Mus musculus OX=10090 GN=Rnf214 PE=1 SV=1	74 kDa	1
RING1 and YY1-binding protein OS=Mus musculus OX=10090 GN=Rybp PE=1 SV=1	25 kDa	2
RING-type E3 ubiquitin transferase OS=Mus musculus OX=10090 GN=Arih1 PE=1 SV=1	42 kDa	5
RNA binding protein fox-1 homolog 2 OS=Mus musculus OX=10090 GN=Rbfox2 PE=1 SV=2	47 kDa	1
RNA cytidine acetyltransferase OS=Mus musculus OX=10090 GN=Nat10 PE=1 SV=1	115 kDa	1
RNA polymerase II subunit A C-terminal domain phosphatase OS=Mus musculus OX=10090 GN=Ctdp1 PE=1 SV=1	105 kDa	7
RNA polymerase II subunit A C-terminal domain phosphatase SSU72 OS=Mus musculus OX=10090 GN=Ssu72 PE=1 SV=1	23 kDa	2
RNA polymerase-associated protein LEO1 OS=Mus musculus OX=10090 GN=Leo1 PE=1 SV=2	76 kDa	1
RNA transcription, translation and transport factor protein OS=Mus musculus OX=10090 GN=RTRAF PE=1 SV=1	28 kDa	3
RNA-binding motif protein 15 OS=Mus musculus OX=10090 GN=Rbm15 PE=1 SV=1	106 kDa	1
RNA-binding motif, single-stranded-interacting protein 1 OS=Mus musculus OX=10090 GN=Rbms1 PE=1 SV=1	45 kDa	1
RNA-binding motif, single-stranded-interacting protein 2 OS=Mus musculus OX=10090 GN=Rbms2 PE=1 SV=1	38 kDa	1
RNA-binding protein 10 OS=Mus musculus OX=10090 GN=Rbm10 PE=1 SV=1	103 kDa	1
RNA-binding protein 14 OS=Mus musculus OX=10090 GN=Rbm14 PE=1 SV=1	69 kDa	3
RNA-binding protein 25 OS=Mus musculus OX=10090 GN=Rbm25 PE=1 SV=2	100 kDa	2
RNA-binding protein 28 (Fragment) OS=Mus musculus OX=10090 GN=Rbm28 PE=1 SV=1	15 kDa	1
RNA-binding protein 28 OS=Mus musculus OX=10090 GN=Rbm28 PE=1 SV=4	84 kDa	1
RNA-binding protein 3 OS=Mus musculus OX=10090 GN=Rbm3 PE=1 SV=1	17 kDa	10
RNA-binding protein 33 OS=Mus musculus OX=10090 GN=Rbm33 PE=1 SV=1	133 kDa	1
RNA-binding protein 34 OS=Mus musculus OX=10090 GN=Rbm34 PE=1 SV=1	47 kDa	1
RNA-binding protein 39 OS=Mus musculus OX=10090 GN=Rbm39 PE=1 SV=2	59 kDa	7
RNA-binding protein 8A OS=Mus musculus OX=10090 GN=Rbm8a PE=1 SV=4	20 kDa	6
RNA-binding protein EWS OS=Mus musculus OX=10090 GN=Ewsr1 PE=1 SV=2	68 kDa	15

RNA-binding protein FUS OS=Mus musculus OX=10090 GN=Fus PE=1 SV=1	53 kDa	24
RNA-binding protein NOB1 OS=Mus musculus OX=10090 GN=Nob1 PE=1 SV=1	45 kDa	1
RNA-binding protein PNO1 OS=Mus musculus OX=10090 GN=Pno1 PE=1 SV=1	27 kDa	1
RNA-binding protein Raly (Fragment) OS=Mus musculus OX=10090 GN=Raly PE=1 SV=1	32 kDa	5
RNA-binding protein with multiple splicing OS=Mus musculus OX=10090 GN=Rbpms PE=1 SV=2	22 kDa	1
RNA-binding protein with serine-rich domain 1 OS=Mus musculus OX=10090 GN=Rnps1 PE=1 SV=1	34 kDa	4
RNA-binding Raly-like protein (Fragment) OS=Mus musculus OX=10090 GN=Ralyl PE=1 SV=1	18 kDa	1
RNMT-activating mini protein OS=Mus musculus OX=10090 GN=Fam103a1 PE=3 SV=1	15 kDa	1
rRNA 2'-O-methyltransferase fibrillarin OS=Mus musculus OX=10090 GN=Fbl PE=1 SV=2	34 kDa	5
rRNA methyltransferase 3, mitochondrial OS=Mus musculus OX=10090 GN=Mrm3 PE=2 SV=1	47 kDa	1
rRNA/tRNA 2'-O-methyltransferase fibrillarin-like protein 1 OS=Mus musculus OX=10090 GN=Fbll1 PE=1 SV=1	33 kDa	3
RRP12-like protein OS=Mus musculus OX=10090 GN=Rrp12 PE=1 SV=1	143 kDa	1
RRP15-like protein OS=Mus musculus OX=10090 GN=Rrp15 PE=1 SV=2	31 kDa	2
RUN and FYVE domain-containing protein 1 OS=Mus musculus OX=10090 GN=Rufy1 PE=1 SV=1	80 kDa	1
RuvB-like 1 OS=Mus musculus OX=10090 GN=Ruvbl1 PE=1 SV=1	50 kDa	38
RuvB-like 2 OS=Mus musculus OX=10090 GN=Ruvbl2 PE=1 SV=3	51 kDa	56
S-adenosylhomocysteine hydrolase-like protein 1 OS=Mus musculus OX=10090 GN=Ahcyl1 PE=1 SV=1	59 kDa	2
S-adenosylmethionine synthase OS=Mus musculus OX=10090 GN=Mat2a PE=1 SV=1	40 kDa	8
SAFB-like transcription modulator OS=Mus musculus OX=10090 GN=Sltm PE=1 SV=1	117 kDa	1
SAP domain-containing ribonucleoprotein OS=Mus musculus OX=10090 GN=Sarnp PE=1 SV=3	24 kDa	15
SAP30-binding protein OS=Mus musculus OX=10090 GN=Sap30bp PE=1 SV=2	34 kDa	1
SAPS domain family, member 3, isoform CRA_c OS=Mus musculus OX=10090 GN=Ppp6r3 PE=1 SV=1	98 kDa	2
Scaffold attachment factor B1 OS=Mus musculus OX=10090 GN=Safb PE=1 SV=1	105 kDa	8
Scavenger receptor class F member 2 OS=Mus musculus OX=10090 GN=Scarf2 PE=1 SV=1	88 kDa	1
SEC23-interacting protein OS=Mus musculus OX=10090 GN=Sec23ip PE=1 SV=2	111 kDa	4

Sec24-related gene family, member B (S. cerevisiae) OS=Mus musculus OX=10090 GN=Sec24b PE=1 SV=1	136 kDa	1
Sec24-related gene family, member D (S. cerevisiae) OS=Mus musculus OX=10090 GN=Sec24d PE=1 SV=1	113 kDa	2
Secretory carrier-associated membrane protein (Fragment) OS=Mus musculus OX=10090 GN=Scamp2 PE=1 SV=1	22 kDa	2
Secretory carrier-associated membrane protein OS=Mus musculus OX=10090 GN=Scamp1 PE=1 SV=1	32 kDa	2
Segment polarity protein dishevelled homolog DVL-2 OS=Mus musculus OX=10090 GN=Dvl2 PE=1 SV=2	79 kDa	2
Selenide, water dikinase 1 OS=Mus musculus OX=10090 GN=Sephs1 PE=1 SV=1	43 kDa	6
Selenium-binding protein 1 OS=Mus musculus OX=10090 GN=Selenbp1 PE=1 SV=2	53 kDa	3
Sentrin-specific protease 3 OS=Mus musculus OX=10090 GN=Senp3 PE=1 SV=1	64 kDa	1
Sepiapterin reductase OS=Mus musculus OX=10090 GN=Spr PE=1 SV=1	23 kDa	3
Septin-10 OS=Mus musculus OX=10090 GN=Sept10 PE=1 SV=1	50 kDa	10
Septin-11 OS=Mus musculus OX=10090 GN=Sept11 PE=1 SV=1	49 kDa	38
Septin-2 OS=Mus musculus OX=10090 GN=Sept2 PE=1 SV=2	42 kDa	40
Septin-6 OS=Mus musculus OX=10090 GN=Sept6 PE=1 SV=4	50 kDa	8
Septin-7 OS=Mus musculus OX=10090 GN=Sept7 PE=1 SV=2	51 kDa	40
Septin-7 OS=Mus musculus OX=10090 GN=Sept7 PE=1 SV=2	51 kDa	39
Septin-8 OS=Mus musculus OX=10090 GN=Sept8 PE=1 SV=1	51 kDa	17
Septin-9 OS=Mus musculus OX=10090 GN=Sept9 PE=1 SV=1	64 kDa	65
Sequestosome-1 OS=Mus musculus OX=10090 GN=Sqstm1 PE=1 SV=1	42 kDa	10
Serine hydroxymethyltransferase OS=Mus musculus OX=10090 GN=Shmt2 PE=1 SV=1	55 kDa	110
Serine hydroxymethyltransferase, cytosolic OS=Mus musculus OX=10090 GN=Shmt1 PE=1 SV=3	53 kDa	12
Serine protease HTRA2, mitochondrial OS=Mus musculus OX=10090 GN=Htra2 PE=1 SV=1	46 kDa	4
Serine/arginine repetitive matrix protein 1 OS=Mus musculus OX=10090 GN=Srrm1 PE=1 SV=1	103 kDa	9
Serine/arginine repetitive matrix protein 2 (Fragment) OS=Mus musculus OX=10090 GN=Srrm2 PE=1 SV=1	20 kDa	2
Serine/arginine repetitive matrix protein 2 OS=Mus musculus OX=10090 GN=Srrm2 PE=1 SV=3	295 kDa	41
Serine/arginine-rich splicing factor 2 OS=Mus musculus OX=10090 GN=Srsf2 PE=1 SV=4	25 kDa	17

Serine/arginine-rich splicing factor 3 OS=Mus musculus OX=10090 GN=Srsf3 PE=1 SV=1	19 kDa	15
Serine/arginine-rich splicing factor 6 OS=Mus musculus OX=10090 GN=Srsf6 PE=1 SV=1	39 kDa	7
Serine/arginine-rich splicing factor 7 OS=Mus musculus OX=10090 GN=Srsf7 PE=1 SV=1	31 kDa	7
Serine/arginine-rich-splicing factor 1 OS=Mus musculus OX=10090 GN=Srsf1 PE=1 SV=1	28 kDa	23
Serine/arginine-rich-splicing factor 10 OS=Mus musculus OX=10090 GN=Srsf10 PE=1 SV=1	22 kDa	2
Serine/arginine-rich-splicing factor 11 OS=Mus musculus OX=10090 GN=Srsf11 PE=1 SV=1	57 kDa	2
Serine/arginine-rich-splicing factor 12 (Fragment) OS=Mus musculus OX=10090 GN=Srsf12 PE=1 SV=1	30 kDa	1
Serine/arginine-rich-splicing factor 4 OS=Mus musculus OX=10090 GN=Srsf4 PE=1 SV=1	56 kDa	3
Serine/threonine-protein kinase 24 OS=Mus musculus OX=10090 GN=Stk24 PE=1 SV=1	48 kDa	1
Serine/threonine-protein kinase 3 OS=Mus musculus OX=10090 GN=Stk3 PE=4 SV=1	57 kDa	2
Serine/threonine-protein kinase ICK OS=Mus musculus OX=10090 GN=Ick PE=1 SV=2	71 kDa	2
Serine/threonine-protein kinase MAK OS=Mus musculus OX=10090 GN=Mak PE=4 SV=1	70 kDa	2
Serine/threonine-protein kinase OSR1 OS=Mus musculus OX=10090 GN=Oxsr1 PE=1 SV=1	58 kDa	1
Serine/threonine-protein kinase PAK 2 OS=Mus musculus OX=10090 GN=Pak2 PE=1 SV=1	58 kDa	23
Serine/threonine-protein kinase SIK3 OS=Mus musculus OX=10090 GN=Sik3 PE=1 SV=1	151 kDa	1
Serine/threonine-protein kinase WNK1 OS=Mus musculus OX=10090 GN=Wnk1 PE=1 SV=2	251 kDa	9
Serine/threonine-protein kinase WNK2 OS=Mus musculus OX=10090 GN=Wnk2 PE=1 SV=1	226 kDa	1
Serine/threonine-protein kinase WNK4 OS=Mus musculus OX=10090 GN=Wnk4 PE=1 SV=1	132 kDa	1
Serine/threonine-protein phosphatase 1 regulatory subunit 10 OS=Mus musculus OX=10090 GN=Ppp1r10 PE=1 SV=1	94 kDa	1
Serine/threonine-protein phosphatase 2A 55 kDa regulatory subunit B alpha isoform OS=Mus musculus OX=10090 GN=Ppp2r2a PE=1 SV=1	52 kDa	10
Serine/threonine-protein phosphatase 2A 56 kDa regulatory subunit alpha isoform OS=Mus musculus OX=10090 GN=Ppp2r5a PE=1 SV=1	56 kDa	1
Serine/threonine-protein phosphatase 2A 56 kDa regulatory subunit OS=Mus musculus OX=10090 GN=Ppp2r5d PE=1 SV=1	69 kDa	1
Serine/threonine-protein phosphatase 2A 65 kDa regulatory subunit A alpha isoform OS=Mus musculus OX=10090 GN=Ppp2r1a PE=1 SV=3	65 kDa	13

Serine/threonine-protein phosphatase 2A 65 kDa regulatory subunit A beta isoform OS=Mus musculus OX=10090 GN=Ppp2r1b PE=1 SV=1	76 kDa	3
Serine/threonine-protein phosphatase 2A activator OS=Mus musculus OX=10090 GN=Ptpa PE=1 SV=1	37 kDa	4
Serine/threonine-protein phosphatase 2A catalytic subunit alpha isoform OS=Mus musculus OX=10090 GN=Ppp2ca PE=1 SV=1	36 kDa	13
Serine/threonine-protein phosphatase 2A catalytic subunit beta isoform OS=Mus musculus OX=10090 GN=Ppp2cb PE=1 SV=1	36 kDa	12
Serine/threonine-protein phosphatase 2B catalytic subunit alpha isoform OS=Mus musculus OX=10090 GN=Ppp3ca PE=1 SV=1	59 kDa	14
Serine/threonine-protein phosphatase 2B catalytic subunit gamma isoform OS=Mus musculus OX=10090 GN=Ppp3cc PE=4 SV=1	60 kDa	4
Serine/threonine-protein phosphatase 4 catalytic subunit OS=Mus musculus OX=10090 GN=Ppp4c PE=1 SV=2	35 kDa	3
Serine/threonine-protein phosphatase 4 regulatory subunit 1 OS=Mus musculus OX=10090 GN=Ppp4r1 PE=1 SV=1	105 kDa	3
Serine/threonine-protein phosphatase 4 regulatory subunit 2 OS=Mus musculus OX=10090 GN=Ppp4r2 PE=1 SV=1	46 kDa	11
Serine/threonine-protein phosphatase 4 regulatory subunit 3A OS=Mus musculus OX=10090 GN=Ppp4r3a PE=1 SV=1	95 kDa	2
Serine/threonine-protein phosphatase 5 OS=Mus musculus OX=10090 GN=Ppp5c PE=1 SV=3	57 kDa	7
Serine/threonine-protein phosphatase 6 regulatory subunit 1 OS=Mus musculus OX=10090 GN=Ppp6r1 PE=1 SV=1	95 kDa	1
Serine/threonine-protein phosphatase CPPED1 OS=Mus musculus OX=10090 GN=Cpped1 PE=1 SV=1	35 kDa	7
Serine/threonine-protein phosphatase OS=Mus musculus OX=10090 GN=Ppp3cb PE=1 SV=1	59 kDa	6
Serine/threonine-protein phosphatase OS=Mus musculus OX=10090 GN=Ppp6c PE=1 SV=1	33 kDa	6
Serine/threonine-protein phosphatase PP1-alpha catalytic subunit OS=Mus musculus OX=10090 GN=Ppp1ca PE=1 SV=1	38 kDa	32
Serine/threonine-protein phosphatase PP1-beta catalytic subunit OS=Mus musculus OX=10090 GN=Ppp1cb PE=1 SV=3	37 kDa	23
Serine/threonine-protein phosphatase PP1-gamma catalytic subunit OS=Mus musculus OX=10090 GN=Ppp1cc PE=1 SV=1	37 kDa	25
Serine-threonine kinase receptor-associated protein OS=Mus musculus OX=10090 GN=Strap PE=1 SV=2	38 kDa	32
Serine--tRNA ligase, cytoplasmic OS=Mus musculus OX=10090 GN=Sars PE=1 SV=1	61 kDa	28
Serine--tRNA ligase, mitochondrial OS=Mus musculus OX=10090 GN=Sars2 PE=1 SV=2	58 kDa	1

Serpin B6 OS=Mus musculus OX=10090 GN=Serpinb6 PE=1 SV=1	43 kDa	33
Serpin H1 OS=Mus musculus OX=10090 GN=Serpinh1 PE=1 SV=3	47 kDa	29
Serrate RNA effector molecule homolog OS=Mus musculus OX=10090 GN=Srrt PE=1 SV=1	100 kDa	7
Serum albumin OS=Bos taurus GN=ALB PE=1 SV=4	69 kDa	20
Serum albumin OS=Homo sapiens GN=ALB PE=1 SV=2	69 kDa	2
Sestrin-2 OS=Mus musculus OX=10090 GN=Sesn2 PE=1 SV=1	54 kDa	1
SET and MYND domain-containing protein 5 OS=Mus musculus OX=10090 GN=Smyd5 PE=1 SV=2	47 kDa	1
S-formylglutathione hydrolase OS=Mus musculus OX=10090 GN=Esd PE=1 SV=1	31 kDa	31
SH3 and PX domain-containing protein 2B OS=Mus musculus OX=10090 GN=Sh3pxd2b PE=1 SV=1	102 kDa	2
SH3 domain-binding glutamic acid-rich-like protein 2 OS=Mus musculus OX=10090 GN=Sh3bgrl2 PE=1 SV=1	12 kDa	1
SH3 domain-binding glutamic acid-rich-like protein 3 OS=Mus musculus OX=10090 GN=Sh3bgrl3 PE=1 SV=1	10 kDa	6
SH3 domain-binding glutamic acid-rich-like protein OS=Mus musculus OX=10090 GN=Sh3bgrl PE=1 SV=1	13 kDa	6
SH3 domain-containing kinase-binding protein 1 OS=Mus musculus OX=10090 GN=Sh3kbp1 PE=1 SV=1	78 kDa	24
SH3 domain-containing kinase-binding protein 1 OS=Mus musculus OX=10090 GN=Sh3kbp1 PE=1 SV=1	49 kDa	15
SHC-transforming protein 1 OS=Mus musculus OX=10090 GN=Shc1 PE=1 SV=3	63 kDa	2
Short-chain specific acyl-CoA dehydrogenase, mitochondrial OS=Mus musculus OX=10090 GN=Acads PE=1 SV=2	45 kDa	4
Sialic acid synthase OS=Mus musculus OX=10090 GN=Nans PE=1 SV=1	40 kDa	14
Sideroflexin-1 OS=Mus musculus OX=10090 GN=Sfxn1 PE=1 SV=3	36 kDa	4
Sideroflexin-3 OS=Mus musculus OX=10090 GN=Sfxn3 PE=1 SV=1	31 kDa	1
Sigma non-opioid intracellular receptor 1 OS=Mus musculus OX=10090 GN=Sigmar1 PE=1 SV=1	25 kDa	1
Signal peptidase complex subunit 1 OS=Mus musculus OX=10090 GN=Spcs1 PE=4 SV=1	12 kDa	1
Signal peptidase complex subunit 2 OS=Mus musculus OX=10090 GN=Spcs2 PE=1 SV=1	28 kDa	1
Signal peptidase complex subunit 3 OS=Mus musculus OX=10090 GN=Spcs3 PE=1 SV=1	20 kDa	1
Signal peptide peptidase-like 2A OS=Mus musculus OX=10090 GN=Sppl2a PE=1 SV=2	58 kDa	1
Signal recognition particle 14 kDa protein OS=Mus musculus OX=10090 GN=Srp14 PE=1 SV=1	13 kDa	6

Signal recognition particle 19 kDa protein OS=Mus musculus OX=10090 GN=Srp19 PE=1 SV=1	14 kDa	2
Signal recognition particle 9 kDa protein OS=Mus musculus OX=10090 GN=Srp9 PE=1 SV=2	10 kDa	4
Signal recognition particle subunit SRP68 OS=Mus musculus OX=10090 GN=Srp68 PE=1 SV=2	71 kDa	3
Signal recognition particle subunit SRP72 OS=Mus musculus OX=10090 GN=Srp72 PE=1 SV=1	68 kDa	3
Signal sequence receptor, delta OS=Mus musculus OX=10090 GN=Ssr4 PE=1 SV=1	19 kDa	2
Signal-induced proliferation-associated protein 1 OS=Mus musculus OX=10090 GN=Sipa1 PE=1 SV=2	112 kDa	1
Single-stranded DNA binding protein 1 OS=Mus musculus OX=10090 GN=Ssbp1 PE=1 SV=1	17 kDa	12
Single-stranded DNA-binding protein 3 (Fragment) OS=Mus musculus OX=10090 GN=Ssbp3 PE=1 SV=8	24 kDa	1
Sister chromatid cohesion protein PDS5 homolog A OS=Mus musculus OX=10090 GN=Pds5a PE=1 SV=1	150 kDa	1
Sister chromatid cohesion protein PDS5 homolog B OS=Mus musculus OX=10090 GN=Pds5b PE=1 SV=1	165 kDa	1
Sjoegren syndrome/scleroderma autoantigen 1 homolog OS=Mus musculus OX=10090 GN=Sssca1 PE=1 SV=1	21 kDa	1
SLAIN motif-containing protein 2 OS=Mus musculus OX=10090 GN=Slain2 PE=1 SV=2	62 kDa	2
Small acidic protein OS=Mus musculus OX=10090 GN=Smap PE=1 SV=1	20 kDa	7
Small EDRK-rich factor 2 OS=Mus musculus OX=10090 GN=Serf2 PE=1 SV=1	7 kDa	2
Small glutamine-rich tetratricopeptide repeat-containing protein alpha (Fragment) OS=Mus musculus OX=10090 GN=Sgta PE=1 SV=1	23 kDa	16
Small glutamine-rich tetratricopeptide repeat-containing protein alpha OS=Mus musculus OX=10090 GN=Sgta PE=1 SV=2	34 kDa	18
Small nuclear ribonucleoprotein E OS=Mus musculus OX=10090 GN=Snrpe PE=1 SV=1	11 kDa	8
Small nuclear ribonucleoprotein E OS=Mus musculus OX=10090 GN=Snrpe PE=1 SV=1	6 kDa	4
Small nuclear ribonucleoprotein F OS=Mus musculus OX=10090 GN=Snrpf PE=1 SV=1	10 kDa	8
Small nuclear ribonucleoprotein G OS=Mus musculus OX=10090 GN=Snrpg PE=1 SV=1	8 kDa	2
Small nuclear ribonucleoprotein Sm D1 OS=Mus musculus OX=10090 GN=Snrpd1 PE=1 SV=1	13 kDa	10
Small nuclear ribonucleoprotein Sm D2 OS=Mus musculus OX=10090 GN=Snrpd2 PE=1 SV=1	14 kDa	17
Small nuclear ribonucleoprotein Sm D3 OS=Mus musculus OX=10090 GN=Snrpd3 PE=1 SV=1	14 kDa	14
Small nuclear ribonucleoprotein-associated protein B OS=Mus musculus OX=10090 GN=Snrpb PE=1 SV=1	24 kDa	11

Small ubiquitin-related modifier 1 OS=Homo sapiens GN=SUMO1 PE=1 SV=1	12 kDa	2
Small ubiquitin-related modifier 2 OS=Mus musculus OX=10090 GN=Sumo2 PE=1 SV=1	8 kDa	14
S-methyl-5'-thioadenosine phosphorylase OS=Mus musculus OX=10090 GN=Mtap PE=1 SV=1	31 kDa	27
SMT3 suppressor of mif two 3 homolog 3 (Yeast), isoform CRA_b OS=Mus musculus OX=10090 GN=Sumo3 PE=1 SV=1	11 kDa	7
SNARE-associated protein Snapin OS=Mus musculus OX=10090 GN=Snapin PE=1 SV=1	15 kDa	2
SNW domain-containing protein 1 OS=Mus musculus OX=10090 GN=Snw1 PE=1 SV=3	61 kDa	5
Sodium/potassium-transporting ATPase subunit alpha OS=Mus musculus OX=10090 GN=Atp1a3 PE=1 SV=1	113 kDa	3
Sodium/potassium-transporting ATPase subunit alpha-1 OS=Mus musculus OX=10090 GN=Atp1a1 PE=1 SV=1	113 kDa	9
Sodium/potassium-transporting ATPase subunit alpha-2 OS=Mus musculus OX=10090 GN=Atp1a2 PE=1 SV=1	112 kDa	3
Sodium/potassium-transporting ATPase subunit beta-3 OS=Mus musculus OX=10090 GN=Atp1b3 PE=1 SV=1	32 kDa	1
Solute carrier family 12 member 2 OS=Mus musculus OX=10090 GN=Slc12a2 PE=1 SV=1	131 kDa	1
Solute carrier family 4 (anion exchanger), member 1, adaptor protein OS=Mus musculus OX=10090 GN=Slc4a1ap PE=1 SV=1	83 kDa	3
Something about silencing protein 10 OS=Mus musculus OX=10090 GN=Utp3 PE=1 SV=1	53 kDa	1
Sorbin and SH3 domain-containing protein 1 OS=Mus musculus OX=10090 GN=Sorbs1 PE=1 SV=1	143 kDa	1
Sorbs3 protein OS=Mus musculus OX=10090 GN=Sorbs3 PE=2 SV=1	77 kDa	1
Sorcin OS=Mus musculus OX=10090 GN=Sri PE=1 SV=1	22 kDa	5
Sorting nexin 1 OS=Mus musculus OX=10090 GN=Snx1 PE=1 SV=1	59 kDa	9
Sorting nexin 12, isoform CRA_b OS=Mus musculus OX=10090 GN=Snx12 PE=1 SV=1	19 kDa	4
Sorting nexin 3, isoform CRA_b OS=Mus musculus OX=10090 GN=Snx3 PE=1 SV=1	19 kDa	4
Sorting nexin-2 OS=Mus musculus OX=10090 GN=Snx2 PE=1 SV=2	58 kDa	3
Sorting nexin-5 OS=Mus musculus OX=10090 GN=Snx5 PE=1 SV=1	47 kDa	1
Sorting nexin-6 OS=Mus musculus OX=10090 GN=Snx6 PE=1 SV=2	47 kDa	1
Sorting nexin-9 OS=Mus musculus OX=10090 GN=Snx9 PE=1 SV=1	67 kDa	6
SPARC OS=Mus musculus OX=10090 GN=Sparc PE=1 SV=1	38 kDa	1

Spastic paraplegia 20, spartin (Troyer syndrome) homolog (human) OS=Mus musculus OX=10090 GN=Spg20 PE=1 SV=1	63 kDa	4
Spectrin alpha chain, non-erythrocytic 1 OS=Mus musculus OX=10090 GN=Sptan1 PE=1 SV=4	285 kDa	39
Spectrin beta chain OS=Mus musculus OX=10090 GN=Sptbn4 PE=1 SV=1	289 kDa	5
Spectrin beta chain, non-erythrocytic 1 OS=Mus musculus OX=10090 GN=Sptbn1 PE=1 SV=2	274 kDa	10
Sperm-associated antigen 7 OS=Mus musculus OX=10090 GN=Spag7 PE=1 SV=1	26 kDa	9
Spermatogenesis-associated serine-rich protein 2 OS=Mus musculus OX=10090 GN=Spats2 PE=1 SV=1	59 kDa	1
Spermidine synthase OS=Mus musculus OX=10090 GN=Srm PE=1 SV=1	34 kDa	19
Spermine synthase OS=Mus musculus OX=10090 GN=Sms PE=1 SV=1	29 kDa	1
S-phase kinase-associated protein 1 OS=Mus musculus OX=10090 GN=Skp1 PE=1 SV=3	19 kDa	3
Sphingolipid delta(4)-desaturase DES1 OS=Mus musculus OX=10090 GN=Degs1 PE=1 SV=1	38 kDa	1
Sphingosine-1-phosphate lyase 1 OS=Mus musculus OX=10090 GN=Sgpl1 PE=1 SV=1	55 kDa	2
Spliceosome RNA helicase Ddx39b OS=Mus musculus OX=10090 GN=Ddx39b PE=1 SV=1	49 kDa	28
Spliceosome-associated protein CWC15 homolog OS=Mus musculus OX=10090 GN=Cwc15 PE=1 SV=1	27 kDa	5
Splicing factor 1 OS=Mus musculus OX=10090 GN=Sf1 PE=1 SV=1	60 kDa	33
Splicing factor 3A subunit 1 OS=Mus musculus OX=10090 GN=Sf3a1 PE=1 SV=1	89 kDa	18
Splicing factor 3A subunit 2 OS=Mus musculus OX=10090 GN=Sf3a2 PE=1 SV=1	51 kDa	6
Splicing factor 3A subunit 3 OS=Mus musculus OX=10090 GN=Sf3a3 PE=1 SV=2	59 kDa	5
Splicing factor 3B subunit 1 OS=Mus musculus OX=10090 GN=Sf3b1 PE=1 SV=1	146 kDa	3
Splicing factor 3B subunit 3 OS=Mus musculus OX=10090 GN=Sf3b3 PE=1 SV=1	136 kDa	50
Splicing factor 3B subunit 4 OS=Mus musculus OX=10090 GN=Sf3b4 PE=1 SV=1	44 kDa	3
Splicing factor 3B subunit 5 OS=Mus musculus OX=10090 GN=Sf3b5 PE=1 SV=1	10 kDa	2
Splicing factor 3B subunit 6 (Fragment) OS=Mus musculus OX=10090 GN=Sf3b6 PE=1 SV=1	13 kDa	2
Splicing factor 3b, subunit 2 OS=Mus musculus OX=10090 GN=Sf3b2 PE=1 SV=1	98 kDa	12
Splicing factor 45 OS=Mus musculus OX=10090 GN=Rbm17 PE=1 SV=1	45 kDa	2

Splicing factor U2AF 35 kDa subunit OS=Mus musculus OX=10090 GN=U2af1 PE=1 SV=4	28 kDa	6
Splicing factor, proline- and glutamine-rich OS=Mus musculus OX=10090 GN=Sfpq PE=1 SV=1	75 kDa	34
Squamous cell carcinoma antigen recognized by T-cells 3 OS=Mus musculus OX=10090 GN=Sart3 PE=1 SV=1	110 kDa	1
SRA stem-loop-interacting RNA-binding protein, mitochondrial (Fragment) OS=Mus musculus OX=10090 GN=Slirp PE=1 SV=1	11 kDa	1
Src substrate cortactin OS=Mus musculus OX=10090 GN=Cttn PE=1 SV=2	61 kDa	16
SR-related CTD-associated factor 11 OS=Mus musculus OX=10090 GN=Scaf11 PE=4 SV=1	154 kDa	3
SR-related CTD-associated factor 4 OS=Mus musculus OX=10090 GN=Scaf4 PE=1 SV=1	132 kDa	1
Staphylococcal nuclease domain-containing protein 1 OS=Mus musculus OX=10090 GN=Snd1 PE=1 SV=1	102 kDa	28
Stathmin OS=Mus musculus OX=10090 GN=Stmn1 PE=1 SV=2	17 kDa	35
STE20/SPS1-related proline-alanine-rich protein kinase OS=Mus musculus OX=10090 GN=Stk39 PE=1 SV=1	60 kDa	1
STE20-like serine/threonine-protein kinase OS=Mus musculus OX=10090 GN=Slk PE=1 SV=2	141 kDa	3
Steroid receptor RNA activator 1 (Fragment) OS=Mus musculus OX=10090 GN=Sra1 PE=1 SV=1	25 kDa	3
Stomatin-like protein 2, mitochondrial OS=Mus musculus OX=10090 GN=Stoml2 PE=1 SV=1	38 kDa	11
Stonin-1 OS=Mus musculus OX=10090 GN=Ston1 PE=1 SV=2	82 kDa	1
Stress-70 protein, mitochondrial OS=Mus musculus OX=10090 GN=Hspa9 PE=1 SV=3	73 kDa	107
Stress-induced-phosphoprotein 1 OS=Mus musculus OX=10090 GN=Stip1 PE=1 SV=1	63 kDa	117
Striatin OS=Mus musculus OX=10090 GN=Strn PE=1 SV=1	69 kDa	6
Striatin-3 OS=Mus musculus OX=10090 GN=Strn3 PE=1 SV=1	78 kDa	4
Striatin-4 OS=Mus musculus OX=10090 GN=Strn4 PE=1 SV=2	82 kDa	2
Stromal cell-derived factor 2-like protein 1 OS=Mus musculus OX=10090 GN=Sdf2l1 PE=1 SV=2	24 kDa	2
Stromal membrane-associated protein 1 OS=Mus musculus OX=10090 GN=Smap1 PE=1 SV=1	48 kDa	3
Stromal membrane-associated protein 1 OS=Mus musculus OX=10090 GN=Smap1 PE=1 SV=1	38 kDa	2
Structural maintenance of chromosomes flexible hinge domain-containing protein 1 OS=Mus musculus OX=10090 GN=Smchd1 PE=1 SV=2	226 kDa	1
Structural maintenance of chromosomes protein 1A OS=Mus musculus OX=10090 GN=Smc1a PE=1 SV=4	143 kDa	13

Structural maintenance of chromosomes protein 2 OS=Mus musculus OX=10090 GN=Smc2 PE=1 SV=2	134 kDa	11
Structural maintenance of chromosomes protein 3 OS=Mus musculus OX=10090 GN=Smc3 PE=1 SV=2	142 kDa	12
Structural maintenance of chromosomes protein OS=Mus musculus OX=10090 GN=Smc4 PE=1 SV=1	144 kDa	4
Succinate dehydrogenase [ubiquinone] flavoprotein subunit, mitochondrial OS=Mus musculus OX=10090 GN=Sdha PE=1 SV=1	73 kDa	42
Succinate dehydrogenase [ubiquinone] iron-sulfur subunit, mitochondrial OS=Mus musculus OX=10090 GN=Sdhb PE=1 SV=1	32 kDa	17
Succinate--CoA ligase [ADP/GDP-forming] subunit alpha, mitochondrial OS=Mus musculus OX=10090 GN=Suclg1 PE=1 SV=4	36 kDa	4
Succinate--CoA ligase [GDP-forming] subunit beta, mitochondrial OS=Mus musculus OX=10090 GN=Suclg2 PE=1 SV=3	47 kDa	7
Succinate-semialdehyde dehydrogenase, mitochondrial OS=Mus musculus OX=10090 GN=Aldh5a1 PE=1 SV=1	56 kDa	2
Succinyl-CoA:3-ketoacid coenzyme A transferase 1, mitochondrial OS=Mus musculus OX=10090 GN=Oxct1 PE=1 SV=1	56 kDa	7
Sulfatase-modifying factor 2 OS=Mus musculus OX=10090 GN=Sumf2 PE=1 SV=2	35 kDa	3
Sulfurtransferase OS=Mus musculus OX=10090 GN=Mpst PE=1 SV=1	33 kDa	1
SUMO/sentrin specific peptidase 8, isoform CRA_a OS=Mus musculus OX=10090 GN=Senp8 PE=1 SV=2	25 kDa	1
SUMO-activating enzyme subunit 1 OS=Mus musculus OX=10090 GN=Sae1 PE=1 SV=1	39 kDa	20
SUMO-activating enzyme subunit 2 OS=Mus musculus OX=10090 GN=Uba2 PE=1 SV=1	71 kDa	16
SUMO-conjugating enzyme UBC9 (Fragment) OS=Mus musculus OX=10090 GN=Ube2i PE=1 SV=1	12 kDa	11
Superoxide dismutase [Cu-Zn] OS=Mus musculus OX=10090 GN=Sod1 PE=1 SV=2	16 kDa	38
Superoxide dismutase [Mn], mitochondrial OS=Mus musculus OX=10090 GN=Sod2 PE=1 SV=3	25 kDa	4
Suppressor of Ty 16 OS=Mus musculus OX=10090 GN=Supt16 PE=1 SV=1	120 kDa	9
Surfeit locus protein 4 OS=Mus musculus OX=10090 GN=Surf4 PE=1 SV=1	10 kDa	2
SUZ domain-containing protein 1 (Fragment) OS=Mus musculus OX=10090 GN=Szrd1 PE=1 SV=1	16 kDa	3
SWI/SNF complex subunit SMARCC1 OS=Mus musculus OX=10090 GN=Smarcc1 PE=1 SV=1	123 kDa	5
SWI/SNF complex subunit SMARCC2 OS=Mus musculus OX=10090 GN=Smarcc2 PE=1 SV=1	125 kDa	3

SWI/SNF-related matrix-associated actin-dependent regulator of chromatin subfamily A member 5 OS=Mus musculus OX=10090 GN=Smarca5 PE=1 SV=1	122 kDa	4
SWI/SNF-related matrix-associated actin-dependent regulator of chromatin subfamily E member 1 OS=Mus musculus OX=10090 GN=Smarce1 PE=1 SV=1	47 kDa	4
Swi5-dependent recombination DNA repair protein 1 homolog OS=Mus musculus OX=10090 GN=Sfr1 PE=1 SV=2	35 kDa	10
Switch-associated protein 70 OS=Mus musculus OX=10090 GN=Swap70 PE=1 SV=2	69 kDa	1
Synaptic functional regulator FMR1 OS=Mus musculus OX=10090 GN=Fmr1 PE=1 SV=1	67 kDa	1
Synaptic vesicle membrane protein VAT-1 homolog OS=Mus musculus OX=10090 GN=Vat1 PE=1 SV=3	43 kDa	41
Synaptobrevin homolog YKT6 OS=Mus musculus OX=10090 GN=Ykt6 PE=1 SV=1	22 kDa	1
Synaptophysin-like protein OS=Mus musculus OX=10090 GN=Sypl PE=1 SV=1	9 kDa	1
Synaptosomal-associated protein (Fragment) OS=Mus musculus OX=10090 GN=Snap23 PE=1 SV=8	18 kDa	4
Synaptosomal-associated protein 29 OS=Mus musculus OX=10090 GN=Snap29 PE=1 SV=1	30 kDa	1
Syndecan-1 OS=Mus musculus OX=10090 GN=Sdc1 PE=1 SV=1	33 kDa	1
Syntaxin-12 OS=Mus musculus OX=10090 GN=Stx12 PE=1 SV=1	31 kDa	5
Syntaxin-6 OS=Mus musculus OX=10090 GN=Stx6 PE=1 SV=1	23 kDa	2
Syntaxin-7 OS=Mus musculus OX=10090 GN=Stx7 PE=1 SV=1	30 kDa	2
Syntaxin-8 OS=Mus musculus OX=10090 GN=Stx8 PE=1 SV=1	27 kDa	1
TAF15 RNA polymerase II, TATA box binding protein (TBP)-associated factor OS=Mus musculus OX=10090 GN=Taf15 PE=1 SV=1	59 kDa	9
Talin-1 OS=Mus musculus OX=10090 GN=Tln1 PE=1 SV=2	270 kDa	38
Talin-2 OS=Mus musculus OX=10090 GN=Tln2 PE=1 SV=1	272 kDa	4
TAR DNA-binding protein 43 OS=Mus musculus OX=10090 GN=Tardbp PE=1 SV=1	45 kDa	10
Target of EGR1 protein 1 OS=Mus musculus OX=10090 GN=Toe1 PE=1 SV=1	57 kDa	1
Target of Myb protein 1 OS=Mus musculus OX=10090 GN=Tom1 PE=1 SV=1	57 kDa	1
TBC1 domain family member 15 OS=Mus musculus OX=10090 GN=Tbc1d15 PE=1 SV=1	77 kDa	2
T-complex protein 1 subunit alpha OS=Mus musculus OX=10090 GN=Tcp1 PE=1 SV=3	60 kDa	92

T-complex protein 1 subunit beta OS=Mus musculus OX=10090 GN=Cct2 PE=1 SV=4	57 kDa	109
T-complex protein 1 subunit delta OS=Mus musculus OX=10090 GN=Cct4 PE=1 SV=3	58 kDa	92
T-complex protein 1 subunit epsilon (Fragment) OS=Mus musculus OX=10090 GN=Cct5 PE=1 SV=1	22 kDa	16
T-complex protein 1 subunit epsilon OS=Mus musculus OX=10090 GN=Cct5 PE=1 SV=1	60 kDa	85
T-complex protein 1 subunit eta OS=Mus musculus OX=10090 GN=Cct7 PE=1 SV=1	60 kDa	79
T-complex protein 1 subunit gamma OS=Mus musculus OX=10090 GN=Cct3 PE=1 SV=1	61 kDa	104
T-complex protein 1 subunit theta OS=Mus musculus OX=10090 GN=Cct8 PE=1 SV=3	60 kDa	124
T-complex protein 1 subunit zeta OS=Mus musculus OX=10090 GN=Cct6a PE=1 SV=3	58 kDa	51
Telomerase RNA component interacting RNase OS=Mus musculus OX=10090 GN=Trir PE=1 SV=1	18 kDa	4
Telomere length and silencing protein 1 homolog OS=Mus musculus OX=10090 PE=1 SV=2	34 kDa	1
Testin OS=Mus musculus OX=10090 GN=Tes PE=1 SV=1	47 kDa	30
Testis-expressed gene 264 OS=Mus musculus OX=10090 GN=Tex264 PE=1 SV=1	34 kDa	3
Tether containing UBX domain for GLUT4 OS=Mus musculus OX=10090 GN=Aspscr1 PE=1 SV=1	60 kDa	2
Tetraspanin (Fragment) OS=Mus musculus OX=10090 GN=Cd63 PE=1 SV=1	18 kDa	5
Tetraspanin-31 OS=Mus musculus OX=10090 GN=Tspan31 PE=1 SV=1	23 kDa	1
Tetratricopeptide repeat protein 1 OS=Mus musculus OX=10090 GN=Ttc1 PE=1 SV=1	29 kDa	4
TFG protein OS=Mus musculus OX=10090 GN=Tfg PE=1 SV=1	43 kDa	6
Thimet oligopeptidase OS=Mus musculus OX=10090 GN=Thop1 PE=1 SV=1	78 kDa	27
Thioredoxin domain-containing protein 15 OS=Mus musculus OX=10090 GN=Txndc15 PE=1 SV=1	38 kDa	2
Thioredoxin domain-containing protein 17 OS=Mus musculus OX=10090 GN=Txndc17 PE=1 SV=1	14 kDa	11
Thioredoxin domain-containing protein 5 OS=Mus musculus OX=10090 GN=Txndc5 PE=1 SV=2	46 kDa	42
Thioredoxin domain-containing protein 9 (Fragment) OS=Mus musculus OX=10090 GN=Txndc9 PE=1 SV=1	20 kDa	2
Thioredoxin OS=Mus musculus OX=10090 GN=Txn PE=1 SV=3	12 kDa	54
Thioredoxin reductase 1, cytoplasmic OS=Mus musculus OX=10090 GN=Txnrd1 PE=1 SV=3	67 kDa	30
Thioredoxin reductase 2, mitochondrial OS=Mus musculus OX=10090 GN=Txnrd2 PE=1 SV=1	53 kDa	7

Thioredoxin, mitochondrial OS=Mus musculus OX=10090 GN=Txn2 PE=1 SV=1	16 kDa	3
Thioredoxin-dependent peroxide reductase, mitochondrial OS=Mus musculus OX=10090 GN=Prdx3 PE=1 SV=1	28 kDa	19
Thioredoxin-like protein 1 OS=Mus musculus OX=10090 GN=Txnl1 PE=1 SV=3	32 kDa	14
Thioredoxin-related transmembrane protein 1 OS=Mus musculus OX=10090 GN=Tmx1 PE=1 SV=1	31 kDa	5
THO complex subunit 4 OS=Mus musculus OX=10090 GN=Alyref PE=1 SV=3	27 kDa	13
Threonine--tRNA ligase, cytoplasmic OS=Mus musculus OX=10090 GN=Tars PE=1 SV=2	83 kDa	21
THUMP domain-containing protein 1 OS=Mus musculus OX=10090 GN=Thumpd1 PE=1 SV=1	39 kDa	9
THUMP domain-containing protein 2 OS=Mus musculus OX=10090 GN=Thumpd2 PE=2 SV=3	58 kDa	1
THUMP domain-containing protein 3 OS=Mus musculus OX=10090 GN=Thumpd3 PE=1 SV=1	56 kDa	3
Thymidine kinase, cytosolic OS=Mus musculus OX=10090 GN=Tk1 PE=1 SV=3	26 kDa	4
Thymidylate kinase OS=Mus musculus OX=10090 GN=Dtymk PE=1 SV=2	24 kDa	6
Thymosin beta-10 OS=Mus musculus OX=10090 GN=Tmsb10 PE=1 SV=3	5 kDa	5
Thyroid hormone receptor interactor 11 OS=Mus musculus OX=10090 GN=Trip11 PE=1 SV=1	226 kDa	1
Thyroid hormone receptor-associated protein 3 OS=Mus musculus OX=10090 GN=Thrap3 PE=1 SV=1	108 kDa	4
Tight junction protein ZO-1 OS=Mus musculus OX=10090 GN=Tjp1 PE=1 SV=1	189 kDa	10
TM2 domain-containing protein 2 OS=Mus musculus OX=10090 GN=Tm2d2 PE=2 SV=1	23 kDa	1
Torsin-1A-interacting protein 1 OS=Mus musculus OX=10090 GN=Tor1aip1 PE=1 SV=3	67 kDa	5
TP53-binding protein 1 OS=Mus musculus OX=10090 GN=Tp53bp1 PE=1 SV=3	213 kDa	4
Trafficking protein particle complex subunit 12 OS=Mus musculus OX=10090 GN=Trappc12 PE=1 SV=2	88 kDa	1
Trafficking protein particle complex subunit 3 OS=Mus musculus OX=10090 GN=Trappc3 PE=1 SV=1	20 kDa	2
Trafficking protein particle complex subunit 6B OS=Mus musculus OX=10090 GN=Trappc6b PE=1 SV=1	14 kDa	2
Trans-acting transcription factor 1 OS=Mus musculus OX=10090 GN=Sp1 PE=1 SV=1	80 kDa	1
Transaldolase OS=Mus musculus OX=10090 GN=Taldo1 PE=1 SV=1	42 kDa	32
Transcription activator BRG1 OS=Mus musculus OX=10090 GN=Smarca4 PE=1 SV=1	182 kDa	4
Transcription and mRNA export factor ENY2 OS=Mus musculus OX=10090 GN=Eny2 PE=4 SV=1	11 kDa	6

Transcription elongation factor A protein 1 OS=Mus musculus OX=10090 GN=Tcea1 PE=1 SV=2	34 kDa	24
Transcription elongation factor A protein 2 (Fragment) OS=Mus musculus OX=10090 GN=Tcea2 PE=4 SV=1	33 kDa	2
Transcription elongation factor SPT5 OS=Mus musculus OX=10090 GN=Supt5h PE=1 SV=1	121 kDa	3
Transcription elongation regulator 1 OS=Mus musculus OX=10090 GN=Tcerg1 PE=1 SV=2	124 kDa	2
Transcription factor 12 OS=Mus musculus OX=10090 GN=Tcf12 PE=1 SV=2	76 kDa	1
Transcription factor A, mitochondrial OS=Mus musculus OX=10090 GN=Tfam PE=1 SV=1	23 kDa	1
Transcription factor BTF3 homolog 4 OS=Mus musculus OX=10090 GN=Btf3l4 PE=1 SV=1	17 kDa	2
Transcription factor BTF3 OS=Mus musculus OX=10090 GN=Btf3 PE=1 SV=3	22 kDa	27
Transcription factor E2-alpha OS=Mus musculus OX=10090 GN=Tcf3 PE=1 SV=1	68 kDa	1
Transcription factor jun-D OS=Mus musculus OX=10090 GN=Jund PE=1 SV=1	35 kDa	1
Transcription factor p65 OS=Mus musculus OX=10090 GN=Rela PE=1 SV=1	60 kDa	3
Transcription factor Sp3 OS=Mus musculus OX=10090 GN=Sp3 PE=1 SV=2	82 kDa	1
Transcription initiation factor IIA subunit 2 (Fragment) OS=Mus musculus OX=10090 GN=Gtf2a2 PE=1 SV=1	11 kDa	1
Transcription initiation factor TFIID subunit 10 OS=Mus musculus OX=10090 GN=Taf10 PE=1 SV=1	22 kDa	1
Transcription initiation factor TFIID subunit 8 OS=Mus musculus OX=10090 GN=Taf8 PE=2 SV=1	34 kDa	1
Transcription intermediary factor 1-beta OS=Mus musculus OX=10090 GN=Trim28 PE=1 SV=3	89 kDa	57
Transcriptional activator protein Pur-alpha OS=Mus musculus OX=10090 GN=Pura PE=1 SV=1	35 kDa	5
Transcriptional activator protein Pur-beta OS=Mus musculus OX=10090 GN=Purb PE=1 SV=3	34 kDa	10
Transcriptional coactivator YAP1 (Fragment) OS=Mus musculus OX=10090 GN=Yap1 PE=1 SV=1	43 kDa	2
Transcriptional coactivator YAP1 OS=Mus musculus OX=10090 GN=Yap1 PE=1 SV=2	52 kDa	7
Transcriptional repressor p66 alpha OS=Mus musculus OX=10090 GN=Gatad2a PE=1 SV=1	67 kDa	3
Transcriptional repressor protein YY1 OS=Mus musculus OX=10090 GN=Yy1 PE=1 SV=1	45 kDa	3
Transferrin receptor protein 1 OS=Mus musculus OX=10090 GN=Tfrc PE=1 SV=1	86 kDa	40
Transformer-2 protein homolog alpha OS=Mus musculus OX=10090 GN=Tra2a PE=1 SV=1	32 kDa	2
Transformer-2 protein homolog beta OS=Mus musculus OX=10090 GN=Tra2b PE=1 SV=1	34 kDa	3

Transforming growth factor beta receptor type 3 OS=Mus musculus OX=10090 GN=Tgfbr3 PE=1 SV=1	94 kDa	1
Transforming protein RhoA OS=Mus musculus OX=10090 GN=Rhoa PE=1 SV=1	22 kDa	36
Transgelin-2 OS=Mus musculus OX=10090 GN=Tagln2 PE=1 SV=4	22 kDa	58
Transgelin-3 OS=Mus musculus OX=10090 GN=Tagln3 PE=1 SV=1	22 kDa	11
Transitional endoplasmic reticulum ATPase OS=Mus musculus OX=10090 GN=Vcp PE=1 SV=4	89 kDa	162
Transketolase OS=Mus musculus OX=10090 GN=Tkt PE=1 SV=1	68 kDa	123
Trans-L-3-hydroxyproline dehydratase OS=Mus musculus OX=10090 GN=L3hypdh PE=1 SV=1	38 kDa	2
Translation initiation factor eIF-2B subunit epsilon OS=Mus musculus OX=10090 GN=Eif2b5 PE=1 SV=1	80 kDa	3
Translation machinery-associated protein 7 OS=Mus musculus OX=10090 GN=Tma7 PE=3 SV=1	7 kDa	7
Translationally-controlled tumor protein OS=Mus musculus OX=10090 GN=Tpt1 PE=1 SV=1	19 kDa	40
Translin OS=Mus musculus OX=10090 GN=Tsn PE=1 SV=1	26 kDa	20
Translin-associated protein X OS=Mus musculus OX=10090 GN=Tsnax PE=1 SV=1	33 kDa	11
Translocation protein SEC62 OS=Mus musculus OX=10090 GN=Sec62 PE=1 SV=1	46 kDa	3
Translocon-associated protein subunit alpha OS=Mus musculus OX=10090 GN=Ssr1 PE=1 SV=1	32 kDa	7
Translocon-associated protein subunit gamma OS=Mus musculus OX=10090 GN=Ssr3 PE=1 SV=1	21 kDa	3
Transmembrane emp24 domain-containing protein 10 OS=Mus musculus OX=10090 GN=Tmed10 PE=1 SV=1	25 kDa	5
Transmembrane emp24 domain-containing protein 2 (Fragment) OS=Mus musculus OX=10090 GN=Tmed2 PE=1 SV=1	20 kDa	1
Transmembrane emp24 domain-containing protein 4 OS=Mus musculus OX=10090 GN=Tmed4 PE=1 SV=1	26 kDa	4
Transmembrane emp24 domain-containing protein 9 OS=Mus musculus OX=10090 GN=Tmed9 PE=1 SV=2	27 kDa	8
Transmembrane glycoprotein NMB OS=Mus musculus OX=10090 GN=Gpnmb PE=1 SV=2	64 kDa	1
Transmembrane p24-trafficking protein 7 OS=Mus musculus OX=10090 GN=Tmed7 PE=1 SV=1	25 kDa	11
Transmembrane protein 106B (Fragment) OS=Mus musculus OX=10090 GN=Tmem106b PE=1 SV=8	21 kDa	3
Transmembrane protein 109 (Fragment) OS=Mus musculus OX=10090 GN=Tmem109 PE=1 SV=1	10 kDa	3
Transmembrane protein 14C OS=Mus musculus OX=10090 GN=Tmem14c PE=1 SV=1	12 kDa	2
Transmembrane protein 179B OS=Mus musculus OX=10090 GN=Tmem179b PE=1 SV=1	24 kDa	1

Transmembrane protein 237 (Fragment) OS=Mus musculus OX=10090 GN=Tmem237 PE=1 SV=1	26 kDa	1
Transmembrane protein 263 OS=Mus musculus OX=10090 GN=Tmem263 PE=1 SV=1	12 kDa	1
Transmembrane protein 43 OS=Mus musculus OX=10090 GN=Tmem43 PE=1 SV=1	45 kDa	9
Transmembrane protein 59 OS=Mus musculus OX=10090 GN=Tmem59 PE=1 SV=1	29 kDa	1
Transport and Golgi organization protein 1 homolog OS=Mus musculus OX=10090 GN=Mia3 PE=1 SV=1	87 kDa	5
Transport and Golgi organization protein 1 homolog OS=Mus musculus OX=10090 GN=Mia3 PE=1 SV=2	214 kDa	16
Transportin-1 OS=Mus musculus OX=10090 GN=Tnpo1 PE=1 SV=2	102 kDa	13
Transportin-2 OS=Mus musculus OX=10090 GN=Tnpo2 PE=1 SV=1	100 kDa	6
Treacle protein OS=Mus musculus OX=10090 GN=Tcof1 PE=1 SV=1	139 kDa	18
Trifunctional enzyme subunit alpha, mitochondrial OS=Mus musculus OX=10090 GN=Hadha PE=1 SV=1	83 kDa	2
Trifunctional enzyme subunit beta, mitochondrial OS=Mus musculus OX=10090 GN=Hadhb PE=1 SV=1	51 kDa	2
Trifunctional purine biosynthetic protein adenosine-3 OS=Mus musculus OX=10090 GN=Gart PE=1 SV=3	108 kDa	26
Trimethyllysine dioxygenase, mitochondrial OS=Mus musculus OX=10090 GN=Tmlhe PE=1 SV=2	50 kDa	1
TRIO and F-actin-binding protein OS=Mus musculus OX=10090 GN=Triobp PE=1 SV=3	223 kDa	3
Triosephosphate isomerase OS=Mus musculus OX=10090 GN=Tpi1 PE=1 SV=4	32 kDa	97
Tripartite motif-containing protein 47 OS=Mus musculus OX=10090 GN=Trim47 PE=1 SV=2	70 kDa	1
Tripeptidyl-peptidase 1 OS=Mus musculus OX=10090 GN=Tpp1 PE=1 SV=2	61 kDa	2
Tripeptidyl-peptidase 2 OS=Mus musculus OX=10090 GN=Tpp2 PE=1 SV=3	140 kDa	35
Trk-fused OS=Mus musculus OX=10090 GN=Tfg PE=1 SV=1	16 kDa	3
tRNA (adenine(58)-N(1))-methyltransferase catalytic subunit TRMT61A OS=Mus musculus OX=10090 GN=Trmt61a PE=1 SV=1	32 kDa	1
tRNA (adenine(58)-N(1))-methyltransferase non-catalytic subunit TRM6 OS=Mus musculus OX=10090 GN=Trmt6 PE=1 SV=1	56 kDa	3
tRNA (cytosine(34)-C(5))-methyltransferase OS=Mus musculus OX=10090 GN=Nsun2 PE=1 SV=2	85 kDa	41
tRNA (guanine(26)-N(2))-dimethyltransferase OS=Mus musculus OX=10090 GN=Trmt1 PE=1 SV=1	72 kDa	4

tRNA (guanine-N(7)-)-methyltransferase non-catalytic subunit WDR4 OS=Mus musculus OX=10090 GN=Wdr4 PE=1 SV=1	50 kDa	1
tRNA (guanine-N(7)-)-methyltransferase OS=Mus musculus OX=10090 GN=Mettl1 PE=1 SV=1	31 kDa	3
tRNA-dihydrouridine(47) synthase [NAD(P)(+)] OS=Mus musculus OX=10090 GN=Dus3l PE=1 SV=1	71 kDa	4
tRNA-splicing endonuclease subunit Sen15 OS=Mus musculus OX=10090 GN=Tsen15 PE=1 SV=1	14 kDa	1
tRNA-splicing endonuclease subunit Sen34 (Fragment) OS=Mus musculus OX=10090 GN=Tsen34 PE=1 SV=1	17 kDa	1
tRNA-splicing ligase RtcB homolog OS=Mus musculus OX=10090 GN=Rtcb PE=1 SV=1	55 kDa	9
Tropomodulin-3 OS=Mus musculus OX=10090 GN=Tmod3 PE=1 SV=1	40 kDa	5
Tropomyosin 1, alpha, isoform CRA_k OS=Mus musculus OX=10090 GN=Tpm1 PE=1 SV=1	29 kDa	55
Tropomyosin 3, related sequence 7 OS=Mus musculus OX=10090 GN=Tpm3-rs7 PE=3 SV=1	29 kDa	104
Tropomyosin alpha-1 chain OS=Mus musculus OX=10090 GN=Tpm1 PE=1 SV=1	32 kDa	72
Tropomyosin alpha-3 chain OS=Mus musculus OX=10090 GN=Tpm3 PE=1 SV=1	29 kDa	87
Tropomyosin alpha-3 chain OS=Mus musculus OX=10090 GN=Tpm3 PE=1 SV=1	33 kDa	86
Tropomyosin alpha-4 chain OS=Mus musculus OX=10090 GN=Tpm4 PE=1 SV=3	28 kDa	55
Tropomyosin beta chain OS=Mus musculus OX=10090 GN=Tpm2 PE=1 SV=1	33 kDa	47
Trypsin OS=Sus scrofa PE=1 SV=1	24 kDa	5
Tryptophan--tRNA ligase, cytoplasmic OS=Mus musculus OX=10090 GN=Wars PE=1 SV=2	54 kDa	32
Tubulin alpha chain (Fragment) OS=Mus musculus OX=10090 GN=Tuba4a PE=1 SV=1	53 kDa	129
Tubulin alpha-1C chain OS=Mus musculus OX=10090 GN=Tuba1c PE=1 SV=1	50 kDa	132
Tubulin beta-2B chain OS=Mus musculus OX=10090 GN=Tubb2b PE=1 SV=1	50 kDa	254
Tubulin beta-4B chain OS=Mus musculus OX=10090 GN=Tubb4b PE=1 SV=1	50 kDa	292
Tubulin beta-5 chain OS=Mus musculus OX=10090 GN=Tubb5 PE=1 SV=1	50 kDa	312
Tubulin beta-6 chain OS=Mus musculus OX=10090 GN=Tubb6 PE=1 SV=1	50 kDa	140
Tubulin gamma-1 chain OS=Mus musculus OX=10090 GN=Tubg1 PE=1 SV=1	51 kDa	1
Tubulin polymerization-promoting protein family member 3 OS=Mus musculus OX=10090 GN=Tppp3 PE=1 SV=1	19 kDa	1
Tubulin-folding cofactor B OS=Mus musculus OX=10090 GN=Tbcb PE=1 SV=2	27 kDa	1

Tubulin-specific chaperone A OS=Mus musculus OX=10090 GN=Tbca PE=1 SV=1	10 kDa	10
Tubulin-specific Chaperone C OS=Mus musculus OX=10090 GN=Tbcc PE=1 SV=1	38 kDa	2
Tubulin-specific chaperone D OS=Mus musculus OX=10090 GN=Tbcd PE=1 SV=1	133 kDa	1
Tudor domain-containing protein 5 OS=Mus musculus OX=10090 GN=Tdrd5 PE=1 SV=1	107 kDa	3
Tumor necrosis factor alpha-induced protein 2 OS=Mus musculus OX=10090 GN=Tnfaip2 PE=1 SV=2	80 kDa	1
Tumor protein D52 OS=Mus musculus OX=10090 GN=Tpd52 PE=1 SV=1	21 kDa	7
Tumor protein D54 OS=Mus musculus OX=10090 GN=Tpd52l2 PE=1 SV=1	25 kDa	12
Twinfilin-1 OS=Mus musculus OX=10090 GN=Twf1 PE=1 SV=2	40 kDa	11
Twinfilin-2 OS=Mus musculus OX=10090 GN=Twf2 PE=1 SV=1	39 kDa	4
Tyrosine-protein kinase BAZ1B OS=Mus musculus OX=10090 GN=Baz1b PE=1 SV=2	171 kDa	1
Tyrosine-protein kinase OS=Mus musculus OX=10090 GN=Hck PE=1 SV=1	59 kDa	2
Tyrosine-protein phosphatase non-receptor type 23 OS=Mus musculus OX=10090 GN=Ptpn23 PE=1 SV=2	185 kDa	1
Tyrosine--tRNA ligase, cytoplasmic OS=Mus musculus OX=10090 GN=Yars PE=1 SV=3	59 kDa	14
U1 small nuclear ribonucleoprotein 70 kDa OS=Mus musculus OX=10090 GN=Snrnp70 PE=1 SV=1	13 kDa	4
U1 small nuclear ribonucleoprotein 70 kDa OS=Mus musculus OX=10090 GN=Snrnp70 PE=1 SV=2	52 kDa	9
U1 small nuclear ribonucleoprotein A OS=Mus musculus OX=10090 GN=Snrpa PE=1 SV=3	32 kDa	14
U1 small nuclear ribonucleoprotein C OS=Mus musculus OX=10090 GN=Snrpc PE=1 SV=1	17 kDa	6
U2 small nuclear ribonucleoprotein A' OS=Mus musculus OX=10090 GN=Snrpa1 PE=1 SV=2	28 kDa	7
U2 small nuclear ribonucleoprotein B'' OS=Mus musculus OX=10090 GN=Snrpb2 PE=1 SV=1	25 kDa	10
U2 snRNP auxiliary factor large subunit OS=Mus musculus OX=10090 GN=U2af2 PE=1 SV=1	53 kDa	4
U2 snRNP-associated SURP motif-containing protein OS=Mus musculus OX=10090 GN=U2surp PE=1 SV=3	118 kDa	3
U3 small nucleolar RNA-associated protein 18 homolog OS=Mus musculus OX=10090 GN=Utp18 PE=1 SV=1	61 kDa	1
U3 small nucleolar RNA-associated protein 4 homolog OS=Mus musculus OX=10090 GN=Utp4 PE=2 SV=3	77 kDa	1
U3 small nucleolar RNA-interacting protein 2 OS=Mus musculus OX=10090 GN=Rrp9 PE=1 SV=1	52 kDa	4
U4/U6 small nuclear ribonucleoprotein Prp3 OS=Mus musculus OX=10090 GN=Prpf3 PE=1 SV=1	77 kDa	1

U4/U6 small nuclear ribonucleoprotein Prp4 OS=Mus musculus OX=10090 GN=Prpf4 PE=1 SV=1	58 kDa	8
U4/U6.U5 tri-snRNP-associated protein 1 OS=Mus musculus OX=10090 GN=Sart1 PE=1 SV=1	91 kDa	5
U5 small nuclear ribonucleoprotein 200 kDa helicase OS=Mus musculus OX=10090 GN=Snrnp200 PE=1 SV=1	245 kDa	10
U5 small nuclear ribonucleoprotein 40 kDa protein OS=Mus musculus OX=10090 GN=Snrnp40 PE=1 SV=1	39 kDa	3
U6 snRNA-associated Sm-like protein LSm2 OS=Mus musculus OX=10090 GN=Lsm2 PE=1 SV=1	11 kDa	4
U6 snRNA-associated Sm-like protein LSm3 OS=Mus musculus OX=10090 GN=Lsm3 PE=1 SV=2	12 kDa	2
U6 snRNA-associated Sm-like protein LSm4 OS=Mus musculus OX=10090 GN=Lsm4 PE=1 SV=1	15 kDa	4
U6 snRNA-associated Sm-like protein LSm5 OS=Mus musculus OX=10090 GN=Lsm5 PE=1 SV=1	7 kDa	1
U6 snRNA-associated Sm-like protein LSm6 OS=Mus musculus OX=10090 GN=Lsm6 PE=1 SV=1	9 kDa	7
U6 snRNA-associated Sm-like protein LSm8 OS=Mus musculus OX=10090 GN=Lsm8 PE=1 SV=3	10 kDa	6
Ubiquilin-1 OS=Mus musculus OX=10090 GN=Ubqln1 PE=1 SV=1	62 kDa	17
Ubiquilin-2 OS=Mus musculus OX=10090 GN=Ubqln2 PE=1 SV=2	67 kDa	25
Ubiquinone biosynthesis protein COQ9, mitochondrial OS=Mus musculus OX=10090 GN=Coq9 PE=1 SV=1	35 kDa	1
Ubiquitin carboxyl-terminal hydrolase 10 OS=Mus musculus OX=10090 GN=Usp10 PE=1 SV=3	87 kDa	1
Ubiquitin carboxyl-terminal hydrolase 14 OS=Mus musculus OX=10090 GN=Usp14 PE=1 SV=1	52 kDa	12
Ubiquitin carboxyl-terminal hydrolase 15 OS=Mus musculus OX=10090 GN=Usp15 PE=1 SV=1	112 kDa	1
Ubiquitin carboxyl-terminal hydrolase 19 OS=Mus musculus OX=10090 GN=Usp19 PE=1 SV=1	146 kDa	2
Ubiquitin carboxyl-terminal hydrolase 47 OS=Mus musculus OX=10090 GN=Usp47 PE=1 SV=1	157 kDa	4
Ubiquitin carboxyl-terminal hydrolase isozyme L3 OS=Mus musculus OX=10090 GN=Uchl3 PE=1 SV=2	26 kDa	8
Ubiquitin carboxyl-terminal hydrolase isozyme L5 OS=Mus musculus OX=10090 GN=Uchl5 PE=1 SV=2	38 kDa	5
Ubiquitin domain-containing protein UBFD1 OS=Mus musculus OX=10090 GN=Ubfd1 PE=1 SV=2	40 kDa	2
Ubiquitin recognition factor in ER-associated degradation protein 1 OS=Mus musculus OX=10090 GN=Ufd1 PE=1 SV=2	34 kDa	6
Ubiquitin thioesterase OTUB1 OS=Mus musculus OX=10090 GN=Otub1 PE=1 SV=1	28 kDa	8
Ubiquitin-40S ribosomal protein S27a OS=Mus musculus OX=10090 GN=Rps27a PE=1 SV=2	18 kDa	88

Ubiquitin-associated protein 2 OS=Mus musculus OX=10090 GN=Ubap2 PE=1 SV=1	118 kDa	13
Ubiquitin-associated protein 2-like OS=Mus musculus OX=10090 GN=Ubap2l PE=1 SV=1	117 kDa	26
Ubiquitin-conjugating enzyme E2 A OS=Mus musculus OX=10090 GN=Ube2a PE=1 SV=1	14 kDa	1
Ubiquitin-conjugating enzyme E2 C OS=Mus musculus OX=10090 GN=Ube2c PE=1 SV=1	18 kDa	2
Ubiquitin-conjugating enzyme E2 H OS=Mus musculus OX=10090 GN=Ube2h PE=1 SV=1	21 kDa	3
Ubiquitin-conjugating enzyme E2 K OS=Mus musculus OX=10090 GN=Ube2k PE=1 SV=1	16 kDa	7
Ubiquitin-conjugating enzyme E2 L3 OS=Mus musculus OX=10090 GN=Ube2l3 PE=1 SV=1	18 kDa	18
Ubiquitin-conjugating enzyme E2 N OS=Mus musculus OX=10090 GN=Ube2n PE=1 SV=1	17 kDa	14
Ubiquitin-conjugating enzyme E2 R1 OS=Mus musculus OX=10090 GN=Cdc34 PE=1 SV=1	21 kDa	1
Ubiquitin-conjugating enzyme E2 R2 OS=Mus musculus OX=10090 GN=Ube2r2 PE=1 SV=1	27 kDa	1
Ubiquitin-conjugating enzyme E2 variant 1 OS=Mus musculus OX=10090 GN=Ube2v1 PE=1 SV=1	16 kDa	9
Ubiquitin-conjugating enzyme E2 variant 2 OS=Mus musculus OX=10090 GN=Ube2v2 PE=1 SV=4	16 kDa	6
Ubiquitin-conjugating enzyme E2 variant 3 OS=Mus musculus OX=10090 GN=Uevld PE=1 SV=1	52 kDa	1
Ubiquitin-conjugating enzyme E2 Z OS=Mus musculus OX=10090 GN=Ube2z PE=1 SV=2	38 kDa	2
Ubiquitin-fold modifier 1 OS=Mus musculus OX=10090 GN=Ufm1 PE=1 SV=1	9 kDa	1
Ubiquitin-fold modifier-conjugating enzyme 1 OS=Mus musculus OX=10090 GN=Ufc1 PE=1 SV=1	19 kDa	5
Ubiquitin-like modifier-activating enzyme 1 OS=Mus musculus OX=10090 GN=Uba1 PE=1 SV=1	118 kDa	32
Ubiquitin-like modifier-activating enzyme 1 Y OS=Mus musculus OX=10090 GN=Uba1y PE=1 SV=2	118 kDa	2
Ubiquitin-like modifier-activating enzyme 5 OS=Mus musculus OX=10090 GN=Uba5 PE=1 SV=2	45 kDa	4
Ubiquitin-like modifier-activating enzyme 6 OS=Mus musculus OX=10090 GN=Uba6 PE=1 SV=1	115 kDa	2
Ubiquitin-like protein 4A OS=Mus musculus OX=10090 GN=Ubl4a PE=1 SV=1	18 kDa	1
Ubiquitin-protein ligase E3A (Fragment) OS=Mus musculus OX=10090 GN=Ube3a PE=1 SV=1	35 kDa	2
Ubiquitinyl hydrolase 1 OS=Mus musculus OX=10090 GN=Usp5 PE=1 SV=1	93 kDa	31
UBX domain-containing protein 1 OS=Mus musculus OX=10090 GN=Ubxn1 PE=1 SV=1	34 kDa	13
UBX domain-containing protein 7 OS=Mus musculus OX=10090 GN=Ubxn7 PE=1 SV=1	55 kDa	5

UDP-glucose 6-dehydrogenase OS=Mus musculus OX=10090 GN=Ugdh PE=1 SV=1	55 kDa	10
UDP-glucose:glycoprotein glucosyltransferase 1 OS=Mus musculus OX=10090 GN=Uggt1 PE=1 SV=4	176 kDa	27
UDP-N-acetylglucosamine--peptide N-acetylglucosaminyltransferase 110 kDa subunit OS=Mus musculus OX=10090 GN=Ogt PE=1 SV=2	117 kDa	1
UDP-N-acetylhexosamine pyrophosphorylase OS=Mus musculus OX=10090 GN=Uap1 PE=1 SV=1	58 kDa	5
UDP-N-acetylhexosamine pyrophosphorylase-like protein 1 OS=Mus musculus OX=10090 GN=Uap1l1 PE=1 SV=1	57 kDa	25
UMP-CMP kinase OS=Mus musculus OX=10090 GN=Cmpk1 PE=1 SV=1	26 kDa	8
Uncharacterized protein C11orf98 homolog OS=Mus musculus OX=10090 PE=1 SV=1	14 kDa	3
Uncharacterized protein C7orf50 homolog OS=Mus musculus OX=10090 PE=1 SV=3	22 kDa	2
Uncharacterized protein KIAA1143 homolog OS=Mus musculus OX=10090 PE=1 SV=1	17 kDa	1
Unconventional myosin-Ic OS=Mus musculus OX=10090 GN=Myo1c PE=1 SV=2	122 kDa	1
UPF0160 protein MYG1, mitochondrial OS=Mus musculus OX=10090 GN=Myg1 PE=1 SV=1	43 kDa	12
UPF0598 protein C8orf82 homolog OS=Mus musculus OX=10090 PE=1 SV=1	24 kDa	1
Up-regulated during skeletal muscle growth protein 5 OS=Mus musculus OX=10090 GN=Usmg5 PE=1 SV=1	6 kDa	5
Uridine 5'-monophosphate synthase OS=Mus musculus OX=10090 GN=Umps PE=1 SV=3	52 kDa	17
Uroporphyrinogen decarboxylase OS=Mus musculus OX=10090 GN=Urod PE=1 SV=2	41 kDa	4
UV excision repair protein RAD23 homolog A (Fragment) OS=Mus musculus OX=10090 GN=Rad23a PE=1 SV=1	35 kDa	4
UV excision repair protein RAD23 homolog B OS=Mus musculus OX=10090 GN=Rad23b PE=1 SV=2	44 kDa	27
Vacuolar protein sorting-associated protein 26A OS=Mus musculus OX=10090 GN=Vps26a PE=1 SV=1	38 kDa	3
Vacuolar protein sorting-associated protein 26B OS=Mus musculus OX=10090 GN=Vps26b PE=1 SV=1	39 kDa	1
Vacuolar protein sorting-associated protein 28 homolog OS=Mus musculus OX=10090 GN=Vps28 PE=1 SV=1	25 kDa	1
Vacuolar protein sorting-associated protein 29 (Fragment) OS=Mus musculus OX=10090 GN=Vps29 PE=1 SV=1	14 kDa	11
Vacuolar protein sorting-associated protein 29 OS=Mus musculus OX=10090 GN=Vps29 PE=1 SV=1	10 kDa	6
Vacuolar protein sorting-associated protein 35 OS=Mus musculus OX=10090 GN=Vps35 PE=1 SV=1	92 kDa	1
Vacuolar protein sorting-associated protein 4B OS=Mus musculus OX=10090 GN=Vps4b PE=1 SV=2	49 kDa	2

Vacuolar protein sorting-associated protein VTA1 homolog (Fragment) OS=Mus musculus OX=10090 GN=Vta1 PE=1 SV=8	28 kDa	1
Vacuolar protein-sorting-associated protein 25 OS=Mus musculus OX=10090 GN=Vps25 PE=1 SV=1	22 kDa	3
Vacuolar protein-sorting-associated protein 25 OS=Mus musculus OX=10090 GN=Vps25 PE=1 SV=1	15 kDa	3
Valine--tRNA ligase OS=Mus musculus OX=10090 GN=Vars PE=1 SV=1	140 kDa	88
Vascular cell adhesion protein 1 OS=Mus musculus OX=10090 GN=Vcam1 PE=1 SV=1	81 kDa	10
Vasodilator-stimulated phosphoprotein OS=Mus musculus OX=10090 GN=Vasp PE=1 SV=4	40 kDa	5
Very long-chain specific acyl-CoA dehydrogenase, mitochondrial OS=Mus musculus OX=10090 GN=Acadvl PE=1 SV=3	71 kDa	8
Vesicle transport protein GOT1B OS=Mus musculus OX=10090 GN=Golt1b PE=1 SV=1	15 kDa	3
Vesicle transport protein SEC20 OS=Mus musculus OX=10090 GN=Bnip1 PE=1 SV=1	22 kDa	1
Vesicle transport through interaction with t-SNAREs 1B homolog OS=Mus musculus OX=10090 GN=Vti1b PE=1 SV=1	27 kDa	1
Vesicle-associated membrane protein 3 OS=Mus musculus OX=10090 GN=Vamp3 PE=1 SV=1	11 kDa	6
Vesicle-associated membrane protein 4 OS=Mus musculus OX=10090 GN=Vamp4 PE=1 SV=1	16 kDa	1
Vesicle-associated membrane protein 8 OS=Mus musculus OX=10090 GN=Vamp8 PE=1 SV=1	11 kDa	5
Vesicle-associated membrane protein, associated protein B and C OS=Mus musculus OX=10090 GN=Vapb PE=1 SV=1	27 kDa	17
Vesicle-associated membrane protein-associated protein A OS=Mus musculus OX=10090 GN=Vapa PE=1 SV=2	28 kDa	45
Vesicle-trafficking protein SEC22b OS=Mus musculus OX=10090 GN=Sec22b PE=1 SV=1	19 kDa	2
Vesicular integral-membrane protein VIP36 OS=Mus musculus OX=10090 GN=Lman2 PE=1 SV=2	40 kDa	8
Vigilin OS=Mus musculus OX=10090 GN=Hdlbp PE=1 SV=1	142 kDa	24
Vimentin OS=Mus musculus OX=10090 GN=Vim PE=1 SV=3	54 kDa	124
Vinculin OS=Mus musculus OX=10090 GN=Vcl PE=1 SV=4	117 kDa	122
Vitamin K epoxide reductase complex subunit 1 OS=Mus musculus OX=10090 GN=Vkorc1 PE=1 SV=1	18 kDa	1
Voltage-dependent anion-selective channel protein 1 OS=Mus musculus OX=10090 GN=Vdac1 PE=1 SV=1	28 kDa	7
Voltage-dependent anion-selective channel protein 1 OS=Mus musculus OX=10090 GN=Vdac1 PE=1 SV=3	32 kDa	10
Voltage-dependent anion-selective channel protein 2 OS=Mus musculus OX=10090 GN=Vdac2 PE=1 SV=2	32 kDa	16

Voltage-dependent anion-selective channel protein 3 OS=Mus musculus OX=10090 GN=Vdac3 PE=1 SV=1	31 kDa	10
von Willebrand factor A domain-containing protein 5A OS=Mus musculus OX=10090 GN=Vwa5a PE=1 SV=2	87 kDa	2
V-type proton ATPase catalytic subunit A OS=Mus musculus OX=10090 GN=Atp6v1a PE=1 SV=2	68 kDa	27
V-type proton ATPase subunit B, brain isoform OS=Mus musculus OX=10090 GN=Atp6v1b2 PE=1 SV=1	57 kDa	10
V-type proton ATPase subunit D OS=Mus musculus OX=10090 GN=Atp6v1d PE=1 SV=1	28 kDa	2
V-type proton ATPase subunit E 1 (Fragment) OS=Mus musculus OX=10090 GN=Atp6v1e1 PE=1 SV=1	9 kDa	2
V-type proton ATPase subunit E 1 OS=Mus musculus OX=10090 GN=Atp6v1e1 PE=1 SV=2	26 kDa	2
V-type proton ATPase subunit G 1 OS=Mus musculus OX=10090 GN=Atp6v1g1 PE=1 SV=3	14 kDa	9
V-type proton ATPase subunit H OS=Mus musculus OX=10090 GN=Atp6v1h PE=1 SV=1	51 kDa	1
Wapal protein OS=Mus musculus OX=10090 GN=Wapl PE=1 SV=1	133 kDa	2
WAS/WASL-interacting protein family member 1 OS=Mus musculus OX=10090 GN=Wipf1 PE=1 SV=1	50 kDa	3
WASH complex subunit 1 OS=Mus musculus OX=10090 GN=Washc1 PE=1 SV=1	52 kDa	1
WASH complex subunit 2 OS=Mus musculus OX=10090 GN=Washc2 PE=1 SV=1	145 kDa	4
WD repeat and HMG-box DNA-binding protein 1 OS=Mus musculus OX=10090 GN=Wdhd1 PE=1 SV=1	120 kDa	3
WD repeat-containing protein 1 OS=Mus musculus OX=10090 GN=Wdr1 PE=1 SV=3	66 kDa	57
WD repeat-containing protein 18 OS=Mus musculus OX=10090 GN=Wdr18 PE=1 SV=1	47 kDa	1
WD repeat-containing protein 37 OS=Mus musculus OX=10090 GN=Wdr37 PE=1 SV=1	55 kDa	1
WD repeat-containing protein 41 OS=Mus musculus OX=10090 GN=Wdr41 PE=1 SV=1	35 kDa	1
WD repeat-containing protein 43 OS=Mus musculus OX=10090 GN=Wdr43 PE=2 SV=2	75 kDa	2
WD repeat-containing protein 44 OS=Mus musculus OX=10090 GN=Wdr44 PE=1 SV=1	102 kDa	1
WD repeat-containing protein 48 OS=Mus musculus OX=10090 GN=Wdr48 PE=1 SV=1	76 kDa	1
WD repeat-containing protein 5 OS=Mus musculus OX=10090 GN=Wdr5 PE=1 SV=1	37 kDa	7
WD repeat-containing protein 61 OS=Mus musculus OX=10090 GN=Wdr61 PE=1 SV=1	34 kDa	5
WD repeat-containing protein 82 OS=Mus musculus OX=10090 GN=Wdr82 PE=1 SV=1	35 kDa	1
WD repeat-containing protein 89 OS=Mus musculus OX=10090 GN=Wdr89 PE=2 SV=1	42 kDa	1

WD repeat-containing protein 92 OS=Mus musculus OX=10090 GN=Wdr92 PE=1 SV=1	40 kDa	3
WD40 repeat-containing protein SMU1 OS=Mus musculus OX=10090 GN=Smu1 PE=2 SV=2	58 kDa	13
Wiskott-Aldrich syndrome protein family member 2 OS=Mus musculus OX=10090 GN=Wasf2 PE=1 SV=1	54 kDa	6
WW domain-binding protein 11 OS=Mus musculus OX=10090 GN=Wbp11 PE=1 SV=2	70 kDa	8
WW domain-binding protein 2 OS=Mus musculus OX=10090 GN=Wbp2 PE=1 SV=1	28 kDa	1
Xaa-Pro dipeptidase OS=Mus musculus OX=10090 GN=Pepd PE=1 SV=3	55 kDa	9
Xanthine dehydrogenase/oxidase OS=Mus musculus OX=10090 GN=Xdh PE=1 SV=5	147 kDa	5
X-prolyl aminopeptidase (Aminopeptidase P) 1, soluble, isoform CRA_b OS=Mus musculus OX=10090 GN=Xpnpep1 PE=1 SV=1	75 kDa	5
X-ray repair cross-complementing protein 6 OS=Mus musculus OX=10090 GN=Xrcc6 PE=1 SV=1	69 kDa	1
Y-box-binding protein 3 OS=Mus musculus OX=10090 GN=Ybx3 PE=1 SV=2	39 kDa	32
YLP motif-containing protein 1 OS=Mus musculus OX=10090 GN=Ylpm1 PE=1 SV=2	241 kDa	2
YTH domain-containing family protein 2 OS=Mus musculus OX=10090 GN=Ythdf2 PE=1 SV=1	62 kDa	2
YTH domain-containing family protein 3 OS=Mus musculus OX=10090 GN=Ythdf3 PE=1 SV=2	64 kDa	4
YY1-associated factor 2 OS=Mus musculus OX=10090 GN=Yaf2 PE=1 SV=1	20 kDa	1
Zinc finger C2HC domain-containing protein 1A OS=Mus musculus OX=10090 GN=Zc2hc1a PE=1 SV=1	35 kDa	1
Zinc finger CCCH domain-containing protein 11A OS=Mus musculus OX=10090 GN=Zc3h11a PE=1 SV=1	86 kDa	1
Zinc finger CCCH domain-containing protein 14 OS=Mus musculus OX=10090 GN=Zc3h14 PE=1 SV=1	82 kDa	3
Zinc finger CCCH domain-containing protein 15 OS=Mus musculus OX=10090 GN=Zc3h15 PE=1 SV=2	48 kDa	7
Zinc finger CCCH domain-containing protein 4 OS=Mus musculus OX=10090 GN=Zc3h4 PE=1 SV=1	127 kDa	2
Zinc finger matrin type 3 OS=Mus musculus OX=10090 GN=Zmat3 PE=1 SV=1	32 kDa	1
Zinc finger MIZ domain-containing protein 1 OS=Mus musculus OX=10090 GN=Zmiz1 PE=1 SV=1	116 kDa	1
Zinc finger protein 706 OS=Mus musculus OX=10090 GN=Znf706 PE=1 SV=1	8 kDa	2
Zinc finger protein 830 OS=Mus musculus OX=10090 GN=Znf830 PE=1 SV=1	41 kDa	1
Zinc finger protein 830 OS=Mus musculus OX=10090 GN=Znf830 PE=1 SV=1	41 kDa	1

Zinc finger protein ubi-d4 OS=Mus musculus OX=10090 GN=Dpf2 PE=1 SV=1	46 kDa	2
Zinc finger protein ZPR1 (Fragment) OS=Mus musculus OX=10090 GN=Zpr1 PE=1 SV=1	42 kDa	3
Zinc finger protein-like 1 (Fragment) OS=Mus musculus OX=10090 GN=Zfpl1 PE=1 SV=1	15 kDa	1
Zinc finger Ran-binding domain-containing protein 2 OS=Mus musculus OX=10090 GN=Zranb2 PE=1 SV=2	36 kDa	4
Zinc finger RNA-binding protein OS=Mus musculus OX=10090 GN=Zfr PE=1 SV=2	117 kDa	1
Zinc phosphodiesterase ELAC protein 2 OS=Mus musculus OX=10090 GN=Elac2 PE=1 SV=1	92 kDa	7
Zinc transporter ZIP10 OS=Mus musculus OX=10090 GN=Slc39a10 PE=1 SV=1	94 kDa	1
ZW10 interactor OS=Mus musculus OX=10090 GN=Zwint PE=1 SV=1	29 kDa	1
Zyx protein OS=Mus musculus OX=10090 GN=Zyx PE=1 SV=1	57 kDa	38
